# Proteomic and transcriptomic characterisation of FIA10, a novel murine leukemic cell line that metastasizes into the brain

**DOI:** 10.1371/journal.pone.0295641

**Published:** 2024-01-12

**Authors:** Ursula Just, Helmut Burtscher, Sylvia Jeratsch, Meike Fischer, Carol Stocking, Jens Preussner, Mario Looso, Ralf Schwanbeck, Stefan Günther, Ralf Huss, Lynne Mullen, Thomas Braun

**Affiliations:** 1 Department of Cardiac Development and Remodeling, Max-Planck-Institute for Heart and Lung Research, Bad Nauheim, Germany; 2 Leibniz Institute for Virology, Hamburg, Germany; 3 Department of Biochemistry, Christian-Albrechts-University zu Kiel, Kiel, Germany; 4 Pharma Research Penzberg, Roche Diagnostics GmbH, Penzberg, Germany; 5 Biomolecular Mass Spectrometry, Max Planck Institute for Heart and Lung Research, Bad Nauheim, Germany; 6 Bioinformatics Core Unit, Max Planck Institute for Heart and Lung Research, Bad Nauheim, Germany; 7 QIAGEN, Redwood City, California, United States of America; NCI: National Cancer Institute, UNITED STATES

## Abstract

Brain metastasis leads to increased mortality and is a major site of relapse for several cancers, yet the molecular mechanisms of brain metastasis are not well understood. In this study, we established and characterized a new leukemic cell line, FIA10, that metastasizes into the central nervous system (CNS) following injection into the tail vein of syngeneic mice. Mice injected with FIA10 cells developed neurological symptoms such as loss of balance, tremor, ataxic gait and seizures, leading to death within 3 months. Histopathology coupled with PCR analysis clearly showed infiltration of leukemic FIA10 cells into the brain parenchyma of diseased mice, with little involvement of bone marrow, peripheral blood and other organs. To define pathways that contribute to CNS metastasis, global transcriptome and proteome analysis was performed on FIA10 cells and compared with that of the parental stem cell line FDCP-Mix and the related FIA18 cells, which give rise to myeloid leukemia without CNS involvement. 188 expressed genes (RNA level) and 189 proteins were upregulated (log2 ratio FIA10/FIA18 ≥ 1) and 120 mRNAs and 177 proteins were downregulated (log2 ratio FIA10/FIA18 ≤ 1) in FIA10 cells compared with FIA18 cells. Major upregulated pathways in FIA10 cells revealed by biofunctional analyses involved immune response components, adhesion molecules and enzymes implicated in extracellular matrix remodeling, opening and crossing the blood-brain barrier (BBB), molecules supporting migration within the brain parenchyma, alterations in metabolism necessary for growth within the brain microenvironment, and regulators for these functions. Downregulated RNA and protein included several tumor suppressors and DNA repair enzymes. In line with the function of FIA10 cells to specifically infiltrate the brain, FIA10 cells have acquired a phenotype that permits crossing the BBB and adapting to the brain microenvironment thereby escaping immune surveillance. These data and our model system FIA10 will be valuable resources to study the occurrence of brain metastases and may help in the development of potential therapies against brain invasion.

## Introduction

CNS involvement has an incidence of 6–29% at diagnosis of pediatric acute myeloid leukemia and CNS relapses are more frequent in patients with initial CNS involvement [1]. Although the frequency of CNS leukemia in adult acute myeloid leukemia is generally low (0.6% at first diagnosis and 2.9% at relapse), it is associated with poor prognosis [2]. Current therapies include intrathecal and systemic chemotherapy and/or cranial irradiation, which are, however, associated with severe side effects, such as secondary cancers, endocrine disorders, neurocognitive deficits and growth impairment in childhood leukemia [1]. To develop better treatments and diagnostic measures, experimental models are needed that recapitulate the complex process of central nervous metastasis.

Little is known about how leukemic or other tumor cells enter the CNS. The BBB insulates the CNS from circulation, forming a highly exclusive barrier against transit of cells into the brain (reviewed by [3]). After passage of the BBB, a dynamic interaction between the tumor cells and the brain microenvironment takes place, establishing a metastatic niche, which is a critical regulator of cancer progression and therapeutic efficacy in primary and metastatic brain malignancies (reviewed by [4].

Here, we investigated and compared the gene and protein expression profiles of two clonal leukemic cell lines derived from a murine hematopoietic progenitor cell line (FDCP-Mix) that after injection into the tail vein of syngeneic mice result either in myeloid leukemia (FIA18 cells [5]) or brain metastasis (FIA10 cells, this study), in contrast to the parental cells that contribute to normal blood development (FDCP-Mix [6]). Analysis of the differences in the proteome and transcriptome identified several proteins and molecular pathways that may participate in the underlying basis of leukemic metastasis to the CNS.

## Material and methods

### Cell lines

Cell lines were maintained in Iscove’s modified Dulbecco’s medium (IMDM) supplemented with 20% preselected horse serum (HS) and, where indicated, mouse IL-3 conditioned medium corresponding to 100 U rIL-3 per ml [5, 7, 8]. Cells were kept at a density of between 6x10^4^ to 1x10^6^ cells/ml and were regularly checked to be free of mycoplasma contamination by PCR. The clonal cell lines used in this study were:

- FDCP-Mix is a well-characterized clonal hematopoietic progenitor cell line derived from long term hematopoietic cultures from male BDF1 mice [6, 9]. When injected into the tail vain of irradiated syngeneic BDF1 mice, FDCP-Mix cells contribute to the reconstitution of the hematopoietic system [6]. FDCP-Mix cells exhibit a predominant blast cell morphology when maintained in the presence of IL-3 and HS, whereas when exposed to fetal calf serum (FCS) differentiation into all myeloid lineages is observed [6].
- GMV-FDCP-Mix cell clones were established from FDCP-Mix cells after retroviral-mediated transduction with vectors coexpressing murine GM-CSF (granulo-macrophage stimulating factor) and the G418-resistance gene neo [8]. Transduced clones were selected in the presence of G418 (1 mg/ml) and IL-3 in soft agar, isolated after 9–14 days, and expanded in IMDM with 20% HS and IL-3 and 1 mg/ml G418 [8]. Removal of IL-3 results in synchronized myeloid differentiation of the cultures mediated by the endogenous expression of GM-CSF [8].
- FIA18 and FIA10 cells are two clonal variants of GMV-FDCP-Mix that were selected by cloning assays performed on GMV-FDCP-Mix cells cultivated in IL-3 free medium at a cell density of 4x10^5^ cells/ml for two weeks [5]. Cells of the plucked clones were expanded and their capacity for proliferation, clonability, morphology and differentiation capacity in the absence of IL-3 was extensively examined [5]. Several independent clones were tested for leukemogenicity by injection into the tail vein of syngeneic female BDF1 mice and subsequent analyses for presence of male specific sequences in hematopoietic tissues of injected mice [5]. Clones FIA18 and FIA10 were selected for further studies, as outlined in results.

Specific growth conditions (e.g. G418 and HS), clonability, morphology, and differentiation capacity of the clonal cell lines excludes the presence of contaminating cells.

### Characterization of cells

For morphological characterisation of cells, suspension cultures were concentrated on slides by cytocentrifugation (Cytospin, Shandon). The cells were stained with May-Grünwald-Giemsa. To assay differentiation capacity, cells were washed and cultured in IMDM supplemented with 20% pretested FCS, 5 U/ml rIL-3, and 2 U/ml erythropoietin [5]. After 7 to 10 days of incubation, cytospin preparations were made and the cells stained as described above. For in vitro colony-forming cell determinations, cells were plated in soft agar in IMDM supplemented with 20% HS in the absence of IL-3. After 7 to 10 days of incubation, colonies of more than 50 cells were scored. Cultures were incubated in a fully humified atmosphere of 5% CO_2_, 5% O_2_ in N_2_.

### Animal experiments and histopathology

All animal experiments were conducted in accordance with approved protocols and the requirements of the German Animal Welfare Act. Animal experiments and protocols were approved with written permission by local animal committees in Hamburg and Kiel (Ministerium für Landwirtschaft, Umwelt und ländliche Räume des Landes Schleswig-Holstein, Germany, permit no. V 312–72241.121–3 36-3/07).

Mice were kept on a C57BL/6 background and housed under pathogen-free conditions at room temperature under a 12 h light-dark cycle with free access to food and water. In 5 independent experiments 113 female mice (8–20 weeks) with a body weight of 18–24 g and 23 male mice (8–20 weeks) with a body weight of 22–35 g were randomly distributed into designated groups. B6D2F1/J mice were bred in the animal facility of the HPI Leibniz Institute for Virology, Hamburg, Germany, animal facility of the Christian-Albrechts-University zu Kiel, Germany or purchased from Charles River Laboratories. In total, 136 mice were included in this study.

Cells cultured in IMDM supplemented with 20% HS and with or without IL3 or GM-CSF were washed twice in IMDM and resuspended in PBS. 5x10^6^ cells in 0.5 ml PBS were injected into the tail vein of syngeneic B6D2F1 mice that had received a sublethal dose of irradiation (4 Gy Cobalt-60 source, dose rate 1 Gy/h) or were left untreated [5]. Mice were inspected every 1–2 days for the following signs of morbidity: loss of balance, ataxic gait, tremor, uninduced as well as induced seizures as signs for CNS metastasis and weight loss, fatigue or infections as signs for leukemia. Hind limb paralysis occured in both groups additionally to either CNS or leukemic symptoms. Animals were euthanized by cervical dislocation upon showing any signs of disease.

Organs were inspected macroscopically at autopsy. Spleen sizes were determined. Femurs were dissected and bone marrow was flushed out into IMDM supplemented with 10% FCS. Cells were counted and spun onto slides at 1000 rpm using a microspin cytocentrifuge (Shandon, USA) and stained with May-Grünwald-Giemsa. Peripheral blood was taken up with a heparinised syringe, blood smears were prepared and stained with May-Grünwald Giemsa. Peripheral blood was subjected to hemolysis by incubation for 5 min in 1 ml hemolysis buffer (155 mM NH_4_Cl, 10 mM KHCO_3_ 0.08 mM EDTA-Titriplex pH 7.4) at RT. Subsequently, cells from bone marrow and peripheral blood were centrifuged by 400xg for 10 min, resuspended in 500 μl Tens lysis buffer and DNA was extracted from the cell pellets as described below.

For pathological analysis of brains simultaneously with determination of y-chromosome sequences, whole brains were dissected unfixed, sectioned sagittally with 2 mm slices, examined, samples taken for DNA extraction to be used for y-chromosome specific PCR and the adjacent slice(s) fixed overnight in 4% buffered formaldehyde. These slices were embedded in paraplast (Sherwood Medical Industries, St. Louis, Missouri, USA), sectioned at 3 μm, placed on microscope slides, stained with hematoxylin and eosin and examined for leukemic infiltration.

In one experiment with 23 male and 12 female BDF1 mice, animals were anaesthetized with pentobarbital, then injected with saline into the right ventricle and subsequently perfused with 4% PFA for fixation. For histologic inspection of brain, animals were decapitated, skull preparated and after opening the foramen magnum the brain was further fixed in by Calfix (Quaratett), a formaline containing solution, for 1 week at 4°C, followed by decalcification for 10 days by Calless (Quaratett) before paraffin embedding. Embedded brains were sectioned sagittally into 3 levels and sectioned at 3 μm, placed on microscope slides, stained with hematoxylin and eosin and examined for leukemic infiltration.

### Determination of male-specific sequences

Genomic DNA was extracted from crushed tissue pieces by digestion with freshly added 800 μg/ml Proteinase K in 3 ml Tens lysis buffer (10 mM Tris-HCl pH 8.0, 25 mM EDTA pH 8.0, 100 mM NaCl, 0.5% SDS) per tissue sample overnight at 56°C with shaking [10]. After debris removal by centrifugation at 5000 rpm, genomic DNA in the supernatant was precipitated with an equal amount of Isopropanol and subsequent centrifugation at 13000 rpm for 20 min at 4°C and washing twice with 80% EtOH. After air drying for 10 min, the DNA was dissolved in TE buffer pH 8.0 by incubation at 37°C for 2 hrs. In the first set of FIA18 and FIA10 injected mice, DNA was extracted using the Phenol:Cloroform method (Isolation of High- Molecular-Weight DNA using organic solvents, Sambrook, J., Fritsch, E.F., Maniatis, T.: Molecular Cloning: A Laboratory Manual, 2^nd^ ed., Cold Spring Harbour Laboratory Press, Cold Spring Harbour, New York 1989).

The 260/280 absorbance ratio of the DNAs used for subsequent PCR was between 1.8–2.0. The DNA yield was quantified spectrophotometrically by absorbance at 260. PCR was used to detect male-specific sequences in genomic DNA of female mice injected with male FIA10 cells. As primers for y-specific PCR sense: cactattttcccagtggtctgtgaa and antisense: aaagtacacggaaggattggctaga were used [11]. Cycling parameters were as follows: a hot start at 94°C for 2 minutes, denaturing at 94°C for 1 minute, annealing at 67°C for 2 minutes, and elongation at 72°C for 3 minutes, repeated for 30 cycles and followed by elongation at 72°C for 10 minutes. Standard curves were generated by serially diluting male mouse genomic DNA from FIA10 cells into female mouse genomic DNA prepared from brain. The presence of y-PCR amplified sequences was confirmed by Southern Blotting with pY353/B [11]. Detection limit of the semiquantitative PCR was 0.1% of male sequences in a female background. Determination of y-specific sequences was quantified with real-time PCR (n = 12) using SYBR Green qPCR Select Master Mix (Applied Biosystems) with primers sense: gttttgggactggtgacaattg and antisense: gtcttgcctgtatgtgg [12]. Detection limit of the real-time PCR was 0,03% (1.6 pg) of male sequences in a female background (5000 pg).

### Microarrays

Frozen vials from time points, when the cells injected into syngeneic mice showed the respective phenotype, i.e. brain leukemia for FIA10 and myeloid leukemia for FIA18, and of FDCP-Mix control cells were thawed for microarray analyses. After establishing log-phase growth, 1x10^7^ cells at a cell density of 5x10^5^/ml from 3 independent cultures of FIA10, FIA18 cells and FDCP-Mix control cells, respectively, were harvested. Morphology of the cultured cells showed the respective phenotype, blast cells with myeloid differentiation for FIA10 and FIA18, and blast cells for FDCP-Mix. RNA from the different cell populations was isolated using RNeasy Midi Kit (Qiagen). A_260/280_ ratios were between 2.0–2.1 and 18S and 28S ribosomal RNA bands clearly visible for all RNAs isolated. 5 μg of total RNA was used for cDNA synthesis (Expression Analysis Technical Manual, Affymetrix, Santa Clara, CA, USA). cRNA was generated (BioArray High-Yield Transcript Labeling kit, ENZO, Farmingdale, NY, USA) and hybridized to Affymetrix GeneChip Mouse Genome 430 2.0 Array (15 μg cRNA, 16 h, 45°C) that contain ∼45,000 probe sets of over 39,000 murine transcripts. GeneChip arrays were stained, washed and scanned according to the manufacturer’s specifications.) Analyses were performed with dChip Software. Principle component analysis (PCA) was performed using the R software (http://www.r-project.org/). Scanned GeneChip.DAT files were analyzed by dChip Software (Built date Aug 21, 2008;(http://dchip.org). Relative expression was visualized via heatmap using the MeV 4.2 of the TM4 software suite (http://www.tm4.org/; [13]). Values were Log2 transformed and normalized within each gene. Furthermore, genes were classified into Gene Ontology [14] or PANTHER groups [15] using the DAVID functional annotation analysis tool (http://david.abcc.ncifcrf.gov/). The GO and PANTHER terms used were: GOTERM_BP_ALL: GO:0006350∼transcription OR PANTHER_MF_ALL: MF00036: Transcription factor (‘Transcription and TFs’); GOTERM_BP_ALL: GO:0006915∼apoptosis; GOTERM_BP_ALL: GO:0030154∼cell differentiation; GOTERM_BP_ALL: GO:0007049∼cell cycle; GOTERM_BP_ALL: GO:0007154∼cell communication; GOTERM_BP_ALL: GO:0007155∼cell adhesion; GOTERM_BP_ALL: GO:0007242∼intracellular signaling cascade; GOTERM_BP_ALL: GO:0019222∼regulation of metabolic process; GOTERM_CC_ALL: GO:0005856∼cytoskeleton; GOTERM_BP_ALL: GO:0008283∼cell proliferation; PANTHER_BP_ALL: BP00248:Mesoderm development. Genes of the groups were reassigned to their induction values and visualized as a heatmap with the MeV 4.2 software.

### qRT-PCR / real-time RT-PCR

Total RNA was isolated using RNeasy Mini Kit (Qiagen) and then reverse transcribed using Reverse Transcriptase Kit (ThermoFisher). Relative expression levels were determined by real-time PCR on a 7900HT Fast Real-Time PCR System (Applied Biosystems) in 384-well PCR-plates (ABgene) using the TaqMan Gene Expression Assays-on-Demand system (Applied Bioystems, Foster City, USA) as described previously [16] with minor changes. Primers used in qRT-PCR analyses are summarized in S1 Table. The relative expression of mRNAs was quantified by the ΔCt method with efficiency values measured in a pilot experiment for each expression assay. Relative expressions were visualized via heatmaps using MeV 4.2 software.

### Protein mass spectrometry

Cells were grown in SILAC medium for at least five cell divisions as described previously to ensure complete labeling of cells [17]. Cells were washed 6 times in serum-free medium, and incubated in serum-free medium. The cells were lysed in SDS buffer (4% SDS in 0.1 M Tris/HCl ph 7.6) before heating the samples at 95°C for 5 min. DNA was sheared by sonication and cell debris was removed by centrifugation at 16.000 g for 10 min. DC protein assay (BioRad, Hercules, CA) was used to determine the concentration of the solubilized proteins in supernatants after centrifugation. Equal amounts of proteins (40 μg each) were loaded on a gradient Bis-Tris gel (4–12%, Novex) and separated by SDS-PAGE before in-gel digestion [18]. Proteins in the gel were fixed and stained with coomassie solution (Instantblue, Expedeon) before cutting each lane into ten slices. Gel slices were destained, washed and dehydrated with ethanol and 50mM ammonium bicarbonate. Proteins were reduced and alkylated with 10 mM dithiotreitol (Sigma) and 55 mM iodoacetamide (Sigma), respectively. Proteolytic digestion was carried out with 12.5 ng/μl trypsin solution (Promega, Mannheim, Germany) overnight. Obtained peptides were subsequently extracted in five steps with increasing concentrations of acetonitrile. Peptide fractions were pooled, concentrated in a vacuum concentrator and desalted via the stop and go extraction technique [19].

For liquid chromatography and mass spectrometry (MS) analysis, the LC-MS-system consisting of a nano-flow HPLC system (Easy nLC 1000; Thermo Fisher Scientific) connected to a QExactive HF mass spectrometer (ThermoFisher Scientific) via a nano electrospray ionization source (ThermoFisher Scientific) was used. Peptides were separated according to their hydrophobicities on a 15 cm in-house packed RP-column (ID 75μm; C-18-beads, diameter 3μm, Dr. Maisch GmbH, Ammerbuch, Germany) with a binary solvent system (solvent A: 0.5% acetic acid; solvent B: 80% acetonitrile, 0.1% formic acid). Peptide elution was achieved by increasing the relative amount of B from 10% to 38% in a linear gradient within 50 min, followed by 5 min up to 60% and another 5 min to 95%. Re-equilibration was done within 2 min with 5% of solvent B.

Mass spectra were acquired with a data dependent Top15 method with a resolution of 60,000 at 200 m/z and AGC target of 3x10^6^ at a maximum injection time of 20 ms. The 15 most intense peaks were further fragmented with higher energy collisional dissociation (HCD) and MS² spectra were generated at 15,000 resolution, AGC target of 1x10^5^ with a maximum injection time of 25 ms.

### Proteomic data analyses

80 raw files of controls and mutants in duplicates were analyzed using MaxQuant (v1.5.3.12 [20]) and the implemented Andromeda search engine [21]. Protein assignment was accomplished with correlation of fragment spectra with the Uniprot mouse database (2016). A list of common contaminants was used to exclude these from the analysis. Searches were performed with tryptic specifications and settings for mass tolerance for MS and MS/MS spectra as default. Carbamidomethylation at cysteine residues was set as fixed modification together with oxidation of methionine, acetylation at the N-terminus and phosphorylation of serine, threonine and tyrosine as variable modifications. The minimal peptide length was set to 7 amino acids by default and the false discovery rate on protein and peptide level was 1%. Matching between runs was enabled for the analysis. Prior to further processing of the data, contaminants, reverse entries and proteins that were only identified by a modification site were filtered out. Gene Ontology (GO) annotations for biological process, molecular function and cellular compartments as well as Kyoto Encyclopedia of Genes and Genomes (KEGG) annotation and Pfam annotation for protein families were performed with Perseus (v1.5.0.31). For all comparisons, median values of the two replicates of each condition were used.

### Bioinformatics and statistical analysis

For Venn diagrams the program Venny was applied [22]. Venn diagrams were generated to display intersecting or non-intersecting groups of differentially expressed RNA or protein. The Gene Ontology (GO) enrichment tool [23, 24] and QIAGEN Ingenuity Pathway Analysis (QIAGEN IPA, https://www.qiagen.com) software (QIAGEN, Redwood City, CA, USA) were employed to group differentially expressed RNA and protein into functional categories [25]. Statistical analysis for RNA and protein expression data T-Test was used. The p-value for GO analyses was computed according to the mHG or HG model. The Diseases & Functions QIAGEN IPA Analysis identified the biological functions and/or diseases that were most significant from the data set. Molecules from the dataset that met the p-value cutoff of 0.05 and log2ratio cutoff of ≥ 1.0 or ≤ 1.0 and were associated with biological functions and/or diseases in the QIAGEN Knowledge Base were considered for the analysis. A right-tailed Fisher’s Exact Test was used to calculate a p-value determining the probability that each biological function and/or disease assigned to that data set is due to chance alone.

## Results

### Mice injected with FIA10 cells into the tail vein develop CNS leukemia

We have previously reported a multipotent cell line, GMV-FDCP-Mix, in which self-renewing cell growth is maintained by IL-3, whereas autocrine stimulation by GM-CSF induces differentiation along the granulocyte-macrophage pathway and clonal extinction [8]. Neither these cells nor the parental FDCP-Mix cells are tumorigenic in vivo. FIA mutant cell clones that escape clonal extinction upon IL3 removal can be isolated from GMV-FDCP-Mix cells at a low incidence [5]. In contrast to GMV-FDCP-Mix cells, the FIA mutants studied to date induce myeloid leukemia when transplanted into sublethally irradiated mice [5] and unpublished.

In this study, we characterize mutant cell line FIA10, which induced strikingly different symptoms in syngenic mice after injection into the tail vein. In contrast to mice injected with mutant FIA18, which developed myeloid leukemia, all mice injected with FIA10 cells (115 mice in 5 independent experiments) experienced neurological failure 2 to 11 weeks after injection, characterized by loss of balance, ataxic gait, tremor and seizures, leading to death within 3 months.

**Fig 1:**
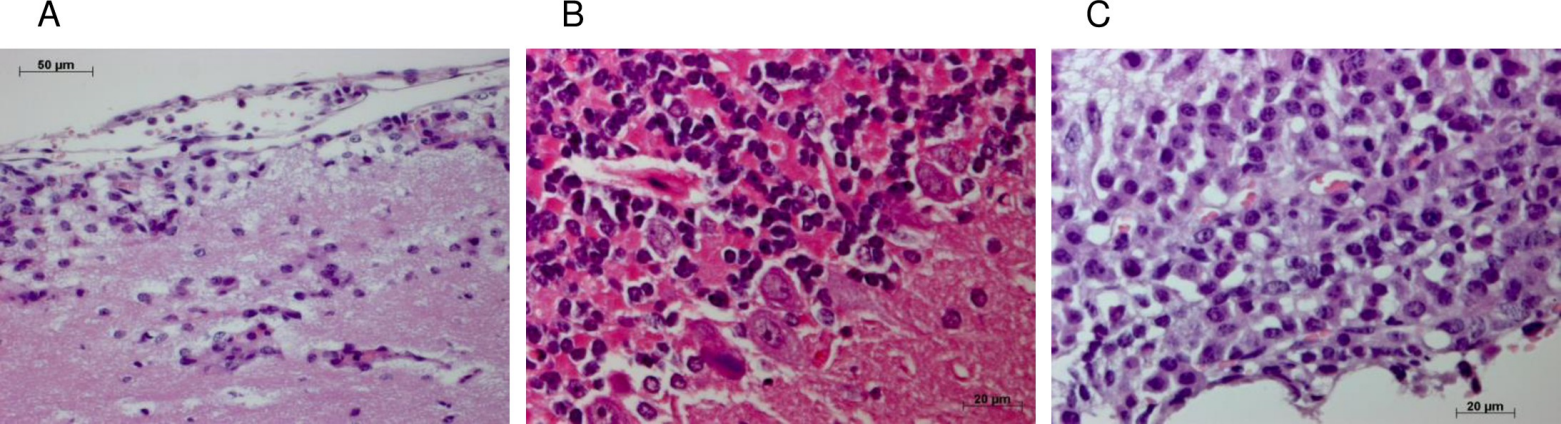
FDCP-Mix derived leukemic cell line FIA10 invades the brain after injection into the tail vein. A-C: Five months old female BDF1 mice 15 days after injection of 5 x 10^6^ FIA10 cells into the tail vein were analysed. Shown are histological examples of three different mouse brains. Clinical signs of these diseased animals were a clear ataxia and inducible, generalized seizures. (A) Mononuclear, hematopoietic cells including several cells with immature blast morphology infiltrate the stratum moleculare of the CNS from the subarachnoid cavity and penetrating leptomenigeal vessels. (B) Infiltrating hematopoietic cells clearly differ from the neural stratum granulosum cells of the cerebellum by their eosinophilic cytoplasm and partially segmented, dark nuclei and seem to be connected to the perivascular space. Large Purkinje cells are also present separating the molecular from the granular layer of the cerebellar cortex. (C) Nodular, partially already organized leukemic infiltrate of the leptomeninx and the subarachnoid cavity of a cistern at the base of the brain. Staining: HE.

Microscopic examination of brains of FIA10 transplanted mice showing neurological symptoms revealed focal leukemic infiltration of the brain parenchyma and hemorrhagias (Fig 1A–1C). Leukemic infiltration of the brain parenchyma consisted of immature blasts and myeloid differentiated cells (Fig 1A–1C). Spleens of FIA10 injected mice varied considerably in size, with a median of 360 mg. Peripheral blood and bone marrow cellularity were within normal range (Table 1) and no obvious alterations in other organs except the CNS were observed. In contrast, control mice injected with leukemic FIA18 cells developed a myeloid leukemia with enlarged spleen (460-1120mg), a marked increase in in cellularity in peripheral blood (4-15x10^7^/ml), and a depression of bone marrow cellularity (1-5x10^6^/ml) in line with previous results [5]. Differential cell counts performed on stained smears from FIA18 transplanted mice revealed a marked elevation of myeloblasts, immature and mature neutrophils in peripheral blood and an increase in myeloblasts, monocytes and macrophages in stained bone marrow cytospin preparations (Table 1). Mice injected with FDCP-Mix control cells (n = 17) showed no evidence of disease over a 3 months observation period as described previously [5]. To exclude that the low dose of irradiation of the animals prior to the administration of FIA10 cells allows the cells to transit the BBB, FIA10 cells were injected into unirradiated recipients (8 mice). Irrespective of prior irradiation or not, all mice succumbed to neurological disease within 3 months and leukemic cells were found in the brain parenchyma. The origin of leukemic cells growing in the brain of diseased mice injected with FIA10 cells was determined assessing the presence of Y-chromosome specific sequences. In all diseased female mice analysed, brain samples were clearly positive for y-specific sequences at variable levels ranging from 0.1% to 0.7%, while Y-chromosome specific sequences were not detected in peripheral blood or bone marrow.

**Table 1:** Differential analysis of blood and bone marrow from sublethally irradiated mice injected with mutants FIA10 and FIA18.

Cell injected	Mouse #	Tissue	Bl	EG	LG	Mo	Ebl	Ly
FIA10	1	PB	0	0	28	4	0	68
	2		0	0	30	0	0	70
	3		0	0	24	4	0	72
FIA10	1	BM	2	19	49	5	20	5
	2		3	17	50	4	17	4
	3		3	16	45	1	34	1
FIA18	1	PB	19	31	49	0	1	5
	2		24	26	44	0	0	6
	3		10	27	63	0	0	0
FIA18	1	BM	3	22	22	51	1	1
	2		0	10	20	64	6	0
	3		0	9	18	66	7	0
FDCP-Mix	1	PB	0	0	25	2	0	73
	2		0	0	27	4	0	69
	3		0	0	24	3	0	73
FDCP-Mix	1	BM	2	23	39	3	24	8
	2		3	18	52	4	16	7
	3		3	14	43	2	30	5

31 mice injected with FIA10 and 8 mice injected with FIA18 cells were examined at onset of disease. 6 mice injected with FDCP-Mix cells were used as controls. The data are from three representative mice. PB = peripheral blood; BM = bone marrow; Bl = primitive blast cells; EG = early granulocytes (promyelocytes and myelocytes); LG = late granulocytes (metamyelocytes and mature granulocytes); Mono = monocytes and macrophages; Ebl = nucleated erythroid cells; Ly = lymphocytes.

### *In vitro* comparison of brain tropic FIA10 cell line with leukemic FIA18 cell line

*In vitro* cultures of FIA10 cells and FIA18 cells grown in self-renewing conditions consist of a mixture of suspension and adherent cells with a high percentage of immature blasts and some differentiated myeloid forms including monocytic cells, macrophages and granulocytes (Table 2). In the presence of differentiation-inducing factors, such as FCS and hematopoietic cytokines, FIA10 cells as well as FIA18 cells differentiate further into mature granulocytes, monocytes, macrophages and erythroid cells (Table 2) in line with other factor-independent GMV-FDCP-mix derived mutants [5]. In soft agar without additional cytokines, mutant cell clone FIA10 form undifferentiated and multicenter colonies (S1 Fig), further indicating an immature phenotype with myeloid differentiation. Taken together these results demonstrate that FIA10 and FIA18 cells have very similar myeloid differentiation potential and cloning capacity *in vitro*, despite the quite distinct disease pattern induced after injection into mice.

**Table 2:** Characterisation of FIA10 and FA18 cells *in vitro*.

Cell line	Cell Culture Conditions	Doubling Time	Morphology % Cells in Suspension						Adherent Cells
			Bl	Gr	Mono	Eo	Mast	Ebl	
FIA10	20% HS, IL-3 100 U/ml	24–48 hrs	91	4	1	1	0	0	+
FIA18	20% HS, IL-3 100 U/ml	24–48 hrs	96	4	0	0	0	0	+
FDCP-Mix	20% HS, IL-3 100 U/ml	24 hrs	99	0	1	0	0	0	-
FIA10	20% HS, no cytokines	48 hrs	62	31	0	7	0	0	++
FIA18	20% HS, no cytokines	48 hrs	60	34	2	4	0	0	++
FIA10	d7, 20% FCS,	n.d.	22	15	60	0	0	2	+++
	5 U/ml IL-3, 2 U/ml Epo								
FIA18	d7, 20% FCS,	n.d.	18	19	58	1	1	3	+++
	5 U/ml IL-3, 2 U/ml Epo								
FDCP-Mix	d7, 20% FCS,	n.d.	8	12	58	2	15	2	+++
	5 U/ml IL-3, 2 U/ml Epo								

Abbreviations: n.d, not determined; HS, horse serum; FCS, fetal calf serum; Epo, erythropoietin; IL-3, interleukin-3; Gr, granulocytes; Mo, monocytes and macrophages; Eo, eosinophils; Mast, mast cells; Ebl, erythroblasts. Cells were cultured for 7 days in IMDM, HS or FCS and cytokines as indicated in the presence of 5% CO_2_ and 5% O_2_. Self-renewing conditions: 20% HS with 100 U/ml IL-3; Multipotent differentiation conditions: 20% FCS, 5 U/ml IL-3, 2 U/ml Epo; FDCP-Mix cells died in the absence of growth factors within 24 hrs. Three replicates per experiment.

### CNS infiltrating FIA10 cells display altered RNA and protein expression compared with leukemic clone FIA18 that does not metastasize into the brain and the parental hematopoietic progenitor FDCP-Mix line

**Fig 2:**
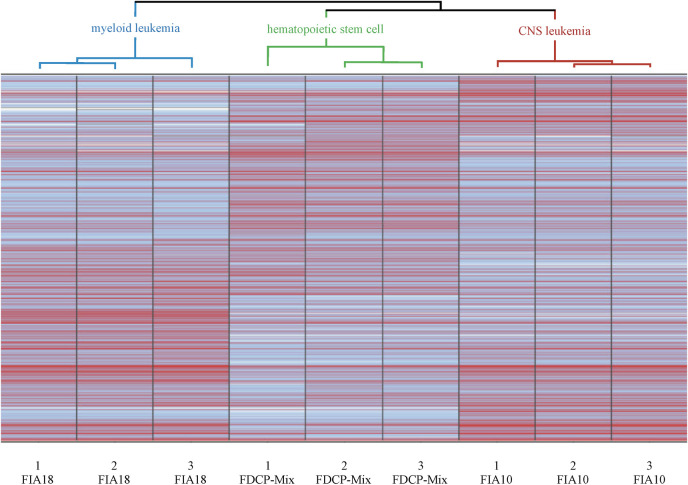
Hierarchical cluster analysis between FIA10, FIA18 and FDCP-Mix cells. Abundance (log2 values) from low (white) to intermediate (blue) to high (red) expression from all probe sets. Analyses were done in triplicates. Values within the triplicates are similar with minor variances. mRNA expressions patterns of FIA10 compared with FIA18 and FDCP-Mix are considerably different with FIA10 being more close to FDCP-Mix but also sharing expression levels for a number of mRNAs with FIA18.

**Fig 3:**
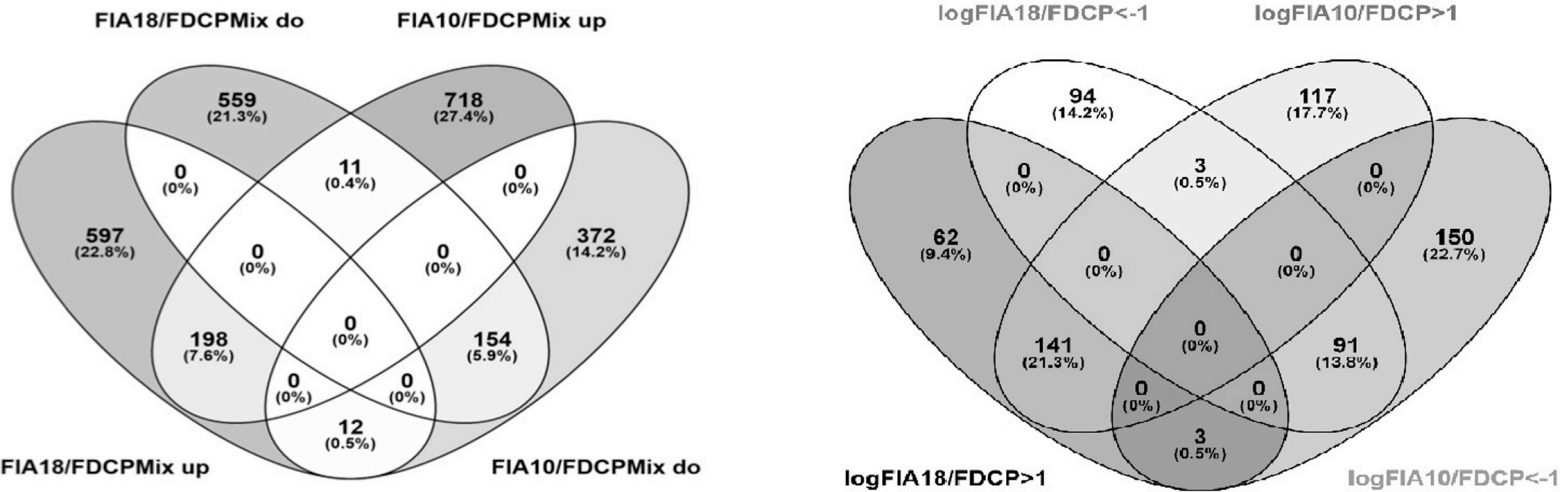
Venn diagrams of differential RNA and protein expression in FIA10 and FIA18 in relation to FDCP-Mix cells. RNAs and proteins were selected as up- or down-regulated if they had a log2fold change ≥ 1.0 or ≤ 1.0 with a p value ≤ 0.05. (A) Venn diagram of differential RNA expression. (B) Venn diagram of differential protein expression.

In search for potential mechanism(s) that selectively mediate leukemic metastasis into the brain, we compared genome-wide RNA and protein expression of FIA10 cells, which are capable of breaking down the BBB and specifically infiltrate the brain parenchyma, with FIA18 cells, which give rise to leukemia but do not metastasize to the brain, as well as with the parental FDCP-Mix hematopoietic progenitor cell line [5, 8]. As shown in Fig 2, RNA expression of FIA10 cells had overlapping as well as opposing expression levels with the leukemic FIA18 as well as with parental FDCP-Mix hematopoietic progenitor cell lines. Concerning differences in RNA expression of FIA10 compared with FIA18 cells, 188 genes were overexpressed and 116 underexpressed (S2 and S3 Tables). 10 genes upregulated and 10 genes downregulated in FIA10 cells compared with FIA18 cells in the gene expression arrays were chosen for independent validation by quantitative real time PCR with reverse transcription (RT-qPCR, S4 Table). At the protein level, 189 genes were upregulated (log2 ratio FIA10/FIA18 ≥ 1) and 177 genes were downregulated (log2 ratio FIA10/FIA18 ≤ 1) in FIA10 cells compared with FIA18 cells (S5 Table). Comparing differentially expressed genes of FIA10 cells with FIA18 and FDCP-Mix cell lines at the RNA and protein level, most genes are either up- or downregulated in FIA10 at the RNA and protein level, which is shown at the Venn diagrams for differentially expresssed RNAs or proteins (Fig 3A and 3B).

### Ingenuity Pathway Analysis of differentially expressed RNA and protein of the brain leukemic FIA10 cell line compared with the parental hematopoietic FDCP-Mix and myeloid leukemic FIA18 cell lines

Biofunctional analyses were performed on the sets of differentially expressed RNAs and proteins of FIA10 cells vs controls (S2 and S3 Tables). To interpret the biological significance of expression data, QIAGEN Ingenuity Pathway Analysis (QIAGEN IPA) was performed on differentially expressed RNA and protein of FIA10 cells compared with FIA18 and FDCP-Mix cells, respectively. In line with the observed brain metastasis, top alterations in RNA and protein expression between FIA10 cells and control cells were found in processes involved in the inflammatory response, inflammatory disease, organismal injury and abnormalities, cellular movement, cell-to-cell signaling, cell death and survival, immune cell trafficking, hematopoietic system development and function and tissue morphology (Table 3). In all but two categories considerable correlation between the mRNA and protein results were found, corroborating the significance of these results. Some discordance is expected as the spatial and temporal variations of protein and mRNAs influence the relationship between protein levels and their coding transcripts [26, 27].

**Table 3:** Top diseases and biological functions based on differentially expressed RNA and protein of brain metastasing FIA10 cells.

Category	Protein expression of brain metastatic FIA10 cells vs. leukemic non-brain metastatic FIA18 cells		RNA expression of brain metastatic FIA10 cells vs. hematopoietic stem cell lines FDCP-Mix	
	**p-value**	**#Molecules**	**p-value**	**#Molecules**
**Diseases and Disorders**				
Inflammatory Response	2,65E-03–1,02E-10	76	1,86E-05–1,46E-24	69
Organismal Injury and Abnormalities	2,65E-03–1,39E-09	161	1,87E-05–6,57E-19	42
Inflammatory Disease	2,65E-03–4,97E-08	53	1,49E-05–6,57E-19	59
Connective Tissue Disorders			1,49E-05–6,57E-19	42
Neurological Disease	2,48E-03–4,97E-08	48		
**Molecular and Cellular Functions**				
Cellular Movement	2,68E-03–3,69E-12	62	1,86E-05–1,44E-28	63
Cell-To-Cell Signaling and Interaction	2,63E-03–8,54E-10	59	1,56E-05–6,22E-18	56
Cell Death and Survival	2,65E-03–3,29E-11	83	1,43E-05–3,12E-14	61
Cellular Function and Maintenance	2,07E-03–7,13E-09	60		
Cell Morphology	2,61E-03–7,65E-08	32		
Cellular Development			1,76E-05–3,38E-15	48
Cellular Growth and Proliferation			1,76E-05–3,38E-15	49
**Physiological System: Development and Function**				
Immune Cell Trafficking	2,60E-03–3,15E-11	43	1,48E-05–1,44E-28	54
Hematological System Development and Function	2,68E-03–8,54E-10	59	1,56E-05–1,63E-26	64
Tissue Morphology	2,42E-03–5,63E-08	44	8,23E-06–4,22E-19	51
Lymphoid Tissue Structure and Development	2,29E-03–2,42E-06	41	1,47E-05–3,38E-15	32

QIAGEN Ingenuity Pathway Analysis (IPA) was performed on differentially expressed RNA and protein of FIA10 compared with FIA18 and the parental FDCP-Mix cells to interpret biological significance of expression data with the QIAGEN Knowledge Base as a reference data set. Right-tailed Fisher’s Exact Test p-values indicate the significance of relationships between the analysed data sets and the functional frameworks generated by IPA.

### GO enrichment analysis of differentially expressed RNA and protein of the brain leukemic FIA10 cell line compared with the parental hematopoietic FDCP-Mix and myeloid leukemic FIA18 cell lines

Next, we analysed functional groups that were significantly differentially represented in FIA10 cells with a GO enrichment analysis. Tables 4, 5 shows enrichment in specific cellular component, molecular function and biological processes of selected upregulated (Table 4) and down-regulated (Table 5) protein and RNA expression of FIA10 cells compared with FIA18 cells. All differentially expressed genes are categorized in S2–S5 Figs and S6–S17 Tables. In the cellular component domain, the categories extracellular region (RNA and protein up- and down-regulated) and MHC protein complex (RNA and protein up-regulated), lytic vacuoles (RNA and protein upregulated) and nucleosomes (protein upregulated) were prevalent in FIA10. In the molecular function domain, the most significantly regulated categories included cytokine receptor activity (RNA up-regulated and protein down-regulated). In addition, down-regulation of cell-adhesion molecule binding, lipoprotein particle binding, lipopolysaccharide binding and integrin binding (all RNA) as well as upregulation of MHC protein complex binding (RNA and protein), monocarboxylic acid binding (protein), antigen binding (RNA and protein) and chemokine activity (RNA), cytokine binding and signaling receptor activity (RNA), and single-stranded DNA 5’-3’ exodeoxyribonuclease activity (RNA) were all significantly differentially regulated.

**Table 4:** Gene ontology enrichment analysis based on of differentially expressed RNA and protein of brain metastatic FIA10 cells: Upregulated protein and RNA expression of brain metastatic FIA10 cells compared with leukemic but non-brain metastatic FIA18 cells.

Category	Upregulated protein expression of FIA10 vs. FIA18 cells	Upregulated RNA expression of FIA10 vs. FIA18 cells
**Cellular component**	MHC protein complex (9.5E-15)	MHC protein complex (2.1E-13)
	Extracellular region (2.1E-10)	External side of plasma membrane (2.4E-11)
	Plasma membrane protein complex (2.9E-7)	Extracellular region part (1.6E-10)
	Vacuolar part (6.9E-7)	Extracellular space (3.5E-10)
	Lytic vacuole (4.4E-6)	Lytic vacuole (2E-8)
	Nucleosome (8.4E-5)	Lysosome (2E-8)
	Extracellular space (1.4E-5)	Vacuole (1.6E-7)
	Extracellular matrix (1.2E-4)	Plasma membrane part (7.9E-7)
		Cell surface (7.3E-6)
		Membrane part (1.4E-5)
		Extracellular region (2.3E-5)
		Extracellular matrix (2.1E-4)
		CSF1-CSF1R complex (1.5E-4)
**Molecular function**	MHC protein complex binding (5.0E-6)	Cytokine binding (1.2E-7)
	MHC class II protein complex binding (1.0E-6)	Signaling receptor binding (1.1E-7)
	Antigen binding (4.9E-5)	Chemokine receptor binding (3.6E-6)
	Peptide antigen binding (1.3E-5)	Chemokine activity (2.9E-6)
	Monocarboxylic acid binding (6.8E-4)	Antigen binding (1.6E-5)
		Signaling receptor activity (2.3E-5)
		MHC class II protein complex binding (3.8E-5)
		Cytokine receptor activity (4.6E-5)
		Molecular transducer activity (4.7E-5)
		Receptor ligand activity (1.9E-4)
		MHC protein complex binding (2.3E-4)
		Amide binding (3.1E-4)
		CCR1 chemokine receptor binding (3.2E-4)
		Receptor regulator activity (4.3E-4)
		Single stranded DNA 5’-3’ exodesoxyribonuclease activity (6.4E-4)
		Scavenger receptor binding (6.4E-4)
**Biological process**	Immune system process (3.0E-8)	Immune system process (5.2E-20)
	Regulation of immune system process (2.5E-4)	Regulation of immune system process (9.0E-18)
	Immune response (7.4E-9)	Immune response (1.2E-17)
	Regulation of multicellular organismal process (6.8E-5)	Response to cytokine (9.7E-14)
	Antigen processing & presentation (1.4E-10)	Regulation of multicellular organismal process (9.0E-13)
	Defense response (1.8E-5)	Antigen processing and presentation (4.2E-13)
	Chromatin silencing (3.5E-6)	Defense response (6.9E-12)
	Negative regulation of gene expression, epigenetic (1.4E-5)	Regulation of inflammatory response (3.3E-11)
	Regulation of antigen processing and presentation (6.5E-5)	Inflammatory response (2.8E-11)
	Plasma membrane repair (3.1E-4)	Positive regulation of cell-cell adhesion (1.4E-9)
	Iron ion transport (5.9E-4)	Regulation of cell migration (8.2E-8)
		Negative regulation of cell adhesion (4.2E-8)
		Regulation of cell motility (6.8E-8)
		Collagen catabolic process (4.6E-7)
		Cell chemotaxis (5.8E-7)
		Positive regulation of cell migration (1.4E-6)
		Negative regulation of cell death (3.0E-6)
		Phagocytosis (3.1E-6)
		Regulation of neuroinflammatory response (5.2E-6)
		Cell motility (1.3E-5)
		Collagen metabolic process (1.6E-5)
		Positive regulation of microglial migration (2.2E-5)
		Regulation of cell shape (2.2 E-5)
		Negative regulation of immune response (3.4E-5)
		Positive regulation of glial cell migration (2.9E-4)
		Long–chain fatty acid metabolic process (4.7E-4)
		Regulation of neurological system process (5.4E-4)
		Response to amyloid-beta (7.7E-4)
		Cellular extravasation (8.1E-4)

Differentially expressed RNA and protein were ranked according to their p-values of differential expression and degree of enrichment compared with the total number of expressed genes analysed (17680 GO terms for RNA and 6008 GO terms for protein). The GOrilla database updated on Mar 6, 2021 was used. Within parenthesis is the enrichment p-value computed according to the mHG or HG model.

**Table 5:** Gene ontology enrichment analysis based on of differentially expressed RNA and protein of brain metastatic FIA10 cells: Downregulated protein and RNA expression of brain metastatic FIA10 cells compared with leukemic but non-brain metastatic FIA18 cells.

Category	Downregulated protein expression of FIA10 vs. FIA18 cells	Downregulated RNA expression of FIA10 vs. FIA18 cells
**Cellular component**		
	Extracellular region part (9.2E-5)	Extracellular region part (3.2E-4)
		Cell surface (6.9E-4)
		Extracellular space (5.4E-4)
**Molecular function**	Cytokine receptor activity (7.7E-4)	Cell-adhesion molecule binding (7.6E-5)
		Lipoprotein particle binding (5.8E-5)
		Protein-lipid complex binding (5.8E-5)
		Lipopolysaccharide binding (9.0E-4)
		Integrin-binding (4.0E-4)
		Protein-containing complex binding (1.4E-4)
**Biological process**	Leukocyte migration involved in inflammatory response (2.9E-4)	Inflammatory response (5.8E-6)
		Lipopolycaccharide transport (5.4E-5)
		Phagocytosis, recognition (2.7E-5)
		Defense response (2.5E-5)
		Acute inflammatory response (9.4E-4)
		Response to biotic stimulus (8.4E-4)
		Regulation of cell migration (8.3E-4)
		Immunoglobulin mediated immune response (8.0E-4)
		Regulation of immune system process (7.9E-4)
		Negative regulation of lipid metabolic process (7.5E-4)
		Response to external biotic stimulus (6.2E-4)
		Regulation of protein exit from endoplasmic reticulum (6.2E-4)
		Leukocyte migration involved in inflammatory response (5.4E-4)
		Regulation of phospholipid catabolic process (5.3E-4)
		Recognition of apoptotic cell (5.3E-4)
		Regulation of triglyceride metabolic process (4.6E-4)
		Positive regulation of immune system process (3.7E-4)
		Regulation of toll-like receptor 4 signaling pathway (3.5E-4)
		Pyrimidine dimer repair by nucleotide-excision repair (3.2E-4)
		Innate immune response (2.8E-4)
		Regulation of phospholipid metabolic process (2.0E-4)

Differentially expressed RNA and protein were ranked according to their p-values of differential expression and degree of enrichment compared with the total number of expressed genes analysed (15727 GO terms for RNA and 6003 GO terms for protein). The GOrilla database updated on Mar 6, 2021 was used. Within parenthesis is the enrichment p-value computed according to the mHG or HG model.

In the biological process domain, the prevalent categories for upregulated RNA as well as protein of FIA10 compared with FIA18 cells were immune system process and its regulation, regulation of multicellular organismal process, antigen processing and presentation and defense response (Table 4). For upregulated protein of FIA10 vs FIA18 cells, chromatin silencing and negative epigenetic regulation of gene expression were listed, while upregulated FIA10 vs FIA18 RNA further included response to cytokine, inflammatory response and its regulation, positive regulation of cell-cell adhesion, (positive) regulation of cell migration, cell motility and its regulation, collagen catabolic and metabolic process, cell chemotaxis, negative regulation of cell death, regulation of neuroinflammatory response, positive regulation of microgial and glial migration, long-chain fatty acid metabolic process and cellular extravasation (Table 4, S2 and S3 Figs and S6–S11 Tables). For down-regulated FIA10 protein and RNA, the prevalent category in the biological process domain was leukocyte migration involved in the inflammatory response (Table 5). In addition, downregulated FIA10 vs FIA18 RNA in the biological process domain extended to further immune response categories including the inflammatory response, defence response, innate immune response, regulation of immune system process and acute inflammatory response. Other categories for down-regulated FIA10 vs FIA18 RNA encompassed regulation of cell migration, pyrimidine dimer repair by nucleotide-excision repair and regulation of triglyceride metabolic process (Table 5, S4 and S5 Figs and S12–S17 Tables).

## Discussion

In this study we have described a novel myeloid leukemic cell line, FIA10, that specifically metastasizes into the brain without major involvement of hematopoietic organs such as bone marrow, spleen, and peripheral blood. Comparison of differential RNA and protein expression of FIA10 with another myeloid leukemic cell line, FIA18, and the parental hematopoietic FDCP-Mix stem cell line, points to biological processes and molecular functions that may be responsible for the newly acquired phenotype. As discussed below, several of the deregulated RNAs and proteins may be involved in these processes.

In IPA as well as GO analyses, immune system processes were the top pathways of differentially expressed proteins and RNAs in FIA10 cells compared to FIA18 cells, suggesting a critical involvement of the immune system in our model system of brain metastasis. In the cellular component and molecular function categories, the major histocompatibility complex (MHC) was prevalent for upregulated protein and RNA expression of FIA10 vs. FIA18. Down-regulation of MHC expression by leukemia cells is a critical mechanism of immune escape and immune-resistant phenotypes of malignant cells [28, 29]. Compared with the hematopoietic stem cell line FDCP-Mix, leukemic FIA18 cells have downregulated expression of MHC class I and, even more pronounced, MHC class II molecules. This suggests that the tumorigenic potential of FIA18 is augmented by escaping tumor-surveillance by CD4 T-cells and class I-restricted CD8^+^ cytotoxic T cells. In contrast, similar to glioblastoma multiforme [30], brain metastasizing FIA10 cells express similar levels of MHC class I and II antigens as the parental FDCP-Mix cells and much higher levels compared with leukemic clone FIA18. Interestingly, FIA10 cells were found in the CNS but not in peripheral blood or bone marrow at onset of CNS disease. We thus speculate that FIA10 cells migrate into the brain, an immune-privileged site shielded by the BBB, which may help FIA10 cells to escape immune recognition.

Despite the BBB, the brain holds a specific immune activity that involves antigen-presenting cells such as dendritic cells, macrophages and microglia, as well as T-cells [31]. An important mechanism allowing tumor cells to escape immune recognition are tumor-associated anti-inflammatory innate immune cells (Tumor-Associated Macrophages, TAMs). Upon settling in the parenchyma, metastatic tumor cells release cytokines, such as GM-CSF, to recruit TAMs to the tumor microenvironment [31]. Tumor-derived GM-CSF further supports survival and pro-tumorigenic polarisation of brain-resident microglia and macrophages [32]. FIA10 as well as FIA18 cells express GM-CSF/CSF2 [8], which may lead to activation of TAMs and may particularly help FIA10 cells to evade the immune system in brain. In addition, FIA10 show higher RNA and protein expression of MGL2, a C-type lectin receptor with immune suppressive properties [33], which is regulated by GM-CSF. Further, compared with FIA18 and FDCP-Mix cells, FIA10 cells show highly up-regulated RNA expression of CSF1/M-CSF and several chemokine (CCL) ligands, i.e. Ccl2, Ccl3, Ccl4, Ccl5 and Ccl17, that cause an immunosuppressive M2 phenotype of TAMs [34]. Taken together, these results suggest that FIA10 cells may polarize TAMs to an anti-inflammatory, immunosuppressive phenotype, thereby assisting FIA10 cells in evading immunity in brain.

To metastasize into the brain, FIA10 cells have to cross the BBB. In GO analysis, the cellular component categories ’extracellular region’ and ’extracellular space’ showed an enrichment of 4-fold for upregulated protein and 3-fold for RNA, respectively, and include several proteases implicated in BBB opening. Of potential importance, FIA10 highly express matrix metalloproteases (MMPs) 2, 8, 9, 12, and 13, which facilitate crossing the BBB [35]. Expression of MMP2 and MMP9 is regulated by fatty acid-binding protein 5 (FABP5), a cancer metastasis promoting protein [36, 37]. FABP5 was highly upregulated in FIA10 cells compared with FIA18 cells and thus may, besides its other roles for tumor growth (see below), induce MMP expression in FIA10 cells. Further, Cathepsin S, which mediates BBB transmigration through proteolytic processing of the junctional adhesion molecule JAM-B in breast to brain metastasis [38], was highly up-regulated in FIA10 cells along with Cathepsin B and O. In addition, upregulated expression of Capase 1 in FIA10 cells may support BBB transmigration [39].

After opening the BBB, the invading cells need to dock and adhere to the local environment as well as to remodel the surrounding extracellular matrix for invasion and migration into the brain parenchyma. In IPA analysis, the categories ’Connective Tissue Disorders’, ’Cell-To-Cell Signaling and Interaction’ and ’Tissue Morphology’ were among the top diseases and biological functions for differential RNA and protein expression, implicating a high importance of these processes in brain metastasis of FIA10 cells. In GO analysis, the cellular component function categories ’extracellular region’, ’extracellular matrix’ and ’plasma membrane protein complex’ had a high degree of differential expression and enrichment for upregulated protein and RNA of several receptors, adhesion molecules and enzymes with potential involvement in modulation of cell-matrix interactions. Which of the numerous differentially expressed molecules with a potential function for these processes, such as the extracellular matrix crosslinking enzyme transglutaminase 2 [40], Rho GTPases (Cdc42-GTP) involving formins, e.g. Formin-like 1 and Diaphanous-related Diap2 (mDia3) [41] or matrix metalloproteases as downstream effectors, remains to be tested *in vitro* models of brain metastasis.

In the brain microenvironment, consumption of oxygen may be rate limiting for tumor proliferation, requiring increased glycolysis and decreased mitochondrial activity [42]. Similar to the metabolomes of brain tumors [43], FIA10 cells likely rely on glycolysis for energy production rather than mitochondrial activity or oxidative phosphorylation, reminiscent of the Warburg effect [44], as they have highly up-regulated brain specific aldolase C (ALDOC) [45] RNA and protein expression. The aldolase family enzymes predominantly function in glycolysis and ALDOC expression is mainly found in the cerebellum [46], a major site to which FIA10 cells metastasized. Along this line, microglia cells were shown to upregulate the expression of ALDOC in brain-metastasizing melanoma cells, thereby facilitating brain metastasis formation [47, 48]. Further, GO analysis revealed several upregulated proteins of FIA10 vs. FIA18 in the molecular function category ’monocarboxylic acid binding’. Among those, the glycogen phosphorylase PYGL, which supports glycogenolysis, was highly up-regulated at the protein and RNA level, underlining a potential dependence and use of glucose and glycogen in brain by FIA10 cells.

Fatty acids and their metabolism are important in tumor development, as lipids also serve as energy sources and are required for membrane biosynthesis. In the GO analysis ’biological function’ category the ’long-chain fatty acid metabolic process’ was enriched for FIA10 vs. FIA18 upregulated RNA expression, and ’negative regulation of lipid metabolic process’ and ’regulation of phospholipid and triglyceride metabolic processes’ for FIA10 vs. FIA18 downregulated RNA expression, implicating a potential important role for lipid metabolism alterations in FIA10 metastasis in brain. In line with other studies showing a correlation of high FABP5 expression with poor prognosis and metastasis in different tumor types [49], the brain-typed FABP5, which participates in long-chain fatty acid uptake, transport and metabolism was highly upregulated at the RNA and protein level in FIA10. Further, the mitochondrial Acyl/Malonyl-CoA synthetases ACSF2 and ACSF3, which activate fatty acids for metabolism and phospholipid synthesis and remodeling, were both up-regulated in FA10. In glioblastoma and other tumor cells, fatty acids are stored in lipid droplets to maintain energy homeostasis allowing growth via autophagic release of the stored fatty acids [50]. In GO analysis, the ’lytic vacuole’ cellular component was highly enriched for FIA10 vs. FIA18 cells at the protein and RNA level. It is tempting to speculate, that FIA10 may use a similar mechanism for energy production upon glucose reduction.

Taken together our proteomic and transcriptomic comparison of the brain metastasizing FIA10 cell line with the leukemic FIA18 and parental FDCP-Mix cell lines points out several alterations, in particular evasion of the immune response, transmigration of the BBB, invasion of the brain parenchyma and adaptation to the brain microenvironment including metabolic reprogramming, that may be critically involved in brain metastasis.

## Supporting information

S1 Fig FIA10 cells form undifferentiated multicenter and compact colonies in soft agar. (A) Multicenter colony of FIA10 cells. (B) Compact colony of FIA10 cells.
(TIFF)

S2 Fig GO enrichment analysis biological process for differentially expressed RNA and protein in FIA10 cells: FIA10 RNA up-regulated. Differentially expressed RNAs were ranked according to their p-values of differential expression and degree of enrichment compared with the total number of expressed genes analysed.
(PDF)

S3 Fig GO enrichment analysis biological process for differentially expressed RNA and protein in FIA10 cells: FIA10 protein up-regulated. Differentially expressed proteins were ranked according to their p-values of differential expression and degree of enrichment compared with the total number of expressed genes analysed.
(PDF)

S4 Fig GO enrichment analysis biological process for differentially expressed RNA and protein in FIA10 cells: FIA10 RNA down-regulated. Differentially expressed RNAs were ranked according to their p-values of differential expression and degree of enrichment compared with the total number of expressed genes analysed.
(PDF)

S5 Fig GO enrichment analysis biological process for differentially expressed RNA and protein in FIA10 cells: FIA10 protein down-regulated. Differentially expressed proteins were ranked according to their p-values of differential expression and degree of enrichment compared with the total number of expressed genes analysed.
(PDF)

S1 Table Primer sequences for RT-qPCR. (DOCX)

S2 Table Differential underexpressed RNA FIA10 vs FIA18. (XLSX)

S3 Table Differential overexpressed RNA FIA10 vs FIA18. (XLSX)

S4 Table Differentially expressed proteins FIA10 vs FIA18. (XLSX)

S5 Table qPCR validation of FIA10 and FIA18 mRNA expression. (DOCX)

S6 Table GO enrichment analysis of FIA10 RNA vs FIA18 upregulated ‐ Biological process. (DOCX)

S7 Table GO enrichment analysis of FIA10 RNA vs FIA18 upregulated ‐ Molecular function. (DOCX)

S8 Table GO enrichment analysis of FIA10 RNA vs FIA18 upregulated ‐ Cellular component. (DOCX)

S9 Table GO enrichment analysis of FIA10 Protein vs FIA18 upregulated ‐ Biological process. (DOCX)

S10 Table GO enrichment analysis of FIA10 Protein vs FIA18 upregulated ‐ Molecular function. (DOCX)

S11 Table GO enrichment analysis of FIA10 Protein vs FIA18 upregulated ‐ Cellular component. (DOCX)

S12 Table GO enrichment analysis of FIA10 RNA vs FIA18 downregulated ‐ Biological process. (DOCX)

S13 Table GO enrichment analysis of FIA10 RNA vs FIA18 downregulated ‐ Molecular function. (DOCX)

S14 Table GO enrichment analysis of FIA10 RNA vs FIA18 downregulated ‐ Cellular component. (DOCX)

S15 Table GO enrichment analysis of FIA10 Protein vs FIA18 downregulated ‐ Biological process. (DOCX)

S16 Table GO enrichment analysis of FIA10 Protein vs FIA18 downregulated ‐ Molecular function. (DOCX)

S17 Table GO enrichment analysis of FIA10 Protein vs FIA18 downregulated ‐ Cellular component. (DOCX)

## References

[1] Creutzig U, Dworzak MN, Zimmermann M, Reinhardt D, Sramkova L, Bourquin JP, et al. Characteristics and outcome in patients with central nervous system involvement treated in European pediatric acute myeloid leukemia study groups. Pediatr Blood Cancer. 2017;64(12). Epub 2017/06/10. doi: 10.1002/pbc.26664 . 28598536

[2] Alakel N, Stolzel F, Mohr B, Kramer M, Oelschlagel U, Rollig C, et al. Symptomatic central nervous system involvement in adult patients with acute myeloid leukemia. Cancer Manag Res. 2017;9:97–102. Epub 2017/04/25. doi: 10.2147/CMAR.S125259 ; PubMed Central PMCID: PMC5386598. 28435324 PMC5386598

[3] Liebner S, Dijkhuizen RM, Reiss Y, Plate KH, Agalliu D, Constantin G. Functional morphology of the blood-brain barrier in health and disease. Acta Neuropathol. 2018;135(3):311–36. doi: 10.1007/s00401-018-1815-1 . 29411111 PMC6781630

[4] Quail DF, Joyce JA. The Microenvironmental Landscape of Brain Tumors. Cancer Cell. 2017;31(3):326–41. Epub 2017/03/16. doi: 10.1016/j.ccell.2017.02.009 ; PubMed Central PMCID: PMC5424263. 28292436 PMC5424263

[5] Just U, Spooncer E, Lohler J, Stocking C, Ostertag W, Dexter TM. Mutants of a multipotent hematopoietic cell line blocked in GM-CSF-induced differentiation are leukemogenic in vivo. Exp Hematol. 1994;22(9):933–40. Epub 1994/08/01. . 8062891

[6] Spooncer E, Heyworth CM, Dunn A, Dexter TM. Self-renewal and differentiation of interleukin-3-dependent multipotent stem cells are modulated by stromal cells and serum factors. Differentiation. 1986;31(2):111–8. Epub 1986/01/01. doi: 10.1111/j.1432-0436.1986.tb00391.x . 3091439

[7] Karasuyama H. [Establishment of mouse cell lines which constitutively secrete large quantities of interleukin 2, 3, 4 or 5, using high-copy cDNA expression vectors]. Tanpakushitsu Kakusan Koso. 1988;33(14):2527–32. Epub 1988/11/01. . 3266805

[8] Just U, Stocking C, Spooncer E, Dexter TM, Ostertag W. Expression of the GM-CSF gene after retroviral transfer in hematopoietic stem cell lines induces synchronous granulocyte-macrophage differentiation. Cell. 1991;64(6):1163–73. Epub 1991/03/22. doi: 10.1016/0092-8674(91)90271-y . 2004422

[9] Boettiger D, Anderson S, Dexter TM. Effect of src infection on long-term marrow cultures: increased self-renewal of hemopoietic progenitor cells without leukemia. Cell. 1984;36(3):763–73. Epub 1984/03/01. doi: 10.1016/0092-8674(84)90356-8 . 6321038

[10] Wu Q, Chen M, Buchwald M, Phillips RA. A simple, rapid method for isolation of high quality genomic DNA from animal tissues. Nucleic Acids Res. 1995;23(24):5087–8. Epub 1995/12/25. doi: 10.1093/nar/23.24.5087 ; PubMed Central PMCID: PMC307519. 8559671 PMC307519

[11] Bishop CE, Boursot P, Baron B, Bonhomme F, Hatat D. Most classical Mus musculus domesticus laboratory mouse strains carry a Mus musculus musculus Y chromosome. Nature. 1985;315(6014):70–2. doi: 10.1038/315070a0 . 2986012

[12] Homma R, Yoshikawa H, Takeno M, Kurokawa MS, Masuda C, Takada E, et al. Induction of epithelial progenitors in vitro from mouse embryonic stem cells and application for reconstruction of damaged cornea in mice. Invest Ophthalmol Vis Sci. 2004;45(12):4320–6. Epub 2004/11/24. doi: 10.1167/iovs.04-0044 . 15557438

[13] Saeed AI, Sharov V, White J, Li J, Liang W, Bhagabati N, et al. TM4: a free, open-source system for microarray data management and analysis. Biotechniques. 2003;34(2):374–8. Epub 2003/03/05. doi: 10.2144/03342mt01 . 12613259

[14] Ashburner M, Ball CA, Blake JA, Botstein D, Butler H, Cherry JM, et al. Gene ontology: tool for the unification of biology. The Gene Ontology Consortium. Nat Genet. 2000;25(1):25–9. doi: 10.1038/75556 ; PubMed Central PMCID: PMC3037419. 10802651 PMC3037419

[15] Thomas PD, Campbell MJ, Kejariwal A, Mi H, Karlak B, Daverman R, et al. PANTHER: a library of protein families and subfamilies indexed by function. Genome Res. 2003;13(9):2129–41. doi: 10.1101/gr.772403 ; PubMed Central PMCID: PMC403709. 12952881 PMC403709

[16] Schroeder T, Meier-Stiegen F, Schwanbeck R, Eilken H, Nishikawa S, Hasler R, et al. Activated Notch1 alters differentiation of embryonic stem cells into mesodermal cell lineages at multiple stages of development. Mech Dev. 2006;123(7):570–9. doi: 10.1016/j.mod.2006.05.002 . 16822655

[17] Kruger M, Moser M, Ussar S, Thievessen I, Luber CA, Forner F, et al. SILAC mouse for quantitative proteomics uncovers kindlin-3 as an essential factor for red blood cell function. Cell. 2008;134(2):353–64. doi: 10.1016/j.cell.2008.05.033 . 18662549

[18] Shevchenko A, Tomas H, Havlis J, Olsen JV, Mann M. In-gel digestion for mass spectrometric characterization of proteins and proteomes. Nat Protoc. 2006;1(6):2856–60. doi: 10.1038/nprot.2006.468 . 17406544

[19] Rappsilber J, Ishihama Y, Mann M. Stop and go extraction tips for matrix-assisted laser desorption/ionization, nanoelectrospray, and LC/MS sample pretreatment in proteomics. Anal Chem. 2003;75(3):663–70. doi: 10.1021/ac026117i . 12585499

[20] Cox J, Mann M. MaxQuant enables high peptide identification rates, individualized p.p.b.-range mass accuracies and proteome-wide protein quantification. Nat Biotechnol. 2008;26(12):1367–72. doi: 10.1038/nbt.1511 . 19029910

[21] Cox J, Neuhauser N, Michalski A, Scheltema RA, Olsen JV, Mann M. Andromeda: a peptide search engine integrated into the MaxQuant environment. J Proteome Res. 2011;10(4):1794–805. doi: 10.1021/pr101065j . 21254760

[22] Oliveros JC. An interactive tool for comparing lists with Venn’s diagrams. 2007–2015.

[23] Eden E, Navon R, Steinfeld I, Lipson D, Yakhini Z. GOrilla: a tool for discovery and visualization of enriched GO terms in ranked gene lists. BMC Bioinformatics. 2009;10:48. Epub 2009/02/05. doi: 10.1186/1471-2105-10-48 ; PubMed Central PMCID: PMC2644678. 19192299 PMC2644678

[24] Eden E, Lipson D, Yogev S, Yakhini Z. Discovering motifs in ranked lists of DNA sequences. PLoS Comput Biol. 2007;3(3):e39. Epub 2007/03/27. doi: 10.1371/journal.pcbi.0030039 ; PubMed Central PMCID: PMC1829477. 17381235 PMC1829477

[25] Kramer A, Green J, Pollard J Jr., Tugendreich S. Causal analysis approaches in Ingenuity Pathway Analysis. Bioinformatics. 2014;30(4):523–30. Epub 2013/12/18. doi: 10.1093/bioinformatics/btt703 ; PubMed Central PMCID: PMC3928520. 24336805 PMC3928520

[26] Liu Y, Beyer A, Aebersold R. On the Dependency of Cellular Protein Levels on mRNA Abundance. Cell. 2016;165(3):535–50. Epub 2016/04/23. doi: 10.1016/j.cell.2016.03.014 . 27104977

[27] Buccitelli C, Selbach M. mRNAs, proteins and the emerging principles of gene expression control. Nat Rev Genet. 2020;21(10):630–44. Epub 2020/07/28. doi: 10.1038/s41576-020-0258-4 . 32709985

[28] Pagliuca S, Gurnari C, Hercus C, Hergalant S, Hong S, Dhuyser A, et al. Leukemia relapse via genetic immune escape after allogeneic hematopoietic cell transplantation. Nat Commun. 2023;14(1):3153. Epub 2023/06/01. doi: 10.1038/s41467-023-38113-4 ; PubMed Central PMCID: PMC10232425. 37258544 PMC10232425

[29] Tarafdar A, Hopcroft LE, Gallipoli P, Pellicano F, Cassels J, Hair A, et al. CML cells actively evade host immune surveillance through cytokine-mediated downregulation of MHC-II expression. Blood. 2017;129(2):199–208. Epub 2016/10/30. doi: 10.1182/blood-2016-09-742049 ; PubMed Central PMCID: PMC5305055. 27793879 PMC5305055

[30] Wang L, Wei B, Hu G, Wang L, Bi M, Sun Z, et al. Screening of differentially expressed genes associated with human glioblastoma and functional analysis using a DNA microarray. Mol Med Rep. 2015;12(2):1991–6. Epub 2015/04/23. doi: 10.3892/mmr.2015.3659 . 25901754

[31] Fares J, Ulasov I, Timashev P, Lesniak MS. Emerging principles of brain immunology and immune checkpoint blockade in brain metastases. Brain. 2021;144(4):1046–66. Epub 2021/04/25. doi: 10.1093/brain/awab012 ; PubMed Central PMCID: PMC8105040. 33893488 PMC8105040

[32] Sielska M, Przanowski P, Pasierbinska M, Wojnicki K, Poleszak K, Wojtas B, et al. Tumour-derived CSF2/granulocyte macrophage colony stimulating factor controls myeloid cell accumulation and progression of gliomas. Br J Cancer. 2020;123(3):438–48. Epub 2020/05/12. doi: 10.1038/s41416-020-0862-2 ; PubMed Central PMCID: PMC7403321. 32390004 PMC7403321

[33] Kenkel JA, Tseng WW, Davidson MG, Tolentino LL, Choi O, Bhattacharya N, et al. An Immunosuppressive Dendritic Cell Subset Accumulates at Secondary Sites and Promotes Metastasis in Pancreatic Cancer. Cancer Res. 2017;77(15):4158–70. Epub 2017/06/15. doi: 10.1158/0008-5472.CAN-16-2212 ; PubMed Central PMCID: PMC5550516. 28611041 PMC5550516

[34] Bayik D, Lathia JD. Cancer stem cell-immune cell crosstalk in tumour progression. Nat Rev Cancer. 2021;21(8):526–36. Epub 2021/06/10. doi: 10.1038/s41568-021-00366-w ; PubMed Central PMCID: PMC8740903. 34103704 PMC8740903

[35] Rempe RG, Hartz AMS, Bauer B. Matrix metalloproteinases in the brain and blood-brain barrier: Versatile breakers and makers. J Cereb Blood Flow Metab. 2016;36(9):1481–507. Epub 2016/06/22. doi: 10.1177/0271678X16655551 ; PubMed Central PMCID: PMC5012524. 27323783 PMC5012524

[36] Wang W, Chu HJ, Liang YC, Huang JM, Shang CL, Tan H, et al. FABP5 correlates with poor prognosis and promotes tumor cell growth and metastasis in cervical cancer. Tumour Biol. 2016;37(11):14873–83. Epub 2016/09/21. doi: 10.1007/s13277-016-5350-1 . 27644245

[37] Wu G, Xu Y, Wang Q, Li J, Li L, Han C, et al. FABP5 is correlated with poor prognosis and promotes tumour cell growth and metastasis in clear cell renal cell carcinoma. Eur J Pharmacol. 2019;862:172637. Epub 2019/09/07. doi: 10.1016/j.ejphar.2019.172637 . 31491402

[38] Sevenich L, Bowman RL, Mason SD, Quail DF, Rapaport F, Elie BT, et al. Analysis of tumour- and stroma-supplied proteolytic networks reveals a brain-metastasis-promoting role for cathepsin S. Nat Cell Biol. 2014;16(9):876–88. Epub 2014/08/05. doi: 10.1038/ncb3011 ; PubMed Central PMCID: PMC4249762. 25086747 PMC4249762

[39] Israelov H, Ravid O, Atrakchi D, Rand D, Elhaik S, Bresler Y, et al. Caspase-1 has a critical role in blood-brain barrier injury and its inhibition contributes to multifaceted repair. J Neuroinflammation. 2020;17(1):267. Epub 2020/09/11. doi: 10.1186/s12974-020-01927-w ; PubMed Central PMCID: PMC7488082. 32907600 PMC7488082

[40] Shinde A, Paez JS, Libring S, Hopkins K, Solorio L, Wendt MK. Transglutaminase-2 facilitates extracellular vesicle-mediated establishment of the metastatic niche. Oncogenesis. 2020;9(2):16. Epub 2020/02/15. doi: 10.1038/s41389-020-0204-5 ; PubMed Central PMCID: PMC7018754. 32054828 PMC7018754

[41] Arden JD, Lavik KI, Rubinic KA, Chiaia N, Khuder SA, Howard MJ, et al. Small-molecule agonists of mammalian Diaphanous-related (mDia) formins reveal an effective glioblastoma anti-invasion strategy. Mol Biol Cell. 2015;26(21):3704–18. Epub 2015/09/12. doi: 10.1091/mbc.E14-11-1502 ; PubMed Central PMCID: PMC4626057. 26354425 PMC4626057

[42] Chen Y, Cairns R, Papandreou I, Koong A, Denko NC. Oxygen consumption can regulate the growth of tumors, a new perspective on the Warburg effect. PLoS One. 2009;4(9):e7033. Epub 2009/09/16. doi: 10.1371/journal.pone.0007033 ; PubMed Central PMCID: PMC2737639. 19753307 PMC2737639

[43] Xiong N, Gao X, Zhao H, Cai F, Zhang FC, Yuan Y, et al. Using arterial-venous analysis to characterize cancer metabolic consumption in patients. Nat Commun. 2020;11(1):3169. Epub 2020/06/25. doi: 10.1038/s41467-020-16810-8 ; PubMed Central PMCID: PMC7311411. 32576825 PMC7311411

[44] Warburg O. On respiratory impairment in cancer cells. Science. 1956;124(3215):269–70. Epub 1956/08/10. . 13351639

[45] Penhoet EE, Kochman M, Rutter WJ. Molecular and catalytic properties of aldolase C. Biochemistry. 1969;8(11):4396–402. Epub 1969/11/01. doi: 10.1021/bi00839a026 . 4982047

[46] Fujita H, Aoki H, Ajioka I, Yamazaki M, Abe M, Oh-Nishi A, et al. Detailed expression pattern of aldolase C (Aldoc) in the cerebellum, retina and other areas of the CNS studied in Aldoc-Venus knock-in mice. PLoS One. 2014;9(1):e86679. Epub 2014/01/30. doi: 10.1371/journal.pone.0086679 ; PubMed Central PMCID: PMC3903578. 24475166 PMC3903578

[47] Izraely S, Ben-Menachem S, Sagi-Assif O, Meshel T, Malka S, Telerman A, et al. The melanoma brain metastatic microenvironment: aldolase C partakes in shaping the malignant phenotype of melanoma cells ‐ a case of inter-tumor heterogeneity. Mol Oncol. 2020. Epub 2020/12/05. doi: 10.1002/1878-0261.12872 . 33274599 PMC8096793

[48] Welinder C, Pawlowski K, Szasz AM, Yakovleva M, Sugihara Y, Malm J, et al. Correlation of histopathologic characteristics to protein expression and function in malignant melanoma. PLoS One. 2017;12(4):e0176167. Epub 2017/04/27. doi: 10.1371/journal.pone.0176167 ; PubMed Central PMCID: PMC5405986. 28445515 PMC5405986

[49] George Warren W, Osborn M, Yates A, Wright K, O’Sullivan SE. The emerging role of fatty acid binding protein 5 (FABP5) in cancers. Drug Discov Today. 2023;28(7):103628. Epub 2023/05/26. doi: 10.1016/j.drudis.2023.103628 . 37230284

[50] Wu X, Geng F, Cheng X, Guo Q, Zhong Y, Cloughesy TF, et al. Lipid Droplets Maintain Energy Homeostasis and Glioblastoma Growth via Autophagic Release of Stored Fatty Acids. iScience. 2020;23(10):101569. Epub 2020/10/22. doi: 10.1016/j.isci.2020.101569 ; PubMed Central PMCID: PMC7549116. 33083736 PMC7549116

