# Supplementary figures and images for: Proteomic and transcriptomic characterisation of FIA10, a novel murine leukemic cell line that metastasizes into the brain
Source: PLoS One. 2024 Jan 12;19(1):e0295641. doi: 10.1371/journal.pone.0295641 (PMC10786371; doi:10.1371/journal.pone.0295641)

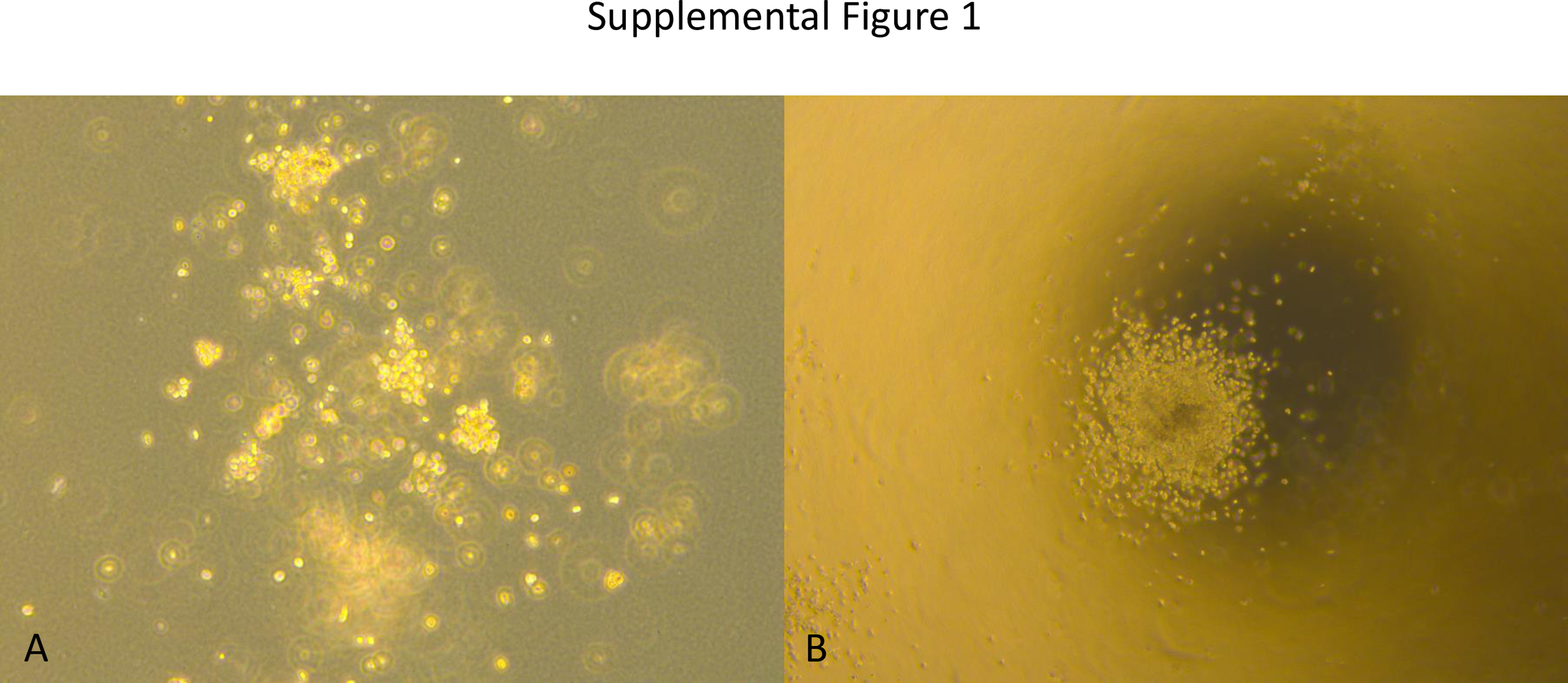

Supplement: S1 Fig — (A) Multicenter colony of FIA10 cells. (B) Compact colony of FIA10 cells. (TIFF) [file pone.0295641.s001.tiff]

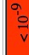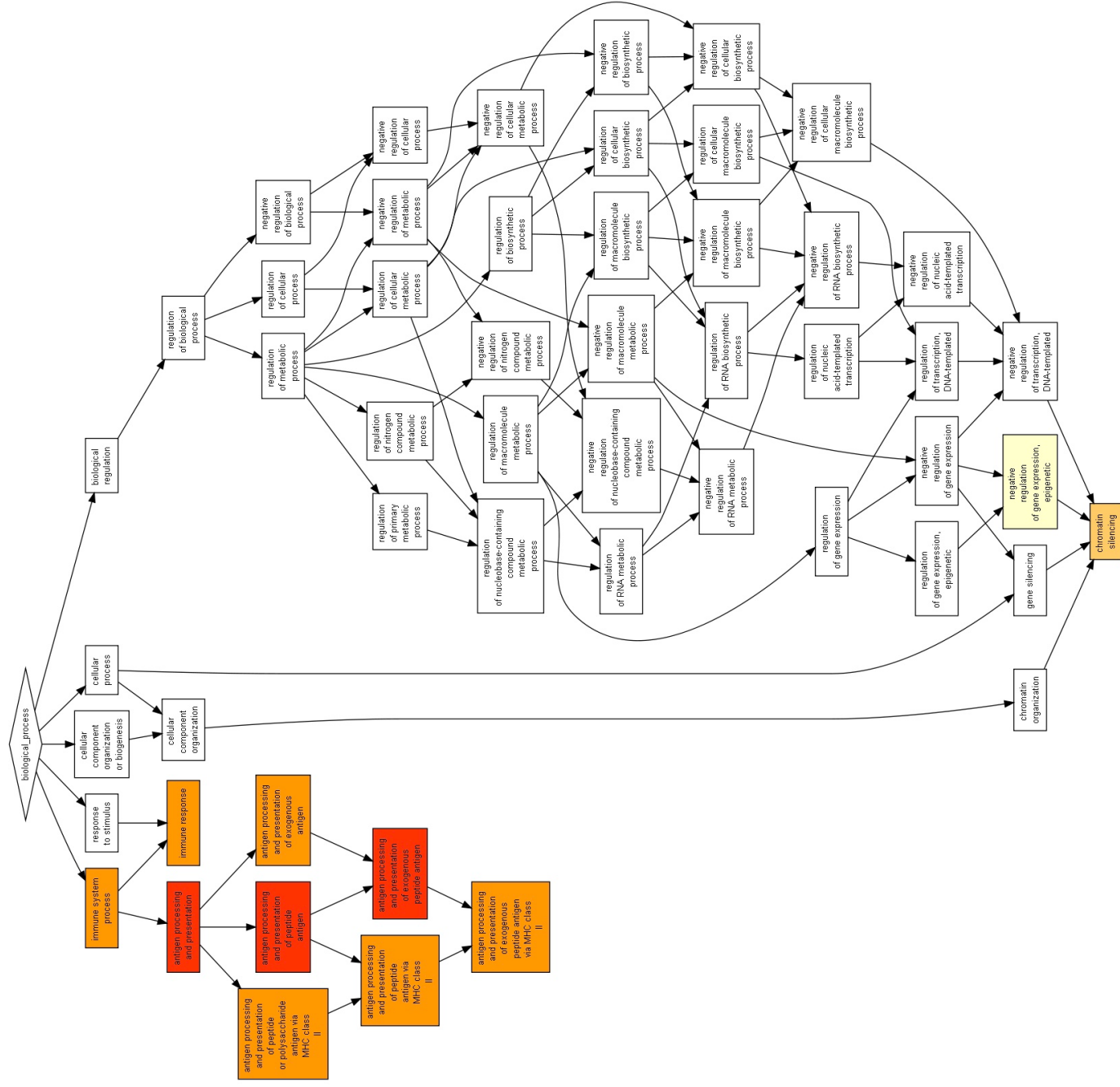

Supplement: S3 Fig — Differentially expressed proteins were ranked according to their p-values of differential expression and degree of enrichment compared with the total number of expressed genes analysed. (PDF) [file pone.0295641.s003.pdf]

*P-value color scale*

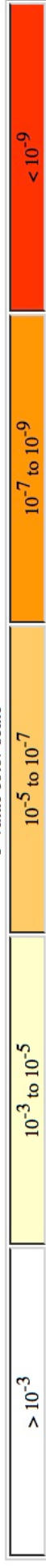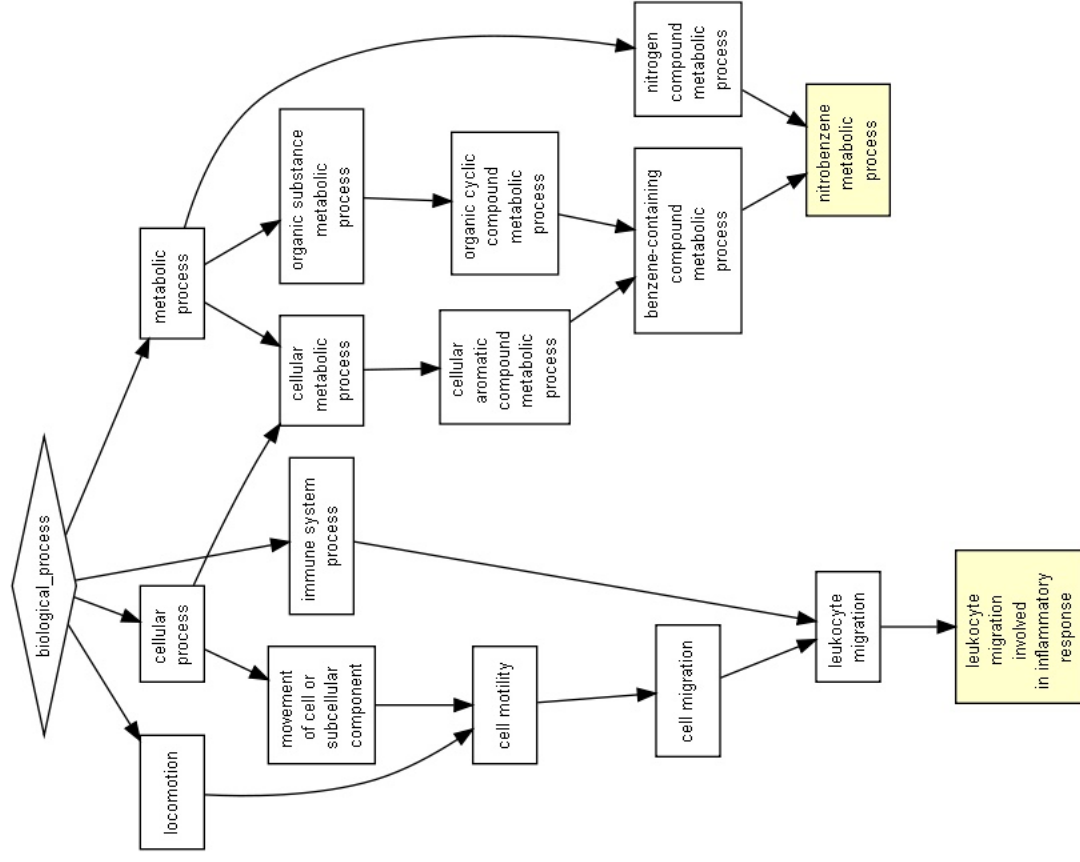

Supplement: S5 Fig — Differentially expressed proteins were ranked according to their p-values of differential expression and degree of enrichment compared with the total number of expressed genes analysed. (PDF) [file pone.0295641.s005.pdf]
