# Supplementary material for: Proteomic and transcriptomic characterisation of FIA10, a novel murine leukemic cell line that metastasizes into the brain
Source: PLoS One. 2024 Jan 12;19(1):e0295641. doi: 10.1371/journal.pone.0295641 (PMC10786371; doi:10.1371/journal.pone.0295641)
Supplement: S1 Table — (DOCX) [file pone.0295641.s006.docx]

**S1 Table. Primer sequences**

GAPDH forward CTCTCTGCTCCTCCTGTTCGAC

GAPDH reverse TGAGCGATGTGGCTCGGCT

CCL2 forward CATCCACGTGTTGGCTCA

CCL2 reverse GATCATCTTGCTGGTGAATGAGT

CCL3 forward TGCCCTTGCTGTTCTTCTCT

CCL3 reverse GTGGAATCTTCCGGCTGTTAG

CTSB forward GTGTCTGCTGAAGACCTGCTT

CTSB reverse GGGATAGCCACCATTACAGC

EGR3 forward CAATCTGTACCCCGAGGAGA

EGR3 reverse CCGATGTCCATCACATTCTCT

EVL forward GAGATCCTGGAGGAGGTGGT

EVL reverse ACTTAGCTCCTGCCTGATGG

HCK forward AGCCACTGCCAAAACTCATT

HCK reverse GCTCAATGAAGGCCATGC

LPP forward GGCTTTCCCTGTGCTAAACC

LPP reverse TGGTGGCTAGCTAGGAGGAA

MMP8 forward AATGGCATTCAGACAATCTATGG

MMP8 reverse AATCTCAGGTGGGGGTCAC

MMP12 forward TTGTGGATAAACACTACTGGAGGT

MMP12 reverse AAATCAGCTTGGGGTAAGCA

SAA3 forward ATGCTCGGGGGAACTATGAT

SAA3 reverse ACAGCCTCTCTGGCATCG

DAB2IP forward CCCCACAGGGATAGGCTAAG

DAB2IP reverse TTGTAGCACTGCCAGATCCTT

G0S2 forward TCTCTTCCCACTGCACCCTA

G0S2 reverse TCCTGCACACTTTCCATCTG

HTATIP2 forward TCATGATGTTGGATTCTGTTGC

HTATIP2 reverse GTCAACACGAACAAACCCTTC

IL1R2 forward TCTGGTACCTACATTTGCACATTC

IL1R2 reverse AAAGACCTTGAGTTCCACAGACA

MTUS1 forward GGCTGTGTTAGAGATCAAGAATGA

MTUS1 reverse CCAATGCTGTGTTATTGTCCA

NDGR1 forward GTGCAGGGCATGGGATAC

NDGR1 reverse TCCAGGGATGTGACACTGG

PKB2 forward TCCCAGTGTGAAAAAGACTGC

PKB2 reverse ACCAAATCTGGTAGAGTTTCTTTAGC

SERPINF1 forward GGACTCTGATCTCAACTGCAAG

SERPINF1 reverse AAGTTCTGGGTCACGGTCAG

TGFBI forward GAAGGGAGACAATCGTTTTAGC

TGFBI reverse CGGTTGAGGATCTCCATGA

THBS1 forward TCGGCTGGAAAGATTTCACT

THBS1 reverse TCCTTCATACATACATCACCACTCTGA
