# Supplementary material for: Proteomic and transcriptomic characterisation of FIA10, a novel murine leukemic cell line that metastasizes into the brain
Source: PLoS One. 2024 Jan 12;19(1):e0295641. doi: 10.1371/journal.pone.0295641 (PMC10786371; doi:10.1371/journal.pone.0295641)
Supplement: S5 Table — (DOCX) [file pone.0295641.s010.docx]

**S5 Table. qPCR validation of FIA10 and FIA18 mRNA Expression**

FIA10 cells FIA18 cells T-Test

mRNA expression mRNA expression FIA10/FIA18

-------------------------------------------- -------------------------------------------- ----------------

#1 #2 #3 mean SD #1 #2 #3 mean SD p-value

Gene

Casp1 6.899 6.632 6.809 6.780 0.111 2.620 3.739 3.444 3.268 0.473 0.007

Ccl2 4.109 3.603 5.855 4.522 0.965 0.407 0.591 0.601 0.533 0.089 0.027

Ctsb 2.930 3.235 3.310 3.158 0.164 1.160 2.125 1.480 1.588 0.401 0.019

Egr3 1.357 0.944 0.755 1.019 0.251 0.149 0.043 0.142 0.111 0.048 0.032

Evl 2.083 1.455 2.350 1.963 0.375 0.390 0.235 0.354 0.326 0.066 0.023

Hck 3.887 3.569 3.664 3.707 0.134 0.019 0.014 0.044 0.026 0.013 0.001

Lpp 1.754 1.428 1.651 1.611 0.136 0.804 0.774 1.041 0.873 0.120 0.005

Mmp8 20.568 18.719 18.719 19.335 0.872 9.019 7.572 8.870 8.487 0.650 0.001

Mmp12 100.0 104.56 135.85 113.47 15.94 1.120 1.269 1.178 1.189 0.061 0.010

Saa3 7.295 8.015 7.450 7.587 0.309 2.405 4.400 3.545 3.450 0.817 0.011

Dab2ip 0.049 0.087 0.099 0.078 0.022 0.395 0.572 0.425 0.464 0.077 0.014

G0s2 0.088 0.120 0.106 0.105 0.013 1.739 3.169 1.796 2.235 0.661 0.045

Htatip2 2.321 2.252 2.672 2.415 0.184 6.165 8.682 6.248 7.031 1.167 0.028

Il1r2 0.758 0.930 0.930 0.873 0.081 6.555 6.035 6.035 6.208 0.245 0.001

Mtus1 0.952 0.924 1.316 1.064 0.179 3.121 4.120 2.931 3.391 0.522 0.016

Ndrg1 1.601 1.925 1.956 1.827 0.161 4.175 5.597 5.597 5.123 0.670 0.016

Pkb2 0.230 0.212 0.261 0.234 0.020 1.432 1.632 1.830 1.631 0.162 0.006

Serpinf1 0.045 0.066 0.076 0.062 0.013 0.489 0.727 0.493 0.570 0.570 0.022

Tgfbi 0.018 0.017 0.018 0.018 0.001 0.315 0.458 0.345 0.372 0.372 0.015

Thbs1 1.365 1.253 1.797 1.472 0.234 44.379 62.020 36.430 47.610 10.694 0.025

Cells were cultured in IMDM, 20 % HS in the presence of 5% CO_2_ and 5% O_2_. Three replicates per experiment.

SD: Standard deviation.
