# Supplementary material for: Proteomic and transcriptomic characterisation of FIA10, a novel murine leukemic cell line that metastasizes into the brain
Source: PLoS One. 2024 Jan 12;19(1):e0295641. doi: 10.1371/journal.pone.0295641 (PMC10786371; doi:10.1371/journal.pone.0295641)
Supplement: S6 Table — (DOCX) [file pone.0295641.s011.docx]

**Gene Ontology: Biological process FIA10 vs FIA18 RNA upregulated**

| **GO term** | **Description** | **P-value** | **FDR q-value** | **Enrichment (N, B, n, b)** | **Genes** |
| --- | --- | --- | --- | --- | --- |
| GO:0002376 | immune system process | 5.25E-20 | 8.06E-16 | 3.64 (15721,1430,184,61) | Trf - transferrin  Fpr1 - formyl peptide receptor 1  Fpr2 - formyl peptide receptor 2  Cd74 - cd74 antigen (invariant polypeptide of major histocompatibility complex, class ii antigen-associated)  Prkcb - protein kinase c, beta  H2-Oa - histocompatibility 2, o region alpha locus  Alcam - activated leukocyte cell adhesion molecule  H2-M3 - histocompatibility 2, m region locus 3  H2-DMa - histocompatibility 2, class ii, locus dma  Ccl2 - chemokine (c-c motif) ligand 2  Itgax - integrin alpha x  Ear2 - eosinophil-associated, ribonuclease a family, member 2  Pde4b - phosphodiesterase 4b, camp specific  Ccl17 - chemokine (c-c motif) ligand 17  Tnfrsf1b - tumor necrosis factor receptor superfamily, member 1b  Clec4n - c-type lectin domain family 4, member n  Slc11a1 - solute carrier family 11 (proton-coupled divalent metal ion transporters), member 1  Ifi205 - interferon activated gene 205  Cd34 - cd34 antigen  Polr3k - polymerase (rna) iii (dna directed) polypeptide k P2ry14 - purinergic receptor p2y, g-protein coupled, 14  Ccrl2 - chemokine (c-c motif) receptor-like 2  Pld4 - phospholipase d family, member 4  Tnfaip3 - tumor necrosis factor, alpha-induced protein 3 AF251705 - cdna sequence af251705  Icam1 - intercellular adhesion molecule 1  Trem2 - triggering receptor expressed on myeloid cells 2  Fgr - gardner-rasheed feline sarcoma viral (fgr) oncogene homolog  Cd274 - cd274 antigen  Fgl2 - fibrinogen-like protein 2  Ifi30 - interferon gamma inducible protein 30  Hck - hemopoietic cell kinase  Slc15a3 - solute carrier family 15, member 3  Cxcl2 - chemokine (c-x-c motif) ligand 2  Csf1r - colony stimulating factor 1 receptor  Ifi203 - interferon activated gene 203  Csf2 - colony stimulating factor 2 (granulocyte-macrophage)  Ccl3 - chemokine (c-c motif) ligand 3  Ccl4 - chemokine (c-c motif) ligand 4  Irg1 - immunoresponsive gene 1  Bmi1 - bmi1 polycomb ring finger oncogene  Ccl5 - chemokine (c-c motif) ligand 5  Csf1 - colony stimulating factor 1 (macrophage)  Ctss - cathepsin s  H2-D1 - histocompatibility 2, d region locus 1  Ccr5 - chemokine (c-c motif) receptor 5  Lgals3 - lectin, galactose binding, soluble 3  Il7r - interleukin 7 receptor  H2-Ab1 - histocompatibility 2, class ii antigen a, beta 1  H2-Aa - histocompatibility 2, class ii antigen a, alpha  Casp1 - caspase 1  Sirpa - signal-regulatory protein alpha  Epx - eosinophil peroxidase  Wfdc17 - wap four-disulfide core domain 17  Thy1 - thymus cell antigen 1, theta  H2-K1 - histocompatibility 2, k1, k region  H2-Eb1 - histocompatibility 2, class ii antigen e beta Kdm5d - lysine (k)-specific demethylase 5d  Il1rn - interleukin 1 receptor antagonist  Pld3 - phospholipase d family, member 3  Prg2 - proteoglycan 2, bone marrow |
|  | regulation of immune system process |  |  |  | Fpr1 - formyl peptide receptor 1  Fpr2 - formyl peptide receptor 2  Cd74 - cd74 antigen (invariant polypeptide of major histocompatibility complex, class ii antigen-associated)  Prkcb - protein kinase c, beta  Cd9 - cd9 antigen  H2-Oa - histocompatibility 2, o region alpha locus  Mmp2 - matrix metallopeptidase 2  Mmp12 - matrix metallopeptidase 12  H2-M3 - histocompatibility 2, m region locus 3  P2rx4 - purinergic receptor p2x, ligand-gated ion channel 4 H2-DMa - histocompatibility 2, class ii, locus dma  Gpnmb - glycoprotein (transmembrane) nmb  Ccl2 - chemokine (c-c motif) ligand 2  Pde4b - phosphodiesterase 4b, camp specific  Tnfrsf1b - tumor necrosis factor receptor superfamily, member 1b  Alox15 - arachidonate 15-lipoxygenase  Slc11a1 - solute carrier family 11 (proton-coupled divalent metal ion transporters), member 1  Mmp8 - matrix metallopeptidase 8  Ifi205 - interferon activated gene 205 |
| GO:0002682 | regulation of immune system process | 9.02E-18 | 6.92E-14 | 3.69 (15721,1252,184,54) | Mpp1 - membrane protein, palmitoylated  Padi2 - peptidyl arginine deiminase, type ii  Lpxn - leupaxin  Tnfaip3 - tumor necrosis factor, alpha-induced protein 3 Ikbke - inhibitor of kappab kinase epsilon  Cebpa - ccaat/enhancer binding protein (c/ebp), alpha Icam1 - intercellular adhesion molecule 1  Inpp4b - inositol polyphosphate-4-phosphatase, type ii Trem2 - triggering receptor expressed on myeloid cells 2 Cst7 - cystatin f (leukocystatin)  Cd274 - cd274 antigen  Fgr - gardner-rasheed feline sarcoma viral (fgr) oncogene homolog  Fgl2 - fibrinogen-like protein 2  Il1rl1 - interleukin 1 receptor-like 1  Slc15a3 - solute carrier family 15, member 3  Csf1r - colony stimulating factor 1 receptor  Ifi203 - interferon activated gene 203  Clec7a - c-type lectin domain family 7, member a  Ccl3 - chemokine (c-c motif) ligand 3  Ccl4 - chemokine (c-c motif) ligand 4  Irg1 - immunoresponsive gene 1  Bmi1 - bmi1 polycomb ring finger oncogene  Ccl5 - chemokine (c-c motif) ligand 5  Csf1 - colony stimulating factor 1 (macrophage)  H2-D1 - histocompatibility 2, d region locus 1  Lgals3 - lectin, galactose binding, soluble 3  Il7r - interleukin 7 receptor  H2-Ab1 - histocompatibility 2, class ii antigen a, beta 1  H2-Aa - histocompatibility 2, class ii antigen a, alpha  Sirpa - signal-regulatory protein alpha  Thy1 - thymus cell antigen 1, theta  H2-K1 - histocompatibility 2, k1, k region  Inhba - inhibin beta-a  Runx1 - runt related transcription factor 1  Egr3 - early growth response 3 |
| GO:0006955 | immune response | 1.18E-17 | 6.02E-14 | 4.64 (15721,792,184,43) | Icam1 - intercellular adhesion molecule 1  Trf - transferrin  Trem2 - triggering receptor expressed on myeloid cells 2  Fgr - gardner-rasheed feline sarcoma viral (fgr) oncogene homolog  Cd274 - cd274 antigen  Hck - hemopoietic cell kinase  Prkcb - protein kinase c, beta  Slc15a3 - solute carrier family 15, member 3  Cd74 - cd74 antigen (invariant polypeptide of major histocompatibility complex, class ii antigen-associated)  Cxcl2 - chemokine (c-x-c motif) ligand 2  Csf1r - colony stimulating factor 1 receptor  Csf2 - colony stimulating factor 2 (granulocyte-macrophage)  Alcam - activated leukocyte cell adhesion molecule  Ccl3 - chemokine (c-c motif) ligand 3  H2-M3 - histocompatibility 2, m region locus 3  Ccl4 - chemokine (c-c motif) ligand 4  Irg1 - immunoresponsive gene 1  H2-DMa - histocompatibility 2, class ii, locus dma  Bmi1 - bmi1 polycomb ring finger oncogene  Csf1 - colony stimulating factor 1 (macrophage)  Ccl5 - chemokine (c-c motif) ligand 5  Ctss - cathepsin s  Ear2 - eosinophil-associated, ribonuclease a family, member 2  Ccl2 - chemokine (c-c motif) ligand 2  Lgals3 - lectin, galactose binding, soluble 3  Ccr5 - chemokine (c-c motif) receptor 5  H2-D1 - histocompatibility 2, d region locus 1  Ccl17 - chemokine (c-c motif) ligand 17  Tnfrsf1b - tumor necrosis factor receptor superfamily, member 1b  H2-Ab1 - histocompatibility 2, class ii antigen a, beta 1  Clec4n - c-type lectin domain family 4, member n  H2-Aa - histocompatibility 2, class ii antigen a, alpha Wfdc17 - wap four-disulfide core domain 17  Slc11a1 - solute carrier family 11 (proton-coupled divalent metal ion transporters), member 1  H2-K1 - histocompatibility 2, k1, k region  H2-Eb1 - histocompatibility 2, class ii antigen e beta  Il1rn - interleukin 1 receptor antagonist  Polr3k - polymerase (rna) iii (dna directed) polypeptide k  P2ry14 - purinergic receptor p2y, g-protein coupled, 14 Ccrl2 - chemokine (c-c motif) receptor-like 2  Pld3 - phospholipase d family, member 3  Pld4 - phospholipase d family, member 4  Prg2 - proteoglycan 2, bone marrow |
| GO:0002684 | positive regulation of immune system process | 3.44E-16 | 1.32E-12 | 4.33 (15721,828,184,42) | Ikbke - inhibitor of kappab kinase epsilon  Cebpa - ccaat/enhancer binding protein (c/ebp), alpha Icam1 - intercellular adhesion molecule 1  Trem2 - triggering receptor expressed on myeloid cells 2  Fgr - gardner-rasheed feline sarcoma viral (fgr) oncogene homolog  Cd274 - cd274 antigen  Fpr1 - formyl peptide receptor 1  Il1rl1 - interleukin 1 receptor-like 1  Fpr2 - formyl peptide receptor 2  Slc15a3 - solute carrier family 15, member 3  Prkcb - protein kinase c, beta  Cd74 - cd74 antigen (invariant polypeptide of major histocompatibility complex, class ii antigen-associated)  Mmp2 - matrix metallopeptidase 2  Csf1r - colony stimulating factor 1 receptor  Ifi203 - interferon activated gene 203  Clec7a - c-type lectin domain family 7, member a  Mmp12 - matrix metallopeptidase 12  Ccl3 - chemokine (c-c motif) ligand 3  H2-M3 - histocompatibility 2, m region locus 3  Ccl4 - chemokine (c-c motif) ligand 4  Irg1 - immunoresponsive gene 1  P2rx4 - purinergic receptor p2x, ligand-gated ion channel 4 Bmi1 - bmi1 polycomb ring finger oncogene  H2-DMa - histocompatibility 2, class ii, locus dma  Ccl5 - chemokine (c-c motif) ligand 5  Csf1 - colony stimulating factor 1 (macrophage)  Ccl2 - chemokine (c-c motif) ligand 2  Pde4b - phosphodiesterase 4b, camp specific  Lgals3 - lectin, galactose binding, soluble 3  H2-D1 - histocompatibility 2, d region locus 1  Il7r - interleukin 7 receptor  H2-Ab1 - histocompatibility 2, class ii antigen a, beta 1  H2-Aa - histocompatibility 2, class ii antigen a, alpha  Sirpa - signal-regulatory protein alpha  Thy1 - thymus cell antigen 1, theta  Slc11a1 - solute carrier family 11 (proton-coupled divalent metal ion transporters), member 1  Mmp8 - matrix metallopeptidase 8  H2-K1 - histocompatibility 2, k1, k region  Ifi205 - interferon activated gene 205  Inhba - inhibin beta-a  Runx1 - runt related transcription factor 1  Egr3 - early growth response 3 |
| GO:0001817 | regulation of cytokine production | 5.91E-15 | 1.81E-11 | 4.69 (15721,656,184,36) | Rnf128 - ring finger protein 128  AF251705 - cdna sequence af251705  Trem2 - triggering receptor expressed on myeloid cells 2 Cd274 - cd274 antigen  Fgr - gardner-rasheed feline sarcoma viral (fgr) oncogene homolog  Il1rl1 - interleukin 1 receptor-like 1  Cd74 - cd74 antigen (invariant polypeptide of major histocompatibility complex, class ii antigen-associated)  Csf1r - colony stimulating factor 1 receptor  Csf2 - colony stimulating factor 2 (granulocyte-macrophage)  Clec7a - c-type lectin domain family 7, member a  Ccl3 - chemokine (c-c motif) ligand 3  Mmp12 - matrix metallopeptidase 12  Ccl4 - chemokine (c-c motif) ligand 4  H2-M3 - histocompatibility 2, m region locus 3  Irg1 - immunoresponsive gene 1  Gpnmb - glycoprotein (transmembrane) nmb  Ccl5 - chemokine (c-c motif) ligand 5  Ccl2 - chemokine (c-c motif) ligand 2  Ccr5 - chemokine (c-c motif) receptor 5  Pde4b - phosphodiesterase 4b, camp specific  Ndrg2 - n-myc downstream regulated gene 2  Tnfrsf1b - tumor necrosis factor receptor superfamily, member 1b  Clec4n - c-type lectin domain family 4, member n  Sirpa - signal-regulatory protein alpha  Casp1 - caspase 1  Epx - eosinophil peroxidase  Tnfrsf9 - tumor necrosis factor receptor superfamily, member 9  Slc11a1 - solute carrier family 11 (proton-coupled divalent metal iontransporters), member 1  Mmp8 - matrix metallopeptidase 8  Ifi205 - interferon activated gene 205  Cd34 - cd34 antigen  Pld3 - phospholipase d family, member 3  Pld4 - phospholipase d family, member 4  Runx1 - runt related transcription factor 1  Tnfaip3 - tumor necrosis factor, alpha-induced protein 3  Prg2 - proteoglycan 2, bone marrow |
| GO:0034341 | response to interferon-gamma | 6.95E-14 | 1.78E-10 | 13.14 (15721,104,184,16) | Ccl2 - chemokine (c-c motif) ligand 2  Ccl17 - chemokine (c-c motif) ligand 17  Mrc1 - mannose receptor, c type 1  H2-Ab1 - histocompatibility 2, class ii antigen a, beta 1  H2-Aa - histocompatibility 2, class ii antigen a, alpha  Sirpa - signal-regulatory protein alpha  Casp1 - caspase 1  Slc11a1 - solute carrier family 11 (proton-coupled divalent metal ion transporters), member 1  H2-Eb1 - histocompatibility 2, class ii antigen e beta  Cd74 - cd74 antigen (invariant polypeptide of major histocompatibility complex, class ii antigen-associated)  Evl - ena-vasodilator stimulated phosphoprotein  Ccl3 - chemokine (c-c motif) ligand 3  Ccl4 - chemokine (c-c motif) ligand 4  Gch1 - gtp cyclohydrolase 1  Irg1 - immunoresponsive gene 1  Ccl5 - chemokine (c-c motif) ligand 5 |
| GO:0048002 | antigen processing and presentation of peptide antigen | 9.46E-14 | 2.07E-10 | 22.78 (15721,45,184,12) | Cd74 - cd74 antigen (invariant polypeptide of major histocompatibility complex, class ii antigen-associated)  H2-D1 - histocompatibility 2, d region locus 1  H2-Oa - histocompatibility 2, o region alpha locus  H2-Ab1 - histocompatibility 2, class ii antigen a, beta 1  H2-Aa - histocompatibility 2, class ii antigen a, alpha  H2-M3 - histocompatibility 2, m region locus 3  Slc11a1 - solute carrier family 11 (proton-coupled divalent metal ion transporters), member 1  H2-DMa - histocompatibility 2, class ii, locus dma  H2-K1 - histocompatibility 2, k1, k region  H2-Eb1 - histocompatibility 2, class ii antigen e beta  Ctss - cathepsin s  Ifi30 - interferon gamma inducible protein 30 |
| GO:0034097 | response to cytokine | 9.75E-14 | 1.87E-10 | 5.02 (15721,528,184,31) | Mrc1 - mannose receptor, c type 1  Ikbke - inhibitor of kappab kinase epsilon  Cebpa - ccaat/enhancer binding protein (c/ebp), alpha Icam1 - intercellular adhesion molecule 1  Ifi202b - interferon activated gene 202b  Cd274 - cd274 antigen  Cd74 - cd74 antigen (invariant polypeptide of major histocompatibility complex, class ii antigen-associated)  Csf1r - colony stimulating factor 1 receptor  Csf2ra - colony stimulating factor 2 receptor, alpha, low-affinity (granulocyte-macrophage)  Ifi203 - interferon activated gene 203  Csf2 - colony stimulating factor 2 (granulocyte-macrophage)  Socs3 - suppressor of cytokine signaling 3  Evl - ena-vasodilator stimulated phosphoprotein  Snx10 - sorting nexin 10  Ccl3 - chemokine (c-c motif) ligand 3  Ccl4 - chemokine (c-c motif) ligand 4  Gch1 - gtp cyclohydrolase 1  Irg1 - immunoresponsive gene 1  Saa3 - serum amyloid a 3  Ccl5 - chemokine (c-c motif) ligand 5  Ccl2 - chemokine (c-c motif) ligand 2  Ccl17 - chemokine (c-c motif) ligand 17  H2-Ab1 - histocompatibility 2, class ii antigen a, beta 1  H2-Aa - histocompatibility 2, class ii antigen a, alpha  Sirpa - signal-regulatory protein alpha  Casp1 - caspase 1  Alox15 - arachidonate 15-lipoxygenase  Slc11a1 - solute carrier family 11 (proton-coupled divalent metal ion transporters), member 1  H2-Eb1 - histocompatibility 2, class ii antigen e beta  Ifi205 - interferon activated gene 205  Padi2 - peptidyl arginine deiminase, type ii |
| GO:0002478 | antigen processing and presentation of exogenous peptide antigen | 1.7E-13 | 2.91E-10 | 32.86 (15721,26,184,10) | Cd74 - cd74 antigen (invariant polypeptide of major histocompatibility complex, class ii antigen-associated)  H2-Oa - histocompatibility 2, o region alpha locus  H2-Ab1 - histocompatibility 2, class ii antigen a, beta 1  H2-Aa - histocompatibility 2, class ii antigen a, alpha  H2-M3 - histocompatibility 2, m region locus 3  H2-DMa - histocompatibility 2, class ii, locus dma  H2-K1 - histocompatibility 2, k1, k region  H2-Eb1 - histocompatibility 2, class ii antigen e beta  Ctss - cathepsin s  Ifi30 - interferon gamma inducible protein 30 |
| GO:0031347 | regulation of defense response | 2.22E-13 | 3.41E-10 | 4.55 (15721,620,184,33) | Mgll - monoglyceride lipase  Ikbke - inhibitor of kappab kinase epsilon  Cebpa - ccaat/enhancer binding protein (c/ebp), alpha Trem2 - triggering receptor expressed on myeloid cells 2 Cst7 - cystatin f (leukocystatin)  Fgr - gardner-rasheed feline sarcoma viral (fgr) oncogene homolog  Fgl2 - fibrinogen-like protein 2  Il1rl1 - interleukin 1 receptor-like 1  Fpr2 - formyl peptide receptor 2  Slc15a3 - solute carrier family 15, member 3  Cd74 - cd74 antigen (invariant polypeptide of major histocompatibility complex, class ii antigen-associated)  Mmp2 - matrix metallopeptidase 2  Ifi203 - interferon activated gene 203  Socs3 - suppressor of cytokine signaling 3  Clec7a - c-type lectin domain family 7, member a  Mmp12 - matrix metallopeptidase 12  Ccl3 - chemokine (c-c motif) ligand 3  H2-M3 - histocompatibility 2, m region locus 3  Irg1 - immunoresponsive gene 1  Ccl5 - chemokine (c-c motif) ligand 5  Ctss - cathepsin s  Ccr5 - chemokine (c-c motif) receptor 5  Cd200r3 - cd200 receptor 3  Tnfrsf1b - tumor necrosis factor receptor superfamily, member 1b  Sirpa - signal-regulatory protein alpha  Casp1 - caspase 1  Alox15 - arachidonate 15-lipoxygenase  Mmp8 - matrix metallopeptidase 8  Ifi205 - interferon activated gene 205  Tgm2 - transglutaminase 2, c polypeptide  Pld3 - phospholipase d family, member 3  Pld4 - phospholipase d family, member 4  Tnfaip3 - tumor necrosis factor, alpha-induced protein 3 |
| GO:0019882 | antigen processing and presentation | 4.23E-13 | 5.9E-10 | 14.95 (15721,80,184,14) | H2-D1 - histocompatibility 2, d region locus 1  H2-Ab1 - histocompatibility 2, class ii antigen a, beta 1  H2-Aa - histocompatibility 2, class ii antigen a, alpha  Icam1 - intercellular adhesion molecule 1  Slc11a1 - solute carrier family 11 (proton-coupled divalent metal ion transporters), member 1  H2-K1 - histocompatibility 2, k1, k region  Ifi30 - interferon gamma inducible protein 30  H2-Eb1 - histocompatibility 2, class ii antigen e beta  Cd74 - cd74 antigen (invariant polypeptide of major histocompatibility complex, class ii antigen-associated)  H2-Oa - histocompatibility 2, o region alpha locus  Kdm5d - lysine (k)-specific demethylase 5d  H2-M3 - histocompatibility 2, m region locus 3  H2-DMa - histocompatibility 2, class ii, locus dma  Ctss - cathepsin s |
|  |  |  |  |  | Mgll - monoglyceride lipase  Cebpa - ccaat/enhancer binding protein (c/ebp), alpha Trem2 - triggering receptor expressed on myeloid cells 2 Cst7 - cystatin f (leukocystatin)  Fgl2 - fibrinogen-like protein 2  Il1rl1 - interleukin 1 receptor-like 1  Fpr2 - formyl peptide receptor 2  Cd74 - cd74 antigen (invariant polypeptide of major histocompatibility complex, class ii antigen-associated)  Cd9 - cd9 antigen  Csf1r - colony stimulating factor 1 receptor  Socs3 - suppressor of cytokine signaling 3  Clec7a - c-type lectin domain family 7, member a |
| GO:0032101 | regulation of response to external stimulus | 7.94E-13 | 1.02E-9 | 3.98 (15721,772,184,36) | Mmp12 - matrix metallopeptidase 12  Ccl3 - chemokine (c-c motif) ligand 3  Ccl4 - chemokine (c-c motif) ligand 4  Irg1 - immunoresponsive gene 1  P2rx4 - purinergic receptor p2x, ligand-gated ion channel 4 Ccl5 - chemokine (c-c motif) ligand 5  Csf1 - colony stimulating factor 1 (macrophage)  Ctss - cathepsin s  Ccl2 - chemokine (c-c motif) ligand 2  Cd200r3 - cd200 receptor 3  Ccr5 - chemokine (c-c motif) receptor 5  Tnfrsf1b - tumor necrosis factor receptor superfamily, member 1b  Casp1 - caspase 1  Sirpa - signal-regulatory protein alpha  Alox15 - arachidonate 15-lipoxygenase  Mmp8 - matrix metallopeptidase 8  Mpp1 - membrane protein, palmitoylated  Tgm2 - transglutaminase 2, c polypeptide  Cd34 - cd34 antigen  Padi2 - peptidyl arginine deiminase, type ii  Pld3 - phospholipase d family, member 3  Nenf - neuron derived neurotrophic factor  Pld4 - phospholipase d family, member 4  Tnfaip3 - tumor necrosis factor, alpha-induced protein 3 |
| GO:0051239 | regulation of multicellular organismal process | 9.01E-13 | 1.06E-9 | 2.17 (15721,3075,184,78) | Mgll - monoglyceride lipase  Dsc2 - desmocollin 2  Trf - transferrin  Decr1 - 2,4-dienoyl coa reductase 1, mitochondrial  Akap11 - a kinase (prka) anchor protein 11  Prkcb - protein kinase c, beta  Cd74 - cd74 antigen (invariant polypeptide of major histocompatibility complex, class ii antigen-associated)  Cd9 - cd9 antigen  H2-Oa - histocompatibility 2, o region alpha locus  Mmp2 - matrix metallopeptidase 2  Mmp13 - matrix metallopeptidase 13  Dab2 - disabled 2, mitogen-responsive phosphoprotein Mmp12 - matrix metallopeptidase 12  H2-M3 - histocompatibility 2, m region locus 3  Gch1 - gtp cyclohydrolase 1  P2rx4 - purinergic receptor p2x, ligand-gated ion channel 4 H2-DMa - histocompatibility 2, class ii, locus dma  Gpnmb - glycoprotein (transmembrane) nmb  Ccl2 - chemokine (c-c motif) ligand 2  Itgax - integrin alpha x  Pde4b - phosphodiesterase 4b, camp specific  Tnfrsf1b - tumor necrosis factor receptor superfamily, member 1b  Clec4n - c-type lectin domain family 4, member n  Tnfrsf9 - tumor necrosis factor receptor superfamily, member 9 F  abp5 - fatty acid binding protein 5, epidermal  Slc11a1 - solute carrier family 11 (proton-coupled divalent metal ion transporters), member 1  Mmp8 - matrix metallopeptidase 8  Ifi205 - interferon activated gene 205  Cd34 - cd34 antigen  Plxnc1 - plexin c1  Acvr1 - activin a receptor, type 1  Pld4 - phospholipase d family, member 4  Tnfaip3 - tumor necrosis factor, alpha-induced protein 3 Zhx2 - zinc fingers and homeoboxes 2  Rnf128 - ring finger protein 128  AF251705 - cdna sequence af251705  Cebpa - ccaat/enhancer binding protein (c/ebp), alpha Icam1 - intercellular adhesion molecule 1  Rnf157 - ring finger protein 157  Inpp4b - inositol polyphosphate-4-phosphatase, type ii Trem2 - triggering receptor expressed on myeloid cells 2  Fgr - gardner-rasheed feline sarcoma viral (fgr) oncogene homolog  Cd274 - cd274 antigen  Cst7 - cystatin f (leukocystatin)  Fgl2 - fibrinogen-like protein 2  Il1rl1 - interleukin 1 receptor-like 1  Csf1r - colony stimulating factor 1 receptor  Csf2 - colony stimulating factor 2 (granulocyte-macrophage)  Fam20c - family with sequence similarity 20, member c  Evl - ena-vasodilator stimulated phosphoprotein  Clec7a - c-type lectin domain family 7, member a  Ccl3 - chemokine (c-c motif) ligand 3  Ccl4 - chemokine (c-c motif) ligand 4  Irg1 - immunoresponsive gene 1 |
|  |  |  |  |  | Bmi1 - bmi1 polycomb ring finger oncogene  Ccl5 - chemokine (c-c motif) ligand 5  Csf1 - colony stimulating factor 1 (macrophage)  Procr - protein c receptor, endothelial  Ctss - cathepsin s  Ccr5 - chemokine (c-c motif) receptor 5  H2-D1 - histocompatibility 2, d region locus 1  Lgals3 - lectin, galactose binding, soluble 3  Il7r - interleukin 7 receptor  Ndrg2 - n-myc downstream regulated gene 2  H2-Aa - histocompatibility 2, class ii antigen a, alpha  Sirpa - signal-regulatory protein alpha  Casp1 - caspase 1  Epx - eosinophil peroxidase  Thy1 - thymus cell antigen 1, theta  H2-K1 - histocompatibility 2, k1, k region  Zeb2 - zinc finger e-box binding homeobox 2  Inhba - inhibin beta-a  Il1rn - interleukin 1 receptor antagonist  Pld3 - phospholipase d family, member 3  Runx1 - runt related transcription factor 1  Egr3 - early growth response 3  Prg2 - proteoglycan 2, bone marrow  Egr2 - early growth response 2 |
| GO:0019884 | antigen processing and presentation of exogenous antigen | 2.77E-12 | 3.03E-9 | 25.89 (15721,33,184,10) | Cd74 - cd74 antigen (invariant polypeptide of major histocompatibility complex, class ii antigen-associated)  H2-Oa - histocompatibility 2, o region alpha locus  H2-Ab1 - histocompatibility 2, class ii antigen a, beta 1  H2-Aa - histocompatibility 2, class ii antigen a, alpha  H2-M3 - histocompatibility 2, m region locus 3  H2-DMa - histocompatibility 2, class ii, locus dma  H2-K1 - histocompatibility 2, k1, k region  H2-Eb1 - histocompatibility 2, class ii antigen e beta  Ctss - cathepsin s  Ifi30 - interferon gamma inducible protein 30 |
| GO:0001819 | positive regulation of cytokine production | 3.39E-12 | 3.47E-9 | 5.30 (15721,419,184,26) | AF251705 - cdna sequence af251705  Trem2 - triggering receptor expressed on myeloid cells 2 Cd274 - cd274 antigen  Fgr - gardner-rasheed feline sarcoma viral (fgr) oncogene homolog  Il1rl1 - interleukin 1 receptor-like 1  Cd74 - cd74 antigen (invariant polypeptide of major histocompatibility complex, class ii antigen-associated)  Csf1r - colony stimulating factor 1 receptor  Csf2 - colony stimulating factor 2 (granulocyte-macrophage)  Clec7a - c-type lectin domain family 7, member a  Mmp12 - matrix metallopeptidase 12  Ccl3 - chemokine (c-c motif) ligand 3  H2-M3 - histocompatibility 2, m region locus 3  Ccl4 - chemokine (c-c motif) ligand 4  Ccl5 - chemokine (c-c motif) ligand 5  Ccl2 - chemokine (c-c motif) ligand 2  Pde4b - phosphodiesterase 4b, camp specific  Ccr5 - chemokine (c-c motif) receptor 5  Clec4n - c-type lectin domain family 4, member n  Casp1 - caspase 1  Epx - eosinophil peroxidase  Slc11a1 - solute carrier family 11 (proton-coupled divalent metal ion transporters), member 1  Mmp8 - matrix metallopeptidase 8  Ifi205 - interferon activated gene 205  Cd34 - cd34 antigen  Runx1 - runt related transcription factor 1  Prg2 - proteoglycan 2, bone marrow |
| GO:0019886 | antigen processing and presentation of exogenous peptide antigen via MHC class II | 3.59E-12 | 3.45E-9 | 42.72 (15721,16,184,8) | Cd74 - cd74 antigen (invariant polypeptide of major histocompatibility complex, class ii antigen-associated)  H2-Oa - histocompatibility 2, o region alpha locus  H2-Ab1 - histocompatibility 2, class ii antigen a, beta 1  H2-Aa - histocompatibility 2, class ii antigen a, alpha  H2-DMa - histocompatibility 2, class ii, locus dma  Ctss - cathepsin s  H2-Eb1 - histocompatibility 2, class ii antigen e beta  Ifi30 - interferon gamma inducible protein 30 |
| GO:0043207 | response to external biotic stimulus | 5.39E-12 | 4.87E-9 | 3.83 (15721,781,184,35) | Mrc1 - mannose receptor, c type 1  Ikbke - inhibitor of kappab kinase epsilon  Fam46a - family with sequence similarity 46, member a  Trf - transferrin  Trem2 - triggering receptor expressed on myeloid cells 2  Fgr - gardner-rasheed feline sarcoma viral (fgr) oncogene homolog  Cd274 - cd274 antigen  Fpr2 - formyl peptide receptor 2  Hck - hemopoietic cell kinase  Cxcl2 - chemokine (c-x-c motif) ligand 2  Clec7a - c-type lectin domain family 7, member a  Mmp12 - matrix metallopeptidase 12  H2-M3 - histocompatibility 2, m region locus 3  Gch1 - gtp cyclohydrolase 1  Irg1 - immunoresponsive gene 1  Saa3 - serum amyloid a 3  Ccl2 - chemokine (c-c motif) ligand 2  Itgax - integrin alpha x  Pde4b - phosphodiesterase 4b, camp specific  Ccl17 - chemokine (c-c motif) ligand 17  Lgals3 - lectin, galactose binding, soluble 3  Il7r - interleukin 7 receptor  Tnfrsf1b - tumor necrosis factor receptor superfamily, member 1b  Clec4n - c-type lectin domain family 4, member n  Casp1 - caspase 1  Sirpa - signal-regulatory protein alpha  Epx - eosinophil peroxidase  Wfdc17 - wap four-disulfide core domain 17  Slc11a1 - solute carrier family 11 (proton-coupled divalent metal ion transporters), member 1  H2-K1 - histocompatibility 2, k1, k region  Ifi205 - interferon activated gene 205  Polr3k - polymerase (rna) iii (dna directed) polypeptide k Pygl - liver glycogen phosphorylase  Tnfaip3 - tumor necrosis factor, alpha-induced protein 3  Prg2 - proteoglycan 2, bone marrow |
| GO:0006952 | defense response | 6.88E-12 | 5.87E-9 | 3.45 (15721,967,184,39) | Trf - transferrin  Icam1 - intercellular adhesion molecule 1  Trem2 - triggering receptor expressed on myeloid cells 2  Fgr - gardner-rasheed feline sarcoma viral (fgr) oncogene homolog  Fpr1 - formyl peptide receptor 1  Fpr2 - formyl peptide receptor 2  Hck - hemopoietic cell kinase  Cd74 - cd74 antigen (invariant polypeptide of major histocompatibility complex, class ii antigen-associated)  Slc15a3 - solute carrier family 15, member 3  Cxcl2 - chemokine (c-x-c motif) ligand 2  Csf1r - colony stimulating factor 1 receptor  Ccl3 - chemokine (c-c motif) ligand 3  H2-M3 - histocompatibility 2, m region locus 3  Ccl4 - chemokine (c-c motif) ligand 4  Irg1 - immunoresponsive gene 1  Saa3 - serum amyloid a 3  Csf1 - colony stimulating factor 1 (macrophage)  Ccl5 - chemokine (c-c motif) ligand 5  Ear2 - eosinophil-associated, ribonuclease a family, member 2  Ccl2 - chemokine (c-c motif) ligand 2  Itgax - integrin alpha x  Lgals3 - lectin, galactose binding, soluble 3  Ccl17 - chemokine (c-c motif) ligand 17  Ccr5 - chemokine (c-c motif) receptor 5  Il7r - interleukin 7 receptor  Tnfrsf1b - tumor necrosis factor receptor superfamily, member 1b  Clec4n - c-type lectin domain family 4, member n  Epx - eosinophil peroxidase  Wfdc17 - wap four-disulfide core domain 17  Slc11a1 - solute carrier family 11 (proton-coupled divalent metal ion transporters), member 1  H2-K1 - histocompatibility 2, k1, k region  Il1rn - interleukin 1 receptor antagonist  Polr3k - polymerase (rna) iii (dna directed) polypeptide k Acvr1 - activin a receptor, type 1  Ccrl2 - chemokine (c-c motif) receptor-like 2  Pld3 - phospholipase d family, member 3  Pld4 - phospholipase d family, member 4  Tnfaip3 - tumor necrosis factor, alpha-induced protein 3  Prg2 - proteoglycan 2, bone marrow |
| GO:0002504 | antigen processing and presentation of peptide or polysaccharide antigen via MHC class II | 1.2E-11 | 9.67E-9 | 37.97 (15721,18,184,8) | Cd74 - cd74 antigen (invariant polypeptide of major histocompatibility complex, class ii antigen-associated)  H2-Oa - histocompatibility 2, o region alpha locus  H2-Ab1 - histocompatibility 2, class ii antigen a, beta 1  H2-Aa - histocompatibility 2, class ii antigen a, alpha  H2-DMa - histocompatibility 2, class ii, locus dma  Ctss - cathepsin s  H2-Eb1 - histocompatibility 2, class ii antigen e beta  Ifi30 - interferon gamma inducible protein 30 |
| GO:0002495 | antigen processing and presentation of peptide antigen via MHC class II | 1.2E-11 | 9.19E-9 | 37.97 (15721,18,184,8) | Cd74 - cd74 antigen (invariant polypeptide of major histocompatibility complex, class ii antigen-associated)  H2-Oa - histocompatibility 2, o region alpha locus  H2-Ab1 - histocompatibility 2, class ii antigen a, beta 1  H2-Aa - histocompatibility 2, class ii antigen a, alpha  H2-DMa - histocompatibility 2, class ii, locus dma  Ctss - cathepsin s  H2-Eb1 - histocompatibility 2, class ii antigen e beta  Ifi30 - interferon gamma inducible protein 30 |
| GO:0009607 | response to biotic stimulus | 1.26E-11 | 9.23E-9 | 3.71 (15721,805,184,35) | Mrc1 - mannose receptor, c type 1  Ikbke - inhibitor of kappab kinase epsilon  Fam46a - family with sequence similarity 46, member a  Trf - transferrin  Trem2 - triggering receptor expressed on myeloid cells 2  Fgr - gardner-rasheed feline sarcoma viral (fgr) oncogene homolog  Cd274 - cd274 antigen  Fpr2 - formyl peptide receptor 2  Hck - hemopoietic cell kinase  Cxcl2 - chemokine (c-x-c motif) ligand 2  Clec7a - c-type lectin domain family 7, member a  Mmp12 - matrix metallopeptidase 12  H2-M3 - histocompatibility 2, m region locus 3  Gch1 - gtp cyclohydrolase 1  Irg1 - immunoresponsive gene 1  Saa3 - serum amyloid a 3  Ccl2 - chemokine (c-c motif) ligand 2  Itgax - integrin alpha x  Pde4b - phosphodiesterase 4b, camp specific  Ccl17 - chemokine (c-c motif) ligand 17  Lgals3 - lectin, galactose binding, soluble 3  Il7r - interleukin 7 receptor  Tnfrsf1b - tumor necrosis factor receptor superfamily, member 1b  Clec4n - c-type lectin domain family 4, member n  Casp1 - caspase 1  Sirpa - signal-regulatory protein alpha  Epx - eosinophil peroxidase  Wfdc17 - wap four-disulfide core domain 17  Slc11a1 - solute carrier family 11 (proton-coupled divalent metal ion transporters), member 1  H2-K1 - histocompatibility 2, k1, k region  Ifi205 - interferon activated gene 205  Polr3k - polymerase (rna) iii (dna directed) polypeptide k Pygl - liver glycogen phosphorylase  Tnfaip3 - tumor necrosis factor, alpha-induced protein 3 Prg2 - proteoglycan 2, bone marrow |
| GO:0006954 | inflammatory response | 2.8E-11 | 1.95E-8 | 5.27 (15721,389,184,24) | Ccl2 - chemokine (c-c motif) ligand 2  Ccr5 - chemokine (c-c motif) receptor 5  Ccl17 - chemokine (c-c motif) ligand 17  Tnfrsf1b - tumor necrosis factor receptor superfamily, member 1b  Icam1 - intercellular adhesion molecule 1  Trem2 - triggering receptor expressed on myeloid cells 2  Slc11a1 - solute carrier family 11 (proton-coupled divalent metal ion transporters), member 1  Fpr1 - formyl peptide receptor 1  Hck - hemopoietic cell kinase  Fpr2 - formyl peptide receptor 2  Cxcl2 - chemokine (c-x-c motif) ligand 2  Csf1r - colony stimulating factor 1 receptor  Il1rn - interleukin 1 receptor antagonist  Acvr1 - activin a receptor, type 1  Ccl3 - chemokine (c-c motif) ligand 3  Ccl4 - chemokine (c-c motif) ligand 4  Ccrl2 - chemokine (c-c motif) receptor-like 2  Irg1 - immunoresponsive gene 1  Pld3 - phospholipase d family, member 3  Saa3 - serum amyloid a 3  Pld4 - phospholipase d family, member 4  Csf1 - colony stimulating factor 1 (macrophage)  Ccl5 - chemokine (c-c motif) ligand 5  Tnfaip3 - tumor necrosis factor, alpha-induced protein 3 |
| GO:0050727 | regulation of inflammatory response | 3.32E-11 | 2.22E-8 | 5.49 (15721,358,184,23) | Mgll - monoglyceride lipase  Ccr5 - chemokine (c-c motif) receptor 5  Cd200r3 - cd200 receptor 3  Tnfrsf1b - tumor necrosis factor receptor superfamily, member 1b  Cebpa - ccaat/enhancer binding protein (c/ebp), alpha  Casp1 - caspase 1  Sirpa - signal-regulatory protein alpha  Alox15 - arachidonate 15-lipoxygenase  Trem2 - triggering receptor expressed on myeloid cells 2 Cst7 - cystatin f (leukocystatin)  Mmp8 - matrix metallopeptidase 8  Il1rl1 - interleukin 1 receptor-like 1  Fpr2 - formyl peptide receptor 2  Tgm2 - transglutaminase 2, c polypeptide  Socs3 - suppressor of cytokine signaling 3  Clec7a - c-type lectin domain family 7, member a  Ccl3 - chemokine (c-c motif) ligand 3  Irg1 - immunoresponsive gene 1  Pld3 - phospholipase d family, member 3  Pld4 - phospholipase d family, member 4  Ccl5 - chemokine (c-c motif) ligand 5  Tnfaip3 - tumor necrosis factor, alpha-induced protein 3  Ctss - cathepsin s |
| GO:0051240 | positive regulation of multicellular organismal process | 6.09E-11 | 3.89E-8 | 2.52 (15721,1832,184,54) | Trf - transferrin  Decr1 - 2,4-dienoyl coa reductase 1, mitochondrial  Prkcb - protein kinase c, beta  Cd74 - cd74 antigen (invariant polypeptide of major histocompatibility complex, class ii antigen-associated)  Mmp13 - matrix metallopeptidase 13  Dab2 - disabled 2, mitogen-responsive phosphoprotein Mmp12 - matrix metallopeptidase 12  H2-M3 - histocompatibility 2, m region locus 3  Gch1 - gtp cyclohydrolase 1  P2rx4 - purinergic receptor p2x, ligand-gated ion channel 4 H2-DMa - histocompatibility 2, class ii, locus dma  Ccl2 - chemokine (c-c motif) ligand 2  Itgax - integrin alpha x  Pde4b - phosphodiesterase 4b, camp specific  Tnfrsf1b - tumor necrosis factor receptor superfamily, member 1b  Clec4n - c-type lectin domain family 4, member n  Fabp5 - fatty acid binding protein 5, epidermal  Slc11a1 - solute carrier family 11 (proton-coupled divalent metal ion transporters), member 1  Mmp8 - matrix metallopeptidase 8  Ifi205 - interferon activated gene 205  Cd34 - cd34 antigen  Plxnc1 - plexin c1  Acvr1 - activin a receptor, type 1  Tnfaip3 - tumor necrosis factor, alpha-induced protein 3 AF251705 - cdna sequence af251705  Cebpa - ccaat/enhancer binding protein (c/ebp), alpha Icam1 - intercellular adhesion molecule 1  Rnf157 - ring finger protein 157  Trem2 - triggering receptor expressed on myeloid cells 2 Cd274 - cd274 antigen  Cst7 - cystatin f (leukocystatin)  Fgr - gardner-rasheed feline sarcoma viral (fgr) oncogene homolog  Il1rl1 - interleukin 1 receptor-like 1  Csf1r - colony stimulating factor 1 receptor  Csf2 - colony stimulating factor 2 (granulocyte-macrophage)  Fam20c - family with sequence similarity 20, member c Clec7a - c-type lectin domain family 7, member a  Ccl3 - chemokine (c-c motif) ligand 3  Ccl4 - chemokine (c-c motif) ligand 4  Bmi1 - bmi1 polycomb ring finger oncogene  Csf1 - colony stimulating factor 1 (macrophage)  Ccl5 - chemokine (c-c motif) ligand 5  Ccr5 - chemokine (c-c motif) receptor 5  Lgals3 - lectin, galactose binding, soluble 3  Il7r - interleukin 7 receptor  H2-Aa - histocompatibility 2, class ii antigen a, alpha  Casp1 - caspase 1  Epx - eosinophil peroxidase  Zeb2 - zinc finger e-box binding homeobox 2  Inhba - inhibin beta-a  Runx1 - runt related transcription factor 1  Egr3 - early growth response 3  Egr2 - early growth response 2  Prg2 - proteoglycan 2, bone marrow |
| GO:0002694 | regulation of leukocyte activation | 6.66E-11 | 4.09E-8 | 4.46 (15721,517,184,27) | Cebpa - ccaat/enhancer binding protein (c/ebp), alpha Trem2 - triggering receptor expressed on myeloid cells 2 Cd274 - cd274 antigen  Cst7 - cystatin f (leukocystatin)  Fgr - gardner-rasheed feline sarcoma viral (fgr) oncogene homolog  Fgl2 - fibrinogen-like protein 2  Il1rl1 - interleukin 1 receptor-like 1  Cd74 - cd74 antigen (invariant polypeptide of major histocompatibility complex, class ii antigen-associated)  H2-Oa - histocompatibility 2, o region alpha locus  Clec7a - c-type lectin domain family 7, member a  H2-M3 - histocompatibility 2, m region locus 3  Bmi1 - bmi1 polycomb ring finger oncogene  H2-DMa - histocompatibility 2, class ii, locus dma  Gpnmb - glycoprotein (transmembrane) nmb  Ccl5 - chemokine (c-c motif) ligand 5  Ccl2 - chemokine (c-c motif) ligand 2  Lgals3 - lectin, galactose binding, soluble 3  Il7r - interleukin 7 receptor  H2-Ab1 - histocompatibility 2, class ii antigen a, beta 1  Tnfrsf1b - tumor necrosis factor receptor superfamily, member 1b  H2-Aa - histocompatibility 2, class ii antigen a, alpha  Sirpa - signal-regulatory protein alpha  Thy1 - thymus cell antigen 1, theta  Mmp8 - matrix metallopeptidase 8  Runx1 - runt related transcription factor 1  Egr3 - early growth response 3  Tnfaip3 - tumor necrosis factor, alpha-induced protein 3 |
| GO:0050865 | regulation of cell activation | 7.5E-11 | 4.43E-8 | 4.28 (15721,559,184,28) | Cebpa - ccaat/enhancer binding protein (c/ebp), alpha Trem2 - triggering receptor expressed on myeloid cells 2 Cd274 - cd274 antigen  Cst7 - cystatin f (leukocystatin)  Fgr - gardner-rasheed feline sarcoma viral (fgr) oncogene homolog  Fgl2 - fibrinogen-like protein 2  Il1rl1 - interleukin 1 receptor-like 1  Cd74 - cd74 antigen (invariant polypeptide of major histocompatibility complex, class ii antigen-associated)  H2-Oa - histocompatibility 2, o region alpha locus  Cd9 - cd9 antigen  Clec7a - c-type lectin domain family 7, member a  H2-M3 - histocompatibility 2, m region locus 3  Bmi1 - bmi1 polycomb ring finger oncogene  H2-DMa - histocompatibility 2, class ii, locus dma  Gpnmb - glycoprotein (transmembrane) nmb  Ccl5 - chemokine (c-c motif) ligand 5  Ccl2 - chemokine (c-c motif) ligand 2  Lgals3 - lectin, galactose binding, soluble 3  Il7r - interleukin 7 receptor  H2-Ab1 - histocompatibility 2, class ii antigen a, beta 1  Tnfrsf1b - tumor necrosis factor receptor superfamily, member 1b  H2-Aa - histocompatibility 2, class ii antigen a, alpha  Sirpa - signal-regulatory protein alpha  Thy1 - thymus cell antigen 1, theta  Mmp8 - matrix metallopeptidase 8  Runx1 - runt related transcription factor 1  Egr3 - early growth response 3  Tnfaip3 - tumor necrosis factor, alpha-induced protein 3 |
| GO:0071345 | cellular response to cytokine stimulus | 1.18E-10 | 6.7E-8 | 4.92 (15721,417,184,24) | Ccl2 - chemokine (c-c motif) ligand 2  Ccl17 - chemokine (c-c motif) ligand 17  Mrc1 - mannose receptor, c type 1  H2-Ab1 - histocompatibility 2, class ii antigen a, beta 1 Cebpa - ccaat/enhancer binding protein (c/ebp), alpha Ifi202b - interferon activated gene 202b  Sirpa - signal-regulatory protein alpha  Icam1 - intercellular adhesion molecule 1  Casp1 - caspase 1  Alox15 - arachidonate 15-lipoxygenase  Ifi205 - interferon activated gene 205  Csf1r - colony stimulating factor 1 receptor  Csf2ra - colony stimulating factor 2 receptor, alpha, low-affinity (granulocyte-macrophage)  Padi2 - peptidyl arginine deiminase, type ii  Ifi203 - interferon activated gene 203  Csf2 - colony stimulating factor 2 (granulocyte-macrophage)  Socs3 - suppressor of cytokine signaling 3  Snx10 - sorting nexin 10  Evl - ena-vasodilator stimulated phosphoprotein  Ccl3 - chemokine (c-c motif) ligand 3  Ccl4 - chemokine (c-c motif) ligand 4  Irg1 - immunoresponsive gene 1  Saa3 - serum amyloid a 3  Ccl5 - chemokine (c-c motif) ligand 5 |

| GO:0048583 | regulation of response to stimulus | 1.55E-10 | 8.49E-8 | 1.94 (15721,3532,184,80) | Mgll - monoglyceride lipase  Trf - transferrin  Fpr1 - formyl peptide receptor 1  Fpr2 - formyl peptide receptor 2  Prkcb - protein kinase c, beta  Cd74 - cd74 antigen (invariant polypeptide of major histocompatibility complex, class ii antigen-associated)  Cd9 - cd9 antigen  Mmp2 - matrix metallopeptidase 2  Socs3 - suppressor of cytokine signaling 3  Dab2 - disabled 2, mitogen-responsive phosphoprotein Mmp12 - matrix metallopeptidase 12  H2-M3 - histocompatibility 2, m region locus 3  Gch1 - gtp cyclohydrolase 1  P2rx4 - purinergic receptor p2x, ligand-gated ion channel 4 H2-DMa - histocompatibility 2, class ii, locus dma  Gpnmb - glycoprotein (transmembrane) nmb  Neto2 - neuropilin (nrp) and tolloid (tll)-like 2  Ccl2 - chemokine (c-c motif) ligand 2  Ccl17 - chemokine (c-c motif) ligand 17  Pde4b - phosphodiesterase 4b, camp specific  Tnfrsf1b - tumor necrosis factor receptor superfamily, member 1b  Clec4n - c-type lectin domain family 4, member n  Alox15 - arachidonate 15-lipoxygenase  Fabp5 - fatty acid binding protein 5, epidermal  Slc11a1 - solute carrier family 11 (proton-coupled divalent metal ion transporters), member 1  Mmp8 - matrix metallopeptidase 8  Ifi205 - interferon activated gene 205  Mpp1 - membrane protein, palmitoylated  Cd34 - cd34 antigen  Padi2 - peptidyl arginine deiminase, type ii  Acvr1 - activin a receptor, type 1  Nenf - neuron derived neurotrophic factor  Lpxn - leupaxin  Pld4 - phospholipase d family, member 4  Tnfaip3 - tumor necrosis factor, alpha-induced protein 3 Chst11 - carbohydrate sulfotransferase 11  Ikbke - inhibitor of kappab kinase epsilon  Cebpa - ccaat/enhancer binding protein (c/ebp), alpha  Icam1 - intercellular adhesion molecule 1  Rnf157 - ring finger protein 157  Inpp4b - inositol polyphosphate-4-phosphatase, type ii Trem2 - triggering receptor expressed on myeloid cells 2  Fgr - gardner-rasheed feline sarcoma viral (fgr) oncogene homolog  Cd274 - cd274 antigen  Cst7 - cystatin f (leukocystatin)  Fgl2 - fibrinogen-like protein 2  Il1rl1 - interleukin 1 receptor-like 1  Slc15a3 - solute carrier family 15, member 3  Csf1r - colony stimulating factor 1 receptor  Ifi203 - interferon activated gene 203  Csf2 - colony stimulating factor 2 (granulocyte-macrophage) Avpi1 - arginine vasopressin-induced 1  Fam20c - family with sequence similarity 20, member c Clec7a - c-type lectin domain family 7, member a  Ccl3 - chemokine (c-c motif) ligand 3  Ccl4 - chemokine (c-c motif) ligand 4  Irg1 - immunoresponsive gene 1  Bmi1 - bmi1 polycomb ring finger oncogene  Ccl5 - chemokine (c-c motif) ligand 5  Csf1 - colony stimulating factor 1 (macrophage)  Ctss - cathepsin s  Igfbp7 - insulin-like growth factor binding protein 7  Cd200r3 - cd200 receptor 3  Ccr5 - chemokine (c-c motif) receptor 5  H2-D1 - histocompatibility 2, d region locus 1  Lgals3 - lectin, galactose binding, soluble 3  Il7r - interleukin 7 receptor  Ndrg2 - n-myc downstream regulated gene 2  H2-Ab1 - histocompatibility 2, class ii antigen a, beta 1  Sirpa - signal-regulatory protein alpha  Casp1 - caspase 1 |
| --- | --- | --- | --- | --- | --- |

|  |  |  |  |  | Thy1 - thymus cell antigen 1, theta  H2-K1 - histocompatibility 2, k1, k region  Zeb2 - zinc finger e-box binding homeobox 2  Tgm2 - transglutaminase 2, c polypeptide  Inhba - inhibin beta-a  Kdm5d - lysine (k)-specific demethylase 5d  Il1rn - interleukin 1 receptor antagonist  Pld3 - phospholipase d family, member 3  Runx1 - runt related transcription factor 1 |
| --- | --- | --- | --- | --- | --- |
| GO:0051704 | multi-organism process | 1.63E-10 | 8.61E-8 | 3.31 (15721,930,184,36) | Ikbke - inhibitor of kappab kinase epsilon  Fam46a - family with sequence similarity 46, member a  Trf - transferrin  Icam1 - intercellular adhesion molecule 1  Ctsb - cathepsin b  Trem2 - triggering receptor expressed on myeloid cells 2  Fgr - gardner-rasheed feline sarcoma viral (fgr) oncogene homolog Fpr2 - formyl peptide receptor 2  Hck - hemopoietic cell kinase  Cxcl2 - chemokine (c-x-c motif) ligand 2  Csf1r - colony stimulating factor 1 receptor  Clec7a - c-type lectin domain family 7, member a  Mmp12 - matrix metallopeptidase 12  H2-M3 - histocompatibility 2, m region locus 3  Ccl4 - chemokine (c-c motif) ligand 4  Irg1 - immunoresponsive gene 1  Saa3 - serum amyloid a 3  Dach1 - dachshund 1 (drosophila)  Ccl5 - chemokine (c-c motif) ligand 5  Ccl2 - chemokine (c-c motif) ligand 2  Itgax - integrin alpha x  Ccl17 - chemokine (c-c motif) ligand 17  Ccr5 - chemokine (c-c motif) receptor 5  Lgals3 - lectin, galactose binding, soluble 3  Il7r - interleukin 7 receptor  Clec4n - c-type lectin domain family 4, member n  Casp1 - caspase 1  Epx - eosinophil peroxidase  Wfdc17 - wap four-disulfide core domain 17  Slc11a1 - solute carrier family 11 (proton-coupled divalent metal ion transporters), member 1  H2-K1 - histocompatibility 2, k1, k region  Ifi205 - interferon activated gene 205  Polr3k - polymerase (rna) iii (dna directed) polypeptide k  Mcts1 - malignant t cell amplified sequence 1  Pygl - liver glycogen phosphorylase  Prg2 - proteoglycan 2, bone marrow |
| GO:0022407 | regulation of cell-cell adhesion | 3.18E-10 | 1.62E-7 | 4.90 (15721,401,184,23) | Ccl2 - chemokine (c-c motif) ligand 2  Ccr5 - chemokine (c-c motif) receptor 5  Lgals3 - lectin, galactose binding, soluble 3  Il7r - interleukin 7 receptor  H2-Ab1 - histocompatibility 2, class ii antigen a, beta 1  H2-Aa - histocompatibility 2, class ii antigen a, alpha  Sirpa - signal-regulatory protein alpha  Icam1 - intercellular adhesion molecule 1  Alox15 - arachidonate 15-lipoxygenase  Cd274 - cd274 antigen  Thy1 - thymus cell antigen 1, theta  Fgl2 - fibrinogen-like protein 2  Cd74 - cd74 antigen (invariant polypeptide of major histocompatibility complex, class ii antigen-associated)  Cd9 - cd9 antigen  Il1rn - interleukin 1 receptor antagonist  H2-M3 - histocompatibility 2, m region locus 3  H2-DMa - histocompatibility 2, class ii, locus dma  Bmi1 - bmi1 polycomb ring finger oncogene  Runx1 - runt related transcription factor 1  Gpnmb - glycoprotein (transmembrane) nmb  Ccl5 - chemokine (c-c motif) ligand 5  Tnfaip3 - tumor necrosis factor, alpha-induced protein 3  Egr3 - early growth response 3 |
|  |  |  |  |  | Icam1 - intercellular adhesion molecule 1  Cd274 - cd274 antigen  Fgl2 - fibrinogen-like protein 2  Cd74 - cd74 antigen (invariant polypeptide of major histocompatibility complex, class ii antigen-associated)  Mmp2 - matrix metallopeptidase 2 |

| GO:0030155 | regulation of cell adhesion | 4.88E-10 | 2.41E-7 | 3.70 (15721,692,184,30) | Cd9 - cd9 antigen  Mmp12 - matrix metallopeptidase 12  Dab2 - disabled 2, mitogen-responsive phosphoprotein  H2-M3 - histocompatibility 2, m region locus 3  Bmi1 - bmi1 polycomb ring finger oncogene  H2-DMa - histocompatibility 2, class ii, locus dma  Csf1 - colony stimulating factor 1 (macrophage)  Gpnmb - glycoprotein (transmembrane) nmb  Ccl5 - chemokine (c-c motif) ligand 5  Ccl2 - chemokine (c-c motif) ligand 2  Ccr5 - chemokine (c-c motif) receptor 5  Lgals3 - lectin, galactose binding, soluble 3  Il7r - interleukin 7 receptor  H2-Ab1 - histocompatibility 2, class ii antigen a, beta 1  H2-Aa - histocompatibility 2, class ii antigen a, alpha  Sirpa - signal-regulatory protein alpha  Alox15 - arachidonate 15-lipoxygenase  Thy1 - thymus cell antigen 1, theta  Tgm2 - transglutaminase 2, c polypeptide  Il1rn - interleukin 1 receptor antagonist  Plxnc1 - plexin c1  Lpxn - leupaxin  Runx1 - runt related transcription factor 1  Egr3 - early growth response 3  Tnfaip3 - tumor necrosis factor, alpha-induced protein 3 |
| --- | --- | --- | --- | --- | --- |
| GO:0071310 | cellular response to organic substance | 5.04E-10 | 2.42E-7 | 2.87 (15721,1219,184,41) | Mrc1 - mannose receptor, c type 1  Cebpa - ccaat/enhancer binding protein (c/ebp), alpha  Icam1 - intercellular adhesion molecule 1  Ifi202b - interferon activated gene 202b  Trem2 - triggering receptor expressed on myeloid cells 2  Cd274 - cd274 antigen  Fpr2 - formyl peptide receptor 2  Prkcb - protein kinase c, beta  Cxcl2 - chemokine (c-x-c motif) ligand 2  Mmp2 - matrix metallopeptidase 2  Csf1r - colony stimulating factor 1 receptor  Ifi203 - interferon activated gene 203  Csf2ra - colony stimulating factor 2 receptor, alpha, low-affinity (granulocyte-macrophage)  Csf2 - colony stimulating factor 2 (granulocyte-macrophage)  Socs3 - suppressor of cytokine signaling 3  Evl - ena-vasodilator stimulated phosphoprotein  Snx10 - sorting nexin 10  Dab2 - disabled 2, mitogen-responsive phosphoprotein  Ccl3 - chemokine (c-c motif) ligand 3  Ccl4 - chemokine (c-c motif) ligand 4  Gch1 - gtp cyclohydrolase 1  Irg1 - immunoresponsive gene 1  Saa3 - serum amyloid a 3  P2rx4 - purinergic receptor p2x, ligand-gated ion channel 4  Ccl5 - chemokine (c-c motif) ligand 5  Ccl2 - chemokine (c-c motif) ligand 2  Pde4b - phosphodiesterase 4b, camp specific  Ccl17 - chemokine (c-c motif) ligand 17  Tnfrsf1b - tumor necrosis factor receptor superfamily, member 1b H2-Ab1 - histocompatibility 2, class ii antigen a, beta 1  Casp1 - caspase 1  Sirpa - signal-regulatory protein alpha  Alox15 - arachidonate 15-lipoxygenase  Ifi205 - interferon activated gene 205  Inhba - inhibin beta-a  Padi2 - peptidyl arginine deiminase, type ii  Acvr1 - activin a receptor, type 1  Runx1 - runt related transcription factor 1  Tnfaip3 - tumor necrosis factor, alpha-induced protein 3  Egr3 - early growth response 3  Egr2 - early growth response 2 |
|  |  |  |  |  | Ikbke - inhibitor of kappab kinase epsilon  Trem2 - triggering receptor expressed on myeloid cells 2  Cd274 - cd274 antigen  Fgr - gardner-rasheed feline sarcoma viral (fgr) oncogene homolog Fgl2 - fibrinogen-like protein 2  Fpr1 - formyl peptide receptor 1  Il1rl1 - interleukin 1 receptor-like 1  Fpr2 - formyl peptide receptor 2  Prkcb - protein kinase c, beta  Slc15a3 - solute carrier family 15, member 3  Cd74 - cd74 antigen (invariant polypeptide of major histocompatibility complex, class ii antigen-associated) |

| GO:0050776 | regulation of immune response | 5.4E-10 | 2.51E-7 | 3.69 (15721,695,184,30) | Mmp2 - matrix metallopeptidase 2  Ifi203 - interferon activated gene 203  Mmp12 - matrix metallopeptidase 12  H2-M3 - histocompatibility 2, m region locus 3  Irg1 - immunoresponsive gene 1  H2-DMa - histocompatibility 2, class ii, locus dma  Pde4b - phosphodiesterase 4b, camp specific  H2-D1 - histocompatibility 2, d region locus 1  Lgals3 - lectin, galactose binding, soluble 3  Il7r - interleukin 7 receptor  H2-Ab1 - histocompatibility 2, class ii antigen a, beta 1  Tnfrsf1b - tumor necrosis factor receptor superfamily, member 1b Alox15 - arachidonate 15-lipoxygenase  Thy1 - thymus cell antigen 1, theta  Slc11a1 - solute carrier family 11 (proton-coupled divalent metal ion transporters), member 1  H2-K1 - histocompatibility 2, k1, k region  Ifi205 - interferon activated gene 205  Lpxn - leupaxin  Tnfaip3 - tumor necrosis factor, alpha-induced protein 3 |
| --- | --- | --- | --- | --- | --- |
| GO:0048584 | positive regulation of response to stimulus | 5.72E-10 | 2.58E-7 | 2.34 (15721,2004,184,55) | Trf - transferrin  Fpr1 - formyl peptide receptor 1  Fpr2 - formyl peptide receptor 2  Prkcb - protein kinase c, beta  Cd74 - cd74 antigen (invariant polypeptide of major histocompatibility complex, class ii antigen-associated)  Mmp2 - matrix metallopeptidase 2  Mmp12 - matrix metallopeptidase 12  Dab2 - disabled 2, mitogen-responsive phosphoprotein  H2-M3 - histocompatibility 2, m region locus 3  P2rx4 - purinergic receptor p2x, ligand-gated ion channel 4  H2-DMa - histocompatibility 2, class ii, locus dma  Gpnmb - glycoprotein (transmembrane) nmb  Ccl2 - chemokine (c-c motif) ligand 2  Pde4b - phosphodiesterase 4b, camp specific  Ccl17 - chemokine (c-c motif) ligand 17  Clec4n - c-type lectin domain family 4, member n  Alox15 - arachidonate 15-lipoxygenase  Fabp5 - fatty acid binding protein 5, epidermal  Slc11a1 - solute carrier family 11 (proton-coupled divalent metal ion transporters), member 1  Mmp8 - matrix metallopeptidase 8  Ifi205 - interferon activated gene 205  Acvr1 - activin a receptor, type 1  Nenf - neuron derived neurotrophic factor  Tnfaip3 - tumor necrosis factor, alpha-induced protein 3  Ikbke - inhibitor of kappab kinase epsilon  Cebpa - ccaat/enhancer binding protein (c/ebp), alpha  Icam1 - intercellular adhesion molecule 1  Trem2 - triggering receptor expressed on myeloid cells 2  Cd274 - cd274 antigen  Fgr - gardner-rasheed feline sarcoma viral (fgr) oncogene homolog Il1rl1 - interleukin 1 receptor-like 1  Slc15a3 - solute carrier family 15, member 3  Csf1r - colony stimulating factor 1 receptor  Ifi203 - interferon activated gene 203  Csf2 - colony stimulating factor 2 (granulocyte-macrophage)  Avpi1 - arginine vasopressin-induced 1  Clec7a - c-type lectin domain family 7, member a  Ccl3 - chemokine (c-c motif) ligand 3  Ccl4 - chemokine (c-c motif) ligand 4  Irg1 - immunoresponsive gene 1  Csf1 - colony stimulating factor 1 (macrophage)  Ccl5 - chemokine (c-c motif) ligand 5  Ctss - cathepsin s  Ccr5 - chemokine (c-c motif) receptor 5  H2-D1 - histocompatibility 2, d region locus 1  Il7r - interleukin 7 receptor  H2-Ab1 - histocompatibility 2, class ii antigen a, beta 1  Casp1 - caspase 1  Thy1 - thymus cell antigen 1, theta  H2-K1 - histocompatibility 2, k1, k region  Zeb2 - zinc finger e-box binding homeobox 2  Tgm2 - transglutaminase 2, c polypeptide  Inhba - inhibin beta-a  Il1rn - interleukin 1 receptor antagonist  Runx1 - runt related transcription factor 1 |
|  |  |  |  |  |  |

| GO:0050896 | response to stimulus | 7.02E-10 | 3.08E-7 | 1.78 (15721,4284,184,89) | Dsc2 - desmocollin 2  Crip1 - cysteine-rich protein 1 (intestinal)  Zmat3 - zinc finger matrin type 3  Acot2 - acyl-coa thioesterase 2  Ifi202b - interferon activated gene 202b  Trf - transferrin  Fpr1 - formyl peptide receptor 1  Fpr2 - formyl peptide receptor 2  Prkcb - protein kinase c, beta  Cd74 - cd74 antigen (invariant polypeptide of major histocompatibility complex, class ii antigen-associated)  Mmp2 - matrix metallopeptidase 2  Cd9 - cd9 antigen  Socs3 - suppressor of cytokine signaling 3  Mmp13 - matrix metallopeptidase 13  Snx10 - sorting nexin 10  Junb - jun-b oncogene  Alcam - activated leukocyte cell adhesion molecule  Mmp12 - matrix metallopeptidase 12  Dab2 - disabled 2, mitogen-responsive phosphoprotein  H2-M3 - histocompatibility 2, m region locus 3  Gch1 - gtp cyclohydrolase 1  Saa3 - serum amyloid a 3  P2rx4 - purinergic receptor p2x, ligand-gated ion channel 4  H2-DMa - histocompatibility 2, class ii, locus dma  Ccl2 - chemokine (c-c motif) ligand 2  Itgax - integrin alpha x  Ear2 - eosinophil-associated, ribonuclease a family, member 2 Ccl17 - chemokine (c-c motif) ligand 17  Pde4b - phosphodiesterase 4b, camp specific  Cp - ceruloplasmin  Tnfrsf1b - tumor necrosis factor receptor superfamily, member 1b Clec4n - c-type lectin domain family 4, member n  Alox15 - arachidonate 15-lipoxygenase  Slc11a1 - solute carrier family 11 (proton-coupled divalent metal ion transporters), member 1  Ifi205 - interferon activated gene 205  Padi2 - peptidyl arginine deiminase, type ii  Mcts1 - malignant t cell amplified sequence 1  Polr3k - polymerase (rna) iii (dna directed) polypeptide k  P2ry14 - purinergic receptor p2y, g-protein coupled, 14  Pygl - liver glycogen phosphorylase  Acvr1 - activin a receptor, type 1  Ccrl2 - chemokine (c-c motif) receptor-like 2  Pld4 - phospholipase d family, member 4  Tnfaip3 - tumor necrosis factor, alpha-induced protein 3  Mrc1 - mannose receptor, c type 1  Ikbke - inhibitor of kappab kinase epsilon  Cebpa - ccaat/enhancer binding protein (c/ebp), alpha  Fam46a - family with sequence similarity 46, member a  Icam1 - intercellular adhesion molecule 1  Trem2 - triggering receptor expressed on myeloid cells 2  Cd274 - cd274 antigen  Fgr - gardner-rasheed feline sarcoma viral (fgr) oncogene homolog Hck - hemopoietic cell kinase  Cxcl2 - chemokine (c-x-c motif) ligand 2  Slc15a3 - solute carrier family 15, member 3  Csf1r - colony stimulating factor 1 receptor  Csf2ra - colony stimulating factor 2 receptor, alpha, low-affinity (granulocyte-macrophage)  Ifi203 - interferon activated gene 203  Csf2 - colony stimulating factor 2 (granulocyte-macrophage)  Evl - ena-vasodilator stimulated phosphoprotein  Clec7a - c-type lectin domain family 7, member a  Ccl3 - chemokine (c-c motif) ligand 3  Ccl4 - chemokine (c-c motif) ligand 4  Irg1 - immunoresponsive gene 1  Bmi1 - bmi1 polycomb ring finger oncogene  Ccl5 - chemokine (c-c motif) ligand 5  Csf1 - colony stimulating factor 1 (macrophage)  Ctss - cathepsin s  Lgals3 - lectin, galactose binding, soluble 3  Ccr5 - chemokine (c-c motif) receptor 5  H2-D1 - histocompatibility 2, d region locus 1  Il7r - interleukin 7 receptor  H2-Ab1 - histocompatibility 2, class ii antigen a, beta 1  H2-Aa - histocompatibility 2, class ii antigen a, alpha  Sirpa - signal-regulatory protein alpha  Casp1 - caspase 1 Cpne7 - copine vii  Epx - eosinophil peroxidase  Wfdc17 - wap four-disulfide core domain 17 |
| --- | --- | --- | --- | --- | --- |

|  |  |  |  |  | H2-K1 - histocompatibility 2, k1, k region  H2-Eb1 - histocompatibility 2, class ii antigen e beta  Inhba - inhibin beta-a  Il1rn - interleukin 1 receptor antagonist  Serpinb2 - serine (or cysteine) peptidase inhibitor, clade b, member 2 Pld3 - phospholipase d family, member 3  Runx1 - runt related transcription factor 1  Egr3 - early growth response 3  Prg2 - proteoglycan 2, bone marrow  Egr2 - early growth response 2 |
| --- | --- | --- | --- | --- | --- |
| GO:0010033 | response to organic substance | 8.09E-10 | 3.45E-7 | 2.41 (15721,1846,184,52) | Ifi202b - interferon activated gene 202b  Fpr2 - formyl peptide receptor 2  Prkcb - protein kinase c, beta  Cd74 - cd74 antigen (invariant polypeptide of major histocompatibility complex, class ii antigen-associated)  Cd9 - cd9 antigen  Mmp2 - matrix metallopeptidase 2  Socs3 - suppressor of cytokine signaling 3  Mmp13 - matrix metallopeptidase 13  Snx10 - sorting nexin 10  Dab2 - disabled 2, mitogen-responsive phosphoprotein  H2-M3 - histocompatibility 2, m region locus 3  Gch1 - gtp cyclohydrolase 1  Saa3 - serum amyloid a 3  P2rx4 - purinergic receptor p2x, ligand-gated ion channel 4  Ccl2 - chemokine (c-c motif) ligand 2  Pde4b - phosphodiesterase 4b, camp specific  Ccl17 - chemokine (c-c motif) ligand 17  Tnfrsf1b - tumor necrosis factor receptor superfamily, member 1b Alox15 - arachidonate 15-lipoxygenase  Slc11a1 - solute carrier family 11 (proton-coupled divalent metal ion transporters), member 1  Ifi205 - interferon activated gene 205  Padi2 - peptidyl arginine deiminase, type ii  Acvr1 - activin a receptor, type 1  Tnfaip3 - tumor necrosis factor, alpha-induced protein 3  Mrc1 - mannose receptor, c type 1  Ikbke - inhibitor of kappab kinase epsilon  Cebpa - ccaat/enhancer binding protein (c/ebp), alpha  Icam1 - intercellular adhesion molecule 1  Trem2 - triggering receptor expressed on myeloid cells 2  Cd274 - cd274 antigen  Cxcl2 - chemokine (c-x-c motif) ligand 2  Csf1r - colony stimulating factor 1 receptor  Csf2ra - colony stimulating factor 2 receptor, alpha, low-affinity (granulocyte-macrophage)  Ifi203 - interferon activated gene 203  Csf2 - colony stimulating factor 2 (granulocyte-macrophage)  Evl - ena-vasodilator stimulated phosphoprotein  Clec7a - c-type lectin domain family 7, member a  Ccl3 - chemokine (c-c motif) ligand 3  Ccl4 - chemokine (c-c motif) ligand 4  Irg1 - immunoresponsive gene 1  Ccl5 - chemokine (c-c motif) ligand 5  Ccr5 - chemokine (c-c motif) receptor 5  H2-Ab1 - histocompatibility 2, class ii antigen a, beta 1  H2-Aa - histocompatibility 2, class ii antigen a, alpha  Sirpa - signal-regulatory protein alpha  Casp1 - caspase 1  H2-Eb1 - histocompatibility 2, class ii antigen e beta  Inhba - inhibin beta-a  Il1rn - interleukin 1 receptor antagonist  Runx1 - runt related transcription factor 1  Egr3 - early growth response 3  Egr2 - early growth response 2 |
|  |  |  |  |  | Ikbke - inhibitor of kappab kinase epsilon  Fam46a - family with sequence similarity 46, member a  Trf - transferrin  Trem2 - triggering receptor expressed on myeloid cells 2  Fgr - gardner-rasheed feline sarcoma viral (fgr) oncogene homolog Hck - hemopoietic cell kinase  Fpr2 - formyl peptide receptor 2  Cxcl2 - chemokine (c-x-c motif) ligand 2  Clec7a - c-type lectin domain family 7, member a  Mmp12 - matrix metallopeptidase 12  H2-M3 - histocompatibility 2, m region locus 3  Irg1 - immunoresponsive gene 1  Saa3 - serum amyloid a 3 |

| GO:0051707 | response to other organism | 9.22E-10 | 3.83E-7 | 3.83 (15721,624,184,28) | Ccl2 - chemokine (c-c motif) ligand 2  Itgax - integrin alpha x  Lgals3 - lectin, galactose binding, soluble 3  Ccl17 - chemokine (c-c motif) ligand 17  Il7r - interleukin 7 receptor  Clec4n - c-type lectin domain family 4, member n  Casp1 - caspase 1  Epx - eosinophil peroxidase  Wfdc17 - wap four-disulfide core domain 17  Slc11a1 - solute carrier family 11 (proton-coupled divalent metal ion transporters), member 1  H2-K1 - histocompatibility 2, k1, k region  Ifi205 - interferon activated gene 205  Polr3k - polymerase (rna) iii (dna directed) polypeptide k  Pygl - liver glycogen phosphorylase  Prg2 - proteoglycan 2, bone marrow |
| --- | --- | --- | --- | --- | --- |
| GO:0080134 | regulation of response to stress | 1.15E-9 | 4.66E-7 | 2.75 (15721,1306,184,42) | Mgll - monoglyceride lipase  Ikbke - inhibitor of kappab kinase epsilon  Cebpa - ccaat/enhancer binding protein (c/ebp), alpha  Trf - transferrin  Trem2 - triggering receptor expressed on myeloid cells 2  Fgr - gardner-rasheed feline sarcoma viral (fgr) oncogene homolog Cst7 - cystatin f (leukocystatin)  Fgl2 - fibrinogen-like protein 2  Il1rl1 - interleukin 1 receptor-like 1  Fpr2 - formyl peptide receptor 2  Cd74 - cd74 antigen (invariant polypeptide of major histocompatibility complex, class ii antigen-associated)  Slc15a3 - solute carrier family 15, member 3  Mmp2 - matrix metallopeptidase 2  Cd9 - cd9 antigen  Ifi203 - interferon activated gene 203  Socs3 - suppressor of cytokine signaling 3  Clec7a - c-type lectin domain family 7, member a  Mmp12 - matrix metallopeptidase 12  Ccl3 - chemokine (c-c motif) ligand 3  Dab2 - disabled 2, mitogen-responsive phosphoprotein  H2-M3 - histocompatibility 2, m region locus 3  Gch1 - gtp cyclohydrolase 1 Irg1 - immunoresponsive gene 1  Ccl5 - chemokine (c-c motif) ligand 5  Ctss - cathepsin s  Ccl2 - chemokine (c-c motif) ligand 2  Cd200r3 - cd200 receptor 3  Ccr5 - chemokine (c-c motif) receptor 5  Tnfrsf1b - tumor necrosis factor receptor superfamily, member 1b Casp1 - caspase 1  Sirpa - signal-regulatory protein alpha  Alox15 - arachidonate 15-lipoxygenase  Thy1 - thymus cell antigen 1, theta  Mmp8 - matrix metallopeptidase 8  Zeb2 - zinc finger e-box binding homeobox 2  Ifi205 - interferon activated gene 205  Tgm2 - transglutaminase 2, c polypeptide  Cd34 - cd34 antigen  Il1rn - interleukin 1 receptor antagonist  Pld3 - phospholipase d family, member 3  Pld4 - phospholipase d family, member 4  Tnfaip3 - tumor necrosis factor, alpha-induced protein 3 |
| GO:0002696 | positive regulation of leukocyte activation | 1.25E-9 | 4.93E-7 | 5.31 (15721,322,184,20) | Ccl2 - chemokine (c-c motif) ligand 2  Il7r - interleukin 7 receptor  H2-Ab1 - histocompatibility 2, class ii antigen a, beta 1  Cebpa - ccaat/enhancer binding protein (c/ebp), alpha  H2-Aa - histocompatibility 2, class ii antigen a, alpha  Sirpa - signal-regulatory protein alpha  Trem2 - triggering receptor expressed on myeloid cells 2  Cd274 - cd274 antigen  Fgr - gardner-rasheed feline sarcoma viral (fgr) oncogene homolog Thy1 - thymus cell antigen 1, theta  Mmp8 - matrix metallopeptidase 8  Il1rl1 - interleukin 1 receptor-like 1  Cd74 - cd74 antigen (invariant polypeptide of major histocompatibility complex, class ii antigen-associated)  Clec7a - c-type lectin domain family 7, member a  H2-M3 - histocompatibility 2, m region locus 3  Bmi1 - bmi1 polycomb ring finger oncogene  H2-DMa - histocompatibility 2, class ii, locus dma  Runx1 - runt related transcription factor 1 |

|  |  |  |  |  | Egr3 - early growth response 3  Ccl5 - chemokine (c-c motif) ligand 5 |
| --- | --- | --- | --- | --- | --- |
| GO:0050863 | regulation of T cell activation | 2.16E-9 | 8.29E-7 | 5.45 (15721,298,184,19) | Ccl2 - chemokine (c-c motif) ligand 2  Lgals3 - lectin, galactose binding, soluble 3  Il7r - interleukin 7 receptor  Tnfrsf1b - tumor necrosis factor receptor superfamily, member 1b H2-Ab1 - histocompatibility 2, class ii antigen a, beta 1  H2-Aa - histocompatibility 2, class ii antigen a, alpha  Sirpa - signal-regulatory protein alpha  Cd274 - cd274 antigen  Thy1 - thymus cell antigen 1, theta  Fgl2 - fibrinogen-like protein 2  Cd74 - cd74 antigen (invariant polypeptide of major histocompatibility complex, class ii antigen-associated)  H2-Oa - histocompatibility 2, o region alpha locus  H2-M3 - histocompatibility 2, m region locus 3  Bmi1 - bmi1 polycomb ring finger oncogene  H2-DMa - histocompatibility 2, class ii, locus dma  Runx1 - runt related transcription factor 1  Egr3 - early growth response 3  Gpnmb - glycoprotein (transmembrane) nmb  Ccl5 - chemokine (c-c motif) ligand 5 |
| GO:0070887 | cellular response to chemical stimulus | 2.39E-9 | 8.94E-7 | 2.56 (15721,1502,184,45) | Crip1 - cysteine-rich protein 1 (intestinal)  Mrc1 - mannose receptor, c type 1  Cebpa - ccaat/enhancer binding protein (c/ebp), alpha  Trf - transferrin  Icam1 - intercellular adhesion molecule 1  Ifi202b - interferon activated gene 202b  Trem2 - triggering receptor expressed on myeloid cells 2  Cd274 - cd274 antigen  Fpr2 - formyl peptide receptor 2  Prkcb - protein kinase c, beta  Cxcl2 - chemokine (c-x-c motif) ligand 2  Mmp2 - matrix metallopeptidase 2  Csf1r - colony stimulating factor 1 receptor  Csf2ra - colony stimulating factor 2 receptor, alpha, low-affinity (granulocyte-macrophage)  Ifi203 - interferon activated gene 203  Csf2 - colony stimulating factor 2 (granulocyte-macrophage)  Socs3 - suppressor of cytokine signaling 3  Evl - ena-vasodilator stimulated phosphoprotein  Snx10 - sorting nexin 10  Junb - jun-b oncogene  Dab2 - disabled 2, mitogen-responsive phosphoprotein  Ccl3 - chemokine (c-c motif) ligand 3  Ccl4 - chemokine (c-c motif) ligand 4  Gch1 - gtp cyclohydrolase 1  Irg1 - immunoresponsive gene 1  Saa3 - serum amyloid a 3  P2rx4 - purinergic receptor p2x, ligand-gated ion channel 4  Ccl5 - chemokine (c-c motif) ligand 5  Ccl2 - chemokine (c-c motif) ligand 2  Pde4b - phosphodiesterase 4b, camp specific  Ccl17 - chemokine (c-c motif) ligand 17  Tnfrsf1b - tumor necrosis factor receptor superfamily, member 1b H2-Ab1 - histocompatibility 2, class ii antigen a, beta 1  Casp1 - caspase 1  Sirpa - signal-regulatory protein alpha  Alox15 - arachidonate 15-lipoxygenase  Cpne7 - copine vii  Ifi205 - interferon activated gene 205  Inhba - inhibin beta-a  Padi2 - peptidyl arginine deiminase, type ii  Acvr1 - activin a receptor, type 1  Runx1 - runt related transcription factor 1  Tnfaip3 - tumor necrosis factor, alpha-induced protein 3  Egr3 - early growth response 3  Egr2 - early growth response 2 |
| GO:0071346 | cellular response to interferon-gamma | 2.49E-9 | 9.1E-7 | 11.60 (15721,81,184,11) | Ccl2 - chemokine (c-c motif) ligand 2  Ccl17 - chemokine (c-c motif) ligand 17  Mrc1 - mannose receptor, c type 1  H2-Ab1 - histocompatibility 2, class ii antigen a, beta 1  Casp1 - caspase 1  Sirpa - signal-regulatory protein alpha  Evl - ena-vasodilator stimulated phosphoprotein  Ccl3 - chemokine (c-c motif) ligand 3 |

|  |  |  |  |  | Ccl4 - chemokine (c-c motif) ligand 4  Irg1 - immunoresponsive gene 1  Ccl5 - chemokine (c-c motif) ligand 5 |
| --- | --- | --- | --- | --- | --- |
| GO:0050867 | positive regulation of cell activation | 2.75E-9 | 9.82E-7 | 5.07 (15721,337,184,20) | Ccl2 - chemokine (c-c motif) ligand 2  Il7r - interleukin 7 receptor  H2-Ab1 - histocompatibility 2, class ii antigen a, beta 1  Cebpa - ccaat/enhancer binding protein (c/ebp), alpha  H2-Aa - histocompatibility 2, class ii antigen a, alpha  Sirpa - signal-regulatory protein alpha  Trem2 - triggering receptor expressed on myeloid cells 2  Cd274 - cd274 antigen  Fgr - gardner-rasheed feline sarcoma viral (fgr) oncogene homolog Thy1 - thymus cell antigen 1, theta  Mmp8 - matrix metallopeptidase 8  Il1rl1 - interleukin 1 receptor-like 1  Cd74 - cd74 antigen (invariant polypeptide of major histocompatibility complex, class ii antigen-associated)  Clec7a - c-type lectin domain family 7, member a  H2-M3 - histocompatibility 2, m region locus 3  Bmi1 - bmi1 polycomb ring finger oncogene  H2-DMa - histocompatibility 2, class ii, locus dma  Runx1 - runt related transcription factor 1  Egr3 - early growth response 3  Ccl5 - chemokine (c-c motif) ligand 5 |
| GO:0051249 | regulation of lymphocyte activation | 3.75E-9 | 1.31E-6 | 4.51 (15721,417,184,22) | Ccl2 - chemokine (c-c motif) ligand 2  Lgals3 - lectin, galactose binding, soluble 3  Il7r - interleukin 7 receptor  Tnfrsf1b - tumor necrosis factor receptor superfamily, member 1b H2-Ab1 - histocompatibility 2, class ii antigen a, beta 1  H2-Aa - histocompatibility 2, class ii antigen a, alpha  Sirpa - signal-regulatory protein alpha  Fgr - gardner-rasheed feline sarcoma viral (fgr) oncogene homolog Cd274 - cd274 antigen  Thy1 - thymus cell antigen 1, theta  Fgl2 - fibrinogen-like protein 2  Cd74 - cd74 antigen (invariant polypeptide of major histocompatibility complex, class ii antigen-associated)  H2-Oa - histocompatibility 2, o region alpha locus  Clec7a - c-type lectin domain family 7, member a  H2-M3 - histocompatibility 2, m region locus 3  H2-DMa - histocompatibility 2, class ii, locus dma  Bmi1 - bmi1 polycomb ring finger oncogene  Runx1 - runt related transcription factor 1  Gpnmb - glycoprotein (transmembrane) nmb  Ccl5 - chemokine (c-c motif) ligand 5  Tnfaip3 - tumor necrosis factor, alpha-induced protein 3  Egr3 - early growth response 3 |
| GO:0031349 | positive regulation of defense response | 5.12E-9 | 1.75E-6 | 5.17 (15721,314,184,19) | Ccr5 - chemokine (c-c motif) receptor 5  Ikbke - inhibitor of kappab kinase epsilon  Cebpa - ccaat/enhancer binding protein (c/ebp), alpha  Trem2 - triggering receptor expressed on myeloid cells 2  Mmp8 - matrix metallopeptidase 8  Il1rl1 - interleukin 1 receptor-like 1  Fpr2 - formyl peptide receptor 2  Slc15a3 - solute carrier family 15, member 3  Cd74 - cd74 antigen (invariant polypeptide of major histocompatibility complex, class ii antigen-associated)  Ifi205 - interferon activated gene 205  Mmp2 - matrix metallopeptidase 2  Tgm2 - transglutaminase 2, c polypeptide  Ifi203 - interferon activated gene 203  Clec7a - c-type lectin domain family 7, member a  Mmp12 - matrix metallopeptidase 12  Ccl3 - chemokine (c-c motif) ligand 3  H2-M3 - histocompatibility 2, m region locus 3  Ccl5 - chemokine (c-c motif) ligand 5  Ctss - cathepsin s |
|  |  |  |  |  | Ccl2 - chemokine (c-c motif) ligand 2  Ccr5 - chemokine (c-c motif) receptor 5  Il7r - interleukin 7 receptor  H2-Ab1 - histocompatibility 2, class ii antigen a, beta 1  H2-Aa - histocompatibility 2, class ii antigen a, alpha  Sirpa - signal-regulatory protein alpha  Icam1 - intercellular adhesion molecule 1 |

| GO:0022409 | positive regulation of cell-cell adhesion | 5.7E-9 | 1.9E-6 | 5.83 (15721,249,184,17) | Alox15 - arachidonate 15-lipoxygenase  Cd274 - cd274 antigen  Thy1 - thymus cell antigen 1, theta  Cd74 - cd74 antigen (invariant polypeptide of major histocompatibility complex, class ii antigen-associated)  H2-M3 - histocompatibility 2, m region locus 3  Bmi1 - bmi1 polycomb ring finger oncogene  H2-DMa - histocompatibility 2, class ii, locus dma  Runx1 - runt related transcription factor 1  Egr3 - early growth response 3  Ccl5 - chemokine (c-c motif) ligand 5 |
| --- | --- | --- | --- | --- | --- |
| GO:0042221 | response to chemical | 8.4E-9 | 2.74E-6 | 2.14 (15721,2281,184,57) | Crip1 - cysteine-rich protein 1 (intestinal)  Trf - transferrin  Ifi202b - interferon activated gene 202b  Fpr2 - formyl peptide receptor 2  Prkcb - protein kinase c, beta  Cd74 - cd74 antigen (invariant polypeptide of major histocompatibility complex, class ii antigen-associated)  Mmp2 - matrix metallopeptidase 2  Cd9 - cd9 antigen  Socs3 - suppressor of cytokine signaling 3  Mmp13 - matrix metallopeptidase 13  Snx10 - sorting nexin 10  Junb - jun-b oncogene  Dab2 - disabled 2, mitogen-responsive phosphoprotein  H2-M3 - histocompatibility 2, m region locus 3  Gch1 - gtp cyclohydrolase 1  Saa3 - serum amyloid a 3  P2rx4 - purinergic receptor p2x, ligand-gated ion channel 4  Ccl2 - chemokine (c-c motif) ligand 2  Pde4b - phosphodiesterase 4b, camp specific  Ccl17 - chemokine (c-c motif) ligand 17  Cp - ceruloplasmin  Tnfrsf1b - tumor necrosis factor receptor superfamily, member 1b Alox15 - arachidonate 15-lipoxygenase  Slc11a1 - solute carrier family 11 (proton-coupled divalent metal ion transporters), member 1  Ifi205 - interferon activated gene 205  Padi2 - peptidyl arginine deiminase, type ii  Acvr1 - activin a receptor, type 1  Tnfaip3 - tumor necrosis factor, alpha-induced protein 3  Mrc1 - mannose receptor, c type 1  Ikbke - inhibitor of kappab kinase epsilon  Cebpa - ccaat/enhancer binding protein (c/ebp), alpha  Icam1 - intercellular adhesion molecule 1  Trem2 - triggering receptor expressed on myeloid cells 2  Cd274 - cd274 antigen  Cxcl2 - chemokine (c-x-c motif) ligand 2  Csf1r - colony stimulating factor 1 receptor  Csf2ra - colony stimulating factor 2 receptor, alpha, low-affinity (granulocyte-macrophage)  Ifi203 - interferon activated gene 203  Csf2 - colony stimulating factor 2 (granulocyte-macrophage)  Evl - ena-vasodilator stimulated phosphoprotein  Clec7a - c-type lectin domain family 7, member a  Ccl3 - chemokine (c-c motif) ligand 3  Ccl4 - chemokine (c-c motif) ligand 4  Irg1 - immunoresponsive gene 1  Ccl5 - chemokine (c-c motif) ligand 5  Ccr5 - chemokine (c-c motif) receptor 5  H2-Ab1 - histocompatibility 2, class ii antigen a, beta 1  H2-Aa - histocompatibility 2, class ii antigen a, alpha  Sirpa - signal-regulatory protein alpha  Casp1 - caspase 1  Cpne7 - copine vii  H2-Eb1 - histocompatibility 2, class ii antigen e beta  Inhba - inhibin beta-a  Il1rn - interleukin 1 receptor antagonist  Runx1 - runt related transcription factor 1  Egr3 - early growth response 3  Egr2 - early growth response 2 |
|  |  |  |  |  | Ccl2 - chemokine (c-c motif) ligand 2  Lgals3 - lectin, galactose binding, soluble 3  Il7r - interleukin 7 receptor  H2-Ab1 - histocompatibility 2, class ii antigen a, beta 1  H2-Aa - histocompatibility 2, class ii antigen a, alpha  Icam1 - intercellular adhesion molecule 1 |

| GO:1903037 | regulation of leukocyte cell-cell adhesion | 9.41E-9 | 3.01E-6 | 5.28 (15721,291,184,18) | Sirpa - signal-regulatory protein alpha  Cd274 - cd274 antigen  Fgl2 - fibrinogen-like protein 2  Thy1 - thymus cell antigen 1, theta  Cd74 - cd74 antigen (invariant polypeptide of major histocompatibility complex, class ii antigen-associated)  H2-M3 - histocompatibility 2, m region locus 3  Bmi1 - bmi1 polycomb ring finger oncogene  H2-DMa - histocompatibility 2, class ii, locus dma  Runx1 - runt related transcription factor 1  Egr3 - early growth response 3  Gpnmb - glycoprotein (transmembrane) nmb  Ccl5 - chemokine (c-c motif) ligand 5 |
| --- | --- | --- | --- | --- | --- |
| GO:0002683 | negative regulation of immune system process | 1.56E-8 | 4.89E-6 | 4.17 (15721,451,184,22) | Lgals3 - lectin, galactose binding, soluble 3  Il7r - interleukin 7 receptor  H2-Ab1 - histocompatibility 2, class ii antigen a, beta 1  H2-Aa - histocompatibility 2, class ii antigen a, alpha  Alox15 - arachidonate 15-lipoxygenase  Inpp4b - inositol polyphosphate-4-phosphatase, type ii  Fgr - gardner-rasheed feline sarcoma viral (fgr) oncogene homolog Cst7 - cystatin f (leukocystatin)  Cd274 - cd274 antigen  Thy1 - thymus cell antigen 1, theta  Fgl2 - fibrinogen-like protein 2  Il1rl1 - interleukin 1 receptor-like 1  Cd74 - cd74 antigen (invariant polypeptide of major histocompatibility complex, class ii antigen-associated)  H2-Oa - histocompatibility 2, o region alpha locus  Padi2 - peptidyl arginine deiminase, type ii  Mmp12 - matrix metallopeptidase 12  H2-M3 - histocompatibility 2, m region locus 3  Irg1 - immunoresponsive gene 1  Runx1 - runt related transcription factor 1  Lpxn - leupaxin  Gpnmb - glycoprotein (transmembrane) nmb  Tnfaip3 - tumor necrosis factor, alpha-induced protein 3 |
| GO:0065008 | regulation of biological quality | 1.88E-8 | 5.77E-6 | 1.82 (15721,3466,184,74) | Mgll - monoglyceride lipase  Dsc2 - desmocollin 2  Trf - transferrin  Mtmr9 - myotubularin related protein 9  Slc22a3 - solute carrier family 22 (organic cation transporter), member 3  Decr1 - 2,4-dienoyl coa reductase 1, mitochondrial  Fpr1 - formyl peptide receptor 1  Akap11 - a kinase (prka) anchor protein 11  Eif2s3y - eukaryotic translation initiation factor 2, subunit 3, structural gene y-linked  Fpr2 - formyl peptide receptor 2  Cd74 - cd74 antigen (invariant polypeptide of major histocompatibility complex, class ii antigen-associated)  Prkcb - protein kinase c, beta  Cd9 - cd9 antigen  Mmp2 - matrix metallopeptidase 2  Mmp13 - matrix metallopeptidase 13  Snx10 - sorting nexin 10  Dab2 - disabled 2, mitogen-responsive phosphoprotein  H2-M3 - histocompatibility 2, m region locus 3  Gch1 - gtp cyclohydrolase 1  P2rx4 - purinergic receptor p2x, ligand-gated ion channel 4  Ccl2 - chemokine (c-c motif) ligand 2  Pde4b - phosphodiesterase 4b, camp specific  Cp - ceruloplasmin  Tnfrsf1b - tumor necrosis factor receptor superfamily, member 1b Fmnl2 - formin-like 2  Alox15 - arachidonate 15-lipoxygenase  Fabp5 - fatty acid binding protein 5, epidermal  Slc11a1 - solute carrier family 11 (proton-coupled divalent metal ion transporters), member 1  Mmp8 - matrix metallopeptidase 8  Actr3b - arp3 actin-related protein 3b  Cd34 - cd34 antigen  Plxnc1 - plexin c1  Pygl - liver glycogen phosphorylase  P2ry14 - purinergic receptor p2y, g-protein coupled, 14  Ccrl2 - chemokine (c-c motif) receptor-like 2  Parvb - parvin, beta  Nenf - neuron derived neurotrophic factor  Tnfaip3 - tumor necrosis factor, alpha-induced protein 3  Cyp4f18 - cytochrome p450, family 4, subfamily f, polypeptide 18 |

|  |  |  |  |  | Rnf128 - ring finger protein 128  Ikbke - inhibitor of kappab kinase epsilon  Cebpa - ccaat/enhancer binding protein (c/ebp), alpha  Fam46a - family with sequence similarity 46, member a  Ctsb - cathepsin b  Icam1 - intercellular adhesion molecule 1  Acsl4 - acyl-coa synthetase long-chain family member 4  Inpp4b - inositol polyphosphate-4-phosphatase, type ii  Slc6a12 - solute carrier family 6 (neurotransmitter transporter, betaine/gaba), member 12  Gm2a - gm2 ganglioside activator protein  Trem2 - triggering receptor expressed on myeloid cells 2  Fgr - gardner-rasheed feline sarcoma viral (fgr) oncogene homolog Ifi30 - interferon gamma inducible protein 30  Hck - hemopoietic cell kinase  Cxcl2 - chemokine (c-x-c motif) ligand 2  Csf1r - colony stimulating factor 1 receptor  Evl - ena-vasodilator stimulated phosphoprotein  Ccl3 - chemokine (c-c motif) ligand 3  Ccl4 - chemokine (c-c motif) ligand 4  Bmi1 - bmi1 polycomb ring finger oncogene  Ccl5 - chemokine (c-c motif) ligand 5  Csf1 - colony stimulating factor 1 (macrophage)  Procr - protein c receptor, endothelial  Ctss - cathepsin s  Ccr5 - chemokine (c-c motif) receptor 5  Il7r - interleukin 7 receptor  Casp1 - caspase 1  Sirpa - signal-regulatory protein alpha  Thy1 - thymus cell antigen 1, theta  Atp6v0d2 - atpase, h+ transporting, lysosomal v0 subunit d2  Tgm2 - transglutaminase 2, c polypeptide  Inhba - inhibin beta-a  Il1rn - interleukin 1 receptor antagonist  Runx1 - runt related transcription factor 1  Hdc - histidine decarboxylase |
| --- | --- | --- | --- | --- | --- |
| GO:0002685 | regulation of leukocyte migration | 1.98E-8 | 5.95E-6 | 6.22 (15721,206,184,15) | Ccl2 - chemokine (c-c motif) ligand 2  Lgals3 - lectin, galactose binding, soluble 3  Icam1 - intercellular adhesion molecule 1  Trem2 - triggering receptor expressed on myeloid cells 2  Thy1 - thymus cell antigen 1, theta  Fpr2 - formyl peptide receptor 2  Cd74 - cd74 antigen (invariant polypeptide of major histocompatibility complex, class ii antigen-associated)  Mpp1 - membrane protein, palmitoylated  Csf1r - colony stimulating factor 1 receptor  Cd9 - cd9 antigen  Padi2 - peptidyl arginine deiminase, type ii  Ccl4 - chemokine (c-c motif) ligand 4  P2rx4 - purinergic receptor p2x, ligand-gated ion channel 4  Csf1 - colony stimulating factor 1 (macrophage)  Ccl5 - chemokine (c-c motif) ligand 5 |
| GO:0032103 | positive regulation of response to external stimulus | 2.16E-8 | 6.37E-6 | 5.01 (15721,307,184,18) | Ccl2 - chemokine (c-c motif) ligand 2  Ccr5 - chemokine (c-c motif) receptor 5  Cebpa - ccaat/enhancer binding protein (c/ebp), alpha  Trem2 - triggering receptor expressed on myeloid cells 2  Mmp8 - matrix metallopeptidase 8  Il1rl1 - interleukin 1 receptor-like 1  Fpr2 - formyl peptide receptor 2  Cd74 - cd74 antigen (invariant polypeptide of major histocompatibility complex, class ii antigen-associated)  Csf1r - colony stimulating factor 1 receptor  Tgm2 - transglutaminase 2, c polypeptide  Clec7a - c-type lectin domain family 7, member a  Ccl3 - chemokine (c-c motif) ligand 3  Ccl4 - chemokine (c-c motif) ligand 4  Irg1 - immunoresponsive gene 1  P2rx4 - purinergic receptor p2x, ligand-gated ion channel 4  Csf1 - colony stimulating factor 1 (macrophage)  Ccl5 - chemokine (c-c motif) ligand 5  Ctss - cathepsin s |
|  |  |  |  |  | Ccl2 - chemokine (c-c motif) ligand 2  Il7r - interleukin 7 receptor  H2-Ab1 - histocompatibility 2, class ii antigen a, beta 1  H2-Aa - histocompatibility 2, class ii antigen a, alpha  Sirpa - signal-regulatory protein alpha |

| GO:1903039 | positive regulation of leukocyte cell-cell adhesion | 2.25E-8 | 6.52E-6 | 6.16 (15721,208,184,15) | Icam1 - intercellular adhesion molecule 1  Cd274 - cd274 antigen  Thy1 - thymus cell antigen 1, theta  Cd74 - cd74 antigen (invariant polypeptide of major histocompatibility complex, class ii antigen-associated)  H2-M3 - histocompatibility 2, m region locus 3  Bmi1 - bmi1 polycomb ring finger oncogene  H2-DMa - histocompatibility 2, class ii, locus dma  Runx1 - runt related transcription factor 1  Egr3 - early growth response 3  Ccl5 - chemokine (c-c motif) ligand 5 |
| --- | --- | --- | --- | --- | --- |
| GO:0002577 | regulation of antigen processing and presentation | 3.91E-8 | 1.11E-5 | 28.48 (15721,18,184,6) | Cd74 - cd74 antigen (invariant polypeptide of major histocompatibility complex, class ii antigen-associated)  H2-Oa - histocompatibility 2, o region alpha locus  H2-Ab1 - histocompatibility 2, class ii antigen a, beta 1  Trem2 - triggering receptor expressed on myeloid cells 2  Fgl2 - fibrinogen-like protein 2  Slc11a1 - solute carrier family 11 (proton-coupled divalent metal ion transporters), member 1 |
| GO:0050870 | positive regulation of T cell activation | 3.93E-8 | 1.1E-5 | 6.43 (15721,186,184,14) | Ccl2 - chemokine (c-c motif) ligand 2  Il7r - interleukin 7 receptor  H2-Ab1 - histocompatibility 2, class ii antigen a, beta 1  H2-Aa - histocompatibility 2, class ii antigen a, alpha  Sirpa - signal-regulatory protein alpha  Cd274 - cd274 antigen  Thy1 - thymus cell antigen 1, theta  Cd74 - cd74 antigen (invariant polypeptide of major histocompatibility complex, class ii antigen-associated)  H2-M3 - histocompatibility 2, m region locus 3  H2-DMa - histocompatibility 2, class ii, locus dma  Bmi1 - bmi1 polycomb ring finger oncogene  Runx1 - runt related transcription factor 1  Egr3 - early growth response 3  Ccl5 - chemokine (c-c motif) ligand 5 |
| GO:0009617 | response to bacterium | 4.84E-8 | 1.33E-5 | 4.50 (15721,361,184,19) | Ccl2 - chemokine (c-c motif) ligand 2  Il7r - interleukin 7 receptor  Fam46a - family with sequence similarity 46, member a  Trf - transferrin  Casp1 - caspase 1  Epx - eosinophil peroxidase  Wfdc17 - wap four-disulfide core domain 17  Trem2 - triggering receptor expressed on myeloid cells 2  Fgr - gardner-rasheed feline sarcoma viral (fgr) oncogene homolog Slc11a1 - solute carrier family 11 (proton-coupled divalent metal ion transporters), member 1  H2-K1 - histocompatibility 2, k1, k region  Hck - hemopoietic cell kinase  Fpr2 - formyl peptide receptor 2  Ifi205 - interferon activated gene 205  Pygl - liver glycogen phosphorylase  H2-M3 - histocompatibility 2, m region locus 3  Irg1 - immunoresponsive gene 1  Saa3 - serum amyloid a 3  Prg2 - proteoglycan 2, bone marrow |
| GO:0071674 | mononuclear cell migration | 8.87E-8 | 2.39E-5 | 18.12 (15721,33,184,7) | Ccl2 - chemokine (c-c motif) ligand 2  Lgals3 - lectin, galactose binding, soluble 3  Ccl17 - chemokine (c-c motif) ligand 17  Sirpa - signal-regulatory protein alpha  Ccl3 - chemokine (c-c motif) ligand 3  Ccl4 - chemokine (c-c motif) ligand 4  Ccl5 - chemokine (c-c motif) ligand 5 |
| GO:0097530 | granulocyte migration | 9.61E-8 | 2.54E-5 | 9.49 (15721,90,184,10) | Cxcl2 - chemokine (c-x-c motif) ligand 2  Ccl2 - chemokine (c-c motif) ligand 2  Lgals3 - lectin, galactose binding, soluble 3  Pde4b - phosphodiesterase 4b, camp specific  Ccl17 - chemokine (c-c motif) ligand 17  Sirpa - signal-regulatory protein alpha  Epx - eosinophil peroxidase  Ccl3 - chemokine (c-c motif) ligand 3  Ccl4 - chemokine (c-c motif) ligand 4  Ccl5 - chemokine (c-c motif) ligand 5 |
|  |  |  |  |  |  |

| GO:0045785 | positive regulation of cell adhesion | 9.7E-8 | 2.52E-5 | 4.10 (15721,417,184,20) | Ccl2 - chemokine (c-c motif) ligand 2  Ccr5 - chemokine (c-c motif) receptor 5  Il7r - interleukin 7 receptor  H2-Ab1 - histocompatibility 2, class ii antigen a, beta 1  H2-Aa - histocompatibility 2, class ii antigen a, alpha  Sirpa - signal-regulatory protein alpha  Icam1 - intercellular adhesion molecule 1  Alox15 - arachidonate 15-lipoxygenase  Cd274 - cd274 antigen  Thy1 - thymus cell antigen 1, theta  Cd74 - cd74 antigen (invariant polypeptide of major histocompatibility complex, class ii antigen-associated)  Tgm2 - transglutaminase 2, c polypeptide  Dab2 - disabled 2, mitogen-responsive phosphoprotein  H2-M3 - histocompatibility 2, m region locus 3  H2-DMa - histocompatibility 2, class ii, locus dma  Bmi1 - bmi1 polycomb ring finger oncogene  Runx1 - runt related transcription factor 1  Csf1 - colony stimulating factor 1 (macrophage)  Ccl5 - chemokine (c-c motif) ligand 5  Egr3 - early growth response 3 |
| --- | --- | --- | --- | --- | --- |
| GO:0009605 | response to external stimulus | 9.93E-8 | 2.54E-5 | 2.51 (15721,1296,184,38) | Dsc2 - desmocollin 2  Mrc1 - mannose receptor, c type 1  Ikbke - inhibitor of kappab kinase epsilon  Fam46a - family with sequence similarity 46, member a  Trf - transferrin  Trem2 - triggering receptor expressed on myeloid cells 2  Fgr - gardner-rasheed feline sarcoma viral (fgr) oncogene homolog Cd274 - cd274 antigen  Fpr2 - formyl peptide receptor 2  Hck - hemopoietic cell kinase  Cxcl2 - chemokine (c-x-c motif) ligand 2  Mmp2 - matrix metallopeptidase 2  Clec7a - c-type lectin domain family 7, member a  Mmp12 - matrix metallopeptidase 12  H2-M3 - histocompatibility 2, m region locus 3  Gch1 - gtp cyclohydrolase 1  Irg1 - immunoresponsive gene 1  Saa3 - serum amyloid a 3  P2rx4 - purinergic receptor p2x, ligand-gated ion channel 4  Ccl2 - chemokine (c-c motif) ligand 2  Itgax - integrin alpha x  Pde4b - phosphodiesterase 4b, camp specific  Ccl17 - chemokine (c-c motif) ligand 17  Lgals3 - lectin, galactose binding, soluble 3  Il7r - interleukin 7 receptor  Tnfrsf1b - tumor necrosis factor receptor superfamily, member 1b Clec4n - c-type lectin domain family 4, member n  Casp1 - caspase 1  Sirpa - signal-regulatory protein alpha  Epx - eosinophil peroxidase  Wfdc17 - wap four-disulfide core domain 17  Slc11a1 - solute carrier family 11 (proton-coupled divalent metal ion transporters), member 1  H2-K1 - histocompatibility 2, k1, k region  Ifi205 - interferon activated gene 205  Polr3k - polymerase (rna) iii (dna directed) polypeptide k  Pygl - liver glycogen phosphorylase  Tnfaip3 - tumor necrosis factor, alpha-induced protein 3  Prg2 - proteoglycan 2, bone marrow |
| GO:1902107 | positive regulation of leukocyte differentiation | 1.09E-7 | 2.74E-5 | 6.50 (15721,171,184,13) | Il7r - interleukin 7 receptor  H2-Aa - histocompatibility 2, class ii antigen a, alpha  Trem2 - triggering receptor expressed on myeloid cells 2  Cd74 - cd74 antigen (invariant polypeptide of major histocompatibility complex, class ii antigen-associated)  Csf1r - colony stimulating factor 1 receptor  Ccl3 - chemokine (c-c motif) ligand 3  H2-M3 - histocompatibility 2, m region locus 3  H2-DMa - histocompatibility 2, class ii, locus dma  Bmi1 - bmi1 polycomb ring finger oncogene  Runx1 - runt related transcription factor 1  Ccl5 - chemokine (c-c motif) ligand 5  Csf1 - colony stimulating factor 1 (macrophage)  Egr3 - early growth response 3 |
|  |  |  |  |  | H2-D1 - histocompatibility 2, d region locus 1  Il7r - interleukin 7 receptor |

| GO:0002819 | regulation of adaptive immune response | 1.16E-7 | 2.88E-5 | 6.46 (15721,172,184,13) | Tnfrsf1b - tumor necrosis factor receptor superfamily, member 1b H2-Ab1 - histocompatibility 2, class ii antigen a, beta 1  Alox15 - arachidonate 15-lipoxygenase  Cd274 - cd274 antigen  Slc11a1 - solute carrier family 11 (proton-coupled divalent metal ion transporters), member 1  Il1rl1 - interleukin 1 receptor-like 1  H2-K1 - histocompatibility 2, k1, k region  Cd74 - cd74 antigen (invariant polypeptide of major histocompatibility complex, class ii antigen-associated)  H2-M3 - histocompatibility 2, m region locus 3  H2-DMa - histocompatibility 2, class ii, locus dma  Tnfaip3 - tumor necrosis factor, alpha-induced protein 3 |
| --- | --- | --- | --- | --- | --- |
| GO:0007162 | negative regulation of cell adhesion | 1.51E-7 | 3.68E-5 | 4.97 (15721,275,184,16) | Lgals3 - lectin, galactose binding, soluble 3  H2-Ab1 - histocompatibility 2, class ii antigen a, beta 1  H2-Aa - histocompatibility 2, class ii antigen a, alpha  Cd274 - cd274 antigen  Fgl2 - fibrinogen-like protein 2  Cd74 - cd74 antigen (invariant polypeptide of major histocompatibility complex, class ii antigen-associated)  Mmp2 - matrix metallopeptidase 2  Cd9 - cd9 antigen  Il1rn - interleukin 1 receptor antagonist  Plxnc1 - plexin c1  Mmp12 - matrix metallopeptidase 12  H2-M3 - histocompatibility 2, m region locus 3  Lpxn - leupaxin  Runx1 - runt related transcription factor 1  Gpnmb - glycoprotein (transmembrane) nmb  Tnfaip3 - tumor necrosis factor, alpha-induced protein 3 |
| GO:1903708 | positive regulation of hemopoiesis | 1.59E-7 | 3.81E-5 | 5.75 (15721,208,184,14) | Il7r - interleukin 7 receptor  H2-Aa - histocompatibility 2, class ii antigen a, alpha  Trem2 - triggering receptor expressed on myeloid cells 2  Cd74 - cd74 antigen (invariant polypeptide of major histocompatibility complex, class ii antigen-associated)  Inhba - inhibin beta-a  Csf1r - colony stimulating factor 1 receptor  Ccl3 - chemokine (c-c motif) ligand 3  H2-M3 - histocompatibility 2, m region locus 3  H2-DMa - histocompatibility 2, class ii, locus dma  Bmi1 - bmi1 polycomb ring finger oncogene  Runx1 - runt related transcription factor 1  Egr3 - early growth response 3  Ccl5 - chemokine (c-c motif) ligand 5  Csf1 - colony stimulating factor 1 (macrophage) |
| GO:0002687 | positive regulation of leukocyte migration | 1.69E-7 | 3.98E-5 | 6.93 (15721,148,184,12) | Ccl2 - chemokine (c-c motif) ligand 2  Cd74 - cd74 antigen (invariant polypeptide of major histocompatibility complex, class ii antigen-associated)  Lgals3 - lectin, galactose binding, soluble 3  Csf1r - colony stimulating factor 1 receptor  Icam1 - intercellular adhesion molecule 1  Ccl4 - chemokine (c-c motif) ligand 4  Trem2 - triggering receptor expressed on myeloid cells 2  Thy1 - thymus cell antigen 1, theta  P2rx4 - purinergic receptor p2x, ligand-gated ion channel 4  Ccl5 - chemokine (c-c motif) ligand 5  Csf1 - colony stimulating factor 1 (macrophage)  Fpr2 - formyl peptide receptor 2 |
| GO:0071216 | cellular response to biotic stimulus | 1.74E-7 | 4.04E-5 | 6.24 (15721,178,184,13) | Ccl2 - chemokine (c-c motif) ligand 2  Pde4b - phosphodiesterase 4b, camp specific  Mrc1 - mannose receptor, c type 1  Tnfrsf1b - tumor necrosis factor receptor superfamily, member 1b Casp1 - caspase 1  Sirpa - signal-regulatory protein alpha  Trem2 - triggering receptor expressed on myeloid cells 2  Cd274 - cd274 antigen  Cxcl2 - chemokine (c-x-c motif) ligand 2  Clec7a - c-type lectin domain family 7, member a  Gch1 - gtp cyclohydrolase 1  Irg1 - immunoresponsive gene 1  Tnfaip3 - tumor necrosis factor, alpha-induced protein 3 |
|  |  |  |  |  | Rnf128 - ring finger protein 128 |

| GO:0001818 | negative regulation of cytokine production | 2.28E-7 | 5.23E-5 | 5.17 (15721,248,184,15) | Ndrg2 - n-myc downstream regulated gene 2  Sirpa - signal-regulatory protein alpha  Epx - eosinophil peroxidase  Tnfrsf9 - tumor necrosis factor receptor superfamily, member 9 Trem2 - triggering receptor expressed on myeloid cells 2  Cd274 - cd274 antigen  Slc11a1 - solute carrier family 11 (proton-coupled divalent metal ion transporters), member 1  Il1rl1 - interleukin 1 receptor-like 1  Mmp8 - matrix metallopeptidase 8  Cd34 - cd34 antigen  Irg1 - immunoresponsive gene 1  Tnfaip3 - tumor necrosis factor, alpha-induced protein 3  Gpnmb - glycoprotein (transmembrane) nmb  Prg2 - proteoglycan 2, bone marrow |
| --- | --- | --- | --- | --- | --- |
| GO:0071347 | cellular response to interleukin-1 | 2.3E-7 | 5.2E-5 | 12.43 (15721,55,184,8) | Ccl2 - chemokine (c-c motif) ligand 2  Ccl17 - chemokine (c-c motif) ligand 17  Sirpa - signal-regulatory protein alpha  Ccl3 - chemokine (c-c motif) ligand 3  Ccl4 - chemokine (c-c motif) ligand 4  Irg1 - immunoresponsive gene 1  Saa3 - serum amyloid a 3  Ccl5 - chemokine (c-c motif) ligand 5 |
| GO:0071219 | cellular response to molecule of bacterial origin | 2.6E-7 | 5.79E-5 | 6.66 (15721,154,184,12) | Cxcl2 - chemokine (c-x-c motif) ligand 2  Ccl2 - chemokine (c-c motif) ligand 2  Pde4b - phosphodiesterase 4b, camp specific  Mrc1 - mannose receptor, c type 1  Tnfrsf1b - tumor necrosis factor receptor superfamily, member 1b Sirpa - signal-regulatory protein alpha  Casp1 - caspase 1  Irg1 - immunoresponsive gene 1  Trem2 - triggering receptor expressed on myeloid cells 2  Gch1 - gtp cyclohydrolase 1  Cd274 - cd274 antigen  Tnfaip3 - tumor necrosis factor, alpha-induced protein 3 |
| GO:0051270 | regulation of cellular component movement | 2.65E-7 | 5.82E-5 | 2.69 (15721,1018,184,32) | Dsc2 - desmocollin 2 Trf - transferrin  Icam1 - intercellular adhesion molecule 1  Trem2 - triggering receptor expressed on myeloid cells 2  Fgr - gardner-rasheed feline sarcoma viral (fgr) oncogene homolog Cd274 - cd274 antigen  Fpr2 - formyl peptide receptor 2  Cd74 - cd74 antigen (invariant polypeptide of major histocompatibility complex, class ii antigen-associated)  Csf1r - colony stimulating factor 1 receptor  Cd9 - cd9 antigen  Mmp2 - matrix metallopeptidase 2  Clec7a - c-type lectin domain family 7, member a  Evl - ena-vasodilator stimulated phosphoprotein  Dab2 - disabled 2, mitogen-responsive phosphoprotein  Mmp12 - matrix metallopeptidase 12  Ccl4 - chemokine (c-c motif) ligand 4  Dach1 - dachshund 1 (drosophila)  P2rx4 - purinergic receptor p2x, ligand-gated ion channel 4  Gpnmb - glycoprotein (transmembrane) nmb  Csf1 - colony stimulating factor 1 (macrophage)  Ccl5 - chemokine (c-c motif) ligand 5  Itgax - integrin alpha x  Ccl2 - chemokine (c-c motif) ligand 2  Pde4b - phosphodiesterase 4b, camp specific  Ccr5 - chemokine (c-c motif) receptor 5  Lgals3 - lectin, galactose binding, soluble 3  Thy1 - thymus cell antigen 1, theta  Mpp1 - membrane protein, palmitoylated  Padi2 - peptidyl arginine deiminase, type ii  Il1rn - interleukin 1 receptor antagonist  Plxnc1 - plexin c1  Acvr1 - activin a receptor, type 1 |
|  |  |  |  |  | Il7r - interleukin 7 receptor  H2-Aa - histocompatibility 2, class ii antigen a, alpha  Inpp4b - inositol polyphosphate-4-phosphatase, type ii  Trem2 - triggering receptor expressed on myeloid cells 2  Fgl2 - fibrinogen-like protein 2  Cd74 - cd74 antigen (invariant polypeptide of major histocompatibility |

| GO:1902105 | regulation of leukocyte differentiation | 3.11E-7 | 6.72E-5 | 4.71 (15721,290,184,16) | complex, class ii antigen-associated)  H2-Oa - histocompatibility 2, o region alpha locus  Csf1r - colony stimulating factor 1 receptor  Ccl3 - chemokine (c-c motif) ligand 3  H2-M3 - histocompatibility 2, m region locus 3  Bmi1 - bmi1 polycomb ring finger oncogene  H2-DMa - histocompatibility 2, class ii, locus dma  Runx1 - runt related transcription factor 1  Egr3 - early growth response 3  Csf1 - colony stimulating factor 1 (macrophage)  Ccl5 - chemokine (c-c motif) ligand 5 |
| --- | --- | --- | --- | --- | --- |
| GO:0050764 | regulation of phagocytosis | 3.48E-7 | 7.41E-5 | 8.30 (15721,103,184,10) | Ccl2 - chemokine (c-c motif) ligand 2  Clec7a - c-type lectin domain family 7, member a  Sirpa - signal-regulatory protein alpha  Alox15 - arachidonate 15-lipoxygenase  Trem2 - triggering receptor expressed on myeloid cells 2  Fgr - gardner-rasheed feline sarcoma viral (fgr) oncogene homolog Slc11a1 - solute carrier family 11 (proton-coupled divalent metal ion transporters), member 1  Mfge8 - milk fat globule-egf factor 8 protein  Fpr2 - formyl peptide receptor 2  Hck - hemopoietic cell kinase |
| GO:1905523 | positive regulation of macrophage migration | 3.49E-7 | 7.33E-5 | 20.51 (15721,25,184,6) | Ccl2 - chemokine (c-c motif) ligand 2  Csf1r - colony stimulating factor 1 receptor  Trem2 - triggering receptor expressed on myeloid cells 2  P2rx4 - purinergic receptor p2x, ligand-gated ion channel 4  Ccl5 - chemokine (c-c motif) ligand 5  Csf1 - colony stimulating factor 1 (macrophage) |
| GO:1905521 | regulation of macrophage migration | 3.61E-7 | 7.49E-5 | 14.95 (15721,40,184,7) | Ccl2 - chemokine (c-c motif) ligand 2  Csf1r - colony stimulating factor 1 receptor  Cd9 - cd9 antigen  Trem2 - triggering receptor expressed on myeloid cells 2  P2rx4 - purinergic receptor p2x, ligand-gated ion channel 4  Ccl5 - chemokine (c-c motif) ligand 5  Csf1 - colony stimulating factor 1 (macrophage) |
| GO:0050778 | positive regulation of immune response | 3.66E-7 | 7.49E-5 | 3.62 (15721,496,184,21) | Pde4b - phosphodiesterase 4b, camp specific  H2-D1 - histocompatibility 2, d region locus 1  Ikbke - inhibitor of kappab kinase epsilon  H2-Ab1 - histocompatibility 2, class ii antigen a, beta 1  Fgr - gardner-rasheed feline sarcoma viral (fgr) oncogene homolog Cd274 - cd274 antigen  Thy1 - thymus cell antigen 1, theta  Fpr1 - formyl peptide receptor 1  Slc11a1 - solute carrier family 11 (proton-coupled divalent metal ion transporters), member 1  H2-K1 - histocompatibility 2, k1, k region  Fpr2 - formyl peptide receptor 2  Cd74 - cd74 antigen (invariant polypeptide of major histocompatibility complex, class ii antigen-associated)  Ifi205 - interferon activated gene 205  Prkcb - protein kinase c, beta  Slc15a3 - solute carrier family 15, member 3  Mmp2 - matrix metallopeptidase 2  Ifi203 - interferon activated gene 203  Mmp12 - matrix metallopeptidase 12  H2-M3 - histocompatibility 2, m region locus 3  Irg1 - immunoresponsive gene 1  H2-DMa - histocompatibility 2, class ii, locus dma |
|  |  |  |  |  | Trf - transferrin  Icam1 - intercellular adhesion molecule 1  Trem2 - triggering receptor expressed on myeloid cells 2  Cd274 - cd274 antigen  Fgr - gardner-rasheed feline sarcoma viral (fgr) oncogene homolog Fpr2 - formyl peptide receptor 2  Cd74 - cd74 antigen (invariant polypeptide of major histocompatibility complex, class ii antigen-associated)  Csf1r - colony stimulating factor 1 receptor  Cd9 - cd9 antigen  Mmp2 - matrix metallopeptidase 2  Clec7a - c-type lectin domain family 7, member a  Evl - ena-vasodilator stimulated phosphoprotein  Dab2 - disabled 2, mitogen-responsive phosphoprotein |

| GO:2000145 | regulation of cell motility | 4.32E-7 | 8.72E-5 | 2.74 (15721,935,184,30) | Mmp12 - matrix metallopeptidase 12  Ccl4 - chemokine (c-c motif) ligand 4  Dach1 - dachshund 1 (drosophila)  P2rx4 - purinergic receptor p2x, ligand-gated ion channel 4  Gpnmb - glycoprotein (transmembrane) nmb  Csf1 - colony stimulating factor 1 (macrophage)  Ccl5 - chemokine (c-c motif) ligand 5  Itgax - integrin alpha x  Ccl2 - chemokine (c-c motif) ligand 2  Ccr5 - chemokine (c-c motif) receptor 5  Lgals3 - lectin, galactose binding, soluble 3  Thy1 - thymus cell antigen 1, theta  Mpp1 - membrane protein, palmitoylated  Padi2 - peptidyl arginine deiminase, type ii  Il1rn - interleukin 1 receptor antagonist  Plxnc1 - plexin c1  Acvr1 - activin a receptor, type 1 |
| --- | --- | --- | --- | --- | --- |
| GO:0030334 | regulation of cell migration | 5.16E-7 | 1.03E-4 | 2.78 (15721,891,184,29) | Icam1 - intercellular adhesion molecule 1  Trem2 - triggering receptor expressed on myeloid cells 2  Cd274 - cd274 antigen  Fgr - gardner-rasheed feline sarcoma viral (fgr) oncogene homolog Fpr2 - formyl peptide receptor 2  Cd74 - cd74 antigen (invariant polypeptide of major histocompatibility complex, class ii antigen-associated)  Csf1r - colony stimulating factor 1 receptor  Cd9 - cd9 antigen  Mmp2 - matrix metallopeptidase 2  Clec7a - c-type lectin domain family 7, member a  Evl - ena-vasodilator stimulated phosphoprotein  Dab2 - disabled 2, mitogen-responsive phosphoprotein  Mmp12 - matrix metallopeptidase 12  Ccl4 - chemokine (c-c motif) ligand 4  Dach1 - dachshund 1 (drosophila)  P2rx4 - purinergic receptor p2x, ligand-gated ion channel 4  Gpnmb - glycoprotein (transmembrane) nmb  Csf1 - colony stimulating factor 1 (macrophage)  Ccl5 - chemokine (c-c motif) ligand 5  Itgax - integrin alpha x  Ccl2 - chemokine (c-c motif) ligand 2  Ccr5 - chemokine (c-c motif) receptor 5  Lgals3 - lectin, galactose binding, soluble 3  Thy1 - thymus cell antigen 1, theta  Mpp1 - membrane protein, palmitoylated  Padi2 - peptidyl arginine deiminase, type ii  Il1rn - interleukin 1 receptor antagonist  Plxnc1 - plexin c1  Acvr1 - activin a receptor, type 1 |
| GO:0051251 | positive regulation of lymphocyte activation | 5.87E-7 | 1.16E-4 | 4.80 (15721,267,184,15) | Ccl2 - chemokine (c-c motif) ligand 2  Il7r - interleukin 7 receptor  H2-Ab1 - histocompatibility 2, class ii antigen a, beta 1  H2-Aa - histocompatibility 2, class ii antigen a, alpha  Sirpa - signal-regulatory protein alpha  Cd274 - cd274 antigen  Thy1 - thymus cell antigen 1, theta  Cd74 - cd74 antigen (invariant polypeptide of major histocompatibility complex, class ii antigen-associated)  Clec7a - c-type lectin domain family 7, member a  H2-M3 - histocompatibility 2, m region locus 3  Bmi1 - bmi1 polycomb ring finger oncogene  H2-DMa - histocompatibility 2, class ii, locus dma  Runx1 - runt related transcription factor 1  Egr3 - early growth response 3  Ccl5 - chemokine (c-c motif) ligand 5 |
| GO:0050866 | negative regulation of cell activation | 6.28E-7 | 1.22E-4 | 5.58 (15721,199,184,13) | Lgals3 - lectin, galactose binding, soluble 3  H2-Ab1 - histocompatibility 2, class ii antigen a, beta 1  H2-Aa - histocompatibility 2, class ii antigen a, alpha  Cst7 - cystatin f (leukocystatin)  Fgr - gardner-rasheed feline sarcoma viral (fgr) oncogene homolog Cd274 - cd274 antigen  Fgl2 - fibrinogen-like protein 2  Cd74 - cd74 antigen (invariant polypeptide of major histocompatibility complex, class ii antigen-associated)  Cd9 - cd9 antigen  H2-M3 - histocompatibility 2, m region locus 3  Runx1 - runt related transcription factor 1  Tnfaip3 - tumor necrosis factor, alpha-induced protein 3 |

|  |  |  |  |  | Gpnmb - glycoprotein (transmembrane) nmb |
| --- | --- | --- | --- | --- | --- |
| GO:0097529 | myeloid leukocyte migration | 7.62E-7 | 1.46E-4 | 7.63 (15721,112,184,10) | Cxcl2 - chemokine (c-x-c motif) ligand 2  Ccl2 - chemokine (c-c motif) ligand 2  Lgals3 - lectin, galactose binding, soluble 3  Ccl17 - chemokine (c-c motif) ligand 17  Pde4b - phosphodiesterase 4b, camp specific  Sirpa - signal-regulatory protein alpha  Ccl3 - chemokine (c-c motif) ligand 3  Epx - eosinophil peroxidase  Ccl4 - chemokine (c-c motif) ligand 4  Ccl5 - chemokine (c-c motif) ligand 5 |
| GO:0072677 | eosinophil migration | 8.18E-7 | 1.55E-4 | 26.70 (15721,16,184,5) | Ccl2 - chemokine (c-c motif) ligand 2  Lgals3 - lectin, galactose binding, soluble 3  Epx - eosinophil peroxidase  Ccl4 - chemokine (c-c motif) ligand 4  Ccl5 - chemokine (c-c motif) ligand 5 |
| GO:0002548 | monocyte chemotaxis | 9E-7 | 1.68E-4 | 17.68 (15721,29,184,6) | Ccl2 - chemokine (c-c motif) ligand 2  Lgals3 - lectin, galactose binding, soluble 3  Ccl17 - chemokine (c-c motif) ligand 17  Ccl3 - chemokine (c-c motif) ligand 3  Ccl4 - chemokine (c-c motif) ligand 4  Ccl5 - chemokine (c-c motif) ligand 5 |
| GO:0030574 | collagen catabolic process | 9E-7 | 1.66E-4 | 17.68 (15721,29,184,6) | Mmp2 - matrix metallopeptidase 2  Mmp13 - matrix metallopeptidase 13  Ctsb - cathepsin b  Mmp12 - matrix metallopeptidase 12  Mmp8 - matrix metallopeptidase 8  Ctss - cathepsin s |
| GO:0006950 | response to stress | 9.7E-7 | 1.77E-4 | 1.89 (15721,2480,184,55) | Dsc2 - desmocollin 2  Crip1 - cysteine-rich protein 1 (intestinal)  Zmat3 - zinc finger matrin type 3  Acot2 - acyl-coa thioesterase 2  Trf - transferrin  Fpr1 - formyl peptide receptor 1  Fpr2 - formyl peptide receptor 2  Prkcb - protein kinase c, beta  Cd74 - cd74 antigen (invariant polypeptide of major histocompatibility complex, class ii antigen-associated)  Mmp2 - matrix metallopeptidase 2  Mmp12 - matrix metallopeptidase 12  H2-M3 - histocompatibility 2, m region locus 3  Gch1 - gtp cyclohydrolase 1  Saa3 - serum amyloid a 3  P2rx4 - purinergic receptor p2x, ligand-gated ion channel 4  Ear2 - eosinophil-associated, ribonuclease a family, member 2  Ccl2 - chemokine (c-c motif) ligand 2  Itgax - integrin alpha x  Pde4b - phosphodiesterase 4b, camp specific  Ccl17 - chemokine (c-c motif) ligand 17  Tnfrsf1b - tumor necrosis factor receptor superfamily, member 1b Clec4n - c-type lectin domain family 4, member n  Alox15 - arachidonate 15-lipoxygenase  Slc11a1 - solute carrier family 11 (proton-coupled divalent metal ion transporters), member 1  Polr3k - polymerase (rna) iii (dna directed) polypeptide k  Mcts1 - malignant t cell amplified sequence 1  Acvr1 - activin a receptor, type 1  Ccrl2 - chemokine (c-c motif) receptor-like 2  Pld4 - phospholipase d family, member 4  Tnfaip3 - tumor necrosis factor, alpha-induced protein 3  Ikbke - inhibitor of kappab kinase epsilon  Icam1 - intercellular adhesion molecule 1  Trem2 - triggering receptor expressed on myeloid cells 2  Fgr - gardner-rasheed feline sarcoma viral (fgr) oncogene homolog Hck - hemopoietic cell kinase  Cxcl2 - chemokine (c-x-c motif) ligand 2  Slc15a3 - solute carrier family 15, member 3  Csf1r - colony stimulating factor 1 receptor  Ccl3 - chemokine (c-c motif) ligand 3  Ccl4 - chemokine (c-c motif) ligand 4  Irg1 - immunoresponsive gene 1  Csf1 - colony stimulating factor 1 (macrophage)  Ccl5 - chemokine (c-c motif) ligand 5 |

|  |  |  |  |  | Lgals3 - lectin, galactose binding, soluble 3  Ccr5 - chemokine (c-c motif) receptor 5  Il7r - interleukin 7 receptor  Sirpa - signal-regulatory protein alpha  Casp1 - caspase 1  Epx - eosinophil peroxidase  Wfdc17 - wap four-disulfide core domain 17  H2-K1 - histocompatibility 2, k1, k region  Il1rn - interleukin 1 receptor antagonist  Serpinb2 - serine (or cysteine) peptidase inhibitor, clade b, member 2 Pld3 - phospholipase d family, member 3  Prg2 - proteoglycan 2, bone marrow |
| --- | --- | --- | --- | --- | --- |
| GO:0002237 | response to molecule of bacterial origin | 1.05E-6 | 1.89E-4 | 4.92 (15721,243,184,14) | Ccl2 - chemokine (c-c motif) ligand 2  Pde4b - phosphodiesterase 4b, camp specific  Mrc1 - mannose receptor, c type 1  Tnfrsf1b - tumor necrosis factor receptor superfamily, member 1b Sirpa - signal-regulatory protein alpha  Casp1 - caspase 1  Trem2 - triggering receptor expressed on myeloid cells 2  Cd274 - cd274 antigen  Slc11a1 - solute carrier family 11 (proton-coupled divalent metal ion transporters), member 1  Cxcl2 - chemokine (c-x-c motif) ligand 2  H2-M3 - histocompatibility 2, m region locus 3  Irg1 - immunoresponsive gene 1  Gch1 - gtp cyclohydrolase 1  Tnfaip3 - tumor necrosis factor, alpha-induced protein 3 |
| GO:0006935 | chemotaxis | 1.07E-6 | 1.91E-4 | 4.58 (15721,280,184,15) | Ccl2 - chemokine (c-c motif) ligand 2  Ear2 - eosinophil-associated, ribonuclease a family, member 2 Ccl17 - chemokine (c-c motif) ligand 17  Lgals3 - lectin, galactose binding, soluble 3  Pde4b - phosphodiesterase 4b, camp specific  Ccr5 - chemokine (c-c motif) receptor 5  Fpr1 - formyl peptide receptor 1  Fpr2 - formyl peptide receptor 2  Cxcl2 - chemokine (c-x-c motif) ligand 2  Ccl3 - chemokine (c-c motif) ligand 3  Ccl4 - chemokine (c-c motif) ligand 4  Ccrl2 - chemokine (c-c motif) receptor-like 2  Saa3 - serum amyloid a 3  Egr3 - early growth response 3  Ccl5 - chemokine (c-c motif) ligand 5 |
| GO:0071222 | cellular response to  lipo-polysaccharide | 1.09E-6 | 1.92E-4 | 6.48 (15721,145,184,11) | Cxcl2 - chemokine (c-x-c motif) ligand 2  Ccl2 - chemokine (c-c motif) ligand 2  Pde4b - phosphodiesterase 4b, camp specific  Mrc1 - mannose receptor, c type 1  Tnfrsf1b - tumor necrosis factor receptor superfamily, member 1b Sirpa - signal-regulatory protein alpha  Casp1 - caspase 1  Irg1 - immunoresponsive gene 1  Gch1 - gtp cyclohydrolase 1  Cd274 - cd274 antigen  Tnfaip3 - tumor necrosis factor, alpha-induced protein 3 |
| GO:0030593 | neutrophil chemotaxis | 1.23E-6 | 2.14E-4 | 10.05 (15721,68,184,8) | Cxcl2 - chemokine (c-x-c motif) ligand 2  Ccl2 - chemokine (c-c motif) ligand 2  Lgals3 - lectin, galactose binding, soluble 3  Ccl17 - chemokine (c-c motif) ligand 17  Pde4b - phosphodiesterase 4b, camp specific  Ccl3 - chemokine (c-c motif) ligand 3  Ccl4 - chemokine (c-c motif) ligand 4  Ccl5 - chemokine (c-c motif) ligand 5 |
| GO:0002695 | negative regulation of leukocyte activation | 1.23E-6 | 2.13E-4 | 5.76 (15721,178,184,12) | Cd74 - cd74 antigen (invariant polypeptide of major histocompatibility complex, class ii antigen-associated)  Lgals3 - lectin, galactose binding, soluble 3  H2-Ab1 - histocompatibility 2, class ii antigen a, beta 1  H2-Aa - histocompatibility 2, class ii antigen a, alpha  H2-M3 - histocompatibility 2, m region locus 3  Fgr - gardner-rasheed feline sarcoma viral (fgr) oncogene homolog Cd274 - cd274 antigen  Cst7 - cystatin f (leukocystatin) Fgl2 - fibrinogen-like protein 2  Runx1 - runt related transcription factor 1 |

|  |  |  |  |  | Tnfaip3 - tumor necrosis factor, alpha-induced protein 3  Gpnmb - glycoprotein (transmembrane) nmb |
| --- | --- | --- | --- | --- | --- |
| GO:0042330 | taxis | 1.33E-6 | 2.28E-4 | 4.50 (15721,285,184,15) | Ccl2 - chemokine (c-c motif) ligand 2  Ear2 - eosinophil-associated, ribonuclease a family, member 2 Ccl17 - chemokine (c-c motif) ligand 17  Lgals3 - lectin, galactose binding, soluble 3  Pde4b - phosphodiesterase 4b, camp specific  Ccr5 - chemokine (c-c motif) receptor 5  Fpr1 - formyl peptide receptor 1  Fpr2 - formyl peptide receptor 2  Cxcl2 - chemokine (c-x-c motif) ligand 2  Ccl3 - chemokine (c-c motif) ligand 3  Ccl4 - chemokine (c-c motif) ligand 4  Ccrl2 - chemokine (c-c motif) receptor-like 2  Saa3 - serum amyloid a 3  Egr3 - early growth response 3  Ccl5 - chemokine (c-c motif) ligand 5 |
| GO:0022408 | negative regulation of cell-cell adhesion | 1.39E-6 | 2.34E-4 | 5.70 (15721,180,184,12) | Cd74 - cd74 antigen (invariant polypeptide of major histocompatibility complex, class ii antigen-associated)  Lgals3 - lectin, galactose binding, soluble 3  Cd9 - cd9 antigen  Il1rn - interleukin 1 receptor antagonist  H2-Ab1 - histocompatibility 2, class ii antigen a, beta 1  H2-Aa - histocompatibility 2, class ii antigen a, alpha  H2-M3 - histocompatibility 2, m region locus 3  Cd274 - cd274 antigen  Fgl2 - fibrinogen-like protein 2  Runx1 - runt related transcription factor 1  Tnfaip3 - tumor necrosis factor, alpha-induced protein 3  Gpnmb - glycoprotein (transmembrane) nmb |
| GO:0051250 | negative regulation of lymphocyte activation | 1.42E-6 | 2.37E-4 | 6.31 (15721,149,184,11) | Cd74 - cd74 antigen (invariant polypeptide of major histocompatibility complex, class ii antigen-associated)  Lgals3 - lectin, galactose binding, soluble 3  H2-Ab1 - histocompatibility 2, class ii antigen a, beta 1  H2-Aa - histocompatibility 2, class ii antigen a, alpha  H2-M3 - histocompatibility 2, m region locus 3  Fgr - gardner-rasheed feline sarcoma viral (fgr) oncogene homolog Cd274 - cd274 antigen  Fgl2 - fibrinogen-like protein 2  Runx1 - runt related transcription factor 1  Tnfaip3 - tumor necrosis factor, alpha-induced protein 3  Gpnmb - glycoprotein (transmembrane) nmb |
| GO:0051241 | negative regulation of multicellular organismal process | 1.5E-6 | 2.47E-4 | 2.35 (15721,1270,184,35) | Rnf128 - ring finger protein 128  Inpp4b - inositol polyphosphate-4-phosphatase, type ii  Trem2 - triggering receptor expressed on myeloid cells 2  Cd274 - cd274 antigen  Fgl2 - fibrinogen-like protein 2  Il1rl1 - interleukin 1 receptor-like 1  Cd74 - cd74 antigen (invariant polypeptide of major histocompatibility complex, class ii antigen-associated)  Cd9 - cd9 antigen  Mmp2 - matrix metallopeptidase 2  Evl - ena-vasodilator stimulated phosphoprotein  Dab2 - disabled 2, mitogen-responsive phosphoprotein  H2-M3 - histocompatibility 2, m region locus 3  Irg1 - immunoresponsive gene 1  P2rx4 - purinergic receptor p2x, ligand-gated ion channel 4  Gpnmb - glycoprotein (transmembrane) nmb  Procr - protein c receptor, endothelial  Ccl2 - chemokine (c-c motif) ligand 2  Pde4b - phosphodiesterase 4b, camp specific  H2-D1 - histocompatibility 2, d region locus 1  Ccr5 - chemokine (c-c motif) receptor 5  Ndrg2 - n-myc downstream regulated gene 2  Tnfrsf1b - tumor necrosis factor receptor superfamily, member 1b Sirpa - signal-regulatory protein alpha  Tnfrsf9 - tumor necrosis factor receptor superfamily, member 9  Epx - eosinophil peroxidase  Thy1 - thymus cell antigen 1, theta  Slc11a1 - solute carrier family 11 (proton-coupled divalent metal ion transporters), member 1  H2-K1 - histocompatibility 2, k1, k region  Mmp8 - matrix metallopeptidase 8  Inhba - inhibin beta-a  Cd34 - cd34 antigen |

|  |  |  |  |  | Runx1 - runt related transcription factor 1  Tnfaip3 - tumor necrosis factor, alpha-induced protein 3  Prg2 - proteoglycan 2, bone marrow  Zhx2 - zinc fingers and homeoboxes 2 |
| --- | --- | --- | --- | --- | --- |
| GO:0032693 | negative regulation of interleukin-10 production | 1.57E-6 | 2.57E-4 | 23.73 (15721,18,184,5) | Tnfrsf9 - tumor necrosis factor receptor superfamily, member 9  Epx - eosinophil peroxidase  Cd274 - cd274 antigen  Mmp8 - matrix metallopeptidase 8  Prg2 - proteoglycan 2, bone marrow |
| GO:0032653 | regulation of interleukin-10 production | 1.75E-6 | 2.83E-4 | 11.96 (15721,50,184,7) | Cd34 - cd34 antigen  Epx - eosinophil peroxidase  Tnfrsf9 - tumor necrosis factor receptor superfamily, member 9  Trem2 - triggering receptor expressed on myeloid cells 2  Cd274 - cd274 antigen  Mmp8 - matrix metallopeptidase 8  Prg2 - proteoglycan 2, bone marrow |
| GO:0070372 | regulation of ERK1 and ERK2 cascade | 1.81E-6 | 2.89E-4 | 4.39 (15721,292,184,15) | Ccl2 - chemokine (c-c motif) ligand 2  Ccl17 - chemokine (c-c motif) ligand 17  Ndrg2 - n-myc downstream regulated gene 2  Sirpa - signal-regulatory protein alpha  Icam1 - intercellular adhesion molecule 1  Alox15 - arachidonate 15-lipoxygenase  Trem2 - triggering receptor expressed on myeloid cells 2  Fpr2 - formyl peptide receptor 2  Cd74 - cd74 antigen (invariant polypeptide of major histocompatibility complex, class ii antigen-associated)  Csf1r - colony stimulating factor 1 receptor  Dab2 - disabled 2, mitogen-responsive phosphoprotein  Ccl3 - chemokine (c-c motif) ligand 3  Ccl4 - chemokine (c-c motif) ligand 4  Ccl5 - chemokine (c-c motif) ligand 5  Gpnmb - glycoprotein (transmembrane) nmb |
| GO:0060326 | cell chemotaxis | 1.85E-6 | 2.93E-4 | 5.54 (15721,185,184,12) | Cxcl2 - chemokine (c-x-c motif) ligand 2  Ccl2 - chemokine (c-c motif) ligand 2  Lgals3 - lectin, galactose binding, soluble 3  Ccl17 - chemokine (c-c motif) ligand 17  Ccr5 - chemokine (c-c motif) receptor 5  Pde4b - phosphodiesterase 4b, camp specific  Ccl3 - chemokine (c-c motif) ligand 3  Ccl4 - chemokine (c-c motif) ligand 4  Ccrl2 - chemokine (c-c motif) receptor-like 2  Saa3 - serum amyloid a 3  Egr3 - early growth response 3  Ccl5 - chemokine (c-c motif) ligand 5 |
| GO:0040012 | regulation of locomotion | 2.1E-6 | 3.29E-4 | 2.54 (15721,1009,184,30) | Trf - transferrin  Icam1 - intercellular adhesion molecule 1  Trem2 - triggering receptor expressed on myeloid cells 2  Cd274 - cd274 antigen  Fgr - gardner-rasheed feline sarcoma viral (fgr) oncogene homolog  Fpr2 - formyl peptide receptor 2  Cd74 - cd74 antigen (invariant polypeptide of major histocompatibility complex, class ii antigen-associated)  Csf1r - colony stimulating factor 1 receptor  Cd9 - cd9 antigen  Mmp2 - matrix metallopeptidase 2  Clec7a - c-type lectin domain family 7, member a  Evl - ena-vasodilator stimulated phosphoprotein  Dab2 - disabled 2, mitogen-responsive phosphoprotein  Mmp12 - matrix metallopeptidase 12  Ccl4 - chemokine (c-c motif) ligand 4  Dach1 - dachshund 1 (drosophila)  P2rx4 - purinergic receptor p2x, ligand-gated ion channel 4  Gpnmb - glycoprotein (transmembrane) nmb  Csf1 - colony stimulating factor 1 (macrophage)  Ccl5 - chemokine (c-c motif) ligand 5  Itgax - integrin alpha x  Ccl2 - chemokine (c-c motif) ligand 2  Ccr5 - chemokine (c-c motif) receptor 5  Lgals3 - lectin, galactose binding, soluble 3  Thy1 - thymus cell antigen 1, theta  Mpp1 - membrane protein, palmitoylated  Padi2 - peptidyl arginine deiminase, type ii |

|  |  |  |  |  | Il1rn - interleukin 1 receptor antagonist  Plxnc1 - plexin c1  Acvr1 - activin a receptor, type 1 |
| --- | --- | --- | --- | --- | --- |
| GO:0002579 | positive regulation of antigen processing and presentation | 2.19E-6 | 3.39E-4 | 37.97 (15721,9,184,4) | Cd74 - cd74 antigen (invariant polypeptide of major histocompatibility complex, class ii antigen-associated)  H2-Ab1 - histocompatibility 2, class ii antigen a, beta 1  Trem2 - triggering receptor expressed on myeloid cells 2  Slc11a1 - solute carrier family 11 (proton-coupled divalent metal ion transporters), member 1 |
| GO:0002822 | regulation of adaptive immune response based on somatic recombination of immune receptors built from immunoglobulin superfamily domains | 2.24E-6 | 3.43E-4 | 6.02 (15721,156,184,11) | H2-D1 - histocompatibility 2, d region locus 1  Il7r - interleukin 7 receptor  H2-Ab1 - histocompatibility 2, class ii antigen a, beta 1  Tnfrsf1b - tumor necrosis factor receptor superfamily, member 1b  H2-M3 - histocompatibility 2, m region locus 3  Cd274 - cd274 antigen  Slc11a1 - solute carrier family 11 (proton-coupled divalent metal ion transporters), member 1  H2-DMa - histocompatibility 2, class ii, locus dma  H2-K1 - histocompatibility 2, k1, k region  Tnfaip3 - tumor necrosis factor, alpha-induced protein 3  Il1rl1 - interleukin 1 receptor-like 1 |
| GO:0045597 | positive regulation of cell differentiation | 2.28E-6 | 3.47E-4 | 2.48 (15721,1068,184,31) | Cebpa - ccaat/enhancer binding protein (c/ebp), alpha  Trf - transferrin  Rnf157 - ring finger protein 157  Trem2 - triggering receptor expressed on myeloid cells 2  Akap11 - a kinase (prka) anchor protein 11  Cd74 - cd74 antigen (invariant polypeptide of major histocompatibility complex, class ii antigen-associated)  Csf1r - colony stimulating factor 1 receptor  Csf2 - colony stimulating factor 2 (granulocyte-macrophage)  Socs3 - suppressor of cytokine signaling 3  Fam20c - family with sequence similarity 20, member c  Junb - jun-b oncogene  Ccl3 - chemokine (c-c motif) ligand 3  Dab2 - disabled 2, mitogen-responsive phosphoprotein  H2-M3 - histocompatibility 2, m region locus 3  P2rx4 - purinergic receptor p2x, ligand-gated ion channel 4  Bmi1 - bmi1 polycomb ring finger oncogene  H2-DMa - histocompatibility 2, class ii, locus dma  Ccl5 - chemokine (c-c motif) ligand 5  Csf1 - colony stimulating factor 1 (macrophage)  Ccr5 - chemokine (c-c motif) receptor 5  Il7r - interleukin 7 receptor  Tnfrsf1b - tumor necrosis factor receptor superfamily, member 1b  H2-Aa - histocompatibility 2, class ii antigen a, alpha  Zeb2 - zinc finger e-box binding homeobox 2  Cd34 - cd34 antigen  Inhba - inhibin beta-a Plxnc1 - plexin c1  Acvr1 - activin a receptor, type 1  Runx1 - runt related transcription factor 1  Egr3 - early growth response 3  Egr2 - early growth response 2 |
| GO:2000147 | positive regulation of cell motility | 2.43E-6 | 3.66E-4 | 3.22 (15721,558,184,21) | Itgax - integrin alpha x  Ccl2 - chemokine (c-c motif) ligand 2  Lgals3 - lectin, galactose binding, soluble 3  Icam1 - intercellular adhesion molecule 1  Trf - transferrin  Trem2 - triggering receptor expressed on myeloid cells 2  Cd274 - cd274 antigen  Fgr - gardner-rasheed feline sarcoma viral (fgr) oncogene homolog  Thy1 - thymus cell antigen 1, theta  Fpr2 - formyl peptide receptor 2  Cd74 - cd74 antigen (invariant polypeptide of major histocompatibility complex, class ii antigen-associated)  Csf1r - colony stimulating factor 1 receptor  Mmp2 - matrix metallopeptidase 2  Clec7a - c-type lectin domain family 7, member a  Acvr1 - activin a receptor, type 1  Dab2 - disabled 2, mitogen-responsive phosphoprotein  Ccl4 - chemokine (c-c motif) ligand 4  P2rx4 - purinergic receptor p2x, ligand-gated ion channel 4  Csf1 - colony stimulating factor 1 (macrophage)  Gpnmb - glycoprotein (transmembrane) nmb |

|  |  |  |  |  | Ccl5 - chemokine (c-c motif) ligand 5 |
| --- | --- | --- | --- | --- | --- |
| GO:0050900 | leukocyte migration | 2.45E-6 | 3.65E-4 | 5.40 (15721,190,184,12) | Cxcl2 - chemokine (c-x-c motif) ligand 2  Ccl2 - chemokine (c-c motif) ligand 2  Lgals3 - lectin, galactose binding, soluble 3  Cd34 - cd34 antigen  Ccl17 - chemokine (c-c motif) ligand 17  Pde4b - phosphodiesterase 4b, camp specific  Sirpa - signal-regulatory protein alpha  Icam1 - intercellular adhesion molecule 1  Ccl3 - chemokine (c-c motif) ligand 3  Epx - eosinophil peroxidase  Ccl4 - chemokine (c-c motif) ligand 4  Ccl5 - chemokine (c-c motif) ligand 5 |
| GO:0002573 | myeloid leukocyte differentiation | 2.58E-6 | 3.81E-4 | 7.69 (15721,100,184,9) | Csf1r - colony stimulating factor 1 receptor  Csf2 - colony stimulating factor 2 (granulocyte-macrophage)  Cebpa - ccaat/enhancer binding protein (c/ebp), alpha  Trf - transferrin  Snx10 - sorting nexin 10 Junb - jun-b oncogene  Il3ra - interleukin 3 receptor, alpha chain  Trem2 - triggering receptor expressed on myeloid cells 2  Csf1 - colony stimulating factor 1 (macrophage) |
| GO:0071621 | granulocyte chemotaxis | 2.61E-6 | 3.82E-4 | 9.11 (15721,75,184,8) | Cxcl2 - chemokine (c-x-c motif) ligand 2  Ccl2 - chemokine (c-c motif) ligand 2  Lgals3 - lectin, galactose binding, soluble 3  Pde4b - phosphodiesterase 4b, camp specific  Ccl17 - chemokine (c-c motif) ligand 17  Ccl3 - chemokine (c-c motif) ligand 3  Ccl4 - chemokine (c-c motif) ligand 4  Ccl5 - chemokine (c-c motif) ligand 5 |
|  |  |  |  |  | Dsc2 - desmocollin 2  Slc22a3 - solute carrier family 22 (organic cation transporter), member 3  Decr1 - 2,4-dienoyl coa reductase 1, mitochondrial  Akap11 - a kinase (prka) anchor protein 11  Prkcb - protein kinase c, beta  H2-Oa - histocompatibility 2, o region alpha locus  Socs3 - suppressor of cytokine signaling 3  Snx10 - sorting nexin 10  Hivep3 - human immunodeficiency virus type i enhancer binding protein 3  Dab2 - disabled 2, mitogen-responsive phosphoprotein  H2-M3 - histocompatibility 2, m region locus 3  Gch1 - gtp cyclohydrolase 1  Dach1 - dachshund 1 (drosophila)  H2-DMa - histocompatibility 2, class ii, locus dma  Slc2a6 - solute carrier family 2 (facilitated glucose transporter), member 6  Neto2 - neuropilin (nrp) and tolloid (tll)-like 2  Pde4b - phosphodiesterase 4b, camp specific  Fabp5 - fatty acid binding protein 5, epidermal  Sestd1 - sec14 and spectrin domains 1  Slc11a1 - solute carrier family 11 (proton-coupled divalent metal ion transporters), member 1  Padi2 - peptidyl arginine deiminase, type ii  Mcts1 - malignant t cell amplified sequence 1  Plxnc1 - plexin c1  Acvr1 - activin a receptor, type 1  Parvb - parvin, beta  Lpxn - leupaxin  Pilra - paired immunoglobin-like type 2 receptor alpha  Zhx2 - zinc fingers and homeoboxes 2  Cyp4f18 - cytochrome p450, family 4, subfamily f, polypeptide 18 Ikbke - inhibitor of kappab kinase epsilon  Fam46a - family with sequence similarity 46, member a  Icam1 - intercellular adhesion molecule 1  Ctsb - cathepsin b  Inpp4b - inositol polyphosphate-4-phosphatase, type ii  Slc6a12 - solute carrier family 6 (neurotransmitter transporter, betaine/gaba), member 12  Trem2 - triggering receptor expressed on myeloid cells 2  Cd274 - cd274 antigen  Cst7 - cystatin f (leukocystatin)  Emr4 - egf-like module containing, mucin-like, hormone receptor-like sequence 4  Il1rl1 - interleukin 1 receptor-like 1  Ifi30 - interferon gamma inducible protein 30 |

| GO:0065007 | biological regulation | 2.61E-6 | 3.79E-4 | 1.25 (15721,9909,184,145) | Hck - hemopoietic cell kinase  Slc15a3 - solute carrier family 15, member 3  Ifi203 - interferon activated gene 203  Evl - ena-vasodilator stimulated phosphoprotein  Irg1 - immunoresponsive gene 1  Ctss - cathepsin s  Ccr5 - chemokine (c-c motif) receptor 5  Paox - polyamine oxidase (exo-n4-amino)  Casp1 - caspase 1  Sirpa - signal-regulatory protein alpha  Ifi27 - interferon, alpha-inducible protein 27  Epx - eosinophil peroxidase  Uty - ubiquitously transcribed tetratricopeptide repeat gene, y chromosome  Inhba - inhibin beta-a  Serpinb2 - serine (or cysteine) peptidase inhibitor, clade b, member 2 Pld3 - phospholipase d family, member 3  Hdc - histidine decarboxylase  Runx1 - runt related transcription factor 1  Nfkbie - nuclear factor of kappa light polypeptide gene enhancer in b cells inhibitor, epsilon  Mgll - monoglyceride lipase  Crip1 - cysteine-rich protein 1 (intestinal)  Zmat3 - zinc finger matrin type 3  Mtmr9 - myotubularin related protein 9  Trf - transferrin  Acot1 - acyl-coa thioesterase 1  Fpr1 - formyl peptide receptor 1  Eif2s3y - eukaryotic translation initiation factor 2, subunit 3, structural gene y-linked  Fpr2 - formyl peptide receptor 2  Cd74 - cd74 antigen (invariant polypeptide of major histocompatibility complex, class ii antigen-associated)  Cd9 - cd9 antigen  Mmp2 - matrix metallopeptidase 2  Mmp13 - matrix metallopeptidase 13  Junb - jun-b oncogene  Mmp12 - matrix metallopeptidase 12  P2rx4 - purinergic receptor p2x, ligand-gated ion channel 4  Gpnmb - glycoprotein (transmembrane) nmb  Itgax - integrin alpha x  Ccl2 - chemokine (c-c motif) ligand 2  Ccl17 - chemokine (c-c motif) ligand 17  Cp - ceruloplasmin  Tnfrsf1b - tumor necrosis factor receptor superfamily, member 1b Clec4n - c-type lectin domain family 4, member n  Fmnl2 - formin-like 2  Alox15 - arachidonate 15-lipoxygenase  Tnfrsf9 - tumor necrosis factor receptor superfamily, member 9 Mmp8 - matrix metallopeptidase 8  Actr3b - arp3 actin-related protein 3b  Ifi205 - interferon activated gene 205  Mpp1 - membrane protein, palmitoylated  Cd34 - cd34 antigen  P2ry14 - purinergic receptor p2y, g-protein coupled, 14  Pygl - liver glycogen phosphorylase  Ngfrap1 - nerve growth factor receptor (tnfrsf16) associated protein 1 Ccrl2 - chemokine (c-c motif) receptor-like 2  Stxbp6 - syntaxin binding protein 6 (amisyn)  Nenf - neuron derived neurotrophic factor  Pld4 - phospholipase d family, member 4  Tnfaip3 - tumor necrosis factor, alpha-induced protein 3  Rnf128 - ring finger protein 128  Chst11 - carbohydrate sulfotransferase 11  AF251705 - cdna sequence af251705  Cebpa - ccaat/enhancer binding protein (c/ebp), alpha  Rnf157 - ring finger protein 157  Acsl4 - acyl-coa synthetase long-chain family member 4  Siglec5 - sialic acid binding ig-like lectin 5  Gm2a - gm2 ganglioside activator protein  Fgr - gardner-rasheed feline sarcoma viral (fgr) oncogene homolog Fgl2 - fibrinogen-like protein 2  Ppap2c - phosphatidic acid phosphatase type 2c  Cxcl2 - chemokine (c-x-c motif) ligand 2  Csf1r - colony stimulating factor 1 receptor  Csf2ra - colony stimulating factor 2 receptor, alpha, low-affinity (granulocyte-macrophage)  Csf2 - colony stimulating factor 2 (granulocyte-macrophage)  Avpi1 - arginine vasopressin-induced 1  Clec7a - c-type lectin domain family 7, member a Fam20c - family with sequence similarity 20, member c  Ccl3 - chemokine (c-c motif) ligand 3  Ccl4 - chemokine (c-c motif) ligand 4 |
| --- | --- | --- | --- | --- | --- |

|  |  |  |  |  | Degs1 - degenerative spermatocyte homolog 1 (drosophila)  Bmi1 - bmi1 polycomb ring finger oncogene  Csf1 - colony stimulating factor 1 (macrophage)  Ccl5 - chemokine (c-c motif) ligand 5  Procr - protein c receptor, endothelial  Igfbp7 - insulin-like growth factor binding protein 7  H2-D1 - histocompatibility 2, d region locus 1  Cd200r3 - cd200 receptor 3  Lgals3 - lectin, galactose binding, soluble 3  Il7r - interleukin 7 receptor  Ndrg2 - n-myc downstream regulated gene 2  H2-Ab1 - histocompatibility 2, class ii antigen a, beta 1  H2-Aa - histocompatibility 2, class ii antigen a, alpha  Atrnl1 - attractin like 1  Thy1 - thymus cell antigen 1, theta  Atp6v0d2 - atpase, h+ transporting, lysosomal v0 subunit d2  H2-K1 - histocompatibility 2, k1, k region  Zeb2 - zinc finger e-box binding homeobox 2  Tgm2 - transglutaminase 2, c polypeptide  Kdm5d - lysine (k)-specific demethylase 5d  Il1rn - interleukin 1 receptor antagonist  Il3ra - interleukin 3 receptor, alpha chain  Egr3 - early growth response 3  Egr2 - early growth response 2  Mfge8 - milk fat globule-egf factor 8 protein  Prg2 - proteoglycan 2, bone marrow |
| --- | --- | --- | --- | --- | --- |
| GO:0031643 | positive regulation of myelination | 2.8E-6 | 4.01E-4 | 21.36 (15721,20,184,5) | Itgax - integrin alpha x  Tnfrsf1b - tumor necrosis factor receptor superfamily, member 1b Trf - transferrin  Cst7 - cystatin f (leukocystatin)  Egr2 - early growth response 2 |
| GO:1903555 | regulation of tumor necrosis factor superfamily cytokine production | 2.86E-6 | 4.07E-4 | 5.87 (15721,160,184,11) | Ccl2 - chemokine (c-c motif) ligand 2  Cd34 - cd34 antigen  Ccr5 - chemokine (c-c motif) receptor 5  Sirpa - signal-regulatory protein alpha  Ccl3 - chemokine (c-c motif) ligand 3  Ccl4 - chemokine (c-c motif) ligand 4  Trem2 - triggering receptor expressed on myeloid cells 2  Cd274 - cd274 antigen  Tnfaip3 - tumor necrosis factor, alpha-induced protein 3  Mmp8 - matrix metallopeptidase 8  Gpnmb - glycoprotein (transmembrane) nmb |
| GO:0032496 | response to lipopolysaccharide | 2.89E-6 | 4.07E-4 | 4.87 (15721,228,184,13) | Ccl2 - chemokine (c-c motif) ligand 2  Pde4b - phosphodiesterase 4b, camp specific  Mrc1 - mannose receptor, c type 1  Tnfrsf1b - tumor necrosis factor receptor superfamily, member 1b Sirpa - signal-regulatory protein alpha  Casp1 - caspase 1  Trem2 - triggering receptor expressed on myeloid cells 2  Cd274 - cd274 antigen  Slc11a1 - solute carrier family 11 (proton-coupled divalent metal ion transporters), member 1  Cxcl2 - chemokine (c-x-c motif) ligand 2  Irg1 - immunoresponsive gene 1  Gch1 - gtp cyclohydrolase 1  Tnfaip3 - tumor necrosis factor, alpha-induced protein 3 |
| GO:0030316 | osteoclast differentiation | 2.9E-6 | 4.05E-4 | 14.65 (15721,35,184,6) | Csf1r - colony stimulating factor 1 receptor  Snx10 - sorting nexin 10  Trf - transferrin  Junb - jun-b oncogene  Trem2 - triggering receptor expressed on myeloid cells 2  Csf1 - colony stimulating factor 1 (macrophage) |
| GO:1903706 | regulation of hemopoiesis | 2.99E-6 | 4.13E-4 | 3.76 (15721,386,184,17) | Il7r - interleukin 7 receptor  H2-Aa - histocompatibility 2, class ii antigen a, alpha  Inpp4b - inositol polyphosphate-4-phosphatase, type ii  Trem2 - triggering receptor expressed on myeloid cells 2  Fgl2 - fibrinogen-like protein 2  Cd74 - cd74 antigen (invariant polypeptide of major histocompatibility complex, class ii antigen-associated)  Csf1r - colony stimulating factor 1 receptor  H2-Oa - histocompatibility 2, o region alpha locus  Inhba - inhibin beta-a  Ccl3 - chemokine (c-c motif) ligand 3 |

|  |  |  |  |  | H2-M3 - histocompatibility 2, m region locus 3  H2-DMa - histocompatibility 2, class ii, locus dma  Bmi1 - bmi1 polycomb ring finger oncogene  Runx1 - runt related transcription factor 1  Csf1 - colony stimulating factor 1 (macrophage)  Ccl5 - chemokine (c-c motif) ligand 5  Egr3 - early growth response 3 |
| --- | --- | --- | --- | --- | --- |
| GO:1902533 | positive regulation of intracellular signal transduction | 3.22E-6 | 4.41E-4 | 2.54 (15721,976,184,29) | Trf - transferrin  Icam1 - intercellular adhesion molecule 1  Trem2 - triggering receptor expressed on myeloid cells 2  Fgr - gardner-rasheed feline sarcoma viral (fgr) oncogene homolog Fpr2 - formyl peptide receptor 2  Cd74 - cd74 antigen (invariant polypeptide of major histocompatibility complex, class ii antigen-associated)  Slc15a3 - solute carrier family 15, member 3  Prkcb - protein kinase c, beta  Csf1r - colony stimulating factor 1 receptor  Csf2 - colony stimulating factor 2 (granulocyte-macrophage)  Avpi1 - arginine vasopressin-induced 1  Ccl3 - chemokine (c-c motif) ligand 3  Dab2 - disabled 2, mitogen-responsive phosphoprotein  Ccl4 - chemokine (c-c motif) ligand 4  P2rx4 - purinergic receptor p2x, ligand-gated ion channel 4  Gpnmb - glycoprotein (transmembrane) nmb  Ccl5 - chemokine (c-c motif) ligand 5  Csf1 - colony stimulating factor 1 (macrophage)  Ccl2 - chemokine (c-c motif) ligand 2  Ccl17 - chemokine (c-c motif) ligand 17  Il7r - interleukin 7 receptor  Clec4n - c-type lectin domain family 4, member n  Casp1 - caspase 1  Alox15 - arachidonate 15-lipoxygenase  Mmp8 - matrix metallopeptidase 8  Zeb2 - zinc finger e-box binding homeobox 2  Tgm2 - transglutaminase 2, c polypeptide  Il1rn - interleukin 1 receptor antagonist  Nenf - neuron derived neurotrophic factor |
| GO:0070555 | response to interleukin-1 | 3.52E-6 | 4.79E-4 | 8.76 (15721,78,184,8) | Ccl2 - chemokine (c-c motif) ligand 2  Ccl17 - chemokine (c-c motif) ligand 17  Sirpa - signal-regulatory protein alpha  Ccl3 - chemokine (c-c motif) ligand 3  Ccl4 - chemokine (c-c motif) ligand 4  Irg1 - immunoresponsive gene 1  Saa3 - serum amyloid a 3  Ccl5 - chemokine (c-c motif) ligand 5 |
| GO:1990266 | neutrophil migration | 3.88E-6 | 5.23E-4 | 8.65 (15721,79,184,8) | Cxcl2 - chemokine (c-x-c motif) ligand 2  Ccl2 - chemokine (c-c motif) ligand 2  Lgals3 - lectin, galactose binding, soluble 3  Pde4b - phosphodiesterase 4b, camp specific  Ccl17 - chemokine (c-c motif) ligand 17  Ccl3 - chemokine (c-c motif) ligand 3  Ccl4 - chemokine (c-c motif) ligand 4  Ccl5 - chemokine (c-c motif) ligand 5 |
| GO:0051272 | positive regulation of cellular component movement | 4.1E-6 | 5.47E-4 | 3.11 (15721,577,184,21) | Itgax - integrin alpha x  Ccl2 - chemokine (c-c motif) ligand 2  Lgals3 - lectin, galactose binding, soluble 3  Icam1 - intercellular adhesion molecule 1  Trf - transferrin  Trem2 - triggering receptor expressed on myeloid cells 2  Cd274 - cd274 antigen  Fgr - gardner-rasheed feline sarcoma viral (fgr) oncogene homolog Thy1 - thymus cell antigen 1, theta  Fpr2 - formyl peptide receptor 2  Cd74 - cd74 antigen (invariant polypeptide of major histocompatibility complex, class ii antigen-associated)  Csf1r - colony stimulating factor 1 receptor  Mmp2 - matrix metallopeptidase 2  Clec7a - c-type lectin domain family 7, member a  Acvr1 - activin a receptor, type 1  Dab2 - disabled 2, mitogen-responsive phosphoprotein  Ccl4 - chemokine (c-c motif) ligand 4  P2rx4 - purinergic receptor p2x, ligand-gated ion channel 4  Csf1 - colony stimulating factor 1 (macrophage)  Gpnmb - glycoprotein (transmembrane) nmb |

|  |  |  |  |  | Ccl5 - chemokine (c-c motif) ligand 5 |
| --- | --- | --- | --- | --- | --- |
| GO:0009966 | regulation of signal transduction | 4.11E-6 | 5.43E-4 | 1.80 (15721,2664,184,56) | Mgll - monoglyceride lipase  Trf - transferrin  Fpr2 - formyl peptide receptor 2  Prkcb - protein kinase c, beta  Cd74 - cd74 antigen (invariant polypeptide of major histocompatibility complex, class ii antigen-associated)  Socs3 - suppressor of cytokine signaling 3  Dab2 - disabled 2, mitogen-responsive phosphoprotein  Mmp12 - matrix metallopeptidase 12  H2-M3 - histocompatibility 2, m region locus 3  P2rx4 - purinergic receptor p2x, ligand-gated ion channel 4  Gpnmb - glycoprotein (transmembrane) nmb  Neto2 - neuropilin (nrp) and tolloid (tll)-like 2  Ccl2 - chemokine (c-c motif) ligand 2  Pde4b - phosphodiesterase 4b, camp specific  Ccl17 - chemokine (c-c motif) ligand 17  Clec4n - c-type lectin domain family 4, member n  Alox15 - arachidonate 15-lipoxygenase  Fabp5 - fatty acid binding protein 5, epidermal  Mmp8 - matrix metallopeptidase 8  Padi2 - peptidyl arginine deiminase, type ii  Acvr1 - activin a receptor, type 1  Nenf - neuron derived neurotrophic factor  Lpxn - leupaxin  Tnfaip3 - tumor necrosis factor, alpha-induced protein 3  Chst11 - carbohydrate sulfotransferase 11  Ikbke - inhibitor of kappab kinase epsilon  Icam1 - intercellular adhesion molecule 1  Rnf157 - ring finger protein 157  Inpp4b - inositol polyphosphate-4-phosphatase, type ii  Trem2 - triggering receptor expressed on myeloid cells 2  Fgr - gardner-rasheed feline sarcoma viral (fgr) oncogene homolog Il1rl1 - interleukin 1 receptor-like 1  Slc15a3 - solute carrier family 15, member 3  Csf1r - colony stimulating factor 1 receptor  Csf2 - colony stimulating factor 2 (granulocyte-macrophage)  Avpi1 - arginine vasopressin-induced 1  Fam20c - family with sequence similarity 20, member c  Ccl3 - chemokine (c-c motif) ligand 3  Ccl4 - chemokine (c-c motif) ligand 4  Irg1 - immunoresponsive gene 1  Bmi1 - bmi1 polycomb ring finger oncogene  Csf1 - colony stimulating factor 1 (macrophage)  Ccl5 - chemokine (c-c motif) ligand 5  Igfbp7 - insulin-like growth factor binding protein 7  Lgals3 - lectin, galactose binding, soluble 3  Il7r - interleukin 7 receptor  Ndrg2 - n-myc downstream regulated gene 2  Sirpa - signal-regulatory protein alpha  Casp1 - caspase 1  Thy1 - thymus cell antigen 1, theta  Zeb2 - zinc finger e-box binding homeobox 2  Tgm2 - transglutaminase 2, c polypeptide  Inhba - inhibin beta-a  Kdm5d - lysine (k)-specific demethylase 5d  Il1rn - interleukin 1 receptor antagonist  Runx1 - runt related transcription factor 1 |
| GO:0050729 | positive regulation of inflammatory response | 4.49E-6 | 5.89E-4 | 6.28 (15721,136,184,10) | Tgm2 - transglutaminase 2, c polypeptide  Ccr5 - chemokine (c-c motif) receptor 5  Cebpa - ccaat/enhancer binding protein (c/ebp), alpha  Clec7a - c-type lectin domain family 7, member a  Ccl3 - chemokine (c-c motif) ligand 3  Trem2 - triggering receptor expressed on myeloid cells 2  Ccl5 - chemokine (c-c motif) ligand 5  Mmp8 - matrix metallopeptidase 8  Il1rl1 - interleukin 1 receptor-like 1  Ctss - cathepsin s |
|  |  |  |  |  | Ccl2 - chemokine (c-c motif) ligand 2  Ccl17 - chemokine (c-c motif) ligand 17  Icam1 - intercellular adhesion molecule 1  Trf - transferrin  Alox15 - arachidonate 15-lipoxygenase  Trem2 - triggering receptor expressed on myeloid cells 2  Mmp8 - matrix metallopeptidase 8 |

| GO:0043410 | positive regulation of MAPK cascade | 4.65E-6 | 6.05E-4 | 3.33 (15721,488,184,19) | Zeb2 - zinc finger e-box binding homeobox 2  Fpr2 - formyl peptide receptor 2  Cd74 - cd74 antigen (invariant polypeptide of major histocompatibility complex, class ii antigen-associated)  Csf1r - colony stimulating factor 1 receptor  Il1rn - interleukin 1 receptor antagonist  Avpi1 - arginine vasopressin-induced 1  Dab2 - disabled 2, mitogen-responsive phosphoprotein  Ccl3 - chemokine (c-c motif) ligand 3  Ccl4 - chemokine (c-c motif) ligand 4  Nenf - neuron derived neurotrophic factor  Gpnmb - glycoprotein (transmembrane) nmb  Ccl5 - chemokine (c-c motif) ligand 5 |
| --- | --- | --- | --- | --- | --- |
| GO:0070374 | positive regulation of ERK1 and ERK2 cascade | 4.86E-6 | 6.27E-4 | 5.05 (15721,203,184,12) | Ccl2 - chemokine (c-c motif) ligand 2  Cd74 - cd74 antigen (invariant polypeptide of major histocompatibility complex, class ii antigen-associated)  Csf1r - colony stimulating factor 1 receptor  Ccl17 - chemokine (c-c motif) ligand 17  Icam1 - intercellular adhesion molecule 1  Alox15 - arachidonate 15-lipoxygenase  Ccl3 - chemokine (c-c motif) ligand 3  Ccl4 - chemokine (c-c motif) ligand 4  Trem2 - triggering receptor expressed on myeloid cells 2  Gpnmb - glycoprotein (transmembrane) nmb  Ccl5 - chemokine (c-c motif) ligand 5  Fpr2 - formyl peptide receptor 2 |
| GO:0030335 | positive regulation of cell migration | 5.45E-6 | 6.97E-4 | 3.16 (15721,540,184,20) | Itgax - integrin alpha x  Ccl2 - chemokine (c-c motif) ligand 2  Lgals3 - lectin, galactose binding, soluble 3  Icam1 - intercellular adhesion molecule 1  Trem2 - triggering receptor expressed on myeloid cells 2  Cd274 - cd274 antigen  Fgr - gardner-rasheed feline sarcoma viral (fgr) oncogene homolog Thy1 - thymus cell antigen 1, theta  Fpr2 - formyl peptide receptor 2  Cd74 - cd74 antigen (invariant polypeptide of major histocompatibility complex, class ii antigen-associated)  Csf1r - colony stimulating factor 1 receptor  Mmp2 - matrix metallopeptidase 2  Clec7a - c-type lectin domain family 7, member a  Acvr1 - activin a receptor, type 1  Dab2 - disabled 2, mitogen-responsive phosphoprotein  Ccl4 - chemokine (c-c motif) ligand 4  P2rx4 - purinergic receptor p2x, ligand-gated ion channel 4  Csf1 - colony stimulating factor 1 (macrophage)  Gpnmb - glycoprotein (transmembrane) nmb  Ccl5 - chemokine (c-c motif) ligand 5 |
| GO:0040017 | positive regulation of locomotion | 5.49E-6 | 6.97E-4 | 3.05 (15721,588,184,21) | Itgax - integrin alpha x  Ccl2 - chemokine (c-c motif) ligand 2  Lgals3 - lectin, galactose binding, soluble 3  Icam1 - intercellular adhesion molecule 1  Trf - transferrin  Trem2 - triggering receptor expressed on myeloid cells 2  Cd274 - cd274 antigen  Fgr - gardner-rasheed feline sarcoma viral (fgr) oncogene homolog Thy1 - thymus cell antigen 1, theta  Fpr2 - formyl peptide receptor 2  Cd74 - cd74 antigen (invariant polypeptide of major histocompatibility complex, class ii antigen-associated)  Csf1r - colony stimulating factor 1 receptor  Mmp2 - matrix metallopeptidase 2  Clec7a - c-type lectin domain family 7, member a  Acvr1 - activin a receptor, type 1  Dab2 - disabled 2, mitogen-responsive phosphoprotein  Ccl4 - chemokine (c-c motif) ligand 4  P2rx4 - purinergic receptor p2x, ligand-gated ion channel 4  Csf1 - colony stimulating factor 1 (macrophage)  Gpnmb - glycoprotein (transmembrane) nmb  Ccl5 - chemokine (c-c motif) ligand 5 |
| GO:0002688 | regulation of leukocyte chemotaxis | 6.14E-6 | 7.73E-4 | 6.93 (15721,111,184,9) | Mpp1 - membrane protein, palmitoylated  Ccl2 - chemokine (c-c motif) ligand 2  Cd74 - cd74 antigen (invariant polypeptide of major histocompatibility complex, class ii antigen-associated)  Csf1r - colony stimulating factor 1 receptor  Padi2 - peptidyl arginine deiminase, type ii |

|  |  |  |  |  | Ccl4 - chemokine (c-c motif) ligand 4  Ccl5 - chemokine (c-c motif) ligand 5  Csf1 - colony stimulating factor 1 (macrophage)  Fpr2 - formyl peptide receptor 2 |
| --- | --- | --- | --- | --- | --- |
| GO:0006909 | phagocytosis | 6.17E-6 | 7.71E-4 | 8.14 (15721,84,184,8) | Tgm2 - transglutaminase 2, c polypeptide  Alox15 - arachidonate 15-lipoxygenase  Myo7a - myosin viia  Trem2 - triggering receptor expressed on myeloid cells 2  Slc11a1 - solute carrier family 11 (proton-coupled divalent metal ion transporters), member 1  Pld4 - phospholipase d family, member 4  Mfge8 - milk fat globule-egf factor 8 protein  Hck - hemopoietic cell kinase |
| GO:0050793 | regulation of developmental process | 6.78E-6 | 8.39E-4 | 1.79 (15721,2571,184,54) | Mgll - monoglyceride lipase  Trf - transferrin  Akap11 - a kinase (prka) anchor protein 11  Prkcb - protein kinase c, beta  Cd74 - cd74 antigen (invariant polypeptide of major histocompatibility complex, class ii antigen-associated)  H2-Oa - histocompatibility 2, o region alpha locus  Socs3 - suppressor of cytokine signaling 3  Junb - jun-b oncogene  Mmp12 - matrix metallopeptidase 12  Dab2 - disabled 2, mitogen-responsive phosphoprotein  H2-M3 - histocompatibility 2, m region locus 3  P2rx4 - purinergic receptor p2x, ligand-gated ion channel 4  H2-DMa - histocompatibility 2, class ii, locus dma  Ccl2 - chemokine (c-c motif) ligand 2  Itgax - integrin alpha x  Ccl17 - chemokine (c-c motif) ligand 17  Tnfrsf1b - tumor necrosis factor receptor superfamily, member 1b Fmnl2 - formin-like 2  Cd34 - cd34 antigen  Plxnc1 - plexin c1  Acvr1 - activin a receptor, type 1  Parvb - parvin, beta  Tnfaip3 - tumor necrosis factor, alpha-induced protein 3  Zhx2 - zinc fingers and homeoboxes 2  Cebpa - ccaat/enhancer binding protein (c/ebp), alpha  Icam1 - intercellular adhesion molecule 1  Rnf157 - ring finger protein 157  Inpp4b - inositol polyphosphate-4-phosphatase, type ii  Trem2 - triggering receptor expressed on myeloid cells 2  Cd274 - cd274 antigen  Cst7 - cystatin f (leukocystatin)  Fgr - gardner-rasheed feline sarcoma viral (fgr) oncogene homolog Fgl2 - fibrinogen-like protein 2  Hck - hemopoietic cell kinase  Csf1r - colony stimulating factor 1 receptor  Csf2 - colony stimulating factor 2 (granulocyte-macrophage) Fam20c - family with sequence similarity 20, member c  Ccl3 - chemokine (c-c motif) ligand 3  Bmi1 - bmi1 polycomb ring finger oncogene  Csf1 - colony stimulating factor 1 (macrophage)  Ccl5 - chemokine (c-c motif) ligand 5  Lgals3 - lectin, galactose binding, soluble 3  Ccr5 - chemokine (c-c motif) receptor 5  H2-D1 - histocompatibility 2, d region locus 1  Il7r - interleukin 7 receptor  H2-Aa - histocompatibility 2, class ii antigen a, alpha  Thy1 - thymus cell antigen 1, theta  H2-K1 - histocompatibility 2, k1, k region  Zeb2 - zinc finger e-box binding homeobox 2  Inhba - inhibin beta-a  Il1rn - interleukin 1 receptor antagonist  Runx1 - runt related transcription factor 1  Egr3 - early growth response 3  Egr2 - early growth response 2 |
|  |  |  |  |  | Cebpa - ccaat/enhancer binding protein (c/ebp), alpha  Trf - transferrin  Rnf157 - ring finger protein 157  Trem2 - triggering receptor expressed on myeloid cells 2  Cd274 - cd274 antigen  Cst7 - cystatin f (leukocystatin)  Akap11 - a kinase (prka) anchor protein 11  Cd74 - cd74 antigen (invariant polypeptide of major histocompatibility complex, class ii antigen-associated)  Prkcb - protein kinase c, beta |

| GO:0051094 | positive regulation of developmental process | 7.32E-6 | 8.99E-4 | 2.13 (15721,1483,184,37) | Csf1r - colony stimulating factor 1 receptor  Csf2 - colony stimulating factor 2 (granulocyte-macrophage)  Socs3 - suppressor of cytokine signaling 3  Fam20c - family with sequence similarity 20, member c  Junb - jun-b oncogene  Ccl3 - chemokine (c-c motif) ligand 3  Dab2 - disabled 2, mitogen-responsive phosphoprotein  H2-M3 - histocompatibility 2, m region locus 3  P2rx4 - purinergic receptor p2x, ligand-gated ion channel 4  Bmi1 - bmi1 polycomb ring finger oncogene  H2-DMa - histocompatibility 2, class ii, locus dma  Ccl5 - chemokine (c-c motif) ligand 5  Csf1 - colony stimulating factor 1 (macrophage)  Itgax - integrin alpha x  Lgals3 - lectin, galactose binding, soluble 3  Ccr5 - chemokine (c-c motif) receptor 5  Il7r - interleukin 7 receptor  Tnfrsf1b - tumor necrosis factor receptor superfamily, member 1b H2-Aa - histocompatibility 2, class ii antigen a, alpha  Zeb2 - zinc finger e-box binding homeobox 2  Cd34 - cd34 antigen  Inhba - inhibin beta-a  Plxnc1 - plexin c1  Acvr1 - activin a receptor, type 1  Runx1 - runt related transcription factor 1  Tnfaip3 - tumor necrosis factor, alpha-induced protein 3  Egr3 - early growth response 3  Egr2 - early growth response 2 |
| --- | --- | --- | --- | --- | --- |
| GO:0150077 | regulation of neuro-inflammatory response | 7.38E-6 | 8.99E-4 | 17.80 (15721,24,184,5) | Cd200r3 - cd200 receptor 3  Tnfrsf1b - tumor necrosis factor receptor superfamily, member 1b Trem2 - triggering receptor expressed on myeloid cells 2  Cst7 - cystatin f (leukocystatin)  Mmp8 - matrix metallopeptidase 8 |
| GO:0016477 | cell migration | 7.82E-6 | 9.45E-4 | 2.65 (15721,805,184,25) | Icam1 - intercellular adhesion molecule 1  Cxcl2 - chemokine (c-x-c motif) ligand 2  Cd9 - cd9 antigen  Mmp2 - matrix metallopeptidase 2  Ccl3 - chemokine (c-c motif) ligand 3  Mmp12 - matrix metallopeptidase 12  Ccl4 - chemokine (c-c motif) ligand 4  Saa3 - serum amyloid a 3  Ccl5 - chemokine (c-c motif) ligand 5  Ccl2 - chemokine (c-c motif) ligand 2  Ccl17 - chemokine (c-c motif) ligand 17  Ccr5 - chemokine (c-c motif) receptor 5  Lgals3 - lectin, galactose binding, soluble 3  Pde4b - phosphodiesterase 4b, camp specific  Sirpa - signal-regulatory protein alpha  Fmnl2 - formin-like 2  Epx - eosinophil peroxidase  Atrnl1 - attractin like 1  Zeb2 - zinc finger e-box binding homeobox 2  Cd34 - cd34 antigen  Acvr1 - activin a receptor, type 1  Ccrl2 - chemokine (c-c motif) receptor-like 2  Lpxn - leupaxin  Egr3 - early growth response 3  Tnfaip3 - tumor necrosis factor, alpha-induced protein 3 |
| GO:0050868 | negative regulation of T cell activation | 8.21E-6 | 9.84E-4 | 6.69 (15721,115,184,9) | Cd74 - cd74 antigen (invariant polypeptide of major histocompatibility complex, class ii antigen-associated)  Lgals3 - lectin, galactose binding, soluble 3  H2-Ab1 - histocompatibility 2, class ii antigen a, beta 1  H2-Aa - histocompatibility 2, class ii antigen a, alpha  H2-M3 - histocompatibility 2, m region locus 3  Cd274 - cd274 antigen  Fgl2 - fibrinogen-like protein 2  Runx1 - runt related transcription factor 1  Gpnmb - glycoprotein (transmembrane) nmb |
| GO:0002763 | positive regulation of myeloid leukocyte differentiation | 9.5E-6 | 1.13E-3 | 9.35 (15721,64,184,7) | Cd74 - cd74 antigen (invariant polypeptide of major histocompatibility complex, class ii antigen-associated)  Csf1r - colony stimulating factor 1 receptor  Ccl3 - chemokine (c-c motif) ligand 3  Trem2 - triggering receptor expressed on myeloid cells 2 |

|  |  |  |  |  | Runx1 - runt related transcription factor 1  Ccl5 - chemokine (c-c motif) ligand 5  Csf1 - colony stimulating factor 1 (macrophage) |
| --- | --- | --- | --- | --- | --- |
| GO:0042742 | defense response to bacterium | 1.05E-5 | 1.24E-3 | 4.68 (15721,219,184,12) | Il7r - interleukin 7 receptor  Trf - transferrin  Epx - eosinophil peroxidase  Wfdc17 - wap four-disulfide core domain 17  H2-M3 - histocompatibility 2, m region locus 3  Trem2 - triggering receptor expressed on myeloid cells 2  Fgr - gardner-rasheed feline sarcoma viral (fgr) oncogene homolog Slc11a1 - solute carrier family 11 (proton-coupled divalent metal ion transporters), member 1  H2-K1 - histocompatibility 2, k1, k region  Prg2 - proteoglycan 2, bone marrow  Fpr2 - formyl peptide receptor 2  Hck - hemopoietic cell kinase |
| GO:0045580 | regulation of T cell differentiation | 1.07E-5 | 1.26E-3 | 5.70 (15721,150,184,10) | Cd74 - cd74 antigen (invariant polypeptide of major histocompatibility complex, class ii antigen-associated)  H2-Oa - histocompatibility 2, o region alpha locus  Il7r - interleukin 7 receptor  H2-Aa - histocompatibility 2, class ii antigen a, alpha  H2-M3 - histocompatibility 2, m region locus 3  Bmi1 - bmi1 polycomb ring finger oncogene  Fgl2 - fibrinogen-like protein 2  H2-DMa - histocompatibility 2, class ii, locus dma  Runx1 - runt related transcription factor 1  Egr3 - early growth response 3 |
| GO:0030100 | regulation of endocytosis | 1.08E-5 | 1.25E-3 | 4.03 (15721,297,184,14) | Ccl2 - chemokine (c-c motif) ligand 2  Lgals3 - lectin, galactose binding, soluble 3  Sirpa - signal-regulatory protein alpha  Trf - transferrin  Alox15 - arachidonate 15-lipoxygenase  Siglec5 - sialic acid binding ig-like lectin 5  Trem2 - triggering receptor expressed on myeloid cells 2  Fgr - gardner-rasheed feline sarcoma viral (fgr) oncogene homolog Slc11a1 - solute carrier family 11 (proton-coupled divalent metal ion transporters), member 1  Hck - hemopoietic cell kinase  Fpr2 - formyl peptide receptor 2  Clec7a - c-type lectin domain family 7, member a  Dab2 - disabled 2, mitogen-responsive phosphoprotein  Mfge8 - milk fat globule-egf factor 8 protein |
| GO:0040011 | locomotion | 1.09E-5 | 1.26E-3 | 2.43 (15721,984,184,28) | Icam1 - intercellular adhesion molecule 1  Fpr1 - formyl peptide receptor 1  Fpr2 - formyl peptide receptor 2  Cxcl2 - chemokine (c-x-c motif) ligand 2  Cd9 - cd9 antigen  Mmp2 - matrix metallopeptidase 2  Ccl3 - chemokine (c-c motif) ligand 3  Mmp12 - matrix metallopeptidase 12  Ccl4 - chemokine (c-c motif) ligand 4  Saa3 - serum amyloid a 3  Ccl5 - chemokine (c-c motif) ligand 5  Ccl2 - chemokine (c-c motif) ligand 2  Ear2 - eosinophil-associated, ribonuclease a family, member 2 Pde4b - phosphodiesterase 4b, camp specific  Ccl17 - chemokine (c-c motif) ligand 17  Ccr5 - chemokine (c-c motif) receptor 5  Lgals3 - lectin, galactose binding, soluble 3  Sirpa - signal-regulatory protein alpha  Fmnl2 - formin-like 2  Epx - eosinophil peroxidase  Atrnl1 - attractin like 1  Zeb2 - zinc finger e-box binding homeobox 2  Cd34 - cd34 antigen  Acvr1 - activin a receptor, type 1  Ccrl2 - chemokine (c-c motif) receptor-like 2  Lpxn - leupaxin  Tnfaip3 - tumor necrosis factor, alpha-induced protein 3  Egr3 - early growth response 3 |
|  |  |  |  |  | Csf1r - colony stimulating factor 1 receptor  Csf2 - colony stimulating factor 2 (granulocyte-macrophage) |

| GO:0030099 | myeloid cell differentiation | 1.28E-5 | 1.46E-3 | 5.03 (15721,187,184,11) | Cebpa - ccaat/enhancer binding protein (c/ebp), alpha  Trf - transferrin  Snx10 - sorting nexin 10  Junb - jun-b oncogene  Dab2 - disabled 2, mitogen-responsive phosphoprotein  Il3ra - interleukin 3 receptor, alpha chain  Trem2 - triggering receptor expressed on myeloid cells 2  Runx1 - runt related transcription factor 1  Csf1 - colony stimulating factor 1 (macrophage) |
| --- | --- | --- | --- | --- | --- |
| GO:0022603 | regulation of anatomical structure morphogenesis | 1.29E-5 | 1.47E-3 | 2.36 (15721,1049,184,29) | Mgll - monoglyceride lipase  Icam1 - intercellular adhesion molecule 1  Rnf157 - ring finger protein 157  Trem2 - triggering receptor expressed on myeloid cells 2  Fgr - gardner-rasheed feline sarcoma viral (fgr) oncogene homolog Hck - hemopoietic cell kinase  Prkcb - protein kinase c, beta  Csf1r - colony stimulating factor 1 receptor  Ccl3 - chemokine (c-c motif) ligand 3  Dab2 - disabled 2, mitogen-responsive phosphoprotein  H2-M3 - histocompatibility 2, m region locus 3  H2-DMa - histocompatibility 2, class ii, locus dma  Ccl5 - chemokine (c-c motif) ligand 5  Csf1 - colony stimulating factor 1 (macrophage)  Ccl2 - chemokine (c-c motif) ligand 2  Itgax - integrin alpha x  Ccr5 - chemokine (c-c motif) receptor 5  Lgals3 - lectin, galactose binding, soluble 3  Tnfrsf1b - tumor necrosis factor receptor superfamily, member 1b Fmnl2 - formin-like 2  Thy1 - thymus cell antigen 1, theta  Zeb2 - zinc finger e-box binding homeobox 2  Cd34 - cd34 antigen  Il1rn - interleukin 1 receptor antagonist  Plxnc1 - plexin c1  Acvr1 - activin a receptor, type 1  Parvb - parvin, beta  Runx1 - runt related transcription factor 1  Tnfaip3 - tumor necrosis factor, alpha-induced protein 3 |
| GO:0001961 | positive regulation of cytokine-mediated signaling pathway | 1.5E-5 | 1.7E-3 | 11.14 (15721,46,184,6) | Cd74 - cd74 antigen (invariant polypeptide of major histocompatibility complex, class ii antigen-associated)  Ikbke - inhibitor of kappab kinase epsilon  Casp1 - caspase 1  Mmp12 - matrix metallopeptidase 12  Trem2 - triggering receptor expressed on myeloid cells 2  Csf1 - colony stimulating factor 1 (macrophage) |
| GO:0051716 | cellular response to stimulus | 1.54E-5 | 1.73E-3 | 1.79 (15721,2439,184,51) | Dsc2 - desmocollin 2  Crip1 - cysteine-rich protein 1 (intestinal)  Zmat3 - zinc finger matrin type 3  Trf - transferrin  Ifi202b - interferon activated gene 202b  Fpr2 - formyl peptide receptor 2  Prkcb - protein kinase c, beta  Cd9 - cd9 antigen  Mmp2 - matrix metallopeptidase 2  Socs3 - suppressor of cytokine signaling 3  Snx10 - sorting nexin 10  Junb - jun-b oncogene  Dab2 - disabled 2, mitogen-responsive phosphoprotein  Gch1 - gtp cyclohydrolase 1  Saa3 - serum amyloid a 3  P2rx4 - purinergic receptor p2x, ligand-gated ion channel 4  Ccl2 - chemokine (c-c motif) ligand 2  Ccl17 - chemokine (c-c motif) ligand 17  Pde4b - phosphodiesterase 4b, camp specific  Tnfrsf1b - tumor necrosis factor receptor superfamily, member 1b Alox15 - arachidonate 15-lipoxygenase  Ifi205 - interferon activated gene 205  Padi2 - peptidyl arginine deiminase, type ii  Mcts1 - malignant t cell amplified sequence 1  Acvr1 - activin a receptor, type 1  Tnfaip3 - tumor necrosis factor, alpha-induced protein 3  Mrc1 - mannose receptor, c type 1  Ikbke - inhibitor of kappab kinase epsilon  Cebpa - ccaat/enhancer binding protein (c/ebp), alpha  Icam1 - intercellular adhesion molecule 1  Trem2 - triggering receptor expressed on myeloid cells 2 |

|  |  |  |  |  | Cd274 - cd274 antigen  Cxcl2 - chemokine (c-x-c motif) ligand 2  Csf1r - colony stimulating factor 1 receptor  Csf2ra - colony stimulating factor 2 receptor, alpha, low-affinity (granulocyte-macrophage)  Ifi203 - interferon activated gene 203  Csf2 - colony stimulating factor 2 (granulocyte-macrophage)  Evl - ena-vasodilator stimulated phosphoprotein  Clec7a - c-type lectin domain family 7, member a  Ccl3 - chemokine (c-c motif) ligand 3  Ccl4 - chemokine (c-c motif) ligand 4  Irg1 - immunoresponsive gene 1  Ccl5 - chemokine (c-c motif) ligand 5  H2-Ab1 - histocompatibility 2, class ii antigen a, beta 1  Sirpa - signal-regulatory protein alpha  Casp1 - caspase 1  Cpne7 - copine vii Inhba - inhibin beta-a  Runx1 - runt related transcription factor 1  Egr3 - early growth response 3  Egr2 - early growth response 2 |
| --- | --- | --- | --- | --- | --- |
| GO:0033993 | response to lipid | 1.66E-5 | 1.85E-3 | 3.04 (15721,534,184,19) | Ccl2 - chemokine (c-c motif) ligand 2  Ccr5 - chemokine (c-c motif) receptor 5  Pde4b - phosphodiesterase 4b, camp specific  Mrc1 - mannose receptor, c type 1  Tnfrsf1b - tumor necrosis factor receptor superfamily, member 1b Sirpa - signal-regulatory protein alpha  Casp1 - caspase 1  Trem2 - triggering receptor expressed on myeloid cells 2  Cd274 - cd274 antigen  Slc11a1 - solute carrier family 11 (proton-coupled divalent metal ion transporters), member 1  Cxcl2 - chemokine (c-x-c motif) ligand 2  Mmp2 - matrix metallopeptidase 2  Inhba - inhibin beta-a  Il1rn - interleukin 1 receptor antagonist  Dab2 - disabled 2, mitogen-responsive phosphoprotein  Gch1 - gtp cyclohydrolase 1  Irg1 - immunoresponsive gene 1  Runx1 - runt related transcription factor 1  Tnfaip3 - tumor necrosis factor, alpha-induced protein 3 |
|  |  |  |  |  | Dsc2 - desmocollin 2  Decr1 - 2,4-dienoyl coa reductase 1, mitochondrial  Akap11 - a kinase (prka) anchor protein 11  Prkcb - protein kinase c, beta  H2-Oa - histocompatibility 2, o region alpha locus  Socs3 - suppressor of cytokine signaling 3  Hivep3 - human immunodeficiency virus type i enhancer binding protein 3  Dab2 - disabled 2, mitogen-responsive phosphoprotein  H2-M3 - histocompatibility 2, m region locus 3  Gch1 - gtp cyclohydrolase 1  Dach1 - dachshund 1 (drosophila)  H2-DMa - histocompatibility 2, class ii, locus dma  Slc2a6 - solute carrier family 2 (facilitated glucose transporter), member 6  Neto2 - neuropilin (nrp) and tolloid (tll)-like 2  Pde4b - phosphodiesterase 4b, camp specific  Fabp5 - fatty acid binding protein 5, epidermal  Sestd1 - sec14 and spectrin domains 1  Slc11a1 - solute carrier family 11 (proton-coupled divalent metal ion transporters), member 1  Padi2 - peptidyl arginine deiminase, type ii  Mcts1 - malignant t cell amplified sequence 1  Plxnc1 - plexin c1  Acvr1 - activin a receptor, type 1  Parvb - parvin, beta  Lpxn - leupaxin  Pilra - paired immunoglobin-like type 2 receptor alpha  Zhx2 - zinc fingers and homeoboxes 2  Cyp4f18 - cytochrome p450, family 4, subfamily f, polypeptide 18 Ikbke - inhibitor of kappab kinase epsilon  Fam46a - family with sequence similarity 46, member a  Icam1 - intercellular adhesion molecule 1  Ctsb - cathepsin b  Inpp4b - inositol polyphosphate-4-phosphatase, type ii  Trem2 - triggering receptor expressed on myeloid cells 2  Cd274 - cd274 antigen  Cst7 - cystatin f (leukocystatin)  Emr4 - egf-like module containing, mucin-like, hormone receptor-like sequence 4 |

| GO:0050789 | regulation of biological process | 1.68E-5 | 1.86E-3 | 1.25 (15721,9380,184,137) | Il1rl1 - interleukin 1 receptor-like 1  Ifi30 - interferon gamma inducible protein 30  Hck - hemopoietic cell kinase  Slc15a3 - solute carrier family 15, member 3  Ifi203 - interferon activated gene 203  Evl - ena-vasodilator stimulated phosphoprotein  Irg1 - immunoresponsive gene 1  Ctss - cathepsin s  Ccr5 - chemokine (c-c motif) receptor 5  Paox - polyamine oxidase (exo-n4-amino)  Casp1 - caspase 1  Sirpa - signal-regulatory protein alpha  Ifi27 - interferon, alpha-inducible protein 27  Epx - eosinophil peroxidase  Uty - ubiquitously transcribed tetratricopeptide repeat gene, y chromosome  Inhba - inhibin beta-a  Serpinb2 - serine (or cysteine) peptidase inhibitor, clade b, member 2  Pld3 - phospholipase d family, member 3  Runx1 - runt related transcription factor 1  Nfkbie - nuclear factor of kappa light polypeptide gene enhancer in b cells inhibitor, epsilon  Crip1 - cysteine-rich protein 1 (intestinal)  Mgll - monoglyceride lipase  Zmat3 - zinc finger matrin type 3  Mtmr9 - myotubularin related protein 9  Trf - transferrin  Acot1 - acyl-coa thioesterase 1  Fpr1 - formyl peptide receptor 1  Fpr2 - formyl peptide receptor 2  Eif2s3y - eukaryotic translation initiation factor 2, subunit 3, structural gene y-linked  Cd74 - cd74 antigen (invariant polypeptide of major histocompatibility complex, class ii antigen-associated)  Cd9 - cd9 antigen  Mmp2 - matrix metallopeptidase 2  Mmp13 - matrix metallopeptidase 13  Junb - jun-b oncogene  Mmp12 - matrix metallopeptidase 12  P2rx4 - purinergic receptor p2x, ligand-gated ion channel 4  Gpnmb - glycoprotein (transmembrane) nmb  Itgax - integrin alpha x  Ccl2 - chemokine (c-c motif) ligand 2  Ccl17 - chemokine (c-c motif) ligand 17  Tnfrsf1b - tumor necrosis factor receptor superfamily, member 1b Clec4n - c-type lectin domain family 4, member n  Fmnl2 - formin-like 2  Alox15 - arachidonate 15-lipoxygenase  Tnfrsf9 - tumor necrosis factor receptor superfamily, member 9 Mmp8 - matrix metallopeptidase 8  Actr3b - arp3 actin-related protein 3b  Ifi205 - interferon activated gene 205  Mpp1 - membrane protein, palmitoylated  Cd34 - cd34 antigen  P2ry14 - purinergic receptor p2y, g-protein coupled, 14  Ngfrap1 - nerve growth factor receptor (tnfrsf16) associated protein 1 Ccrl2 - chemokine (c-c motif) receptor-like 2  Stxbp6 - syntaxin binding protein 6 (amisyn)  Nenf - neuron derived neurotrophic factor  Pld4 - phospholipase d family, member 4  Tnfaip3 - tumor necrosis factor, alpha-induced protein 3  Rnf128 - ring finger protein 128  Chst11 - carbohydrate sulfotransferase 11  AF251705 - cdna sequence af251705  Cebpa - ccaat/enhancer binding protein (c/ebp), alpha  Rnf157 - ring finger protein 157  Acsl4 - acyl-coa synthetase long-chain family member 4  Siglec5 - sialic acid binding ig-like lectin 5  Fgr - gardner-rasheed feline sarcoma viral (fgr) oncogene homolog Fgl2 - fibrinogen-like protein 2  Ppap2c - phosphatidic acid phosphatase type 2c  Cxcl2 - chemokine (c-x-c motif) ligand 2  Csf1r - colony stimulating factor 1 receptor  Csf2ra - colony stimulating factor 2 receptor, alpha, low-affinity (granulocyte-macrophage)  Csf2 - colony stimulating factor 2 (granulocyte-macrophage)  Avpi1 - arginine vasopressin-induced 1  Clec7a - c-type lectin domain family 7, member a  Fam20c - family with sequence similarity 20, member c  Ccl3 - chemokine (c-c motif) ligand 3  Ccl4 - chemokine (c-c motif) ligand 4  Degs1 - degenerative spermatocyte homolog 1 (drosophila) |
| --- | --- | --- | --- | --- | --- |

|  |  |  |  |  | Bmi1 - bmi1 polycomb ring finger oncogene  Csf1 - colony stimulating factor 1 (macrophage)  Ccl5 - chemokine (c-c motif) ligand 5  Procr - protein c receptor, endothelial  Igfbp7 - insulin-like growth factor binding protein 7  H2-D1 - histocompatibility 2, d region locus 1  Cd200r3 - cd200 receptor 3  Lgals3 - lectin, galactose binding, soluble 3  Il7r - interleukin 7 receptor  Ndrg2 - n-myc downstream regulated gene 2  H2-Ab1 - histocompatibility 2, class ii antigen a, beta 1  H2-Aa - histocompatibility 2, class ii antigen a, alpha  Atrnl1 - attractin like 1  Thy1 - thymus cell antigen 1, theta  H2-K1 - histocompatibility 2, k1, k region  Zeb2 - zinc finger e-box binding homeobox 2  Tgm2 - transglutaminase 2, c polypeptide  Kdm5d - lysine (k)-specific demethylase 5d  Il1rn - interleukin 1 receptor antagonist  Il3ra - interleukin 3 receptor, alpha chain  Egr3 - early growth response 3  Egr2 - early growth response 2  Mfge8 - milk fat globule-egf factor 8 protein  Prg2 - proteoglycan 2, bone marrow |
| --- | --- | --- | --- | --- | --- |
| GO:0032680 | regulation of tumor necrosis factor production | 1.69E-5 | 1.85E-3 | 5.41 (15721,158,184,10) | Ccl2 - chemokine (c-c motif) ligand 2  Cd34 - cd34 antigen  Ccr5 - chemokine (c-c motif) receptor 5  Sirpa - signal-regulatory protein alpha  Ccl3 - chemokine (c-c motif) ligand 3  Ccl4 - chemokine (c-c motif) ligand 4  Trem2 - triggering receptor expressed on myeloid cells 2  Tnfaip3 - tumor necrosis factor, alpha-induced protein 3  Mmp8 - matrix metallopeptidase 8  Gpnmb - glycoprotein (transmembrane) nmb |
| GO:1903038 | negative regulation of leukocyte cell-cell adhesion | 1.72E-5 | 1.87E-3 | 6.10 (15721,126,184,9) | Cd74 - cd74 antigen (invariant polypeptide of major histocompatibility complex, class ii antigen-associated)  Lgals3 - lectin, galactose binding, soluble 3  H2-Ab1 - histocompatibility 2, class ii antigen a, beta 1  H2-Aa - histocompatibility 2, class ii antigen a, alpha  H2-M3 - histocompatibility 2, m region locus 3  Cd274 - cd274 antigen  Fgl2 - fibrinogen-like protein 2  Runx1 - runt related transcription factor 1  Gpnmb - glycoprotein (transmembrane) nmb |
| GO:0032651 | regulation of interleukin-1 beta production | 1.73E-5 | 1.87E-3 | 8.54 (15721,70,184,7) | Ifi205 - interferon activated gene 205  Ccr5 - chemokine (c-c motif) receptor 5  Casp1 - caspase 1  Sirpa - signal-regulatory protein alpha  Ccl3 - chemokine (c-c motif) ligand 3  Trem2 - triggering receptor expressed on myeloid cells 2  Tnfaip3 - tumor necrosis factor, alpha-induced protein 3 |
|  |  |  |  |  | Dsc2 - desmocollin 2 Trf - transferrin  Fpr2 - formyl peptide receptor 2  Prkcb - protein kinase c, beta  Cd74 - cd74 antigen (invariant polypeptide of major histocompatibility complex, class ii antigen-associated)  Cd9 - cd9 antigen  Mmp2 - matrix metallopeptidase 2  Mmp13 - matrix metallopeptidase 13  Dab2 - disabled 2, mitogen-responsive phosphoprotein  Mmp12 - matrix metallopeptidase 12  Dach1 - dachshund 1 (drosophila)  P2rx4 - purinergic receptor p2x, ligand-gated ion channel 4  Gpnmb - glycoprotein (transmembrane) nmb  Neto2 - neuropilin (nrp) and tolloid (tll)-like 2  Ccl2 - chemokine (c-c motif) ligand 2  Itgax - integrin alpha x  Pde4b - phosphodiesterase 4b, camp specific  Tnfrsf1b - tumor necrosis factor receptor superfamily, member 1b Alox15 - arachidonate 15-lipoxygenase  Fabp5 - fatty acid binding protein 5, epidermal  Slc11a1 - solute carrier family 11 (proton-coupled divalent metal ion transporters), member 1  Sestd1 - sec14 and spectrin domains 1 |

| GO:0032879 | regulation of localization | 1.79E-5 | 1.92E-3 | 1.74 (15721,2657,184,54) | Mpp1 - membrane protein, palmitoylated  Cd34 - cd34 antigen  Padi2 - peptidyl arginine deiminase, type ii  Plxnc1 - plexin c1  Acvr1 - activin a receptor, type 1  Stxbp6 - syntaxin binding protein 6 (amisyn)  Cyp4f18 - cytochrome p450, family 4, subfamily f, polypeptide 18 Ikbke - inhibitor of kappab kinase epsilon  Icam1 - intercellular adhesion molecule 1  Acsl4 - acyl-coa synthetase long-chain family member 4  Inpp4b - inositol polyphosphate-4-phosphatase, type ii  Siglec5 - sialic acid binding ig-like lectin 5  Trem2 - triggering receptor expressed on myeloid cells 2  Cd274 - cd274 antigen  Fgr - gardner-rasheed feline sarcoma viral (fgr) oncogene homolog Hck - hemopoietic cell kinase  Csf1r - colony stimulating factor 1 receptor  Evl - ena-vasodilator stimulated phosphoprotein  Clec7a - c-type lectin domain family 7, member a  Ccl4 - chemokine (c-c motif) ligand 4  Csf1 - colony stimulating factor 1 (macrophage)  Ccl5 - chemokine (c-c motif) ligand 5  Ctss - cathepsin s  Ccr5 - chemokine (c-c motif) receptor 5  Lgals3 - lectin, galactose binding, soluble 3  Sirpa - signal-regulatory protein alpha  Ifi27 - interferon, alpha-inducible protein 27  Thy1 - thymus cell antigen 1, theta  Inhba - inhibin beta-a  Il1rn - interleukin 1 receptor antagonist  Runx1 - runt related transcription factor 1  Mfge8 - milk fat globule-egf factor 8 protein |
| --- | --- | --- | --- | --- | --- |
| GO:0060548 | negative regulation of cell death | 1.8E-5 | 1.92E-3 | 2.37 (15721,1011,184,28) | Chst11 - carbohydrate sulfotransferase 11  Icam1 - intercellular adhesion molecule 1  Ctsb - cathepsin b  Rnf157 - ring finger protein 157  Acot1 - acyl-coa thioesterase 1  Trem2 - triggering receptor expressed on myeloid cells 2  Hck - hemopoietic cell kinase  Fpr2 - formyl peptide receptor 2  Cd74 - cd74 antigen (invariant polypeptide of major histocompatibility complex, class ii antigen-associated)  Csf1r - colony stimulating factor 1 receptor  Csf2 - colony stimulating factor 2 (granulocyte-macrophage)  Socs3 - suppressor of cytokine signaling 3  Dab2 - disabled 2, mitogen-responsive phosphoprotein  Bmi1 - bmi1 polycomb ring finger oncogene  Gpnmb - glycoprotein (transmembrane) nmb  Ccl5 - chemokine (c-c motif) ligand 5  Csf1 - colony stimulating factor 1 (macrophage)  Ccr5 - chemokine (c-c motif) receptor 5  Lgals3 - lectin, galactose binding, soluble 3  Il7r - interleukin 7 receptor  Tnfrsf1b - tumor necrosis factor receptor superfamily, member 1b Cd34 - cd34 antigen  Il1rn - interleukin 1 receptor antagonist  Serpinb2 - serine (or cysteine) peptidase inhibitor, clade b, member 2  Acvr1 - activin a receptor, type 1  Nenf - neuron derived neurotrophic factor  Tnfaip3 - tumor necrosis factor, alpha-induced protein 3  Egr3 - early growth response 3 |
| GO:0045582 | positive regulation of T cell differentiation | 1.8E-5 | 1.9E-3 | 7.05 (15721,97,184,8) | Cd74 - cd74 antigen (invariant polypeptide of major histocompatibility complex, class ii antigen-associated)  Il7r - interleukin 7 receptor  H2-Aa - histocompatibility 2, class ii antigen a, alpha  H2-M3 - histocompatibility 2, m region locus 3  Bmi1 - bmi1 polycomb ring finger oncogene  H2-DMa - histocompatibility 2, class ii, locus dma  Runx1 - runt related transcription factor 1  Egr3 - early growth response 3 |
| GO:0045639 | positive regulation of myeloid cell differentiation | 1.8E-5 | 1.89E-3 | 7.05 (15721,97,184,8) | Cd74 - cd74 antigen (invariant polypeptide of major histocompatibility complex, class ii antigen-associated)  Csf1r - colony stimulating factor 1 receptor  Inhba - inhibin beta-a  Ccl3 - chemokine (c-c motif) ligand 3  Trem2 - triggering receptor expressed on myeloid cells 2 |

|  |  |  |  |  | Runx1 - runt related transcription factor 1  Csf1 - colony stimulating factor 1 (macrophage)  Ccl5 - chemokine (c-c motif) ligand 5 |
| --- | --- | --- | --- | --- | --- |
| GO:0098542 | defense response to other organism | 1.9E-5 | 1.98E-3 | 3.43 (15721,399,184,16) | Itgax - integrin alpha x  Il7r - interleukin 7 receptor  Clec4n - c-type lectin domain family 4, member n  Trf - transferrin  Epx - eosinophil peroxidase  Wfdc17 - wap four-disulfide core domain 17  Trem2 - triggering receptor expressed on myeloid cells 2  Fgr - gardner-rasheed feline sarcoma viral (fgr) oncogene homolog Slc11a1 - solute carrier family 11 (proton-coupled divalent metal ion transporters), member 1  H2-K1 - histocompatibility 2, k1, k region  Hck - hemopoietic cell kinase  Fpr2 - formyl peptide receptor 2  Polr3k - polymerase (rna) iii (dna directed) polypeptide k  H2-M3 - histocompatibility 2, m region locus 3  Irg1 - immunoresponsive gene 1  Prg2 - proteoglycan 2, bone marrow |
| GO:0071622 | regulation of granulocyte chemotaxis | 1.93E-5 | 2E-3 | 10.68 (15721,48,184,6) | Mpp1 - membrane protein, palmitoylated  Ccl2 - chemokine (c-c motif) ligand 2  Cd74 - cd74 antigen (invariant polypeptide of major histocompatibility complex, class ii antigen-associated)  Csf1r - colony stimulating factor 1 receptor  Csf1 - colony stimulating factor 1 (macrophage)  Ccl5 - chemokine (c-c motif) ligand 5 |
| GO:0045088 | regulation of innate immune response | 2.02E-5 | 2.08E-3 | 4.07 (15721,273,184,13) | Ikbke - inhibitor of kappab kinase epsilon  Trem2 - triggering receptor expressed on myeloid cells 2  Fgr - gardner-rasheed feline sarcoma viral (fgr) oncogene homolog Fpr2 - formyl peptide receptor 2  Slc15a3 - solute carrier family 15, member 3  Cd74 - cd74 antigen (invariant polypeptide of major histocompatibility complex, class ii antigen-associated)  Ifi205 - interferon activated gene 205  Mmp2 - matrix metallopeptidase 2  Ifi203 - interferon activated gene 203  Mmp12 - matrix metallopeptidase 12  H2-M3 - histocompatibility 2, m region locus 3  Irg1 - immunoresponsive gene 1  Tnfaip3 - tumor necrosis factor, alpha-induced protein 3 |
| GO:1902531 | regulation of intracellular signal transduction | 2.02E-5 | 2.07E-3 | 2.01 (15721,1614,184,38) | Trf - transferrin  Icam1 - intercellular adhesion molecule 1  Rnf157 - ring finger protein 157  Inpp4b - inositol polyphosphate-4-phosphatase, type ii  Trem2 - triggering receptor expressed on myeloid cells 2  Fgr - gardner-rasheed feline sarcoma viral (fgr) oncogene homolog Il1rl1 - interleukin 1 receptor-like 1  Fpr2 - formyl peptide receptor 2  Cd74 - cd74 antigen (invariant polypeptide of major histocompatibility complex, class ii antigen-associated)  Slc15a3 - solute carrier family 15, member 3  Prkcb - protein kinase c, beta  Csf1r - colony stimulating factor 1 receptor  Csf2 - colony stimulating factor 2 (granulocyte-macrophage)  Socs3 - suppressor of cytokine signaling 3  Avpi1 - arginine vasopressin-induced 1  Ccl3 - chemokine (c-c motif) ligand 3  Dab2 - disabled 2, mitogen-responsive phosphoprotein  H2-M3 - histocompatibility 2, m region locus 3  Ccl4 - chemokine (c-c motif) ligand 4  P2rx4 - purinergic receptor p2x, ligand-gated ion channel 4  Ccl5 - chemokine (c-c motif) ligand 5  Csf1 - colony stimulating factor 1 (macrophage)  Gpnmb - glycoprotein (transmembrane) nmb  Ccl2 - chemokine (c-c motif) ligand 2  Pde4b - phosphodiesterase 4b, camp specific  Ccl17 - chemokine (c-c motif) ligand 17  Il7r - interleukin 7 receptor  Ndrg2 - n-myc downstream regulated gene 2  Clec4n - c-type lectin domain family 4, member n  Casp1 - caspase 1  Sirpa - signal-regulatory protein alpha  Alox15 - arachidonate 15-lipoxygenase |

|  |  |  |  |  | Mmp8 - matrix metallopeptidase 8  Zeb2 - zinc finger e-box binding homeobox 2  Tgm2 - transglutaminase 2, c polypeptide  Il1rn - interleukin 1 receptor antagonist  Nenf - neuron derived neurotrophic factor  Tnfaip3 - tumor necrosis factor, alpha-induced protein 3 |
| --- | --- | --- | --- | --- | --- |
| GO:0071675 | regulation of mononuclear cell migration | 2.18E-5 | 2.22E-3 | 10.46 (15721,49,184,6) | Ccl2 - chemokine (c-c motif) ligand 2  Lgals3 - lectin, galactose binding, soluble 3  Csf1r - colony stimulating factor 1 receptor  Csf1 - colony stimulating factor 1 (macrophage)  Ccl5 - chemokine (c-c motif) ligand 5  Fpr2 - formyl peptide receptor 2 |
| GO:0070663 | regulation of leukocyte proliferation | 2.22E-5 | 2.24E-3 | 4.34 (15721,236,184,12) | Cd74 - cd74 antigen (invariant polypeptide of major histocompatibility complex, class ii antigen-associated)  Csf1r - colony stimulating factor 1 receptor  Lgals3 - lectin, galactose binding, soluble 3  H2-Ab1 - histocompatibility 2, class ii antigen a, beta 1  Tnfrsf1b - tumor necrosis factor receptor superfamily, member 1b H2-Aa - histocompatibility 2, class ii antigen a, alpha  H2-M3 - histocompatibility 2, m region locus 3  Cd274 - cd274 antigen  Bmi1 - bmi1 polycomb ring finger oncogene  Csf1 - colony stimulating factor 1 (macrophage)  Gpnmb - glycoprotein (transmembrane) nmb  Ccl5 - chemokine (c-c motif) ligand 5 |
| GO:0048245 | eosinophil chemotaxis | 2.24E-5 | 2.25E-3 | 22.78 (15721,15,184,4) | Ccl2 - chemokine (c-c motif) ligand 2  Lgals3 - lectin, galactose binding, soluble 3  Ccl4 - chemokine (c-c motif) ligand 4  Ccl5 - chemokine (c-c motif) ligand 5 |
| GO:0050766 | positive regulation of phagocytosis | 2.49E-5 | 2.49E-3 | 8.08 (15721,74,184,7) | Ccl2 - chemokine (c-c motif) ligand 2  Sirpa - signal-regulatory protein alpha  Clec7a - c-type lectin domain family 7, member a  Trem2 - triggering receptor expressed on myeloid cells 2  Slc11a1 - solute carrier family 11 (proton-coupled divalent metal ion transporters), member 1  Mfge8 - milk fat globule-egf factor 8 protein  Fpr2 - formyl peptide receptor 2 |
| GO:0032963 | collagen metabolic process | 2.76E-5 | 2.73E-3 | 10.05 (15721,51,184,6) | Mmp2 - matrix metallopeptidase 2  Mmp13 - matrix metallopeptidase 13  Ctsb - cathepsin b  Mmp12 - matrix metallopeptidase 12  Mmp8 - matrix metallopeptidase 8  Ctss - cathepsin s |
| GO:0070098 | chemokine-mediated signaling pathway | 2.76E-5 | 2.71E-3 | 10.05 (15721,51,184,6) | Cxcl2 - chemokine (c-x-c motif) ligand 2  Ccl2 - chemokine (c-c motif) ligand 2  Ccl17 - chemokine (c-c motif) ligand 17  Ccl3 - chemokine (c-c motif) ligand 3  Ccl4 - chemokine (c-c motif) ligand 4  Ccl5 - chemokine (c-c motif) ligand 5 |
| GO:1904141 | positive regulation of microglial cell migration | 3.07E-5 | 3.01E-3 | 42.72 (15721,6,184,3) | Trem2 - triggering receptor expressed on myeloid cells 2  P2rx4 - purinergic receptor p2x, ligand-gated ion channel 4  Csf1 - colony stimulating factor 1 (macrophage) |
| GO:0060760 | positive regulation of response to cytokine stimulus | 3.08E-5 | 3E-3 | 9.86 (15721,52,184,6) | Cd74 - cd74 antigen (invariant polypeptide of major histocompatibility complex, class ii antigen-associated)  Ikbke - inhibitor of kappab kinase epsilon  Casp1 - caspase 1  Mmp12 - matrix metallopeptidase 12  Trem2 - triggering receptor expressed on myeloid cells 2  Csf1 - colony stimulating factor 1 (macrophage) |
|  |  |  |  |  | Icam1 - intercellular adhesion molecule 1  Myo7a - myosin viia  Cxcl2 - chemokine (c-x-c motif) ligand 2  Cd9 - cd9 antigen  Csf1r - colony stimulating factor 1 receptor  Mmp2 - matrix metallopeptidase 2 |

| GO:0006928 | movement of cell or subcellular component | 3.13E-5 | 3.02E-3 | 2.14 (15721,1277,184,32) | Evl - ena-vasodilator stimulated phosphoprotein  Alcam - activated leukocyte cell adhesion molecule  Ccl3 - chemokine (c-c motif) ligand 3  Mmp12 - matrix metallopeptidase 12  Ccl4 - chemokine (c-c motif) ligand 4  Saa3 - serum amyloid a 3  Emb - embigin  Ccl5 - chemokine (c-c motif) ligand 5  Ccl2 - chemokine (c-c motif) ligand 2  Pde4b - phosphodiesterase 4b, camp specific  Ccl17 - chemokine (c-c motif) ligand 17  Ccr5 - chemokine (c-c motif) receptor 5  Lgals3 - lectin, galactose binding, soluble 3  Sirpa - signal-regulatory protein alpha  Fmnl2 - formin-like 2  Epx - eosinophil peroxidase A  trnl1 - attractin like 1  Uty - ubiquitously transcribed tetratricopeptide repeat gene,  y chromosome  Zeb2 - zinc finger e-box binding homeobox 2  Cd34 - cd34 antigen  Acvr1 - activin a receptor, type 1  Ccrl2 - chemokine (c-c motif) receptor-like 2  Lpxn - leupaxin  Tnfaip3 - tumor necrosis factor, alpha-induced protein 3    Egr3 – early growth response 3  Egr2 - early growth response 2 |
| --- | --- | --- | --- | --- | --- |
| GO:0002250 | adaptive immune response | 3.14E-5 | 3.01E-3 | 4.56 (15721,206,184,11) | Prkcb - protein kinase c, beta  Cd74 - cd74 antigen (invariant polypeptide of major histocompatibility complex, class ii antigen-associated)  H2-Ab1 - histocompatibility 2, class ii antigen a, beta 1  Clec4n - c-type lectin domain family 4, member n  H2-Aa - histocompatibility 2, class ii antigen a, alpha A  lcam - activated leukocyte cell adhesion molecule  H2-M3 - histocompatibility 2, m region locus 3  Cd274 - cd274 antigen  Slc11a1 - solute carrier family 11 (proton-coupled divalent metal ion transporters), member 1  H2-DMa - histocompatibility 2, class ii, locus dma  H2-Eb1 - histocompatibility 2, class ii antigen e beta |
| GO:0009967 | positive regulation of signal transduction | 3.17E-5 | 3.02E-3 | 2.05 (15721,1460,184,35) | Ikbke - inhibitor of kappab kinase epsilon  Trf - transferrin  Icam1 - intercellular adhesion molecule 1  Trem2 - triggering receptor expressed on myeloid cells 2  Fgr - gardner-rasheed feline sarcoma viral (fgr) oncogene homolog Fpr2 - formyl peptide receptor 2  Cd74 - cd74 antigen (invariant polypeptide of major histocompatibility complex, class ii antigen-associated)  Slc15a3 - solute carrier family 15, member 3  Prkcb - protein kinase c, beta  Csf1r - colony stimulating factor 1 receptor  Csf2 - colony stimulating factor 2 (granulocyte-macrophage)  Avpi1 - arginine vasopressin-induced 1  Mmp12 - matrix metallopeptidase 12  Ccl3 - chemokine (c-c motif) ligand 3  Dab2 - disabled 2, mitogen-responsive phosphoprotein  Ccl4 - chemokine (c-c motif) ligand 4  P2rx4 - purinergic receptor p2x, ligand-gated ion channel 4  Gpnmb - glycoprotein (transmembrane) nmb  Ccl5 - chemokine (c-c motif) ligand 5  Csf1 - colony stimulating factor 1 (macrophage)  Ccl2 - chemokine (c-c motif) ligand 2  Ccl17 - chemokine (c-c motif) ligand 17  Il7r - interleukin 7 receptor  Clec4n - c-type lectin domain family 4, member n  Casp1 - caspase 1  Alox15 - arachidonate 15-lipoxygenase  Fabp5 - fatty acid binding protein 5, epidermal  Mmp8 - matrix metallopeptidase 8  Zeb2 - zinc finger e-box binding homeobox 2  Tgm2 - transglutaminase 2, c polypeptide  Inhba - inhibin beta-a  Il1rn - interleukin 1 receptor antagonist  Acvr1 - activin a receptor, type 1  Nenf - neuron derived neurotrophic factor  Tnfaip3 - tumor necrosis factor, alpha-induced protein 3 |
|  |  |  |  |  | Csf1r - colony stimulating factor 1 receptor |

| GO:0045672 | positive regulation of osteoclast differentiation | 3.24E-5 | 3.07E-3 | 13.35 (15721,32,184,5) | Ccl3 - chemokine (c-c motif) ligand 3  Trem2 - triggering receptor expressed on myeloid cells 2  Ccl5 - chemokine (c-c motif) ligand 5  Csf1 - colony stimulating factor 1 (macrophage) |
| --- | --- | --- | --- | --- | --- |
| GO:0042127 | regulation of cell proliferation | 3.39E-5 | 3.19E-3 | 1.99 (15721,1589,184,37) | Chst11 - carbohydrate sulfotransferase 11  Cebpa - ccaat/enhancer binding protein (c/ebp), alpha  Trf - transferrin  Cd274 - cd274 antigen  Il1rl1 - interleukin 1 receptor-like 1  Ifi30 - interferon gamma inducible protein 30  Hck - hemopoietic cell kinase  Cd74 - cd74 antigen (invariant polypeptide of major histocompatibility complex, class ii antigen-associated)  Mmp2 - matrix metallopeptidase 2  Csf1r - colony stimulating factor 1 receptor  Cd9 - cd9 antigen  Csf2 - colony stimulating factor 2 (granulocyte-macrophage)  Clec7a - c-type lectin domain family 7, member a  Junb - jun-b oncogene  Mmp12 - matrix metallopeptidase 12  Dab2 - disabled 2, mitogen-responsive phosphoprotein  H2-M3 - histocompatibility 2, m region locus 3  Dach1 - dachshund 1 (drosophila)  Bmi1 - bmi1 polycomb ring finger oncogene  Csf1 - colony stimulating factor 1 (macrophage)  Ccl5 - chemokine (c-c motif) ligand 5  Gpnmb - glycoprotein (transmembrane) nmb  Itgax - integrin alpha x  Ccl2 - chemokine (c-c motif) ligand 2  Lgals3 - lectin, galactose binding, soluble 3  Ccr5 - chemokine (c-c motif) receptor 5  Il7r - interleukin 7 receptor  Ndrg2 - n-myc downstream regulated gene 2  Tnfrsf1b - tumor necrosis factor receptor superfamily, member 1b H2-Ab1 - histocompatibility 2, class ii antigen a, beta  H2-Aa - histocompatibility 2, class ii antigen a, alpha  Tnfrsf9 - tumor necrosis factor receptor superfamily, member 9 Inhba - inhibin beta-a  Tgm2 - transglutaminase 2, c polypeptide  Runx1 - runt related transcription factor 1  Egr3 - early growth response 3  Tnfaip3 - tumor necrosis factor, alpha-induced protein 3 |
| GO:0001959 | regulation of cytokine-mediated signaling pathway | 3.43E-5 | 3.21E-3 | 6.45 (15721,106,184,8) | Cd74 - cd74 antigen (invariant polypeptide of major histocompatibility complex, class ii antigen-associated)  Padi2 - peptidyl arginine deiminase, type ii  Il1rn - interleukin 1 receptor antagonist  Ikbke - inhibitor of kappab kinase epsilon  Casp1 - caspase 1  Mmp12 - matrix metallopeptidase 12  Trem2 - triggering receptor expressed on myeloid cells 2  Csf1 - colony stimulating factor 1 (macrophage) |
| GO:0050921 | positive regulation of chemotaxis | 3.55E-5 | 3.31E-3 | 5.57 (15721,138,184,9) | Ccl2 - chemokine (c-c motif) ligand 2  Cd74 - cd74 antigen (invariant polypeptide of major histocompatibility complex, class ii antigen-associated)  Csf1r - colony stimulating factor 1 receptor    Ccl4 - chemokine (c-c motif) ligand 4  Trem2 - triggering receptor expressed on myeloid cells 2  P2rx4 - purinergic receptor p2x, ligand-gated ion channel 4  Ccl5 - chemokine (c-c motif) ligand 5  Csf1 - colony stimulating factor 1 (macrophage)  Fpr2 - formyl peptide receptor 2 |
| GO:0034612 | response to tumor necrosis factor | 3.67E-5 | 3.4E-3 | 6.39 (15721,107,184,8) | Ccl2 - chemokine (c-c motif) ligand 2  Ccl17 - chemokine (c-c motif) ligand 17  Cebpa - ccaat/enhancer binding protein (c/ebp), alpha  Ccl3 - chemokine (c-c motif) ligand 3  Ccl4 - chemokine (c-c motif) ligand 4  Irg1 - immunoresponsive gene 1 Gch1 - gtp cyclohydrolase 1  Ccl5 - chemokine (c-c motif) ligand 5 |
|  |  |  |  |  | Mpp1 - membrane protein, palmitoylated  Ccl2 - chemokine (c-c motif) ligand 2  Cd74 - cd74 antigen (invariant polypeptide of major histocompatibility complex, class ii antigen-associated) |

| GO:0050920 | regulation of chemotaxis | 3.74E-5 | 3.44E-3 | 4.48 (15721,210,184,11) | Csf1r - colony stimulating factor 1 receptor  Padi2 - peptidyl arginine deiminase, type ii  Ccl4 - chemokine (c-c motif) ligand 4  Trem2 - triggering receptor expressed on myeloid cells 2  P2rx4 - purinergic receptor p2x, ligand-gated ion channel 4  Csf1 - colony stimulating factor 1 (macrophage)  Ccl5 - chemokine (c-c motif) ligand 5  Fpr2 - formyl peptide receptor 2 |
| --- | --- | --- | --- | --- | --- |
| GO:0019221 | cytokine-mediated signaling pathway | 3.76E-5 | 3.44E-3 | 4.12 (15721,249,184,12) | Cxcl2 - chemokine (c-x-c motif) ligand 2  Ccl2 - chemokine (c-c motif) ligand 2  Cd74 - cd74 antigen (invariant polypeptide of major histocompatibility complex, class ii antigen-associated)  Csf1r - colony stimulating factor 1 receptor  Ccl17 - chemokine (c-c motif) ligand 17  Csf2ra - colony stimulating factor 2 receptor, alpha, low-affinity (granulocyte-macrophage)  Cebpa - ccaat/enhancer binding protein (c/ebp), alpha  Ccl3 - chemokine (c-c motif) ligand 3  Ccl4 - chemokine (c-c motif) ligand 4  Il3ra - interleukin 3 receptor, alpha chain  Csf1 - colony stimulating factor 1 (macrophage)  Ccl5 - chemokine (c-c motif) ligand 5 |
| GO:0048518 | positive regulation of biological process | 3.8E-5 | 3.45E-3 | 1.41 (15721,5511,184,91) | Zmat3 - zinc finger matrin type 3  Trf - transferrin  Mtmr9 - myotubularin related protein 9  Decr1 - 2,4-dienoyl coa reductase 1, mitochondrial  Fpr1 - formyl peptide receptor 1  Akap11 - a kinase (prka) anchor protein 11  Eif2s3y - eukaryotic translation initiation factor 2, subunit 3, structural gene y-linked  Fpr2 - formyl peptide receptor 2  Prkcb - protein kinase c, beta  Cd74 - cd74 antigen (invariant polypeptide of major histocompatibility complex, class ii antigen-associated)  Mmp2 - matrix metallopeptidase 2  Socs3 - suppressor of cytokine signaling 3  Mmp13 - matrix metallopeptidase 13  Hivep3 - human immunodeficiency virus type i enhancer binding protein 3  Junb - jun-b oncogene  Mmp12 - matrix metallopeptidase 12  Dab2 - disabled 2, mitogen-responsive phosphoprotein  H2-M3 - histocompatibility 2, m region locus 3  Gch1 - gtp cyclohydrolase 1  P2rx4 - purinergic receptor p2x, ligand-gated ion channel 4  H2-DMa - histocompatibility 2, class ii, locus dma  Gpnmb - glycoprotein (transmembrane) nmb  Ccl2 - chemokine (c-c motif) ligand 2  Itgax - integrin alpha x  Ccl17 - chemokine (c-c motif) ligand 17  Pde4b - phosphodiesterase 4b, camp specific  Tnfrsf1b - tumor necrosis factor receptor superfamily, member 1b Clec4n - c-type lectin domain family 4, member n  Alox15 - arachidonate 15-lipoxygenase  Fabp5 - fatty acid binding protein 5, epidermal  Slc11a1 - solute carrier family 11 (proton-coupled divalent metal ion transporters), member 1  Mmp8 - matrix metallopeptidase 8  Actr3b - arp3 actin-related protein 3b  Ifi205 - interferon activated gene 205  Cd34 - cd34 antigen  Padi2 - peptidyl arginine deiminase, type ii  Plxnc1 - plexin c1  Acvr1 - activin a receptor, type 1  Nenf - neuron derived neurotrophic factor  Tnfaip3 - tumor necrosis factor, alpha-induced protein 3  Cyp4f18 - cytochrome p450, family 4, subfamily f, polypeptide 18 Ikbke - inhibitor of kappab kinase epsilon  AF251705 - cdna sequence af251705  Cebpa - ccaat/enhancer binding protein (c/ebp), alpha  Fam46a - family with sequence similarity 46, member a  Icam1 - intercellular adhesion molecule 1  Acsl4 - acyl-coa synthetase long-chain family member 4  Rnf157 - ring finger protein 157  Trem2 - triggering receptor expressed on myeloid cells 2  Fgr - gardner-rasheed feline sarcoma viral (fgr) oncogene homolog Cd274 - cd274 antigen  Cst7 - cystatin f (leukocystatin) |

|  |  |  |  |  | Il1rl1 - interleukin 1 receptor-like 1  Hck - hemopoietic cell kinase  Slc15a3 - solute carrier family 15, member 3  Csf1r - colony stimulating factor 1 receptor  Ifi203 - interferon activated gene 203  Csf2 - colony stimulating factor 2 (granulocyte-macrophage)  Avpi1 - arginine vasopressin-induced 1  Fam20c - family with sequence similarity 20, member c  Evl - ena-vasodilator stimulated phosphoprotein  Clec7a - c-type lectin domain family 7, member a  Ccl3 - chemokine (c-c motif) ligand 3  Ccl4 - chemokine (c-c motif) ligand 4  Irg1 - immunoresponsive gene 1  Degs1 - degenerative spermatocyte homolog 1 (drosophila)  Bmi1 - bmi1 polycomb ring finger oncogene  Ccl5 - chemokine (c-c motif) ligand 5  Csf1 - colony stimulating factor 1 (macrophage)  Ctss - cathepsin s  Lgals3 - lectin, galactose binding, soluble 3  Ccr5 - chemokine (c-c motif) receptor 5  H2-D1 - histocompatibility 2, d region locus 1  Il7r - interleukin 7 receptor  H2-Ab1 - histocompatibility 2, class ii antigen a, beta 1  H2-Aa - histocompatibility 2, class ii antigen a, alpha  Paox - polyamine oxidase (exo-n4-amino)  Sirpa - signal-regulatory protein alpha  Casp1 - caspase 1  Epx - eosinophil peroxidase  Thy1 - thymus cell antigen 1, theta  H2-K1 - histocompatibility 2, k1, k region  Zeb2 - zinc finger e-box binding homeobox 2  Tgm2 - transglutaminase 2, c polypeptide  Inhba - inhibin beta-a  Il1rn - interleukin 1 receptor antagonist  Runx1 - runt related transcription factor 1  Egr3 - early growth response 3  Prg2 - proteoglycan 2, bone marrow  Mfge8 - milk fat globule-egf factor 8 protein  Egr2 - early growth response 2 |
| --- | --- | --- | --- | --- | --- |
| GO:0010941 | regulation of cell death | 4.15E-5 | 3.75E-3 | 1.97 (15721,1604,184,37) | Zmat3 - zinc finger matrin type 3  Chst11 - carbohydrate sulfotransferase 11  Icam1 - intercellular adhesion molecule 1  Ctsb - cathepsin b  Acot1 - acyl-coa thioesterase 1  Rnf157 - ring finger protein 157  Trem2 - triggering receptor expressed on myeloid cells 2  Cd274 - cd274 antigen  Hck - hemopoietic cell kinase  Fpr2 - formyl peptide receptor 2  Cd74 - cd74 antigen (invariant polypeptide of major histocompatibility complex, class ii antigen-associated)  Mmp2 - matrix metallopeptidase 2  Csf1r - colony stimulating factor 1 receptor  Csf2 - colony stimulating factor 2 (granulocyte-macrophage)  Socs3 - suppressor of cytokine signaling 3  Ccl3 - chemokine (c-c motif) ligand 3  Dab2 - disabled 2, mitogen-responsive phosphoprotein  H2-M3 - histocompatibility 2, m region locus 3  Degs1 - degenerative spermatocyte homolog 1 (drosophila)  Bmi1 - bmi1 polycomb ring finger oncogene  Gpnmb - glycoprotein (transmembrane) nmb  Csf1 - colony stimulating factor 1 (macrophage)  Ccl5 - chemokine (c-c motif) ligand 5  Lgals3 - lectin, galactose binding, soluble 3  Ccr5 - chemokine (c-c motif) receptor 5  Il7r - interleukin 7 receptor  Tnfrsf1b - tumor necrosis factor receptor superfamily, member 1b Casp1 - caspase 1  Inhba - inhibin beta-a  Cd34 - cd34 antigen  Tgm2 - transglutaminase 2, c polypeptide  Il1rn - interleukin 1 receptor antagonist  Serpinb2 - serine (or cysteine) peptidase inhibitor, clade b, member 2 Acvr1 - activin a receptor, type 1  Nenf - neuron derived neurotrophic factor  Egr3 - early growth response 3  Tnfaip3 - tumor necrosis factor, alpha-induced protein 3 |
|  |  |  |  |  | Ccl2 - chemokine (c-c motif) ligand 2 |

| GO:0008360 | regulation of cell shape | 4.21E-5 | 3.78E-3 | 5.45 (15721,141,184,9) | Csf1r - colony stimulating factor 1 receptor  Plxnc1 - plexin c1  Fmnl2 - formin-like 2  Icam1 - intercellular adhesion molecule 1  Ccl3 - chemokine (c-c motif) ligand 3  Fgr - gardner-rasheed feline sarcoma viral (fgr) oncogene homolog Parvb - parvin, beta  Hck - hemopoietic cell kinase |
| --- | --- | --- | --- | --- | --- |
| GO:0043408 | regulation of MAPK cascade | 4.22E-5 | 3.77E-3 | 2.66 (15721,674,184,21) | Ccl2 - chemokine (c-c motif) ligand 2  Ccl17 - chemokine (c-c motif) ligand 17  Ndrg2 - n-myc downstream regulated gene 2  Sirpa - signal-regulatory protein alpha  Icam1 - intercellular adhesion molecule 1  Trf - transferrin  Alox15 - arachidonate 15-lipoxygenase  Trem2 - triggering receptor expressed on myeloid cells 2  Mmp8 - matrix metallopeptidase 8  Zeb2 - zinc finger e-box binding homeobox 2  Fpr2 - formyl peptide receptor 2  Cd74 - cd74 antigen (invariant polypeptide of major histocompatibility complex, class ii antigen-associated)  Csf1r - colony stimulating factor 1 receptor  Il1rn - interleukin 1 receptor antagonist  Avpi1 - arginine vasopressin-induced 1  Ccl3 - chemokine (c-c motif) ligand 3  Dab2 - disabled 2, mitogen-responsive phosphoprotein  Ccl4 - chemokine (c-c motif) ligand 4  Nenf - neuron derived neurotrophic factor  Gpnmb - glycoprotein (transmembrane) nmb  Ccl5 - chemokine (c-c motif) ligand 5 |
| GO:0048519 | negative regulation of biological process | 4.49E-5 | 3.98E-3 | 1.45 (15721,4820,184,82) | Mgll - monoglyceride lipase  Zmat3 - zinc finger matrin type 3  Mtmr9 - myotubularin related protein 9  Acot1 - acyl-coa thioesterase 1  Fpr2 - formyl peptide receptor 2  Prkcb - protein kinase c, beta  Cd74 - cd74 antigen (invariant polypeptide of major histocompatibility complex, class ii antigen-associated)  Cd9 - cd9 antigen  H2-Oa - histocompatibility 2, o region alpha locus  Mmp2 - matrix metallopeptidase 2  Socs3 - suppressor of cytokine signaling 3  Dab2 - disabled 2, mitogen-responsive phosphoprotein  Mmp12 - matrix metallopeptidase 12  H2-M3 - histocompatibility 2, m region locus 3  Dach1 - dachshund 1 (drosophila)  P2rx4 - purinergic receptor p2x, ligand-gated ion channel 4  Gpnmb - glycoprotein (transmembrane) nmb  Ccl2 - chemokine (c-c motif) ligand 2  Ccl17 - chemokine (c-c motif) ligand 17  Pde4b - phosphodiesterase 4b, camp specific  Tnfrsf1b - tumor necrosis factor receptor superfamily, member 1b Alox15 - arachidonate 15-lipoxygenase  Tnfrsf9 - tumor necrosis factor receptor superfamily, member 9 Fabp5 - fatty acid binding protein 5, epidermal  Sestd1 - sec14 and spectrin domains 1  Slc11a1 - solute carrier family 11 (proton-coupled divalent metal ion transporters), member 1  Mmp8 - matrix metallopeptidase 8  Cd34 - cd34 antigen  Padi2 - peptidyl arginine deiminase, type ii  Plxnc1 - plexin c1  Acvr1 - activin a receptor, type 1  Nenf - neuron derived neurotrophic factor  Lpxn - leupaxin  Tnfaip3 - tumor necrosis factor, alpha-induced protein 3  Zhx2 - zinc fingers and homeoboxes 2  Cyp4f18 - cytochrome p450, family 4, subfamily f, polypeptide 18 Rnf128 - ring finger protein 128  Chst11 - carbohydrate sulfotransferase 11  Cebpa - ccaat/enhancer binding protein (c/ebp), alpha  Fam46a - family with sequence similarity 46, member a  Ctsb - cathepsin b  Icam1 - intercellular adhesion molecule 1  Acsl4 - acyl-coa synthetase long-chain family member 4  Rnf157 - ring finger protein 157  Inpp4b - inositol polyphosphate-4-phosphatase, type ii  Trem2 - triggering receptor expressed on myeloid cells 2 |

|  |  |  |  |  | Cd274 - cd274 antigen  Cst7 - cystatin f (leukocystatin)  Fgr - gardner-rasheed feline sarcoma viral (fgr) oncogene homolog Fgl2 - fibrinogen-like protein 2  Il1rl1 - interleukin 1 receptor-like 1  Ifi30 - interferon gamma inducible protein 30  Hck - hemopoietic cell kinase  Csf1r - colony stimulating factor 1 receptor  Csf2 - colony stimulating factor 2 (granulocyte-macrophage)  Evl - ena-vasodilator stimulated phosphoprotein  Ccl4 - chemokine (c-c motif) ligand 4  Irg1 - immunoresponsive gene 1  Bmi1 - bmi1 polycomb ring finger oncogene  Ccl5 - chemokine (c-c motif) ligand 5  Csf1 - colony stimulating factor 1 (macrophage)  Procr - protein c receptor, endothelial  Ccr5 - chemokine (c-c motif) receptor 5  H2-D1 - histocompatibility 2, d region locus 1  Lgals3 - lectin, galactose binding, soluble 3  Il7r - interleukin 7 receptor  Ndrg2 - n-myc downstream regulated gene 2  H2-Ab1 - histocompatibility 2, class ii antigen a, beta 1  H2-Aa - histocompatibility 2, class ii antigen a, alpha  Sirpa - signal-regulatory protein alpha  Epx - eosinophil peroxidase  Ifi27 - interferon, alpha-inducible protein 27  Thy1 - thymus cell antigen 1, theta  H2-K1 - histocompatibility 2, k1, k region  Zeb2 - zinc finger e-box binding homeobox 2  Inhba - inhibin beta-a  Il1rn - interleukin 1 receptor antagonist  Serpinb2 - serine (or cysteine) peptidase inhibitor, clade b, member 2  Runx1 - runt related transcription factor 1  Egr3 - early growth response 3  Prg2 - proteoglycan 2, bone marrow  Nfkbie - nuclear factor of kappa light polypeptide gene enhancer in b cells inhibitor, epsilon |
| --- | --- | --- | --- | --- | --- |
| GO:0048870 | cell motility | 5.06E-5 | 4.46E-3 | 2.37 (15721,900,184,25) | Icam1 - intercellular adhesion molecule 1  Cxcl2 - chemokine (c-x-c motif) ligand 2  Cd9 - cd9 antigen  Mmp2 - matrix metallopeptidase 2  Ccl3 - chemokine (c-c motif) ligand 3  Mmp12 - matrix metallopeptidase 12  Ccl4 - chemokine (c-c motif) ligand 4  Saa3 - serum amyloid a 3  Ccl5 - chemokine (c-c motif) ligand 5  Ccl2 - chemokine (c-c motif) ligand 2  Ccl17 - chemokine (c-c motif) ligand 17  Ccr5 - chemokine (c-c motif) receptor 5  Lgals3 - lectin, galactose binding, soluble 3  Pde4b - phosphodiesterase 4b, camp specific  Sirpa - signal-regulatory protein alpha  Fmnl2 - formin-like 2  Epx - eosinophil peroxidase  Atrnl1 - attractin like 1  Zeb2 - zinc finger e-box binding homeobox 2  Cd34 - cd34 antigen  Acvr1 - activin a receptor, type 1  Ccrl2 - chemokine (c-c motif) receptor-like 2  Lpxn - leupaxin  Egr3 - early growth response 3  Tnfaip3 - tumor necrosis factor, alpha-induced protein 3 |
| GO:0048247 | lymphocyte chemotaxis | 5.08E-5 | 4.45E-3 | 12.21 (15721,35,184,5) | Ccl2 - chemokine (c-c motif) ligand 2  Ccl17 - chemokine (c-c motif) ligand 17  Ccl3 - chemokine (c-c motif) ligand 3  Ccl4 - chemokine (c-c motif) ligand 4  Ccl5 - chemokine (c-c motif) ligand 5 |
| GO:0045621 | positive regulation of lymphocyte differentiation | 5.1E-5 | 4.45E-3 | 6.10 (15721,112,184,8) | Cd74 - cd74 antigen (invariant polypeptide of major histocompatibility complex, class ii antigen-associated)  Il7r - interleukin 7 receptor  H2-Aa - histocompatibility 2, class ii antigen a, alpha  H2-M3 - histocompatibility 2, m region locus 3  Bmi1 - bmi1 polycomb ring finger oncogene  H2-DMa - histocompatibility 2, class ii, locus dma  Runx1 - runt related transcription factor 1  Egr3 - early growth response 3 |

| GO:0007166 | cell surface receptor signaling pathway | 5.17E-5 | 4.48E-3 | 2.00 (15721,1495,184,35) | Cebpa - ccaat/enhancer binding protein (c/ebp), alpha  Trem2 - triggering receptor expressed on myeloid cells 2  Fgr - gardner-rasheed feline sarcoma viral (fgr) oncogene homolog Cd274 - cd274 antigen  Fpr1 - formyl peptide receptor 1  Emr4 - egf-like module containing, mucin-like, hormone receptor-like sequence 4  Fpr2 - formyl peptide receptor 2  Hck - hemopoietic cell kinase  Cxcl2 - chemokine (c-x-c motif) ligand 2  Cd74 - cd74 antigen (invariant polypeptide of major histocompatibility complex, class ii antigen-associated)  Prkcb - protein kinase c, beta  Csf1r - colony stimulating factor 1 receptor  Csf2ra - colony stimulating factor 2 receptor, alpha, low-affinity (granulocyte-macrophage)  Ccl3 - chemokine (c-c motif) ligand 3  Dab2 - disabled 2, mitogen-responsive phosphoprotein  H2-M3 - histocompatibility 2, m region locus 3  Ccl4 - chemokine (c-c motif) ligand 4  P2rx4 - purinergic receptor p2x, ligand-gated ion channel 4  Ccl5 - chemokine (c-c motif) ligand 5  Csf1 - colony stimulating factor 1 (macrophage)  Ccl2 - chemokine (c-c motif) ligand 2  Itgax - integrin alpha x  Pde4b - phosphodiesterase 4b, camp specific  Ccl17 - chemokine (c-c motif) ligand 17  Ndrg2 - n-myc downstream regulated gene 2  Tnfrsf1b - tumor necrosis factor receptor superfamily, member 1b H2-Ab1 - histocompatibility 2, class ii antigen a, beta 1  Thy1 - thymus cell antigen 1, theta  Uty - ubiquitously transcribed tetratricopeptide repeat gene, y chromosome  Inhba - inhibin beta-a  Plxnc1 - plexin c1  Ngfrap1 - nerve growth factor receptor (tnfrsf16) associated protein 1  Acvr1 - activin a receptor, type 1  Il3ra - interleukin 3 receptor, alpha chain  Lpxn - leupaxin |
| --- | --- | --- | --- | --- | --- |
| GO:0045619 | regulation of lymphocyte differentiation | 5.18E-5 | 4.46E-3 | 4.75 (15721,180,184,10) | Cd74 - cd74 antigen (invariant polypeptide of major histocompatibility complex, class ii antigen-associated)  H2-Oa - histocompatibility 2, o region alpha locus  Il7r - interleukin 7 receptor  H2-Aa - histocompatibility 2, class ii antigen a, alpha  H2-M3 - histocompatibility 2, m region locus 3  Bmi1 - bmi1 polycomb ring finger oncogene  Fgl2 - fibrinogen-like protein 2  H2-DMa - histocompatibility 2, class ii, locus dma  Runx1 - runt related transcription factor 1  Egr3 - early growth response 3 |
| GO:0045595 | regulation of cell differentiation | 5.19E-5 | 4.45E-3 | 1.89 (15721,1813,184,40) | Mgll - monoglyceride lipase  Cebpa - ccaat/enhancer binding protein (c/ebp), alpha  Trf - transferrin  Rnf157 - ring finger protein 157  Inpp4b - inositol polyphosphate-4-phosphatase, type ii  Trem2 - triggering receptor expressed on myeloid cells 2  Fgl2 - fibrinogen-like protein 2  Akap11 - a kinase (prka) anchor protein 11  Cd74 - cd74 antigen (invariant polypeptide of major histocompatibility complex, class ii antigen-associated)  H2-Oa - histocompatibility 2, o region alpha locus  Csf1r - colony stimulating factor 1 receptor  Csf2 - colony stimulating factor 2 (granulocyte-macrophage)  Socs3 - suppressor of cytokine signaling 3  Fam20c - family with sequence similarity 20, member c  Junb - jun-b oncogene  Ccl3 - chemokine (c-c motif) ligand 3  Dab2 - disabled 2, mitogen-responsive phosphoprotein  H2-M3 - histocompatibility 2, m region locus 3  P2rx4 - purinergic receptor p2x, ligand-gated ion channel 4  H2-DMa - histocompatibility 2, class ii, locus dma  Bmi1 - bmi1 polycomb ring finger oncogene  Ccl5 - chemokine (c-c motif) ligand 5  Csf1 - colony stimulating factor 1 (macrophage)  H2-D1 - histocompatibility 2, d region locus 1  Ccr5 - chemokine (c-c motif) receptor 5 |

|  |  |  |  |  | Ccl17 - chemokine (c-c motif) ligand 17  Il7r - interleukin 7 receptor  Tnfrsf1b - tumor necrosis factor receptor superfamily, member 1b H2-Aa - histocompatibility 2, class ii antigen a, alpha  Thy1 - thymus cell antigen 1, theta  H2-K1 - histocompatibility 2, k1, k region  Zeb2 - zinc finger e-box binding homeobox 2  Inhba - inhibin beta-a  Cd34 - cd34 antigen  Plxnc1 - plexin c1  Acvr1 - activin a receptor, type 1  Runx1 - runt related transcription factor 1  Egr3 - early growth response 3  Zhx2 - zinc fingers and homeoboxes 2  Egr2 - early growth response 2 |
| --- | --- | --- | --- | --- | --- |
| GO:2000026 | regulation of multicellular organismal development | 5.23E-5 | 4.46E-3 | 1.81 (15721,2076,184,44) | Mgll - monoglyceride lipase  Trf - transferrin  Rnf157 - ring finger protein 157  Inpp4b - inositol polyphosphate-4-phosphatase, type ii  Trem2 - triggering receptor expressed on myeloid cells 2  Cst7 - cystatin f (leukocystatin)  Cd274 - cd274 antigen  Fgl2 - fibrinogen-like protein 2  Akap11 - a kinase (prka) anchor protein 11  Cd74 - cd74 antigen (invariant polypeptide of major histocompatibility complex, class ii antigen-associated)  Prkcb - protein kinase c, beta  H2-Oa - histocompatibility 2, o region alpha locus  Csf1r - colony stimulating factor 1 receptor  Fam20c - family with sequence similarity 20, member c  Mmp12 - matrix metallopeptidase 12  Dab2 - disabled 2, mitogen-responsive phosphoprotein  Ccl3 - chemokine (c-c motif) ligand 3  H2-M3 - histocompatibility 2, m region locus 3  P2rx4 - purinergic receptor p2x, ligand-gated ion channel 4  H2-DMa - histocompatibility 2, class ii, locus dma  Bmi1 - bmi1 polycomb ring finger oncogene  Csf1 - colony stimulating factor 1 (macrophage)  Ccl5 - chemokine (c-c motif) ligand 5  Itgax - integrin alpha x  Ccl2 - chemokine (c-c motif) ligand 2  Lgals3 - lectin, galactose binding, soluble 3  H2-D1 - histocompatibility 2, d region locus 1  Ccr5 - chemokine (c-c motif) receptor 5  Il7r - interleukin 7 receptor  Tnfrsf1b - tumor necrosis factor receptor superfamily, member 1b H2-Aa - histocompatibility 2, class ii antigen a, alpha  Thy1 - thymus cell antigen 1, theta  H2-K1 - histocompatibility 2, k1, k region  Zeb2 - zinc finger e-box binding homeobox 2  Inhba - inhibin beta-a  Cd34 - cd34 antigen  Il1rn - interleukin 1 receptor antagonist  Plxnc1 - plexin c1  Acvr1 - activin a receptor, type 1  Runx1 - runt related transcription factor 1  Egr3 - early growth response 3  Tnfaip3 - tumor necrosis factor, alpha-induced protein 3  Zhx2 - zinc fingers and homeoboxes 2  Egr2 - early growth response 2 |
| GO:0071356 | cellular response to tumor necrosis factor | 5.27E-5 | 4.47E-3 | 7.21 (15721,83,184,7) | Ccl2 - chemokine (c-c motif) ligand 2  Ccl17 - chemokine (c-c motif) ligand 17  Cebpa - ccaat/enhancer binding protein (c/ebp), alpha  Ccl3 - chemokine (c-c motif) ligand 3  Ccl4 - chemokine (c-c motif) ligand 4  Irg1 - immunoresponsive gene 1  Ccl5 - chemokine (c-c motif) ligand 5 |
| GO:1904139 | regulation of microglial cell migration | 5.33E-5 | 4.5E-3 | 36.62 (15721,7,184,3) | Trem2 - triggering receptor expressed on myeloid cells 2  P2rx4 - purinergic receptor p2x, ligand-gated ion channel 4  Csf1 - colony stimulating factor 1 (macrophage) |
| GO:0061517 | macrophage proliferation | 5.33E-5 | 4.47E-3 | 36.62 (15721,7,184,3) | Csf1r - colony stimulating factor 1 receptor  Trem2 - triggering receptor expressed on myeloid cells 2  Csf1 - colony stimulating factor 1 (macrophage) |
|  |  |  |  |  |  |

| GO:0061518 | microglial cell proliferation | 5.33E-5 | 4.45E-3 | 36.62 (15721,7,184,3) | Csf1r - colony stimulating factor 1 receptor  Trem2 - triggering receptor expressed on myeloid cells 2  Csf1 - colony stimulating factor 1 (macrophage) |
| --- | --- | --- | --- | --- | --- |
| GO:2000425 | regulation of apoptotic cell clearance | 5.33E-5 | 4.43E-3 | 36.62 (15721,7,184,3) | Ccl2 - chemokine (c-c motif) ligand 2  Alox15 - arachidonate 15-lipoxygenase  Trem2 - triggering receptor expressed on myeloid cells 2 |
| GO:0072676 | lymphocyte migration | 5.79E-5 | 4.78E-3 | 8.84 (15721,58,184,6) | Ccl2 - chemokine (c-c motif) ligand 2  Ccl17 - chemokine (c-c motif) ligand 17  Icam1 - intercellular adhesion molecule 1  Ccl3 - chemokine (c-c motif) ligand 3  Ccl4 - chemokine (c-c motif) ligand 4  Ccl5 - chemokine (c-c motif) ligand 5 |
| GO:1903556 | negative regulation of tumor necrosis factor superfamily cytokine production | 5.79E-5 | 4.75E-3 | 8.84 (15721,58,184,6) | Cd34 - cd34 antigen  Sirpa - signal-regulatory protein alpha  Trem2 - triggering receptor expressed on myeloid cells 2  Cd274 - cd274 antigen  Tnfaip3 - tumor necrosis factor, alpha-induced protein 3  Gpnmb - glycoprotein (transmembrane) nmb |
| GO:0032944 | regulation of mononuclear cell proliferation | 5.96E-5 | 4.86E-3 | 4.25 (15721,221,184,11) | Cd74 - cd74 antigen (invariant polypeptide of major histocompatibility complex, class ii antigen-associated)  Lgals3 - lectin, galactose binding, soluble 3  H2-Ab1 - histocompatibility 2, class ii antigen a, beta 1  Tnfrsf1b - tumor necrosis factor receptor superfamily, member 1b H2-Aa - histocompatibility 2, class ii antigen a, alpha  H2-M3 - histocompatibility 2, m region locus 3  Cd274 - cd274 antigen  Bmi1 - bmi1 polycomb ring finger oncogene  Csf1 - colony stimulating factor 1 (macrophage)  Gpnmb - glycoprotein (transmembrane) nmb  Ccl5 - chemokine (c-c motif) ligand 5 |
| GO:0010759 | positive regulation of macrophage chemotaxis | 6.14E-5 | 4.98E-3 | 17.99 (15721,19,184,4) | Ccl2 - chemokine (c-c motif) ligand 2  Csf1r - colony stimulating factor 1 receptor  Ccl5 - chemokine (c-c motif) ligand 5  Csf1 - colony stimulating factor 1 (macrophage) |
| GO:0050777 | negative regulation of immune response | 6.48E-5 | 5.23E-3 | 5.16 (15721,149,184,9) | Lgals3 - lectin, galactose binding, soluble 3  Il7r - interleukin 7 receptor  Alox15 - arachidonate 15-lipoxygenase  Mmp12 - matrix metallopeptidase 12  H2-M3 - histocompatibility 2, m region locus 3  Irg1 - immunoresponsive gene 1  Fgl2 - fibrinogen-like protein 2  Tnfaip3 - tumor necrosis factor, alpha-induced protein 3  Il1rl1 - interleukin 1 receptor-like 1 |
| GO:0060759 | regulation of response to cytokine stimulus | 6.55E-5 | 5.26E-3 | 5.89 (15721,116,184,8) | Cd74 - cd74 antigen (invariant polypeptide of major histocompatibility complex, class ii antigen-associated)  Padi2 - peptidyl arginine deiminase, type ii  Il1rn - interleukin 1 receptor antagonist Ikbke - inhibitor of kappab kinase epsilon  Casp1 - caspase 1  Mmp12 - matrix metallopeptidase 12  Trem2 - triggering receptor expressed on myeloid cells 2  Csf1 - colony stimulating factor 1 (macrophage) |
| GO:0002821 | positive regulation of adaptive immune response | 6.55E-5 | 5.23E-3 | 5.89 (15721,116,184,8) | Cd74 - cd74 antigen (invariant polypeptide of major histocompatibility complex, class ii antigen-associated)  H2-D1 - histocompatibility 2, d region locus 1  H2-Ab1 - histocompatibility 2, class ii antigen a, beta 1  H2-M3 - histocompatibility 2, m region locus 3  Cd274 - cd274 antigen  Slc11a1 - solute carrier family 11 (proton-coupled divalent metal ion transporters), member 1  H2-DMa - histocompatibility 2, class ii, locus dma  H2-K1 - histocompatibility 2, k1, k region |
|  |  |  |  |  | Trf - transferrin  Icam1 - intercellular adhesion molecule 1  Trem2 - triggering receptor expressed on myeloid cells 2 |

| GO:0001934 | positive regulation of protein phosphorylation | 7.09E-5 | 5.64E-3 | 2.32 (15721,919,184,25) | Akap11 - a kinase (prka) anchor protein 11  Fpr2 - formyl peptide receptor 2  Cd74 - cd74 antigen (invariant polypeptide of major histocompatibility complex, class ii antigen-associated)  Csf1r - colony stimulating factor 1 receptor  Csf2 - colony stimulating factor 2 (granulocyte-macrophage)  Avpi1 - arginine vasopressin-induced 1  Ccl3 - chemokine (c-c motif) ligand 3  Dab2 - disabled 2, mitogen-responsive phosphoprotein  Ccl4 - chemokine (c-c motif) ligand 4  Ccl5 - chemokine (c-c motif) ligand 5  Csf1 - colony stimulating factor 1 (macrophage)  Gpnmb - glycoprotein (transmembrane) nmb  Ccl2 - chemokine (c-c motif) ligand 2  Ccl17 - chemokine (c-c motif) ligand 17  Alox15 - arachidonate 15-lipoxygenase  Slc11a1 - solute carrier family 11 (proton-coupled divalent metal ion transporters), member 1  Mmp8 - matrix metallopeptidase 8  Zeb2 - zinc finger e-box binding homeobox 2  Inhba - inhibin beta-a  Il1rn - interleukin 1 receptor antagonist  Acvr1 - activin a receptor, type 1  Nenf - neuron derived neurotrophic factor |
| --- | --- | --- | --- | --- | --- |
| GO:0002690 | positive regulation of leukocyte chemotaxis | 7.13E-5 | 5.64E-3 | 6.87 (15721,87,184,7) | Ccl2 - chemokine (c-c motif) ligand 2  Cd74 - cd74 antigen (invariant polypeptide of major histocompatibility complex, class ii antigen-associated)  Csf1r - colony stimulating factor 1 receptor  Ccl4 - chemokine (c-c motif) ligand 4  Ccl5 - chemokine (c-c motif) ligand 5  Csf1 - colony stimulating factor 1 (macrophage)  Fpr2 - formyl peptide receptor 2 |
| GO:0010647 | positive regulation of cell communication | 7.65E-5 | 6.02E-3 | 1.91 (15721,1651,184,37) | Ikbke - inhibitor of kappab kinase epsilon  Trf - transferrin  Icam1 - intercellular adhesion molecule 1  Acsl4 - acyl-coa synthetase long-chain family member 4  Trem2 - triggering receptor expressed on myeloid cells 2  Fgr - gardner-rasheed feline sarcoma viral (fgr) oncogene homolog Fpr2 - formyl peptide receptor 2  Cd74 - cd74 antigen (invariant polypeptide of major histocompatibility complex, class ii antigen-associated)  Slc15a3 - solute carrier family 15, member 3  Prkcb - protein kinase c, beta  Csf1r - colony stimulating factor 1 receptor  Csf2 - colony stimulating factor 2 (granulocyte-macrophage)  Avpi1 - arginine vasopressin-induced 1  Mmp12 - matrix metallopeptidase 12  Ccl3 - chemokine (c-c motif) ligand 3  Dab2 - disabled 2, mitogen-responsive phosphoprotein  Ccl4 - chemokine (c-c motif) ligand 4  P2rx4 - purinergic receptor p2x, ligand-gated ion channel 4  Ccl5 - chemokine (c-c motif) ligand 5  Csf1 - colony stimulating factor 1 (macrophage)  Gpnmb - glycoprotein (transmembrane) nmb  Ccl2 - chemokine (c-c motif) ligand 2  Ccl17 - chemokine (c-c motif) ligand 17  Il7r - interleukin 7 receptor  Clec4n - c-type lectin domain family 4, member n  Casp1 - caspase 1  Alox15 - arachidonate 15-lipoxygenase  Fabp5 - fatty acid binding protein 5, epidermal  Mmp8 - matrix metallopeptidase 8  Zeb2 - zinc finger e-box binding homeobox 2  Inhba - inhibin beta-a  Tgm2 - transglutaminase 2, c polypeptide  Il1rn - interleukin 1 receptor antagonist  Acvr1 - activin a receptor, type 1  Nenf - neuron derived neurotrophic factor  Runx1 - runt related transcription factor 1  Tnfaip3 - tumor necrosis factor, alpha-induced protein 3 |
| GO:0032652 | regulation of interleukin-1 production | 7.67E-5 | 6E-3 | 6.80 (15721,88,184,7) | Ifi205 - interferon activated gene 205  Ccr5 - chemokine (c-c motif) receptor 5  Casp1 - caspase 1  Sirpa - signal-regulatory protein alpha  Ccl3 - chemokine (c-c motif) ligand 3  Trem2 - triggering receptor expressed on myeloid cells 2 |

|  |  |  |  |  | Tnfaip3 - tumor necrosis factor, alpha-induced protein 3 |
| --- | --- | --- | --- | --- | --- |
| GO:0023056 | positive regulation of signaling | 8.46E-5 | 6.59E-3 | 1.91 (15721,1659,184,37) | Ikbke - inhibitor of kappab kinase epsilon  Trf - transferrin  Icam1 - intercellular adhesion molecule 1  Acsl4 - acyl-coa synthetase long-chain family member 4  Trem2 - triggering receptor expressed on myeloid cells 2  Fgr - gardner-rasheed feline sarcoma viral (fgr) oncogene homolog Fpr2 - formyl peptide receptor 2  Cd74 - cd74 antigen (invariant polypeptide of major histocompatibility complex, class ii antigen-associated)  Slc15a3 - solute carrier family 15, member 3  Prkcb - protein kinase c, beta  Csf1r - colony stimulating factor 1 receptor  Csf2 - colony stimulating factor 2 (granulocyte-macrophage)  Avpi1 - arginine vasopressin-induced 1  Mmp12 - matrix metallopeptidase 12  Ccl3 - chemokine (c-c motif) ligand 3  Dab2 - disabled 2, mitogen-responsive phosphoprotein  Ccl4 - chemokine (c-c motif) ligand 4  P2rx4 - purinergic receptor p2x, ligand-gated ion channel 4  Ccl5 - chemokine (c-c motif) ligand 5  Csf1 - colony stimulating factor 1 (macrophage)  Gpnmb - glycoprotein (transmembrane) nmb  Ccl2 - chemokine (c-c motif) ligand 2  Ccl17 - chemokine (c-c motif) ligand 17  Il7r - interleukin 7 receptor  Clec4n - c-type lectin domain family 4, member n  Casp1 - caspase 1  Alox15 - arachidonate 15-lipoxygenase  Fabp5 - fatty acid binding protein 5, epidermal  Mmp8 - matrix metallopeptidase 8  Zeb2 - zinc finger e-box binding homeobox 2  Inhba - inhibin beta-a  Tgm2 - transglutaminase 2, c polypeptide  Il1rn - interleukin 1 receptor antagonist  Acvr1 - activin a receptor, type 1  Nenf - neuron derived neurotrophic factor  Runx1 - runt related transcription factor 1  Tnfaip3 - tumor necrosis factor, alpha-induced protein 3 |
| GO:0002604 | regulation of dendritic cell antigen processing and presentation | 8.46E-5 | 6.56E-3 | 32.04 (15721,8,184,3) | Cd74 - cd74 antigen (invariant polypeptide of major histocompatibility complex, class ii antigen-associated)  Fgl2 - fibrinogen-like protein 2  Slc11a1 - solute carrier family 11 (proton-coupled divalent metal ion transporters), member 1 |
| GO:0042327 | positive regulation of phosphorylation | 9.09E-5 | 7.01E-3 | 2.24 (15721,991,184,26) | Trf - transferrin  Icam1 - intercellular adhesion molecule 1  Trem2 - triggering receptor expressed on myeloid cells 2  Fgr - gardner-rasheed feline sarcoma viral (fgr) oncogene homolog Akap11 - a kinase (prka) anchor protein 11  Fpr2 - formyl peptide receptor 2  Cd74 - cd74 antigen (invariant polypeptide of major histocompatibility complex, class ii antigen-associated)  Csf1r - colony stimulating factor 1 receptor  Csf2 - colony stimulating factor 2 (granulocyte-macrophage)  Avpi1 - arginine vasopressin-induced 1  Ccl3 - chemokine (c-c motif) ligand 3  Dab2 - disabled 2, mitogen-responsive phosphoprotein  Ccl4 - chemokine (c-c motif) ligand 4  Gpnmb - glycoprotein (transmembrane) nmb  Ccl5 - chemokine (c-c motif) ligand 5  Csf1 - colony stimulating factor 1 (macrophage)  Ccl2 - chemokine (c-c motif) ligand 2  Ccl17 - chemokine (c-c motif) ligand 17  Alox15 - arachidonate 15-lipoxygenase  Slc11a1 - solute carrier family 11 (proton-coupled divalent metal ion transporters), member 1  Mmp8 - matrix metallopeptidase 8  Zeb2 - zinc finger e-box binding homeobox 2  Inhba - inhibin beta-a  Il1rn - interleukin 1 receptor antagonist  Acvr1 - activin a receptor, type 1  Nenf - neuron derived neurotrophic factor |
|  |  |  |  |  | Cxcl2 - chemokine (c-x-c motif) ligand 2  Ccl2 - chemokine (c-c motif) ligand 2  Lgals3 - lectin, galactose binding, soluble 3 |

| GO:0030595 | leukocyte chemotaxis | 9.35E-5 | 7.18E-3 | 5.60 (15721,122,184,8) | Ccl17 - chemokine (c-c motif) ligand 17 P  de4b - phosphodiesterase 4b, camp specific  Ccl3 - chemokine (c-c motif) ligand 3  Ccl4 - chemokine (c-c motif) ligand 4  Ccl5 - chemokine (c-c motif) ligand 5 |
| --- | --- | --- | --- | --- | --- |
| GO:0010562 | positive regulation of phosphorus metabolic process | 1.04E-4 | 7.98E-3 | 2.18 (15721,1058,184,27) | Trf - transferrin  Icam1 - intercellular adhesion molecule 1  Mtmr9 - myotubularin related protein 9  Trem2 - triggering receptor expressed on myeloid cells 2  Fgr - gardner-rasheed feline sarcoma viral (fgr) oncogene homolog Akap11 - a kinase (prka) anchor protein 11  Fpr2 - formyl peptide receptor 2  Cd74 - cd74 antigen (invariant polypeptide of major histocompatibility complex, class ii antigen-associated)  Csf1r - colony stimulating factor 1 receptor  Csf2 - colony stimulating factor 2 (granulocyte-macrophage)  Avpi1 - arginine vasopressin-induced 1  Ccl3 - chemokine (c-c motif) ligand 3  Dab2 - disabled 2, mitogen-responsive phosphoprotein  Ccl4 - chemokine (c-c motif) ligand 4  Gpnmb - glycoprotein (transmembrane) nmb  Ccl5 - chemokine (c-c motif) ligand 5  Csf1 - colony stimulating factor 1 (macrophage)  Ccl2 - chemokine (c-c motif) ligand 2  Ccl17 - chemokine (c-c motif) ligand 17  Alox15 - arachidonate 15-lipoxygenase  Slc11a1 - solute carrier family 11 (proton-coupled divalent metal ion transporters), member 1  Mmp8 - matrix metallopeptidase 8  Zeb2 - zinc finger e-box binding homeobox 2  Inhba - inhibin beta-a  Il1rn - interleukin 1 receptor antagonist  Acvr1 - activin a receptor, type 1  Nenf - neuron derived neurotrophic factor |
| GO:0045937 | positive regulation of phosphate metabolic process | 1.04E-4 | 7.94E-3 | 2.18 (15721,1058,184,27) | Trf - transferrin  Icam1 - intercellular adhesion molecule 1  Mtmr9 - myotubularin related protein 9  Trem2 - triggering receptor expressed on myeloid cells 2  Fgr - gardner-rasheed feline sarcoma viral (fgr) oncogene homolog Akap11 - a kinase (prka) anchor protein 11  Fpr2 - formyl peptide receptor 2  Cd74 - cd74 antigen (invariant polypeptide of major histocompatibility complex, class ii antigen-associated)  Csf1r - colony stimulating factor 1 receptor  Csf2 - colony stimulating factor 2 (granulocyte-macrophage)  Avpi1 - arginine vasopressin-induced 1  Ccl3 - chemokine (c-c motif) ligand 3  Dab2 - disabled 2, mitogen-responsive phosphoprotein  Ccl4 - chemokine (c-c motif) ligand 4  Gpnmb - glycoprotein (transmembrane) nmb  Ccl5 - chemokine (c-c motif) ligand 5  Csf1 - colony stimulating factor 1 (macrophage)  Ccl2 - chemokine (c-c motif) ligand 2  Ccl17 - chemokine (c-c motif) ligand 17  Alox15 - arachidonate 15-lipoxygenase  Slc11a1 - solute carrier family 11 (proton-coupled divalent metal ion transporters), member 1  Mmp8 - matrix metallopeptidase 8  Zeb2 - zinc finger e-box binding homeobox 2  Inhba - inhibin beta-a  Il1rn - interleukin 1 receptor antagonist  Acvr1 - activin a receptor, type 1  Nenf - neuron derived neurotrophic factor |
| GO:0002761 | regulation of myeloid leukocyte differentiation | 1.05E-4 | 7.93E-3 | 5.51 (15721,124,184,8) | Cd74 - cd74 antigen (invariant polypeptide of major histocompatibility complex, class ii antigen-associated)  Csf1r - colony stimulating factor 1 receptor  Ccl3 - chemokine (c-c motif) ligand 3  Inpp4b - inositol polyphosphate-4-phosphatase, type ii  Trem2 - triggering receptor expressed on myeloid cells 2  Runx1 - runt related transcription factor 1  Csf1 - colony stimulating factor 1 (macrophage)  Ccl5 - chemokine (c-c motif) ligand 5 |
|  |  |  |  |  | Ccl2 - chemokine (c-c motif) ligand 2  Pde4b - phosphodiesterase 4b, camp specific |

| GO:0071396 | cellular response to lipid | 1.2E-4 | 9.06E-3 | 3.42 (15721,325,184,13) | Mrc1 - mannose receptor, c type 1  Tnfrsf1b - tumor necrosis factor receptor superfamily, member 1b Sirpa - signal-regulatory protein alpha  Casp1 - caspase 1 Cd274 - cd274 antigen  Cxcl2 - chemokine (c-x-c motif) ligand 2  Inhba - inhibin beta-a  Mmp2 - matrix metallopeptidase 2  Irg1 - immunoresponsive gene 1  Gch1 - gtp cyclohydrolase 1  Tnfaip3 - tumor necrosis factor, alpha-induced protein 3 |
| --- | --- | --- | --- | --- | --- |
| GO:0010646 | regulation of cell communication | 1.23E-4 | 9.24E-3 | 1.59 (15721,3062,184,57) | Mgll - monoglyceride lipase  Trf - transferrin  Fpr2 - formyl peptide receptor 2  Prkcb - protein kinase c, beta  Cd74 - cd74 antigen (invariant polypeptide of major histocompatibility complex, class ii antigen-associated)  Socs3 - suppressor of cytokine signaling 3  Dab2 - disabled 2, mitogen-responsive phosphoprotein  Mmp12 - matrix metallopeptidase 12  H2-M3 - histocompatibility 2, m region locus 3  P2rx4 - purinergic receptor p2x, ligand-gated ion channel 4  Gpnmb - glycoprotein (transmembrane) nmb  Neto2 - neuropilin (nrp) and tolloid (tll)-like 2  Ccl2 - chemokine (c-c motif) ligand 2  Pde4b - phosphodiesterase 4b, camp specific  Ccl17 - chemokine (c-c motif) ligand 17  Clec4n - c-type lectin domain family 4, member n  Alox15 - arachidonate 15-lipoxygenase  Fabp5 - fatty acid binding protein 5, epidermal  Mmp8 - matrix metallopeptidase 8  Padi2 - peptidyl arginine deiminase, type ii  Acvr1 - activin a receptor, type 1  Nenf - neuron derived neurotrophic factor  Lpxn - leupaxin  Tnfaip3 - tumor necrosis factor, alpha-induced protein 3  Chst11 - carbohydrate sulfotransferase 11  Ikbke - inhibitor of kappab kinase epsilon  Icam1 - intercellular adhesion molecule 1  Acsl4 - acyl-coa synthetase long-chain family member 4  Rnf157 - ring finger protein 157  Inpp4b - inositol polyphosphate-4-phosphatase, type ii  Trem2 - triggering receptor expressed on myeloid cells 2  Fgr - gardner-rasheed feline sarcoma viral (fgr) oncogene homolog Il1rl1 - interleukin 1 receptor-like 1  Slc15a3 - solute carrier family 15, member 3  Csf1r - colony stimulating factor 1 receptor  Csf2 - colony stimulating factor 2 (granulocyte-macrophage)  Avpi1 - arginine vasopressin-induced 1  Fam20c - family with sequence similarity 20, member c  Ccl3 - chemokine (c-c motif) ligand 3  Ccl4 - chemokine (c-c motif) ligand 4  Irg1 - immunoresponsive gene 1  Bmi1 - bmi1 polycomb ring finger oncogene  Csf1 - colony stimulating factor 1 (macrophage)  Ccl5 - chemokine (c-c motif) ligand 5  Igfbp7 - insulin-like growth factor binding protein 7  Lgals3 - lectin, galactose binding, soluble 3  Il7r - interleukin 7 receptor  Ndrg2 - n-myc downstream regulated gene 2  Sirpa - signal-regulatory protein alpha  Casp1 - caspase 1  Thy1 - thymus cell antigen 1, theta  Zeb2 - zinc finger e-box binding homeobox 2  Tgm2 - transglutaminase 2, c polypeptide  Inhba - inhibin beta-a  Kdm5d - lysine (k)-specific demethylase 5d  Il1rn - interleukin 1 receptor antagonist  Runx1 - runt related transcription factor 1 |
| GO:0043277 | apoptotic cell clearance | 1.35E-4 | 1.01E-2 | 14.86 (15721,23,184,4) | Tgm2 - transglutaminase 2, c polypeptide  Alox15 - arachidonate 15-lipoxygenase  Trem2 - triggering receptor expressed on myeloid cells 2  Mfge8 - milk fat globule-egf factor 8 protein |
|  |  |  |  |  | Lgals3 - lectin, galactose binding, soluble 3  H2-Ab1 - histocompatibility 2, class ii antigen a, beta 1  Tnfrsf1b - tumor necrosis factor receptor superfamily, member 1b |

| GO:0042129 | regulation of T cell proliferation | 1.35E-4 | 1E-2 | 4.69 (15721,164,184,9) | H2-Aa - histocompatibility 2, class ii antigen a, alpha  H2-M3 - histocompatibility 2, m region locus 3  Cd274 - cd274 antigen  Bmi1 - bmi1 polycomb ring finger oncogene  Ccl5 - chemokine (c-c motif) ligand 5  Gpnmb - glycoprotein (transmembrane) nmb |
| --- | --- | --- | --- | --- | --- |
| GO:0072573 | tolerance induction to lipopoly-saccharide | 1.36E-4 | 1.01E-2 | 85.44 (15721,2,184,2) | Irg1 - immunoresponsive gene 1  Tnfaip3 - tumor necrosis factor, alpha-induced protein 3 |
| GO:0023051 | regulation of signaling | 1.4E-4 | 1.03E-2 | 1.58 (15721,3076,184,57) | Mgll - monoglyceride lipase  Trf - transferrin  Fpr2 - formyl peptide receptor 2  Cd74 - cd74 antigen (invariant polypeptide of major histocompatibility complex, class ii antigen-associated)  Prkcb - protein kinase c, beta  Socs3 - suppressor of cytokine signaling 3  Dab2 - disabled 2, mitogen-responsive phosphoprotein  Mmp12 - matrix metallopeptidase 12  H2-M3 - histocompatibility 2, m region locus 3  P2rx4 - purinergic receptor p2x, ligand-gated ion channel 4  Gpnmb - glycoprotein (transmembrane) nmb  Neto2 - neuropilin (nrp) and tolloid (tll)-like 2  Ccl2 - chemokine (c-c motif) ligand 2  Pde4b - phosphodiesterase 4b, camp specific  Ccl17 - chemokine (c-c motif) ligand 17  Clec4n - c-type lectin domain family 4, member n  Alox15 - arachidonate 15-lipoxygenase  Fabp5 - fatty acid binding protein 5, epidermal  Mmp8 - matrix metallopeptidase 8  Padi2 - peptidyl arginine deiminase, type ii  Acvr1 - activin a receptor, type 1  Nenf - neuron derived neurotrophic factor  Lpxn - leupaxin  Tnfaip3 - tumor necrosis factor, alpha-induced protein 3  Chst11 - carbohydrate sulfotransferase 11  Ikbke - inhibitor of kappab kinase epsilon  Icam1 - intercellular adhesion molecule 1  Acsl4 - acyl-coa synthetase long-chain family member 4  Rnf157 - ring finger protein 157  Inpp4b - inositol polyphosphate-4-phosphatase, type ii  Trem2 - triggering receptor expressed on myeloid cells 2  Fgr - gardner-rasheed feline sarcoma viral (fgr) oncogene homolog Il1rl1 - interleukin 1 receptor-like 1  Slc15a3 - solute carrier family 15, member 3  Csf1r - colony stimulating factor 1 receptor  Csf2 - colony stimulating factor 2 (granulocyte-macrophage)  Avpi1 - arginine vasopressin-induced 1  Fam20c - family with sequence similarity 20, member c  Ccl3 - chemokine (c-c motif) ligand 3  Ccl4 - chemokine (c-c motif) ligand 4  Irg1 - immunoresponsive gene 1  Bmi1 - bmi1 polycomb ring finger oncogene  Csf1 - colony stimulating factor 1 (macrophage)  Ccl5 - chemokine (c-c motif) ligand 5  Igfbp7 - insulin-like growth factor binding protein 7  Lgals3 - lectin, galactose binding, soluble 3  Il7r - interleukin 7 receptor  Ndrg2 - n-myc downstream regulated gene 2  Casp1 - caspase 1  Sirpa - signal-regulatory protein alpha  Thy1 - thymus cell antigen 1, theta  Zeb2 - zinc finger e-box binding homeobox 2  Inhba - inhibin beta-a  Tgm2 - transglutaminase 2, c polypeptide  Kdm5d - lysine (k)-specific demethylase 5d  Il1rn - interleukin 1 receptor antagonist  Runx1 - runt related transcription factor 1 |
|  |  |  |  |  | Trf - transferrin  Icam1 - intercellular adhesion molecule 1  Trem2 - triggering receptor expressed on myeloid cells 2  Fgr - gardner-rasheed feline sarcoma viral (fgr) oncogene homolog Akap11 - a kinase (prka) anchor protein 11  Fpr2 - formyl peptide receptor 2  Cd74 - cd74 antigen (invariant polypeptide of major histocompatibility complex, class ii antigen-associated)  Csf1r - colony stimulating factor 1 receptor  Csf2 - colony stimulating factor 2 (granulocyte-macrophage) |

| GO:0001932 | regulation of protein phosphorylation | 1.45E-4 | 1.06E-2 | 2.00 (15721,1322,184,31) | Socs3 - suppressor of cytokine signaling 3  Avpi1 - arginine vasopressin-induced 1  Ccl3 - chemokine (c-c motif) ligand 3  Dab2 - disabled 2, mitogen-responsive phosphoprotein  Ccl4 - chemokine (c-c motif) ligand 4  Gpnmb - glycoprotein (transmembrane) nmb  Ccl5 - chemokine (c-c motif) ligand 5  Csf1 - colony stimulating factor 1 (macrophage)  Ccl2 - chemokine (c-c motif) ligand 2  Ccl17 - chemokine (c-c motif) ligand 17  Ndrg2 - n-myc downstream regulated gene 2  Sirpa - signal-regulatory protein alpha  Alox15 - arachidonate 15-lipoxygenase  Thy1 - thymus cell antigen 1, theta  Slc11a1 - solute carrier family 11 (proton-coupled divalent metal ion transporters), member 1  Mmp8 - matrix metallopeptidase 8  Zeb2 - zinc finger e-box binding homeobox 2  Inhba - inhibin beta-a  Il1rn - interleukin 1 receptor antagonist  Acvr1 - activin a receptor, type 1  Nenf - neuron derived neurotrophic factor  Tnfaip3 - tumor necrosis factor, alpha-induced protein 3 |
| --- | --- | --- | --- | --- | --- |
| GO:0042592 | homeostatic process | 1.61E-4 | 1.17E-2 | 1.94 (15721,1455,184,33) | Cebpa - ccaat/enhancer binding protein (c/ebp), alpha  Trf - transferrin  Icam1 - intercellular adhesion molecule 1  Inpp4b - inositol polyphosphate-4-phosphatase, type ii  Slc6a12 - solute carrier family 6 (neurotransmitter transporter, betaine/gaba), member 12  Fpr1 - formyl peptide receptor 1  Akap11 - a kinase (prka) anchor protein 11  Fpr2 - formyl peptide receptor 2  Cxcl2 - chemokine (c-x-c motif) ligand 2  Prkcb - protein kinase c, beta  Snx10 - sorting nexin 10  Ccl3 - chemokine (c-c motif) ligand 3  P2rx4 - purinergic receptor p2x, ligand-gated ion channel 4  Bmi1 - bmi1 polycomb ring finger oncogene  Ccl5 - chemokine (c-c motif) ligand 5  Csf1 - colony stimulating factor 1 (macrophage)  Ctss - cathepsin s  Ccl2 - chemokine (c-c motif) ligand 2  Pde4b - phosphodiesterase 4b, camp specific  Ccr5 - chemokine (c-c motif) receptor 5  Il7r - interleukin 7 receptor  Cp - ceruloplasmin  Fabp5 - fatty acid binding protein 5, epidermal  Thy1 - thymus cell antigen 1, theta  Slc11a1 - solute carrier family 11 (proton-coupled divalent metal ion transporters), member 1  Atp6v0d2 - atpase, h+ transporting, lysosomal v0 subunit d2  Tgm2 - transglutaminase 2, c polypeptide  Cd34 - cd34 antigen  Il1rn - interleukin 1 receptor antagonist  Pygl - liver glycogen phosphorylase  P2ry14 - purinergic receptor p2y, g-protein coupled, 14  Ccrl2 - chemokine (c-c motif) receptor-like 2  Tnfaip3 - tumor necrosis factor, alpha-induced protein 3 |
| GO:0044057 | regulation of system process | 1.61E-4 | 1.16E-2 | 2.65 (15721,580,184,18) | Itgax - integrin alpha x  Mgll - monoglyceride lipase  Pde4b - phosphodiesterase 4b, camp specific  Dsc2 - desmocollin 2  Tnfrsf1b - tumor necrosis factor receptor superfamily, member 1b Icam1 - intercellular adhesion molecule 1  Trf - transferrin  Fabp5 - fatty acid binding protein 5, epidermal  Cst7 - cystatin f (leukocystatin)  Mmp2 - matrix metallopeptidase 2  Inhba - inhibin beta-a  Mmp13 - matrix metallopeptidase 13  Dab2 - disabled 2, mitogen-responsive phosphoprotein  Gch1 - gtp cyclohydrolase 1  P2rx4 - purinergic receptor p2x, ligand-gated ion channel 4  Runx1 - runt related transcription factor 1  Ctss - cathepsin s  Egr2 - early growth response 2 |
|  |  |  |  |  |  |

| GO:0035821 | modification of morphology or physiology of other organism | 1.62E-4 | 1.17E-2 | 5.18 (15721,132,184,8) | Cxcl2 - chemokine (c-x-c motif) ligand 2  Ccl2 - chemokine (c-c motif) ligand 2  Lgals3 - lectin, galactose binding, soluble 3  Csf1r - colony stimulating factor 1 receptor  Ccl17 - chemokine (c-c motif) ligand 17  Ccr5 - chemokine (c-c motif) receptor 5  Ccl4 - chemokine (c-c motif) ligand 4  Ccl5 - chemokine (c-c motif) ligand 5 |
| --- | --- | --- | --- | --- | --- |
| GO:0031343 | positive regulation of cell killing | 1.67E-4 | 1.2E-2 | 7.32 (15721,70,184,6) | Ccl2 - chemokine (c-c motif) ligand 2  H2-D1 - histocompatibility 2, d region locus 1  Ccr5 - chemokine (c-c motif) receptor 5  Clec7a - c-type lectin domain family 7, member a  H2-M3 - histocompatibility 2, m region locus 3  H2-K1 - histocompatibility 2, k1, k region |
| GO:0006897 | endocytosis | 1.75E-4 | 1.25E-2 | 3.12 (15721,384,184,14) | Mrc1 - mannose receptor, c type 1  Mtmr9 - myotubularin related protein 9  Alox15 - arachidonate 15-lipoxygenase  Myo7a - myosin viia  Trem2 - triggering receptor expressed on myeloid cells 2  Slc11a1 - solute carrier family 11 (proton-coupled divalent metal ion transporters), member 1  Hck - hemopoietic cell kinase  Fpr2 - formyl peptide receptor 2  Cd9 - cd9 antigen  Tgm2 - transglutaminase 2, c polypeptide  Snx10 - sorting nexin 10  Dab2 - disabled 2, mitogen-responsive phosphoprotein  Pld4 - phospholipase d family, member 4  Mfge8 - milk fat globule-egf factor 8 protein |
| GO:0032649 | regulation of interferon-gamma production | 1.83E-4 | 1.3E-2 | 5.92 (15721,101,184,7) | Pde4b - phosphodiesterase 4b, camp specific  Sirpa - signal-regulatory protein alpha  H2-M3 - histocompatibility 2, m region locus 3  Cd274 - cd274 antigen  Slc11a1 - solute carrier family 11 (proton-coupled divalent metal ion transporters), member 1  Runx1 - runt related transcription factor 1  Il1rl1 - interleukin 1 receptor-like 1 |
| GO:0031341 | regulation of cell killing | 1.83E-4 | 1.29E-2 | 5.92 (15721,101,184,7) | Ccl2 - chemokine (c-c motif) ligand 2  H2-D1 - histocompatibility 2, d region locus 1  Ccr5 - chemokine (c-c motif) receptor 5  Il7r - interleukin 7 receptor  Clec7a - c-type lectin domain family 7, member a  H2-M3 - histocompatibility 2, m region locus 3  H2-K1 - histocompatibility 2, k1, k region |
| GO:0071677 | positive regulation of mononuclear cell migration | 1.9E-4 | 1.33E-2 | 13.67 (15721,25,184,4) | Ccl2 - chemokine (c-c motif) ligand 2  Lgals3 - lectin, galactose binding, soluble 3  Ccl5 - chemokine (c-c motif) ligand 5  Fpr2 - formyl peptide receptor 2 |
| GO:0034114 | regulation of heterotypic cell-cell adhesion | 1.9E-4 | 1.33E-2 | 13.67 (15721,25,184,4) | Il1rn - interleukin 1 receptor antagonist  Alox15 - arachidonate 15-lipoxygenase  Thy1 - thymus cell antigen 1, theta  Tnfaip3 - tumor necrosis factor, alpha-induced protein 3 |
| GO:0035456 | response to interferon-beta | 1.93E-4 | 1.35E-2 | 9.29 (15721,46,184,5) | Ifi205 - interferon activated gene 205  Ifi203 - interferon activated gene 203  Ikbke - inhibitor of kappab kinase epsilon  Ifi202b - interferon activated gene 202b  Irg1 - immunoresponsive gene 1 |
| GO:0045670 | regulation of osteoclast differentiation | 1.95E-4 | 1.36E-2 | 7.12 (15721,72,184,6) | Csf1r - colony stimulating factor 1 receptor  Ccl3 - chemokine (c-c motif) ligand 3  Inpp4b - inositol polyphosphate-4-phosphatase, type ii  Trem2 - triggering receptor expressed on myeloid cells 2  Ccl5 - chemokine (c-c motif) ligand 5  Csf1 - colony stimulating factor 1 (macrophage) |
| GO:0043032 | positive regulation of macrophage activation | 2.22E-4 | 1.53E-2 | 13.14 (15721,26,184,4) | Cebpa - ccaat/enhancer binding protein (c/ebp), alpha  Trem2 - triggering receptor expressed on myeloid cells 2 |

|  |  |  |  |  | Mmp8 - matrix metallopeptidase 8  Il1rl1 - interleukin 1 receptor-like 1 |
| --- | --- | --- | --- | --- | --- |
| GO:0045807 | positive regulation of endocytosis | 2.3E-4 | 1.58E-2 | 4.37 (15721,176,184,9) | Ccl2 - chemokine (c-c motif) ligand 2  Clec7a - c-type lectin domain family 7, member a  Trf - transferrin  Sirpa - signal-regulatory protein alpha  Dab2 - disabled 2, mitogen-responsive phosphoprotein  Trem2 - triggering receptor expressed on myeloid cells 2  Slc11a1 - solute carrier family 11 (proton-coupled divalent metal ion transporters), member 1  Mfge8 - milk fat globule-egf factor 8 protein  Fpr2 - formyl peptide receptor 2 |
| GO:0051049 | regulation of transport | 2.51E-4 | 1.72E-2 | 1.81 (15721,1750,184,37) | Cyp4f18 - cytochrome p450, family 4, subfamily f, polypeptide 18 Icam1 - intercellular adhesion molecule 1  Trf - transferrin  Acsl4 - acyl-coa synthetase long-chain family member 4  Inpp4b - inositol polyphosphate-4-phosphatase, type ii  Siglec5 - sialic acid binding ig-like lectin 5  Trem2 - triggering receptor expressed on myeloid cells 2  Fgr - gardner-rasheed feline sarcoma viral (fgr) oncogene homolog Fpr2 - formyl peptide receptor 2  Hck - hemopoietic cell kinase  Cd74 - cd74 antigen (invariant polypeptide of major histocompatibility complex, class ii antigen-associated)  Prkcb - protein kinase c, beta  Mmp13 - matrix metallopeptidase 13  Clec7a - c-type lectin domain family 7, member a  Dab2 - disabled 2, mitogen-responsive phosphoprotein  Ccl4 - chemokine (c-c motif) ligand 4  P2rx4 - purinergic receptor p2x, ligand-gated ion channel 4  Ccl5 - chemokine (c-c motif) ligand 5  Neto2 - neuropilin (nrp) and tolloid (tll)-like 2  Ctss - cathepsin  Ccl2 - chemokine (c-c motif) ligand 2  Pde4b - phosphodiesterase 4b, camp specific  Lgals3 - lectin, galactose binding, soluble 3  Tnfrsf1b - tumor necrosis factor receptor superfamily, member 1b Sirpa - signal-regulatory protein alpha  Alox15 - arachidonate 15-lipoxygenase  Fabp5 - fatty acid binding protein 5, epidermal  Ifi27 - interferon, alpha-inducible protein 27  Slc11a1 - solute carrier family 11 (proton-coupled divalent metal ion transporters), member 1  Sestd1 - sec14 and spectrin domains 1  Thy1 - thymus cell antigen 1, theta  Inhba - inhibin beta-a  Cd34 - cd34 antigen  Il1rn - interleukin 1 receptor antagonist  Stxbp6 - syntaxin binding protein 6 (amisyn)  Runx1 - runt related transcription factor 1  Mfge8 - milk fat globule-egf factor 8 protein |
| GO:0050670 | regulation of lymphocyte proliferation | 2.51E-4 | 1.71E-2 | 3.92 (15721,218,184,10) | Cd74 - cd74 antigen (invariant polypeptide of major histocompatibility complex, class ii antigen-associated)  Lgals3 - lectin, galactose binding, soluble 3  H2-Ab1 - histocompatibility 2, class ii antigen a, beta 1  Tnfrsf1b - tumor necrosis factor receptor superfamily, member 1b H2-Aa - histocompatibility 2, class ii antigen a, alpha  H2-M3 - histocompatibility 2, m region locus 3  Cd274 - cd274 antigen  Bmi1 - bmi1 polycomb ring finger oncogene  Gpnmb - glycoprotein (transmembrane) nmb  Ccl5 - chemokine (c-c motif) ligand 5 |
| GO:0031348 | negative regulation of defense response | 2.51E-4 | 1.71E-2 | 3.92 (15721,218,184,10) | Socs3 - suppressor of cytokine signaling 3  Tnfrsf1b - tumor necrosis factor receptor superfamily, member 1b Sirpa - signal-regulatory protein alpha  Mmp12 - matrix metallopeptidase 12  H2-M3 - histocompatibility 2, m region locus 3  Irg1 - immunoresponsive gene 1  Cst7 - cystatin f (leukocystatin)  Fgl2 - fibrinogen-like protein 2  Tnfaip3 - tumor necrosis factor, alpha-induced protein 3  Fpr2 - formyl peptide receptor 2 |
|  |  |  |  |  | Zmat3 - zinc finger matrin type 3 |

| GO:0042981 | regulation of apoptotic process | 2.55E-4 | 1.72E-2 | 1.91 (15721,1428,184,32) | Chst11 - carbohydrate sulfotransferase 11  Icam1 - intercellular adhesion molecule 1  Rnf157 - ring finger protein 157  Acot1 - acyl-coa thioesterase 1  Trem2 - triggering receptor expressed on myeloid cells 2  Cd274 - cd274 antigen  Fpr2 - formyl peptide receptor 2  Hck - hemopoietic cell kinase  Cd74 - cd74 antigen (invariant polypeptide of major histocompatibility complex, class ii antigen-associated)  Mmp2 - matrix metallopeptidase 2  Csf1r - colony stimulating factor 1 receptor  Csf2 - colony stimulating factor 2 (granulocyte-macrophage)  Socs3 - suppressor of cytokine signaling 3  Ccl3 - chemokine (c-c motif) ligand 3  Dab2 - disabled 2, mitogen-responsive phosphoprotein  H2-M3 - histocompatibility 2, m region locus 3  Degs1 - degenerative spermatocyte homolog 1 (drosophila)  Bmi1 - bmi1 polycomb ring finger oncogene  Ccl5 - chemokine (c-c motif) ligand 5  Ccr5 - chemokine (c-c motif) receptor 5  Lgals3 - lectin, galactose binding, soluble 3  Il7r - interleukin 7 receptor  Tnfrsf1b - tumor necrosis factor receptor superfamily, member 1b Casp1 - caspase 1  Tgm2 - transglutaminase 2, c polypeptide  Inhba - inhibin beta-a  Il1rn - interleukin 1 receptor antagonist  Serpinb2 - serine (or cysteine) peptidase inhibitor, clade b, member 2 Acvr1 - activin a receptor, type 1  Tnfaip3 - tumor necrosis factor, alpha-induced protein 3  Egr3 - early growth response 3 |
| --- | --- | --- | --- | --- | --- |
| GO:0002474 | antigen processing and presentation of peptide antigen via MHC class I | 2.58E-4 | 1.74E-2 | 12.66 (15721,27,184,4) | H2-D1 - histocompatibility 2, d region locus 1  H2-M3 - histocompatibility 2, m region locus 3  H2-K1 - histocompatibility 2, k1, k region  Ifi30 - interferon gamma inducible protein 30 |
| GO:0002824 | positive regulation of adaptive immune response based on somatic recombination of immune receptors built from immunoglobulin superfamily domains | 2.93E-4 | 1.96E-2 | 5.49 (15721,109,184,7) | H2-D1 - histocompatibility 2, d region locus 1  H2-Ab1 - histocompatibility 2, class ii antigen a, beta 1  H2-M3 - histocompatibility 2, m region locus 3  Cd274 - cd274 antigen  Slc11a1 - solute carrier family 11 (proton-coupled divalent metal ion transporters), member 1  H2-DMa - histocompatibility 2, class ii, locus dma  H2-K1 - histocompatibility 2, k1, k region |
| GO:0045089 | positive regulation of innate immune response | 2.95E-4 | 1.97E-2 | 4.23 (15721,182,184,9) | Ifi205 - interferon activated gene 205  Cd74 - cd74 antigen (invariant polypeptide of major histocompatibility complex, class ii antigen-associated)  Slc15a3 - solute carrier family 15, member 3  Mmp2 - matrix metallopeptidase 2  Ifi203 - interferon activated gene 203  Ikbke - inhibitor of kappab kinase epsilon  Mmp12 - matrix metallopeptidase 12  H2-M3 - histocompatibility 2, m region locus 3  Fpr2 - formyl peptide receptor 2 |
| GO:0055082 | cellular chemical homeostasis | 3.32E-4 | 2.2E-2 | 2.42 (15721,670,184,19) | Ccl2 - chemokine (c-c motif) ligand 2  Ccr5 - chemokine (c-c motif) receptor 5  Cp - ceruloplasmin  Icam1 - intercellular adhesion molecule 1  Trf - transferrin  Inpp4b - inositol polyphosphate-4-phosphatase, type ii  Slc6a12 - solute carrier family 6 (neurotransmitter transporter, betaine/gaba), member 12  Slc11a1 - solute carrier family 11 (proton-coupled divalent metal ion transporters), member 1  Fpr1 - formyl peptide receptor 1  Thy1 - thymus cell antigen 1, theta  Atp6v0d2 - atpase, h+ transporting, lysosomal v0 subunit d2  Fpr2 - formyl peptide receptor 2  Prkcb - protein kinase c, beta  Cxcl2 - chemokine (c-x-c motif) ligand 2  Tgm2 - transglutaminase 2, c polypeptide  Ccl3 - chemokine (c-c motif) ligand 3  Ccrl2 - chemokine (c-c motif) receptor-like 2  P2rx4 - purinergic receptor p2x, ligand-gated ion channel 4  Ccl5 - chemokine (c-c motif) ligand 5 |

|  |  |  |  |  |  |
| --- | --- | --- | --- | --- | --- |
| GO:0048585 | negative regulation of response to stimulus | 3.34E-4 | 2.21E-2 | 1.86 (15721,1514,184,33) | Chst11 - carbohydrate sulfotransferase 11  Icam1 - intercellular adhesion molecule 1  Rnf157 - ring finger protein 157  Cst7 - cystatin f (leukocystatin)  Fgl2 - fibrinogen-like protein 2  Il1rl1 - interleukin 1 receptor-like 1  Fpr2 - formyl peptide receptor 2  Cd74 - cd74 antigen (invariant polypeptide of major histocompatibility complex, class ii antigen-associated)  Prkcb - protein kinase c, beta  Cd9 - cd9 antigen  Csf2 - colony stimulating factor 2 (granulocyte-macrophage)  Socs3 - suppressor of cytokine signaling 3  Dab2 - disabled 2, mitogen-responsive phosphoprotein  Mmp12 - matrix metallopeptidase 12  H2-M3 - histocompatibility 2, m region locus 3  Irg1 - immunoresponsive gene 1  Bmi1 - bmi1 polycomb ring finger oncogene  Ccl5 - chemokine (c-c motif) ligand 5  Pde4b - phosphodiesterase 4b, camp specific  Lgals3 - lectin, galactose binding, soluble 3  Il7r - interleukin 7 receptor  Ndrg2 - n-myc downstream regulated gene 2  Tnfrsf1b - tumor necrosis factor receptor superfamily, member 1b Sirpa - signal-regulatory protein alpha  Alox15 - arachidonate 15-lipoxygenase  Thy1 - thymus cell antigen 1, theta  Cd34 - cd34 antigen  Padi2 - peptidyl arginine deiminase, type ii  Il1rn - interleukin 1 receptor antagonist  Acvr1 - activin a receptor, type 1  Nenf - neuron derived neurotrophic factor  Lpxn - leupaxin  Tnfaip3 - tumor necrosis factor, alpha-induced protein 3 |
| GO:0043067 | regulation of programmed cell death | 3.39E-4 | 2.23E-2 | 1.88 (15721,1451,184,32) | Zmat3 - zinc finger matrin type 3  Chst11 - carbohydrate sulfotransferase 11  Icam1 - intercellular adhesion molecule 1  Rnf157 - ring finger protein 157  Acot1 - acyl-coa thioesterase 1  Trem2 - triggering receptor expressed on myeloid cells 2  Cd274 - cd274 antigen  Fpr2 - formyl peptide receptor 2  Hck - hemopoietic cell kinase  Cd74 - cd74 antigen (invariant polypeptide of major histocompatibility complex, class ii antigen-associated)  Mmp2 - matrix metallopeptidase 2  Csf1r - colony stimulating factor 1 receptor  Csf2 - colony stimulating factor 2 (granulocyte-macrophage)  Socs3 - suppressor of cytokine signaling 3  Ccl3 - chemokine (c-c motif) ligand 3  Dab2 - disabled 2, mitogen-responsive phosphoprotein  H2-M3 - histocompatibility 2, m region locus 3  Degs1 - degenerative spermatocyte homolog 1 (drosophila)  Bmi1 - bmi1 polycomb ring finger oncogene  Ccl5 - chemokine (c-c motif) ligand 5  Ccr5 - chemokine (c-c motif) receptor 5  Lgals3 - lectin, galactose binding, soluble 3  Il7r - interleukin 7 receptor  Tnfrsf1b - tumor necrosis factor receptor superfamily, member 1b Casp1 - caspase 1  Tgm2 - transglutaminase 2, c polypeptide  Inhba - inhibin beta-a  Il1rn - interleukin 1 receptor antagonist  Serpinb2 - serine (or cysteine) peptidase inhibitor, clade b, member 2  Acvr1 - activin a receptor, type 1  Tnfaip3 - tumor necrosis factor, alpha-induced protein 3  Egr3 - early growth response 3 |
| GO:0010758 | regulation of macrophage chemotaxis | 3.43E-4 | 2.25E-2 | 11.78 (15721,29,184,4) | Ccl2 - chemokine (c-c motif) ligand 2  Csf1r - colony stimulating factor 1 receptor  Ccl5 - chemokine (c-c motif) ligand 5  Csf1 - colony stimulating factor 1 (macrophage) |
| GO:0043030 | regulation of macrophage activation | 3.46E-4 | 2.26E-2 | 8.22 (15721,52,184,5) | Cebpa - ccaat/enhancer binding protein (c/ebp), alpha  Trem2 - triggering receptor expressed on myeloid cells 2  Cst7 - cystatin f (leukocystatin) |

|  |  |  |  |  | Il1rl1 - interleukin 1 receptor-like 1  Mmp8 - matrix metallopeptidase 8 |
| --- | --- | --- | --- | --- | --- |
| GO:0008285 | negative regulation of cell proliferation | 3.64E-4 | 2.37E-2 | 2.40 (15721,675,184,19) | Ccr5 - chemokine (c-c motif) receptor 5  Lgals3 - lectin, galactose binding, soluble 3  Ndrg2 - n-myc downstream regulated gene 2  H2-Ab1 - histocompatibility 2, class ii antigen a, beta 1  Cebpa - ccaat/enhancer binding protein (c/ebp), alpha  H2-Aa - histocompatibility 2, class ii antigen a, alpha  Tnfrsf9 - tumor necrosis factor receptor superfamily, member 9 Cd274 - cd274 antigen  Il1rl1 - interleukin 1 receptor-like 1  Ifi30 - interferon gamma inducible protein 30  Csf1r - colony stimulating factor 1 receptor  Inhba - inhibin beta-a  Cd9 - cd9 antigen  Dab2 - disabled 2, mitogen-responsive phosphoprotein  H2-M3 - histocompatibility 2, m region locus 3  Dach1 - dachshund 1 (drosophila)  Runx1 - runt related transcription factor 1  Gpnmb - glycoprotein (transmembrane) nmb  Tnfaip3 - tumor necrosis factor, alpha-induced protein 3 |
| GO:0042325 | regulation of phosphorylation | 3.65E-4 | 2.36E-2 | 1.88 (15721,1457,184,32) | Trf - transferrin  Icam1 - intercellular adhesion molecule 1  Trem2 - triggering receptor expressed on myeloid cells 2  Fgr - gardner-rasheed feline sarcoma viral (fgr) oncogene homolog Akap11 - a kinase (prka) anchor protein 11  Fpr2 - formyl peptide receptor 2  Cd74 - cd74 antigen (invariant polypeptide of major histocompatibility complex, class ii antigen-associated)  Csf1r - colony stimulating factor 1 receptor  Csf2 - colony stimulating factor 2 (granulocyte-macrophage)  Socs3 - suppressor of cytokine signaling 3  Avpi1 - arginine vasopressin-induced 1  Ccl3 - chemokine (c-c motif) ligand 3  Dab2 - disabled 2, mitogen-responsive phosphoprotein  Ccl4 - chemokine (c-c motif) ligand 4  Gpnmb - glycoprotein (transmembrane) nmb  Ccl5 - chemokine (c-c motif) ligand 5  Csf1 - colony stimulating factor 1 (macrophage)  Slc2a6 - solute carrier family 2 (facilitated glucose transporter), member 6  Ccl2 - chemokine (c-c motif) ligand 2  Ccl17 - chemokine (c-c motif) ligand 17  Ndrg2 - n-myc downstream regulated gene 2  Sirpa - signal-regulatory protein alpha  Alox15 - arachidonate 15-lipoxygenase  Thy1 - thymus cell antigen 1, theta  Slc11a1 - solute carrier family 11 (proton-coupled divalent metal ion transporters), member 1  Mmp8 - matrix metallopeptidase 8  Zeb2 - zinc finger e-box binding homeobox 2  Inhba - inhibin beta-a  Il1rn - interleukin 1 receptor antagonist  Acvr1 - activin a receptor, type 1  Nenf - neuron derived neurotrophic factor  Tnfaip3 - tumor necrosis factor, alpha-induced protein 3 |
| GO:0007155 | cell adhesion | 3.86E-4 | 2.49E-2 | 2.33 (15721,734,184,20) | Itgax - integrin alpha x  Igfbp7 - insulin-like growth factor binding protein 7  Dsc2 - desmocollin 2  Sirpa - signal-regulatory protein alpha  Icam1 - intercellular adhesion molecule 1  Siglec5 - sialic acid binding ig-like lectin 5  Atrnl1 - attractin like 1  Thy1 - thymus cell antigen 1, theta  Cd34 - cd34 antigen  Cd9 - cd9 antigen  Acvr1 - activin a receptor, type 1  Lpp - lim domain containing preferred translocation partner in lipoma  Alcam - activated leukocyte cell adhesion molecule  Emb - embigin  Parvb - parvin, beta  Lgals3bp - lectin, galactoside-binding, soluble, 3 binding protein Lpxn – leupaxin  Gpnmb - glycoprotein (transmembrane) nmb  Ccl5 - chemokine (c-c motif) ligand 5  Mfge8 - milk fat globule-egf factor 8 protein |
|  |  |  |  |  |  |

| GO:0019724 | B cell mediated immunity | 3.92E-4 | 2.52E-2 | 11.39 (15721,30,184,4) | Cd74 - cd74 antigen (invariant polypeptide of major histocompatibility complex, class ii antigen-associated)  H2-Ab1 - histocompatibility 2, class ii antigen a, beta 1  H2-M3 - histocompatibility 2, m region locus 3  H2-DMa - histocompatibility 2, class ii, locus dma |
| --- | --- | --- | --- | --- | --- |
| GO:0072507 | divalent inorganic cation homeostasis | 4.01E-4 | 2.56E-2 | 2.74 (15721,467,184,15) | Ccl2 - chemokine (c-c motif) ligand 2  Ccr5 - chemokine (c-c motif) receptor 5  Inpp4b - inositol polyphosphate-4-phosphatase, type ii  Slc11a1 - solute carrier family 11 (proton-coupled divalent metal ion transporters), member 1  Fpr1 - formyl peptide receptor 1  Thy1 - thymus cell antigen 1, theta  Fpr2 - formyl peptide receptor 2  Prkcb - protein kinase c, beta  Cxcl2 - chemokine (c-x-c motif) ligand 2  Tgm2 - transglutaminase 2, c polypeptide  Snx10 - sorting nexin 10  Ccl3 - chemokine (c-c motif) ligand 3  Ccrl2 - chemokine (c-c motif) receptor-like 2  P2rx4 - purinergic receptor p2x, ligand-gated ion channel 4  Ccl5 - chemokine (c-c motif) ligand 5 |
| GO:0002215 | defense response to nematode | 4.06E-4 | 2.58E-2 | 56.96 (15721,3,184,2) | Epx - eosinophil peroxidase  Prg2 - proteoglycan 2, bone marrow |
| GO:0002485 | antigen processing and presentation of endogenous peptide antigen via MHC class I via ER pathway, TAP- dependent | 4.06E-4 | 2.57E-2 | 56.96 (15721,3,184,2) | H2-D1 - histocompatibility 2, d region locus 1  H2-K1 - histocompatibility 2, k1, k region |
| GO:1901074 | regulation of engulfment of apoptotic cell | 4.06E-4 | 2.56E-2 | 56.96 (15721,3,184,2) | Alox15 - arachidonate 15-lipoxygenase  Trem2 - triggering receptor expressed on myeloid cells 2 |
| GO:0038145 | macrophage colony-stimulating factor signaling pathway | 4.06E-4 | 2.55E-2 | 56.96 (15721,3,184,2) | Csf1r - colony stimulating factor 1 receptor  Csf1 - colony stimulating factor 1 (macrophage) |
| GO:0002286 | T cell activation involved in immune response | 4.13E-4 | 2.59E-2 | 7.91 (15721,54,184,5) | Cd74 - cd74 antigen (invariant polypeptide of major histocompatibility complex, class ii antigen-associated)  Icam1 - intercellular adhesion molecule 1  H2-M3 - histocompatibility 2, m region locus 3  Fgl2 - fibrinogen-like protein 2  Slc11a1 - solute carrier family 11 (proton-coupled divalent metal ion transporters), member 1 |
| GO:0042116 | macrophage activation | 4.13E-4 | 2.58E-2 | 7.91 (15721,54,184,5) | Csf2 - colony stimulating factor 2 (granulocyte-macrophage)  Casp1 - caspase 1  Trem2 - triggering receptor expressed on myeloid cells 2  Slc11a1 - solute carrier family 11 (proton-coupled divalent metal ion transporters), member 1  Fpr2 - formyl peptide receptor 2 |
| GO:0002486 | antigen processing and presentation of endogenous peptide antigen via MHC class I via ER pathway, TAP- independent | 4.14E-4 | 2.57E-2 | 19.72 (15721,13,184,3) | H2-D1 - histocompatibility 2, d region locus 1  H2-M3 - histocompatibility 2, m region locus 3  H2-K1 - histocompatibility 2, k1, k region |
| GO:1903977 | positive regulation of glial cell migration | 4.14E-4 | 2.56E-2 | 19.72 (15721,13,184,3) | Trem2 - triggering receptor expressed on myeloid cells 2  P2rx4 - purinergic receptor p2x, ligand-gated ion channel 4  Csf1 - colony stimulating factor 1 (macrophage) |
| GO:0006875 | cellular metal ion homeostasis | 4.21E-4 | 2.6E-2 | 2.62 (15721,521,184,16) | Ccl2 - chemokine (c-c motif) ligand 2  Ccr5 - chemokine (c-c motif) receptor 5  Cp - ceruloplasmin  Trf - transferrin  Inpp4b - inositol polyphosphate-4-phosphatase, type ii  Slc11a1 - solute carrier family 11 (proton-coupled divalent metal ion transporters), member 1  Fpr1 - formyl peptide receptor 1  Thy1 - thymus cell antigen 1, theta  Fpr2 - formyl peptide receptor 2  Prkcb - protein kinase c, beta  Cxcl2 - chemokine (c-x-c motif) ligand 2  Tgm2 - transglutaminase 2, c polypeptide  Ccl3 - chemokine (c-c motif) ligand 3  Ccrl2 - chemokine (c-c motif) receptor-like 2  P2rx4 - purinergic receptor p2x, ligand-gated ion channel 4 |

|  |  |  |  |  | Ccl5 - chemokine (c-c motif) ligand 5 |
| --- | --- | --- | --- | --- | --- |
| GO:0098657 | import into cell | 4.21E-4 | 2.59E-2 | 2.62 (15721,521,184,16) | Mrc1 - mannose receptor, c type 1  Mtmr9 - myotubularin related protein 9  Alox15 - arachidonate 15-lipoxygenase  Slc22a3 - solute carrier family 22 (organic cation transporter), member 3  Myo7a - myosin viia  Trem2 - triggering receptor expressed on myeloid cells 2  Slc11a1 - solute carrier family 11 (proton-coupled divalent metal ion transporters), member 1  Hck - hemopoietic cell kinase  Fpr2 - formyl peptide receptor 2  Slc15a3 - solute carrier family 15, member 3  Tgm2 - transglutaminase 2, c polypeptide  Cd9 - cd9 antigen  Snx10 - sorting nexin 10  Dab2 - disabled 2, mitogen-responsive phosphoprotein  Pld4 - phospholipase d family, member 4  Mfge8 - milk fat globule-egf factor 8 protein |
| GO:0022610 | biological adhesion | 4.43E-4 | 2.71E-2 | 2.30 (15721,742,184,20) | Itgax - integrin alpha x  Igfbp7 - insulin-like growth factor binding protein 7  Dsc2 - desmocollin 2  Sirpa - signal-regulatory protein alpha  Icam1 - intercellular adhesion molecule 1  Siglec5 - sialic acid binding ig-like lectin 5  Atrnl1 - attractin like 1  Thy1 - thymus cell antigen 1, theta  Cd34 - cd34 antigen  Cd9 - cd9 antigen  Acvr1 - activin a receptor, type 1  Lpp - lim domain containing preferred translocation partner in lipoma  Alcam - activated leukocyte cell adhesion molecule  Emb - embigin  Parvb - parvin, beta  Lgals3bp - lectin, galactoside-binding, soluble, 3 binding protein Lpxn - leupaxin  Gpnmb - glycoprotein (transmembrane) nmb  Ccl5 - chemokine (c-c motif) ligand 5  Mfge8 - milk fat globule-egf factor 8 protein |
| GO:0045087 | innate immune response | 4.44E-4 | 2.7E-2 | 2.84 (15721,421,184,14) | Ear2 - eosinophil-associated, ribonuclease a family, member 2 Lgals3 - lectin, galactose binding, soluble 3  Clec4n - c-type lectin domain family 4, member n  Wfdc17 - wap four-disulfide core domain 17  Trem2 - triggering receptor expressed on myeloid cells 2  Fgr - gardner-rasheed feline sarcoma viral (fgr) oncogene homolog Hck - hemopoietic cell kinase  Slc15a3 - solute carrier family 15, member 3  Csf1r - colony stimulating factor 1 receptor  Polr3k - polymerase (rna) iii (dna directed) polypeptide k  Irg1 - immunoresponsive gene 1  Pld3 - phospholipase d family, member 3  Pld4 - phospholipase d family, member 4  Csf1 - colony stimulating factor 1 (macrophage) |
| GO:0031641 | regulation of myelination | 4.5E-4 | 2.73E-2 | 7.77 (15721,55,184,5) | Itgax - integrin alpha x  Tnfrsf1b - tumor necrosis factor receptor superfamily, member 1b Trf - transferrin  Cst7 - cystatin f (leukocystatin)  Egr2 - early growth response 2 |
|  |  |  |  |  | Trf - transferrin  Mtmr9 - myotubularin related protein 9  Icam1 - intercellular adhesion molecule 1  Trem2 - triggering receptor expressed on myeloid cells 2  Fgr - gardner-rasheed feline sarcoma viral (fgr) oncogene homolog Akap11 - a kinase (prka) anchor protein 11  Fpr2 - formyl peptide receptor 2  Cd74 - cd74 antigen (invariant polypeptide of major histocompatibility complex, class ii antigen-associated)  Csf1r - colony stimulating factor 1 receptor  Csf2 - colony stimulating factor 2 (granulocyte-macrophage)  Socs3 - suppressor of cytokine signaling 3  Avpi1 - arginine vasopressin-induced 1  Fam20c - family with sequence similarity 20, member c  Ccl3 - chemokine (c-c motif) ligand 3  Dab2 - disabled 2, mitogen-responsive phosphoprotein  Tnfaip3 - tumor necrosis factor, alpha-induced protein 3  Nenf - neuron derived neurotrophic factor  Acvr1 - activin a receptor, type 1  Ccl2 - chemokine (c-c motif) ligand 2  Ccl5 - chemokine (c-c motif) ligand 5  Slc2a6 - solute carrier family 2 (facilitated glucose transporter), member 6  Ccl17 - chemokine (c-c motif) ligand 17  Zeb2 - zinc finger e-box binding homeobox 2  Inhba - inhibin beta-a  Il1rn - interleukin 1 receptor antagonist |

| GO:0051174 | regulation of phosphorus metabolic process | 4.62E-4 | 2.79E-2 | 1.81 (15721,1607,184,34) | Ccl4 - chemokine (c-c motif) ligand 4  Csf1 - colony stimulating factor 1 (macrophage)  Gpnmb - glycoprotein (transmembrane) nmb  Ndrg2 - n-myc downstream regulated gene 2  Sirpa - signal-regulatory protein alpha  Alox15 - arachidonate 15-lipoxygenase  Slc11a1 - solute carrier family 11 (proton-coupled divalent metal ion transporters), member 1  Thy1 - thymus cell antigen 1, theta  Mmp8 - matrix metallopeptidase 8 |
| --- | --- | --- | --- | --- | --- |
| GO:0030003 | cellular cation homeostasis | 4.65E-4 | 2.8E-2 | 2.51 (15721,579,184,17) | Ccl2 - chemokine (c-c motif) ligand 2  Ccr5 - chemokine (c-c motif) receptor 5  Cp - ceruloplasmin  Trf - transferrin  Inpp4b - inositol polyphosphate-4-phosphatase, type ii  Slc11a1 - solute carrier family 11 (proton-coupled divalent metal ion transporters), member 1  Fpr1 - formyl peptide receptor 1  Thy1 - thymus cell antigen 1, theta  Atp6v0d2 - atpase, h+ transporting, lysosomal v0 subunit d2  Fpr2 - formyl peptide receptor 2  Prkcb - protein kinase c, beta  Cxcl2 - chemokine (c-x-c motif) ligand 2  Tgm2 - transglutaminase 2, c polypeptide  Ccl3 - chemokine (c-c motif) ligand 3  Ccrl2 - chemokine (c-c motif) receptor-like 2  P2rx4 - purinergic receptor p2x, ligand-gated ion channel 4  Ccl5 - chemokine (c-c motif) ligand 5 |
| GO:0002697 | regulation of immune effector process | 5.06E-4 | 3.04E-2 | 2.95 (15721,377,184,13) | Ccl2 - chemokine (c-c motif) ligand 2  Lgals3 - lectin, galactose binding, soluble 3  H2-D1 - histocompatibility 2, d region locus 1  Il7r - interleukin 7 receptor  Tnfrsf1b - tumor necrosis factor receptor superfamily, member 1b Fgr - gardner-rasheed feline sarcoma viral (fgr) oncogene homolog Fgl2 - fibrinogen-like protein 2  H2-K1 - histocompatibility 2, k1, k region  Cd74 - cd74 antigen (invariant polypeptide of major histocompatibility complex, class ii antigen-associated)  Mmp12 - matrix metallopeptidase 12  H2-M3 - histocompatibility 2, m region locus 3  Tnfaip3 - tumor necrosis factor, alpha-induced protein 3  Ccl5 - chemokine (c-c motif) ligand 5 |
| GO:0014015 | positive regulation of gliogenesis | 5.13E-4 | 3.06E-2 | 5.96 (15721,86,184,6) | Tnfrsf1b - tumor necrosis factor receptor superfamily, member 1b Trf - transferrin  Trem2 - triggering receptor expressed on myeloid cells 2  P2rx4 - purinergic receptor p2x, ligand-gated ion channel 4  Csf1 - colony stimulating factor 1 (macrophage)  Egr2 - early growth response 2 |
| GO:0002476 | antigen processing and presentation of endogenous peptide antigen via MHC class Ib | 5.22E-4 | 3.11E-2 | 18.31 (15721,14,184,3) | H2-D1 - histocompatibility 2, d region locus 1  H2-M3 - histocompatibility 2, m region locus 3  H2-K1 - histocompatibility 2, k1, k region |
| GO:0002484 | antigen processing and presentation of endogenous peptide antigen via MHC class I via ER pathway | 5.22E-4 | 3.09E-2 | 18.31 (15721,14,184,3) | H2-D1 - histocompatibility 2, d region locus 1  H2-M3 - histocompatibility 2, m region locus 3  H2-K1 - histocompatibility 2, k1, k region |
| GO:0033089 | positive regulation of T cell differentiation in thymus | 5.22E-4 | 3.08E-2 | 18.31 (15721,14,184,3) | Il7r - interleukin 7 receptor  Bmi1 - bmi1 polycomb ring finger oncogene  Egr3 - early growth response 3 |
|  |  |  |  |  | Cd34 - cd34 antigen |

| GO:0032720 | negative regulation of tumor necrosis factor production | 5.32E-4 | 3.13E-2 | 7.49 (15721,57,184,5) | Sirpa - signal-regulatory protein alpha  Trem2 - triggering receptor expressed on myeloid cells 2  Tnfaip3 - tumor necrosis factor, alpha-induced protein 3  Gpnmb - glycoprotein (transmembrane) nmb |
| --- | --- | --- | --- | --- | --- |
| GO:0032642 | regulation of chemokine production | 5.46E-4 | 3.2E-2 | 5.89 (15721,87,184,6) | Cd74 - cd74 antigen (invariant polypeptide of major histocompatibility complex, class ii antigen-associated)  Csf1r - colony stimulating factor 1 receptor  Sirpa - signal-regulatory protein alpha  Trem2 - triggering receptor expressed on myeloid cells 2  Ccl5 - chemokine (c-c motif) ligand 5  Il1rl1 - interleukin 1 receptor-like 1 |
| GO:0045637 | regulation of myeloid cell differentiation | 5.46E-4 | 3.19E-2 | 3.88 (15721,198,184,9) | Cd74 - cd74 antigen (invariant polypeptide of major histocompatibility complex, class ii antigen-associated)  Csf1r - colony stimulating factor 1 receptor  Inhba - inhibin beta-a  Inpp4b - inositol polyphosphate-4-phosphatase, type ii  Ccl3 - chemokine (c-c motif) ligand 3  Trem2 - triggering receptor expressed on myeloid cells 2  Runx1 - runt related transcription factor 1  Csf1 - colony stimulating factor 1 (macrophage)  Ccl5 - chemokine (c-c motif) ligand 5 |
| GO:0032102 | negative regulation of response to external stimulus | 5.64E-4 | 3.28E-2 | 3.08 (15721,333,184,12) | Cd34 - cd34 antigen  Cd9 - cd9 antigen  Padi2 - peptidyl arginine deiminase, type ii  Socs3 - suppressor of cytokine signaling 3  Tnfrsf1b - tumor necrosis factor receptor superfamily, member 1b Sirpa - signal-regulatory protein alpha  Irg1 - immunoresponsive gene 1  Cst7 - cystatin f (leukocystatin)  Fgl2 - fibrinogen-like protein 2  Nenf - neuron derived neurotrophic factor  Tnfaip3 - tumor necrosis factor, alpha-induced protein 3  Fpr2 - formyl peptide receptor 2 |
| GO:0055065 | metal ion homeostasis | 5.65E-4 | 3.27E-2 | 2.47 (15721,589,184,17) | Ccl2 - chemokine (c-c motif) ligand 2  Ccr5 - chemokine (c-c motif) receptor 5  Cp - ceruloplasmin  Trf - transferrin  Inpp4b - inositol polyphosphate-4-phosphatase, type ii  Slc11a1 - solute carrier family 11 (proton-coupled divalent metal ion transporters), member 1  Fpr1 - formyl peptide receptor 1  Thy1 - thymus cell antigen 1, theta  Fpr2 - formyl peptide receptor 2  Prkcb - protein kinase c, beta  Cxcl2 - chemokine (c-x-c motif) ligand 2  Tgm2 - transglutaminase 2, c polypeptide  Snx10 - sorting nexin 10  Ccl3 - chemokine (c-c motif) ligand 3  Ccrl2 - chemokine (c-c motif) receptor-like 2  P2rx4 - purinergic receptor p2x, ligand-gated ion channel 4  Ccl5 - chemokine (c-c motif) ligand 5 |
| GO:0043066 | negative regulation of apoptotic process | 5.86E-4 | 3.38E-2 | 2.15 (15721,875,184,22) | Lgals3 - lectin, galactose binding, soluble 3  Ccr5 - chemokine (c-c motif) receptor 5  Chst11 - carbohydrate sulfotransferase 11  Il7r - interleukin 7 receptor  Icam1 - intercellular adhesion molecule 1  Rnf157 - ring finger protein 157  Acot1 - acyl-coa thioesterase 1  Trem2 - triggering receptor expressed on myeloid cells 2  Fpr2 - formyl peptide receptor 2  Hck - hemopoietic cell kinase  Cd74 - cd74 antigen (invariant polypeptide of major histocompatibility complex, class ii antigen-associated)  Csf1r - colony stimulating factor 1 receptor  Csf2 - colony stimulating factor 2 (granulocyte-macrophage)  Il1rn - interleukin 1 receptor antagonist  Socs3 - suppressor of cytokine signaling 3  Serpinb2 - serine (or cysteine) peptidase inhibitor, clade b, member 2  Acvr1 - activin a receptor, type 1  Dab2 - disabled 2, mitogen-responsive phosphoprotein  Bmi1 - bmi1 polycomb ring finger oncogene  Tnfaip3 - tumor necrosis factor, alpha-induced protein 3  Egr3 - early growth response 3 |

|  |  |  |  |  | Ccl5 - chemokine (c-c motif) ligand 5 |
| --- | --- | --- | --- | --- | --- |
| GO:0055074 | calcium ion homeostasis | 6E-4 | 3.45E-2 | 2.76 (15721,434,184,14) | Ccl2 - chemokine (c-c motif) ligand 2  Ccr5 - chemokine (c-c motif) receptor 5  Inpp4b - inositol polyphosphate-4-phosphatase, type ii  Fpr1 - formyl peptide receptor 1  Thy1 - thymus cell antigen 1, theta  Fpr2 - formyl peptide receptor 2  Prkcb - protein kinase c, beta  Cxcl2 - chemokine (c-x-c motif) ligand 2  Tgm2 - transglutaminase 2, c polypeptide  Snx10 - sorting nexin 10  Ccl3 - chemokine (c-c motif) ligand 3  Ccrl2 - chemokine (c-c motif) receptor-like 2  P2rx4 - purinergic receptor p2x, ligand-gated ion channel 4  Ccl5 - chemokine (c-c motif) ligand 5 |
| GO:0006873 | cellular ion homeostasis | 6.21E-4 | 3.56E-2 | 2.45 (15721,594,184,17) | Ccl2 - chemokine (c-c motif) ligand 2  Ccr5 - chemokine (c-c motif) receptor 5  Cp - ceruloplasmin  Trf - transferrin  Inpp4b - inositol polyphosphate-4-phosphatase, type ii  Slc11a1 - solute carrier family 11 (proton-coupled divalent metal ion transporters), member 1  Fpr1 - formyl peptide receptor 1  Thy1 - thymus cell antigen 1, theta  Atp6v0d2 - atpase, h+ transporting, lysosomal v0 subunit d2  Fpr2 - formyl peptide receptor 2  Prkcb - protein kinase c, beta  Cxcl2 - chemokine (c-x-c motif) ligand 2  Tgm2 - transglutaminase 2, c polypeptide  Ccl3 - chemokine (c-c motif) ligand 3  Ccrl2 - chemokine (c-c motif) receptor-like 2  P2rx4 - purinergic receptor p2x, ligand-gated ion channel 4  Ccl5 - chemokine (c-c motif) ligand 5 |
| GO:0002507 | tolerance induction | 6.47E-4 | 3.69E-2 | 17.09 (15721,15,184,3) | H2-M3 - histocompatibility 2, m region locus 3  Irg1 - immunoresponsive gene 1  Tnfaip3 - tumor necrosis factor, alpha-induced protein 3 |
| GO:0002428 | antigen processing and presentation of peptide antigen via MHC class I | 6.47E-4 | 3.68E-2 | 17.09 (15721,15,184,3) | H2-D1 - histocompatibility 2, d region locus 1  H2-M3 - histocompatibility 2, m region locus 3  H2-K1 - histocompatibility 2, k1, k region |
| GO:1903978 | regulation of microglial cell activation | 6.47E-4 | 3.67E-2 | 17.09 (15721,15,184,3) | Trem2 - triggering receptor expressed on myeloid cells 2  Cst7 - cystatin f (leukocystatin)  Mmp8 - matrix metallopeptidase 8 |
| GO:0048246 | macrophage chemotaxis | 6.47E-4 | 3.65E-2 | 17.09 (15721,15,184,3) | Ccl2 - chemokine (c-c motif) ligand 2  Lgals3 - lectin, galactose binding, soluble 3  Ccl3 - chemokine (c-c motif) ligand 3 |
| GO:0055080 | cation homeostasis | 6.9E-4 | 3.88E-2 | 2.35 (15721,655,184,18) | Ccl2 - chemokine (c-c motif) ligand 2  Ccr5 - chemokine (c-c motif) receptor 5  Cp - ceruloplasmin  Trf - transferrin  Inpp4b - inositol polyphosphate-4-phosphatase, type ii  Slc11a1 - solute carrier family 11 (proton-coupled divalent metal ion transporters), member 1  Fpr1 - formyl peptide receptor 1  Thy1 - thymus cell antigen 1, theta  Atp6v0d2 - atpase, h+ transporting, lysosomal v0 subunit d2  Fpr2 - formyl peptide receptor 2  Prkcb - protein kinase c, beta  Cxcl2 - chemokine (c-x-c motif) ligand 2  Tgm2 - transglutaminase 2, c polypeptide  Snx10 - sorting nexin 10  Ccl3 - chemokine (c-c motif) ligand 3  Ccrl2 - chemokine (c-c motif) receptor-like 2  P2rx4 - purinergic receptor p2x, ligand-gated ion channel 4  Ccl5 - chemokine (c-c motif) ligand 5 |
|  |  |  |  |  | Ccl2 - chemokine (c-c motif) ligand 2  Ccr5 - chemokine (c-c motif) receptor 5  Inpp4b - inositol polyphosphate-4-phosphatase, type ii |

| GO:0072503 | cellular divalent inorganic cation homeostasis | 7.01E-4 | 3.93E-2 | 2.71 (15721,441,184,14) | Slc11a1 - solute carrier family 11 (proton-coupled divalent metal ion transporters), member 1  Fpr1 - formyl peptide receptor 1  Thy1 - thymus cell antigen 1, theta  Fpr2 - formyl peptide receptor 2  Prkcb - protein kinase c, beta  Cxcl2 - chemokine (c-x-c motif) ligand 2  Tgm2 - transglutaminase 2, c polypeptide  Ccl3 - chemokine (c-c motif) ligand 3  Ccrl2 - chemokine (c-c motif) receptor-like 2  P2rx4 - purinergic receptor p2x, ligand-gated ion channel 4  Ccl5 - chemokine (c-c motif) ligand 5 |
| --- | --- | --- | --- | --- | --- |
| GO:0002931 | response to ischemia | 7.16E-4 | 4E-2 | 9.76 (15721,35,184,4) | Csf1r - colony stimulating factor 1 receptor  Trem2 - triggering receptor expressed on myeloid cells 2  P2rx4 - purinergic receptor p2x, ligand-gated ion channel 4  Csf1 - colony stimulating factor 1 (macrophage) |
| GO:0001676 | long-chain fatty acid metabolic process | 7.78E-4 | 4.32E-2 | 5.51 (15721,93,184,6) | Cyp4f18 - cytochrome p450, family 4, subfamily f, polypeptide 18  Mgll - monoglyceride lipase  Acot2 - acyl-coa thioesterase 2  Acsl4 - acyl-coa synthetase long-chain family member 4  Alox15 - arachidonate 15-lipoxygenase  Acot1 - acyl-coa thioesterase 1 |
| GO:0014009 | glial cell proliferation | 7.9E-4 | 4.38E-2 | 16.02 (15721,16,184,3) | Csf1r - colony stimulating factor 1 receptor  Trem2 - triggering receptor expressed on myeloid cells 2  Csf1 - colony stimulating factor 1 (macrophage) |
| GO:0043069 | negative regulation of programmed cell death | 8.03E-4 | 4.43E-2 | 2.10 (15721,896,184,22) | Lgals3 - lectin, galactose binding, soluble 3  Ccr5 - chemokine (c-c motif) receptor 5  Chst11 - carbohydrate sulfotransferase 11  Il7r - interleukin 7 receptor  Icam1 - intercellular adhesion molecule 1  Rnf157 - ring finger protein 157  Acot1 - acyl-coa thioesterase 1  Trem2 - triggering receptor expressed on myeloid cells 2  Fpr2 - formyl peptide receptor 2  Hck - hemopoietic cell kinase  Cd74 - cd74 antigen (invariant polypeptide of major histocompatibility complex, class ii antigen-associated)  Csf1r - colony stimulating factor 1 receptor  Csf2 - colony stimulating factor 2 (granulocyte-macrophage)  Il1rn - interleukin 1 receptor antagonist  Socs3 - suppressor of cytokine signaling 3  Serpinb2 - serine (or cysteine) peptidase inhibitor, clade b, member 2  Acvr1 - activin a receptor, type 1  Dab2 - disabled 2, mitogen-responsive phosphoprotein  Bmi1 - bmi1 polycomb ring finger oncogene  Tnfaip3 - tumor necrosis factor, alpha-induced protein 3  Egr3 - early growth response 3  Ccl5 - chemokine (c-c motif) ligand 5 |
| GO:2000503 | positive regulation of natural killer cell chemotaxis | 8.05E-4 | 4.43E-2 | 42.72 (15721,4,184,2) | Ccl4 - chemokine (c-c motif) ligand 4  Ccl5 - chemokine (c-c motif) ligand 5 |
| GO:0002291 | T cell activation via T cell receptor contact with antigen bound to MHC molecule on antigen presenting cell | 8.05E-4 | 4.41E-2 | 42.72 (15721,4,184,2) | Icam1 - intercellular adhesion molecule 1  Fgl2 - fibrinogen-like protein 2 |
| GO:0002586 | regulation of antigen processing and presentation of peptide antigen via MHC class II | 8.05E-4 | 4.4E-2 | 42.72 (15721,4,184,2) | H2-Oa - histocompatibility 2, o region alpha locus  Trem2 - triggering receptor expressed on myeloid cells 2 |
| GO:0043122 | regulation of I-kappaB kinase/NF-kappaB signaling | 8.51E-4 | 4.63E-2 | 4.04 (15721,169,184,8) | Cd74 - cd74 antigen (invariant polypeptide of major histocompatibility complex, class ii antigen-associated)  Prkcb - protein kinase c, beta  Tgm2 - transglutaminase 2, c polypeptide  Clec4n - c-type lectin domain family 4, member n  Casp1 - caspase 1  Sirpa - signal-regulatory protein alpha  Tnfaip3 - tumor necrosis factor, alpha-induced protein 3  Il1rl1 - interleukin 1 receptor-like 1 |
|  |  |  |  |  | Ccl2 - chemokine (c-c motif) ligand 2  Mmp2 - matrix metallopeptidase 2 |

| GO:0009611 | response to wounding | 8.51E-4 | 4.62E-2 | 4.04 (15721,169,184,8) | Serpinb2 - serine (or cysteine) peptidase inhibitor, clade b, member 2  Alox15 - arachidonate 15-lipoxygenase  Mmp12 - matrix metallopeptidase 12  P2rx4 - purinergic receptor p2x, ligand-gated ion channel 4  Slc11a1 - solute carrier family 11 (proton-coupled divalent metal ion transporters), member 1  Tnfaip3 - tumor necrosis factor, alpha-induced protein 3 |
| --- | --- | --- | --- | --- | --- |
| GO:0098771 | inorganic ion homeostasis | 8.64E-4 | 4.67E-2 | 2.30 (15721,668,184,18) | Ccl2 - chemokine (c-c motif) ligand 2  Ccr5 - chemokine (c-c motif) receptor 5  Cp - ceruloplasmin  Trf - transferrin  Inpp4b - inositol polyphosphate-4-phosphatase, type ii  Slc11a1 - solute carrier family 11 (proton-coupled divalent metal ion transporters), member 1  Fpr1 - formyl peptide receptor 1  Thy1 - thymus cell antigen 1, theta  Atp6v0d2 - atpase, h+ transporting, lysosomal v0 subunit d2  Fpr2 - formyl peptide receptor 2  Prkcb - protein kinase c, beta  Cxcl2 - chemokine (c-x-c motif) ligand 2  Tgm2 - transglutaminase 2, c polypeptide  Snx10 - sorting nexin 10  Ccl3 - chemokine (c-c motif) ligand 3  Ccrl2 - chemokine (c-c motif) receptor-like 2  P2rx4 - purinergic receptor p2x, ligand-gated ion channel 4  Ccl5 - chemokine (c-c motif) ligand 5 |
| GO:0019730 | antimicrobial humoral response | 8.7E-4 | 4.68E-2 | 5.40 (15721,95,184,6) | Cxcl2 - chemokine (c-x-c motif) ligand 2  Ccl2 - chemokine (c-c motif) ligand 2  Lgals3 - lectin, galactose binding, soluble 3  Ccl17 - chemokine (c-c motif) ligand 17  Trf - transferrin  Wfdc17 - wap four-disulfide core domain 17 |
| GO:0001914 | regulation of T cell mediated cytotoxicity | 8.87E-4 | 4.76E-2 | 9.24 (15721,37,184,4) | H2-D1 - histocompatibility 2, d region locus 1  Il7r - interleukin 7 receptor  H2-M3 - histocompatibility 2, m region locus 3  H2-K1 - histocompatibility 2, k1, k region |
| GO:0035458 | cellular response to interferon-beta | 8.87E-4 | 4.74E-2 | 9.24 (15721,37,184,4) | Ifi205 - interferon activated gene 205  Ifi203 - interferon activated gene 203  Ifi202b - interferon activated gene 202b  Irg1 - immunoresponsive gene 1 |
| GO:0048839 | inner ear development | 9.07E-4 | 4.84E-2 | 6.68 (15721,64,184,5) | Igfbp7 - insulin-like growth factor binding protein 7  Cebpa - ccaat/enhancer binding protein (c/ebp), alpha  Myo7a - myosin viia  H2-DMa - histocompatibility 2, class ii, locus dma  H2-K1 - histocompatibility 2, k1, k region |
| GO:0006959 | humoral immune response | 9.19E-4 | 4.88E-2 | 4.00 (15721,171,184,8) | Cxcl2 - chemokine (c-x-c motif) ligand 2  Ccl2 - chemokine (c-c motif) ligand 2  Lgals3 - lectin, galactose binding, soluble 3  Ccl17 - chemokine (c-c motif) ligand 17  H2-Ab1 - histocompatibility 2, class ii antigen a, beta 1  Trf - transferrin  Wfdc17 - wap four-disulfide core domain 17  Bmi1 - bmi1 polycomb ring finger oncogene |
| GO:0044419 | interspecies interaction between organisms | 9.37E-4 | 4.96E-2 | 3.31 (15721,258,184,10) | Cxcl2 - chemokine (c-x-c motif) ligand 2  Ccl2 - chemokine (c-c motif) ligand 2  Csf1r - colony stimulating factor 1 receptor  Ccr5 - chemokine (c-c motif) receptor 5  Lgals3 - lectin, galactose binding, soluble 3  Ccl17 - chemokine (c-c motif) ligand 17  Mcts1 - malignant t cell amplified sequence 1  Ctsb - cathepsin b  Ccl4 - chemokine (c-c motif) ligand 4  Ccl5 - chemokine (c-c motif) ligand 5 |
|  |  |  |  |  | Mtmr9 - myotubularin related protein 9  Icam1 - intercellular adhesion molecule 1  Trf - transferrin  Trem2 - triggering receptor expressed on myeloid cells 2 |

| GO:0019220 | regulation of phosphate metabolic process | 9.42E-4 | 4.97E-2 | 1.76 (15721,1606,184,33) | Fgr - gardner-rasheed feline sarcoma viral (fgr) oncogene homolog  Akap11 - a kinase (prka) anchor protein 11  Fpr2 - formyl peptide receptor 2  Cd74 - cd74 antigen (invariant polypeptide of major histocompatibility complex, class ii antigen-associated)  Csf1r - colony stimulating factor 1 receptor  Csf2 - colony stimulating factor 2 (granulocyte-macrophage)  Socs3 - suppressor of cytokine signaling 3  Avpi1 - arginine vasopressin-induced 1  Ccl3 - chemokine (c-c motif) ligand 3  Dab2 - disabled 2, mitogen-responsive phosphoprotein  Ccl4 - chemokine (c-c motif) ligand 4  Csf1 - colony stimulating factor 1 (macrophage)  Ccl5 - chemokine (c-c motif) ligand 5  Slc2a6 - solute carrier family 2 (facilitated glucose transporter), member 6 Gpnmb - glycoprotein (transmembrane) nmb  Ccl2 - chemokine (c-c motif) ligand 2  Ccl17 - chemokine (c-c motif) ligand 17  Ndrg2 - n-myc downstream regulated gene 2  Sirpa - signal-regulatory protein alpha  Alox15 - arachidonate 15-lipoxygenase  Slc11a1 - solute carrier family 11 (proton-coupled divalent metal ion transporters), member 1  Thy1 - thymus cell antigen 1, theta  Mmp8 - matrix metallopeptidase 8  Zeb2 - zinc finger e-box binding homeobox 2  Inhba - inhibin beta-a  Il1rn - interleukin 1 receptor antagonist  Acvr1 - activin a receptor, type 1  Nenf - neuron derived neurotrophic factor  Tnfaip3 - tumor necrosis factor, alpha-induced protein 3 |
| --- | --- | --- | --- | --- | --- |
| GO:0022604 | regulation of cell morphogenesis | 9.51E-4 | 5E-2 | 2.52 (15721,508,184,15) | Ccl2 - chemokine (c-c motif) ligand 2  Ccr5 - chemokine (c-c motif) receptor 5  Mgll - monoglyceride lipase  Icam1 - intercellular adhesion molecule 1  Fmnl2 - formin-like 2  Rnf157 - ring finger protein 157  Fgr - gardner-rasheed feline sarcoma viral (fgr) oncogene homolog  Thy1 - thymus cell antigen 1, theta  Zeb2 - zinc finger e-box binding homeobox 2  Hck - hemopoietic cell kinase  Csf1r - colony stimulating factor 1 receptor  Plxnc1 - plexin c1  Dab2 - disabled 2, mitogen-responsive phosphoprotein  Ccl3 - chemokine (c-c motif) ligand 3  Parvb - parvin, beta |
| GO:2001237 | negative regulation of extrinsic apoptotic signaling pathway | 9.7E-4 | 5.08E-2 | 5.28 (15721,97,184,6) | Lgals3 - lectin, galactose binding, soluble 3  Csf2 - colony stimulating factor 2 (granulocyte-macrophage)  Icam1 - intercellular adhesion molecule 1  Acvr1 - activin a receptor, type 1  Dab2 - disabled 2, mitogen-responsive phosphoprotein  Tnfaip3 - tumor necrosis factor, alpha-induced protein 3 |
| GO:0031644 | regulation of neurological system process | 9.9E-4 | 5.17E-2 | 3.95 (15721,173,184,8) | Itgax - integrin alpha x  Mgll - monoglyceride lipase  Tnfrsf1b - tumor necrosis factor receptor superfamily, member 1b  Trf - transferrin  Fabp5 - fatty acid binding protein 5, epidermal  Cst7 - cystatin f (leukocystatin)  Egr2 - early growth response 2  Ctss - cathepsin s |

Differentially expressed RNA was ranked according to the p-values of differential expression and degree of enrichment compared with the total number of expressed genes analysed (17680 GO terms). The GOrilla database updated on Mar 6, 2021 was used.

**'P-value'** is the enrichment p-value computed according to the mHG or HG model. This p-value is not corrected for multiple testing of 1953 GO terms.

**'FDR q-value'** is the correction of the above p-value for multiple testing using the Benjamini and Hochberg (1995) method. Namely, for the ith term (ranked according to p-value) the FDR q-value is (p-value * number of GO terms) / i.

**Enrichment (N, B, n, b)** is defined as follows:

N - is the total number of genes

B - is the total number of genes associated with a specific GO term

n - is the number of genes in the top of the user's input list or in the target set when appropriate b - is the number of genes in the intersection

Enrichment = (b/n) / (B/N)

**Genes:** For each GO term you can see the list of associated genes that appear in the optimal top of the list. Each gene name is specified by gene symbol followed by a short description of the gene.
