# Supplementary material for: Proteomic and transcriptomic characterisation of FIA10, a novel murine leukemic cell line that metastasizes into the brain
Source: PLoS One. 2024 Jan 12;19(1):e0295641. doi: 10.1371/journal.pone.0295641 (PMC10786371; doi:10.1371/journal.pone.0295641)
Supplement: S7 Table — (DOCX) [file pone.0295641.s012.docx]

**Gene Ontology: Molecular function FIA10 vs FIA18 RNA upregulated**

| **GO term** | **Description** | **P-value** | **FDR q-value** | **Enrichment (N, B, n, b)** | **Genes** |
| --- | --- | --- | --- | --- | --- |
| GO:0005102 | signaling receptor binding | 1.11E-7 | 5.06E-4 | 2.46 (17680,1525,184,39) | Trf - transferrin  Icam1 - intercellular adhesion molecule 1  Fgr - gardner-rasheed feline sarcoma viral (fgr) oncogene homolog  Fgl2 - fibrinogen-like protein 2  Fpr1 - formyl peptide receptor 1  Fpr2 - formyl peptide receptor 2  Hck - hemopoietic cell kinase  Cd74 - cd74 antigen (invariant polypeptide of major histocompatibility complex, class ii antigen-associated)  Prkcb - protein kinase c, beta  Cxcl2 - chemokine (c-x-c motif) ligand 2  Cd9 - cd9 antigen  Csf2 - colony stimulating factor 2 (granulocyte-macrophage)  Mmp13 - matrix metallopeptidase 13  Dab2 - disabled 2, mitogen-responsive phosphoprotein  Ccl3 - chemokine (c-c motif) ligand 3  H2-M3 - histocompatibility 2, m region locus 3  Ccl4 - chemokine (c-c motif) ligand 4  Saa3 - serum amyloid a 3  P2rx4 - purinergic receptor p2x, ligand-gated ion channel 4  Gpnmb - glycoprotein (transmembrane) nmb  Csf1 - colony stimulating factor 1 (macrophage)  Ccl5 - chemokine (c-c motif) ligand 5  Neto2 - neuropilin (nrp) and tolloid (tll)-like 2  Ccl2 - chemokine (c-c motif) ligand 2  Itgax - integrin alpha x  Lgals3 - lectin, galactose binding, soluble 3  H2-D1 - histocompatibility 2, d region locus 1  Ccl17 - chemokine (c-c motif) ligand 17  Thy1 - thymus cell antigen 1, theta  H2-K1 - histocompatibility 2, k1, k region Inhba - inhibin beta-a  Padi2 - peptidyl arginine deiminase, type ii  Kdm5d - lysine (k)-specific demethylase 5d  Il1rn - interleukin 1 receptor antagonist  Ngfrap1 - nerve growth factor receptor (tnfrsf16) associated protein 1  Ccrl2 - chemokine (c-c motif) receptor-like 2  Nenf - neuron derived neurotrophic factor  Pilra - paired immunoglobin-like type 2 receptor alpha  Mfge8 - milk fat globule-egf factor 8 protein |
| GO:0019955 | cytokine binding | 1.23E-7 | 2.83E-4 | 8.07 (17680,131,184,11) | Cd74 - cd74 antigen (invariant polypeptide of major histocompatibility complex, class ii antigen-associated)  Csf1r - colony stimulating factor 1 receptor  Ccr5 - chemokine (c-c motif) receptor 5  Csf2ra - colony stimulating factor 2 receptor, alpha, low-affinity  (granulocyte-macrophage)  Il1rn - interleukin 1 receptor antagonist  Tnfrsf1b - tumor necrosis factor receptor superfamily, member 1b  Acvr1 - activin a receptor, type 1  Tnfrsf9 - tumor necrosis factor receptor superfamily, member 9  Il3ra - interleukin 3 receptor, alpha chain  Ccrl2 - chemokine (c-c motif) receptor-like 2  Il1rl1 - interleukin 1 receptor-like 1 |
| GO:0042605 | peptide antigen binding | 1.19E-6 | 1.82E-3 | 25.29 (17680,19,184,5) | H2-D1 - histocompatibility 2, d region locus 1  H2-Ab1 - histocompatibility 2, class ii antigen a, beta 1  H2-Aa - histocompatibility 2, class ii antigen a, alpha  H2-M3 - histocompatibility 2, m region locus 3  H2-K1 - histocompatibility 2, k1, k region |
| GO:0008009 | chemokine activity | 2.87E-6 | 3.29E-3 | 14.78 (17680,39,184,6) | Ccl2 - chemokine (c-c motif) ligand 2  Cxcl2 - chemokine (c-x-c motif) ligand 2  Ccl17 - chemokine (c-c motif) ligand 17  Ccl3 - chemokine (c-c motif) ligand 3  Ccl4 - chemokine (c-c motif) ligand 4  Ccl5 - chemokine (c-c motif) ligand 5 |
| GO:0042379 | chemokine receptor binding | 3.58E-6 | 3.28E-3 | 10.85 (17680,62,184,7) | Cxcl2 - chemokine (c-x-c motif) ligand 2  Ccl2 - chemokine (c-c motif) ligand 2  Ccl17 - chemokine (c-c motif) ligand 17  Ccl3 - chemokine (c-c motif) ligand 3  Ccl4 - chemokine (c-c motif) ligand 4  Ccrl2 - chemokine (c-c motif) receptor-like 2  Ccl5 - chemokine (c-c motif) ligand 5 |
| GO:0048020 | CCR chemokine receptor binding | 3.89E-6 | 2.97E-3 | 14.06 (17680,41,184,6) | Ccl2 - chemokine (c-c motif) ligand 2  Ccl17 - chemokine (c-c motif) ligand 17  Ccl3 - chemokine (c-c motif) ligand 3  Ccl4 - chemokine (c-c motif) ligand 4  Ccrl2 - chemokine (c-c motif) receptor-like 2  Ccl5 - chemokine (c-c motif) ligand 5 |
| GO:0003823 | antigen binding | 1.6E-5 | 1.05E-2 | 11.09 (17680,52,184,6) | H2-D1 - histocompatibility 2, d region locus 1  H2-Ab1 - histocompatibility 2, class ii antigen a, beta 1  H2-Aa - histocompatibility 2, class ii antigen a, alpha  Sirpa - signal-regulatory protein alpha  H2-M3 - histocompatibility 2, m region locus 3  H2-K1 - histocompatibility 2, k1, k region |
| GO:0038023 | signaling receptor activity | 2.34E-5 | 1.34E-2 | 2.55 (17680,904,184,24) | Cd200r3 - cd200 receptor 3  Ccr5 - chemokine (c-c motif) receptor 5  Lgals3 - lectin, galactose binding, soluble 3  Mrc1 - mannose receptor, c type 1  Il7r - interleukin 7 receptor  Tnfrsf1b - tumor necrosis factor receptor superfamily, member 1b  AF251705 - cdna sequence af251705  Tnfrsf9 - tumor necrosis factor receptor superfamily, member 9  Trem2 - triggering receptor expressed on myeloid cells 2  Fpr1 - formyl peptide receptor 1  Emr4 - egf-like module containing, mucin-like, hormone receptor-like sequence 4  Il1rl1 - interleukin 1 receptor-like 1  Fpr2 - formyl peptide receptor 2  Cd74 - cd74 antigen (invariant polypeptide of major histocompatibility complex, class ii antigen-associated)  Csf1r - colony stimulating factor 1 receptor  Csf2ra - colony stimulating factor 2 receptor, alpha, low-affinity (granulocyte-macrophage)  Plxnc1 - plexin c1  P2ry14 - purinergic receptor p2y, g-protein coupled, 14  Clec7a - c-type lectin domain family 7, member a  Acvr1 - activin a receptor, type 1  Ccrl2 - chemokine (c-c motif) receptor-like 2  Il3ra - interleukin 3 receptor, alpha chain  P2rx4 - purinergic receptor p2x, ligand-gated ion channel 4  Procr - protein c receptor, endothelial |
| GO:0005125 | cytokine activity | 3.37E-5 | 1.71E-2 | 5.00 (17680,192,184,10) | Cxcl2 - chemokine (c-x-c motif) ligand 2  Ccl2 - chemokine (c-c motif) ligand 2  Ccl17 - chemokine (c-c motif) ligand 17  Inhba - inhibin beta-a  Il1rn - interleukin 1 receptor antagonist  Csf2 - colony stimulating factor 2 (granulocyte-macrophage)  Ccl3 - chemokine (c-c motif) ligand 3  Ccl4 - chemokine (c-c motif) ligand 4  Ccl5 - chemokine (c-c motif) ligand 5  Csf1 - colony stimulating factor 1 (macrophage) |
| GO:0023026 | MHC class II protein complex binding | 3.76E-5 | 1.72E-2 | 41.18 (17680,7,184,3) | Cd74 - cd74 antigen (invariant polypeptide of major histocompatibility complex, class ii antigen-associated)  H2-Oa - histocompatibility 2, o region alpha locus  H2-DMa - histocompatibility 2, class ii, locus dma |
| GO:0042277 | peptide binding | 4.1E-5 | 1.71E-2 | 4.09 (17680,282,184,12) | Cd74 - cd74 antigen (invariant polypeptide of major histocompatibility complex, class ii antigen-associated)  Crip1 - cysteine-rich protein 1 (intestinal)  H2-D1 - histocompatibility 2, d region locus 1  Inhba - inhibin beta-a  Ipo4 - importin 4  H2-Ab1 - histocompatibility 2, class ii antigen a, beta 1  H2-Aa - histocompatibility 2, class ii antigen a, alpha  Ctsb - cathepsin b  H2-M3 - histocompatibility 2, m region locus 3  Trem2 - triggering receptor expressed on myeloid cells 2  H2-K1 - histocompatibility 2, k1, k region  Fpr2 - formyl peptide receptor 2 |
| GO:0004896 | cytokine receptor activity | 4.57E-5 | 1.74E-2 | 7.39 (17680,91,184,7) | Cd74 - cd74 antigen (invariant polypeptide of major histocompatibility complex, class ii antigen-associated)  Ccr5 - chemokine (c-c motif) receptor 5  Il7r - interleukin 7 receptor  Csf2ra - colony stimulating factor 2 receptor, alpha, low-affinity (granulocyte-macrophage)  Il3ra - interleukin 3 receptor, alpha chain  Ccrl2 - chemokine (c-c motif) receptor-like 2  Il1rl1 - interleukin 1 receptor-like 1 |
| GO:0060089 | molecular transducer activity | 4.67E-5 | 1.65E-2 | 2.44 (17680,944,184,24) | Cd200r3 - cd200 receptor 3  Ccr5 - chemokine (c-c motif) receptor 5  Lgals3 - lectin, galactose binding, soluble 3  Mrc1 - mannose receptor, c type 1  Il7r - interleukin 7 receptor  Tnfrsf1b - tumor necrosis factor receptor superfamily, member 1b  AF251705 - cdna sequence af251705  Tnfrsf9 - tumor necrosis factor receptor superfamily, member 9  Trem2 - triggering receptor expressed on myeloid cells 2  Fpr1 - formyl peptide receptor 1  Emr4 - egf-like module containing, mucin-like, hormone receptor-like sequence 4  Il1rl1 - interleukin 1 receptor-like 1  Fpr2 - formyl peptide receptor 2  Cd74 - cd74 antigen (invariant polypeptide of major histocompatibility complex, class ii antigen-associated)  Csf1r - colony stimulating factor 1 receptor  Csf2ra - colony stimulating factor 2 receptor, alpha, low-affinity (granulocyte-macrophage)  Plxnc1 - plexin c1  P2ry14 - purinergic receptor p2y, g-protein coupled, 14  Clec7a - c-type lectin domain family 7, member a  Acvr1 - activin a receptor, type 1  Ccrl2 - chemokine (c-c motif) receptor-like 2  Il3ra - interleukin 3 receptor, alpha chain  P2rx4 - purinergic receptor p2x, ligand-gated ion channel 4  Procr - protein c receptor, endothelial |
| GO:0042610 | CD8 receptor binding | 8.9E-5 | 2.91E-2 | 32.03 (17680,9,184,3) | H2-D1 - histocompatibility 2, d region locus 1  H2-M3 - histocompatibility 2, m region locus 3  H2-K1 - histocompatibility 2, k1, k region |
| GO:0005126 | cytokine receptor binding | 1.15E-4 | 3.5E-2 | 3.96 (17680,267,184,11) | Cxcl2 - chemokine (c-x-c motif) ligand 2  Ccl2 - chemokine (c-c motif) ligand 2  Ccl17 - chemokine (c-c motif) ligand 17  Csf2 - colony stimulating factor 2 (granulocyte-macrophage)  Il1rn - interleukin 1 receptor antagonist  Ngfrap1 - nerve growth factor receptor (tnfrsf16) associated protein 1  Ccl3 - chemokine (c-c motif) ligand 3  Ccl4 - chemokine (c-c motif) ligand 4  Ccrl2 - chemokine (c-c motif) receptor-like 2  Csf1 - colony stimulating factor 1 (macrophage)  Ccl5 - chemokine (c-c motif) ligand 5 |
| GO:0005515 | protein binding | 1.61E-4 | 4.61E-2 | 1.28 (17680,8582,184,114) | Decr1 - 2,4-dienoyl coa reductase 1, mitochondrial  Akap11 - a kinase (prka) anchor protein 11  Prkcb - protein kinase c, beta Ipo4 - importin 4  Socs3 - suppressor of cytokine signaling 3  Snx10 - sorting nexin 10  Dab2 - disabled 2, mitogen-responsive phosphoprotein  H2-M3 - histocompatibility 2, m region locus 3  Gch1 - gtp cyclohydrolase 1  Dach1 - dachshund 1 (drosophila)  H2-DMa - histocompatibility 2, class ii, locus dma  Neto2 - neuropilin (nrp) and tolloid (tll)-like 2  Pde4b - phosphodiesterase 4b, camp specific  Fabp5 - fatty acid binding protein 5, epidermal  Slc11a1 - solute carrier family 11 (proton-coupled divalent metal ion transporters), member 1  Padi2 - peptidyl arginine deiminase, type ii  Acvr1 - activin a receptor, type 1  Parvb - parvin, beta  Lpxn - leupaxin  Pilra - paired immunoglobin-like type 2 receptor alpha  Zhx2 - zinc fingers and homeoboxes 2  Mrc1 - mannose receptor, c type 1  Ikbke - inhibitor of kappab kinase epsilon  Icam1 - intercellular adhesion molecule 1  Ctsb - cathepsin b  Trem2 - triggering receptor expressed on myeloid cells 2  Cst7 - cystatin f (leukocystatin)  Cd274 - cd274 antigen  Il1rl1 - interleukin 1 receptor-like 1  Hck - hemopoietic cell kinase  Ifi203 - interferon activated gene 203  Evl - ena-vasodilator stimulated phosphoprotein  Ctss - cathepsin s  Ccr5 - chemokine (c-c motif) receptor 5  Casp1 - caspase 1  Sirpa - signal-regulatory protein alpha  Ifi27 - interferon, alpha-inducible protein 27  Uty - ubiquitously transcribed tetratricopeptide repeat gene, y chromosome  Inhba - inhibin beta-a  Hdc - histidine decarboxylase  Runx1 - runt related transcription factor 1  Nfkbie - nuclear factor of kappa light polypeptide gene enhancer in b cells inhibitor, epsilon  Mgll - monoglyceride lipase  Mtmr9 - myotubularin related protein 9  Psph - phosphoserine phosphatase  Trf - transferrin  Fpr1 - formyl peptide receptor 1  Fpr2 - formyl peptide receptor 2  Cd74 - cd74 antigen (invariant polypeptide of major histocompatibility complex, class ii antigen-associated)  Cd9 - cd9 antigen  Mmp2 - matrix metallopeptidase 2  Mmp13 - matrix metallopeptidase 13  Lpp - lim domain containing preferred translocation partner in lipoma  Alcam - activated leukocyte cell adhesion molecule  Junb - jun-b oncogene  Emb - embigin  Saa3 - serum amyloid a 3  P2rx4 - purinergic receptor p2x, ligand-gated ion channel 4  Lgals3bp - lectin, galactoside-binding, soluble, 3 binding protein  Gpnmb - glycoprotein (transmembrane) nmb  Itgax - integrin alpha x  Ccl2 - chemokine (c-c motif) ligand 2  Ccl17 - chemokine (c-c motif) ligand 17  Cp - ceruloplasmin  Tnfrsf1b - tumor necrosis factor receptor superfamily, member 1b  Clec4n - c-type lectin domain family 4, member n  Fmnl2 - formin-like 2  Tnfrsf9 - tumor necrosis factor receptor superfamily, member 9  Glb1 - galactosidase, beta 1  Aldoc - aldolase c, fructose-bisphosphate  Actr3b - arp3 actin-related protein 3b  Ifi205 - interferon activated gene 205  Mpp1 - membrane protein, palmitoylated  Cd34 - cd34 antigen  Pygl - liver glycogen phosphorylase  Ngfrap1 - nerve growth factor receptor (tnfrsf16) associated protein 1  Ccrl2 - chemokine (c-c motif) receptor-like 2  Nenf - neuron derived neurotrophic factor  Tnfaip2 - tumor necrosis factor, alpha-induced protein 2  Pld4 - phospholipase d family, member 4  Tnfaip3 - tumor necrosis factor, alpha-induced protein 3  AF251705 - cdna sequence af251705  Cebpa - ccaat/enhancer binding protein (c/ebp), alpha  Rnf157 - ring finger protein 157  Myo7a - myosin viia  Fgr - gardner-rasheed feline sarcoma viral (fgr) oncogene homolog  Fgl2 - fibrinogen-like protein 2  Cxcl2 - chemokine (c-x-c motif) ligand 2  Csf1r - colony stimulating factor 1 receptor  Csf2ra - colony stimulating factor 2 receptor, alpha, low-affinity (granulocyte-macrophage)  Csf2 - colony stimulating factor 2 (granulocyte-macrophage)  Ccl3 - chemokine (c-c motif) ligand 3  Ccl4 - chemokine (c-c motif) ligand 4  Bmi1 - bmi1 polycomb ring finger oncogene  Csf1 - colony stimulating factor 1 (macrophage)  Ccl5 - chemokine (c-c motif) ligand 5  Procr - protein c receptor, endothelial  H2-D1 - histocompatibility 2, d region locus 1  Cd200r3 - cd200 receptor 3  Lgals3 - lectin, galactose binding, soluble 3  Igfbp7 - insulin-like growth factor binding protein 7  H2-Ab1 - histocompatibility 2, class ii antigen a, beta 1  H2-Aa - histocompatibility 2, class ii antigen a, alpha  Atrnl1 - attractin like 1  Thy1 - thymus cell antigen 1, theta  H2-K1 - histocompatibility 2, k1, k region  H2-Eb1 - histocompatibility 2, class ii antigen e beta  Zeb2 - zinc finger e-box binding homeobox 2  Tgm2 - transglutaminase 2, c polypeptide  Kdm5d - lysine (k)-specific demethylase 5d  Il1rn - interleukin 1 receptor antagonist  Il3ra - interleukin 3 receptor, alpha chain  Egr2 - early growth response 2  Mfge8 - milk fat globule-egf factor 8 protein |
| GO:0048018 | receptor ligand activity | 1.91E-4 | 5.13E-2 | 3.09 (17680,435,184,14) | Ccl2 - chemokine (c-c motif) ligand 2  Lgals3 - lectin, galactose binding, soluble 3  Ccl17 - chemokine (c-c motif) ligand 17  Cxcl2 - chemokine (c-x-c motif) ligand 2  Inhba - inhibin beta-a  Il1rn - interleukin 1 receptor antagonist  Csf2 - colony stimulating factor 2 (granulocyte-macrophage)  Ccl3 - chemokine (c-c motif) ligand 3  Ccl4 - chemokine (c-c motif) ligand 4  Saa3 - serum amyloid a 3  Nenf - neuron derived neurotrophic factor  Csf1 - colony stimulating factor 1 (macrophage)  Ccl5 - chemokine (c-c motif) ligand 5  Gpnmb - glycoprotein (transmembrane) nmb |
| GO:0004888 | transmembrane signaling receptor activity | 2.03E-4 | 5.17E-2 | 2.52 (17680,724,184,19) | Ccr5 - chemokine (c-c motif) receptor 5  Mrc1 - mannose receptor, c type 1  Il7r - interleukin 7 receptor  AF251705 - cdna sequence af251705  Tnfrsf1b - tumor necrosis factor receptor superfamily, member 1b  Trem2 - triggering receptor expressed on myeloid cells 2  Fpr1 - formyl peptide receptor 1  Emr4 - egf-like module containing, mucin-like, hormone receptor-like sequence 4  Il1rl1 - interleukin 1 receptor-like 1  Fpr2 - formyl peptide receptor 2  Cd74 - cd74 antigen (invariant polypeptide of major histocompatibility complex, class ii antigen-associated)  Csf1r - colony stimulating factor 1 receptor  Csf2ra - colony stimulating factor 2 receptor, alpha, low-affinity (granulocyte-macrophage)  Plxnc1 - plexin c1  P2ry14 - purinergic receptor p2y, g-protein coupled, 14  Acvr1 - activin a receptor, type 1  Il3ra - interleukin 3 receptor, alpha chain  Ccrl2 - chemokine (c-c motif) receptor-like 2  P2rx4 - purinergic receptor p2x, ligand-gated ion channel 4 |
| GO:0023023 | MHC protein complex binding | 2.28E-4 | 5.49E-2 | 24.02 (17680,12,184,3) | Cd74 - cd74 antigen (invariant polypeptide of major histocompatibility complex, class ii antigen-associated)  H2-Oa - histocompatibility 2, o region alpha locus  H2-DMa - histocompatibility 2, class ii, locus dma |
| GO:0005488 | binding | 2.7E-4 | 6.18E-2 | 1.17 (17680,12063,184,147) | Dsc2 - desmocollin 2  Decr1 - 2,4-dienoyl coa reductase 1, mitochondrial  Akap11 - a kinase (prka) anchor protein 11  Prkcb - protein kinase c, beta  H2-Oa - histocompatibility 2, o region alpha locus  Ipo4 - importin 4  Socs3 - suppressor of cytokine signaling 3  Snx10 - sorting nexin 10  Hivep3 - human immunodeficiency virus type i enhancer binding protein 3 Dab2 - disabled 2, mitogen-responsive phosphoprotein  H2-M3 - histocompatibility 2, m region locus 3  Gch1 - gtp cyclohydrolase 1  Dach1 - dachshund 1 (drosophila)  H2-DMa - histocompatibility 2, class ii, locus dma  Neto2 - neuropilin (nrp) and tolloid (tll)-like 2  Ear2 - eosinophil-associated, ribonuclease a family, member 2  Pde4b - phosphodiesterase 4b, camp specific  Ube2h - ubiquitin-conjugating enzyme e2h  Fabp5 - fatty acid binding protein 5, epidermal  Sestd1 - sec14 and spectrin domains 1  Slc11a1 - solute carrier family 11 (proton-coupled divalent metal ion transporters), member 1  Padi2 - peptidyl arginine deiminase, type ii  Mcts1 - malignant t cell amplified sequence 1  Acvr1 - activin a receptor, type 1  Parvb - parvin, beta  Lpxn - leupaxin  Pilra - paired immunoglobin-like type 2 receptor alpha  Zhx2 - zinc fingers and homeoboxes 2  Cyp4f18 - cytochrome p450, family 4, subfamily f, polypeptide 18  Mrc1 - mannose receptor, c type 1  Ikbke - inhibitor of kappab kinase epsilon  Ctsb - cathepsin b  Icam1 - intercellular adhesion molecule 1  Inpp4b - inositol polyphosphate-4-phosphatase, type ii  Trem2 - triggering receptor expressed on myeloid cells 2  Cd274 - cd274 antigen  Cst7 - cystatin f (leukocystatin)  Emr4 - egf-like module containing, mucin-like, hormone receptor-like sequence 4  Il1rl1 - interleukin 1 receptor-like 1  Hck - hemopoietic cell kinase  Ifi203 - interferon activated gene 203  Evl - ena-vasodilator stimulated phosphoprotein  Parp12 - poly (adp-ribose) polymerase family, member 12  Ctss - cathepsin s  Ccr5 - chemokine (c-c motif) receptor 5  Casp1 - caspase 1  Sirpa - signal-regulatory protein alpha  Ifi27 - interferon, alpha-inducible protein 27  Epx - eosinophil peroxidase  Uty - ubiquitously transcribed tetratricopeptide repeat gene, y chromosome Inhba - inhibin beta-a  Dhx40 - deah (asp-glu-ala-his) box polypeptide 40  Hdc - histidine decarboxylase  Lpcat2 - lysophosphatidylcholine acyltransferase 2  Runx1 - runt related transcription factor 1  Nfkbie - nuclear factor of kappa light polypeptide gene enhancer in b cells inhibitor, epsilon  Mgll - monoglyceride lipase  Crip1 - cysteine-rich protein 1 (intestinal)  Zmat3 - zinc finger matrin type 3  Ddx3y - dead (asp-glu-ala-asp) box polypeptide 3, y-linked  Mtmr9 - myotubularin related protein 9  Trf - transferrin  Psph - phosphoserine phosphatase  Fpr1 - formyl peptide receptor 1  Eif2s3y - eukaryotic translation initiation factor 2, subunit 3, structural gene y-linked  Fpr2 - formyl peptide receptor 2  Cd74 - cd74 antigen (invariant polypeptide of major histocompatibility complex, class ii antigen-associated)  Cd9 - cd9 antigen  Mmp2 - matrix metallopeptidase 2  Mmp13 - matrix metallopeptidase 13  Lpp - lim domain containing preferred translocation partner in lipoma Alcam - activated leukocyte cell adhesion molecule  Junb - jun-b oncogene  Mmp12 - matrix metallopeptidase 12  Emb - embigin  Saa3 - serum amyloid a 3  P2rx4 - purinergic receptor p2x, ligand-gated ion channel 4  Lgals3bp - lectin, galactoside-binding, soluble, 3 binding protein  Gpnmb - glycoprotein (transmembrane) nmb  Itgax - integrin alpha x  Ccl2 - chemokine (c-c motif) ligand 2  Ccl17 - chemokine (c-c motif) ligand 17  Cp - ceruloplasmin  Tnfrsf1b - tumor necrosis factor receptor superfamily, member 1b  Clec4n - c-type lectin domain family 4, member n  Fmnl2 - formin-like 2  Alox15 - arachidonate 15-lipoxygenase  Tnfrsf9 - tumor necrosis factor receptor superfamily, member 9  Glb1 - galactosidase, beta 1  Aldoc - aldolase c, fructose-bisphosphate  Mmp8 - matrix metallopeptidase 8  Actr3b - arp3 actin-related protein 3b  Ifi205 - interferon activated gene 205  Mpp1 - membrane protein, palmitoylated  Cd34 - cd34 antigen  Polr3k - polymerase (rna) iii (dna directed) polypeptide k  Pygl - liver glycogen phosphorylase  Ngfrap1 - nerve growth factor receptor (tnfrsf16) associated protein 1 Hddc2 - hd domain containing 2  Ccrl2 - chemokine (c-c motif) receptor-like 2  Stxbp6 - syntaxin binding protein 6 (amisyn)  Nenf - neuron derived neurotrophic factor  Acss1 - acyl-coa synthetase short-chain family member 1  Pld4 - phospholipase d family, member 4  Tnfaip2 - tumor necrosis factor, alpha-induced protein 2  Tnfaip3 - tumor necrosis factor, alpha-induced protein 3  Rnf128 - ring finger protein 128  AF251705 - cdna sequence af251705  Cebpa - ccaat/enhancer binding protein (c/ebp), alpha  Rnf157 - ring finger protein 157  Acsl4 - acyl-coa synthetase long-chain family member 4  Myo7a - myosin viia  Siglec5 - sialic acid binding ig-like lectin 5  Fgr - gardner-rasheed feline sarcoma viral (fgr) oncogene homolog  Fgl2 - fibrinogen-like protein 2  Cxcl2 - chemokine (c-x-c motif) ligand 2  Csf1r - colony stimulating factor 1 receptor  Csf2ra - colony stimulating factor 2 receptor, alpha, low-affinity (granulocyte-macrophage)  Csf2 - colony stimulating factor 2 (granulocyte-macrophage)  Clec7a - c-type lectin domain family 7, member a  Fam20c - family with sequence similarity 20, member c  Ccl3 - chemokine (c-c motif) ligand 3  Ccl4 - chemokine (c-c motif) ligand 4  Bmi1 - bmi1 polycomb ring finger oncogene  Csf1 - colony stimulating factor 1 (macrophage)  Ccl5 - chemokine (c-c motif) ligand 5  Procr - protein c receptor, endothelial  Igfbp7 - insulin-like growth factor binding protein 7  H2-D1 - histocompatibility 2, d region locus 1  Cd200r3 - cd200 receptor 3  Lgals3 - lectin, galactose binding, soluble 3  H2-Ab1 - histocompatibility 2, class ii antigen a, beta 1  H2-Aa - histocompatibility 2, class ii antigen a, alpha  Cpne7 - copine vii  Atrnl1 - attractin like 1  Thy1 - thymus cell antigen 1, theta  H2-K1 - histocompatibility 2, k1, k region  Zeb2 - zinc finger e-box binding homeobox 2  H2-Eb1 - histocompatibility 2, class ii antigen e beta  Tgm2 - transglutaminase 2, c polypeptide  Kdm5d - lysine (k)-specific demethylase 5d  Il1rn - interleukin 1 receptor antagonist  Il3ra - interleukin 3 receptor, alpha chain  Egr3 - early growth response 3  Egr2 - early growth response 2  Mfge8 - milk fat globule-egf factor 8 protein  Prg2 - proteoglycan 2, bone marrow |
| GO:0033218 | amide binding | 3.12E-4 | 6.79E-2 | 3.29 (17680,350,184,12) | Cd74 - cd74 antigen (invariant polypeptide of major histocompatibility complex, class ii antigen-associated)  Crip1 - cysteine-rich protein 1 (intestinal)  H2-D1 - histocompatibility 2, d region locus 1  Inhba - inhibin beta-a  Ipo4 - importin 4  H2-Ab1 - histocompatibility 2, class ii antigen a, beta 1  H2-Aa - histocompatibility 2, class ii antigen a, alpha  Ctsb - cathepsin b  H2-M3 - histocompatibility 2, m region locus 3  Trem2 - triggering receptor expressed on myeloid cells 2  H2-K1 - histocompatibility 2, k1, k region  Fpr2 - formyl peptide receptor 2 |
| GO:0034988 | Fc-gamma receptor I complex binding | 3.21E-4 | 6.68E-2 | 64.06 (17680,3,184,2) | Lgals3 - lectin, galactose binding, soluble 3  Fgr - gardner-rasheed feline sarcoma viral (fgr) oncogene homolog |
| GO:0031726 | CCR1 chemokine receptor binding | 3.21E-4 | 6.39E-2 | 64.06 (17680,3,184,2) | Ccl4 - chemokine (c-c motif) ligand 4  Ccl5 - chemokine (c-c motif) ligand 5 |
| GO:0030545 | receptor regulator activity | 4.26E-4 | 8.13E-2 | 2.86 (17680,471,184,14) | Ccl2 - chemokine (c-c motif) ligand 2  Lgals3 - lectin, galactose binding, soluble 3  Ccl17 - chemokine (c-c motif) ligand 17  Cxcl2 - chemokine (c-x-c motif) ligand 2  Inhba - inhibin beta-a  Il1rn - interleukin 1 receptor antagonist  Csf2 - colony stimulating factor 2 (granulocyte-macrophage)  Ccl3 - chemokine (c-c motif) ligand 3  Ccl4 - chemokine (c-c motif) ligand 4  Saa3 - serum amyloid a 3  Nenf - neuron derived neurotrophic factor  Csf1 - colony stimulating factor 1 (macrophage)  Ccl5 - chemokine (c-c motif) ligand 5  Gpnmb - glycoprotein (transmembrane) nmb |
| GO:0044877 | protein-containing complex binding | 6.19E-4 | 1.13E-1 | 1.92 (17680,1400,184,28) | Cebpa - ccaat/enhancer binding protein (c/ebp), alpha  Icam1 - intercellular adhesion molecule 1  Ctsb - cathepsin b  Myo7a - myosin viia  Trem2 - triggering receptor expressed on myeloid cells 2  Fgr - gardner-rasheed feline sarcoma viral (fgr) oncogene homolog  Cd74 - cd74 antigen (invariant polypeptide of major histocompatibility complex, class ii antigen-associated)  Cd9 - cd9 antigen  H2-Oa - histocompatibility 2, o region alpha locus  Mmp13 - matrix metallopeptidase 13  Dab2 - disabled 2, mitogen-responsive phosphoprotein  Mmp12 - matrix metallopeptidase 12  Gch1 - gtp cyclohydrolase 1  H2-DMa - histocompatibility 2, class ii, locus dma  Gpnmb - glycoprotein (transmembrane) nmb  Ctss - cathepsin s  H2-D1 - histocompatibility 2, d region locus 1  Lgals3 - lectin, galactose binding, soluble 3  H2-Ab1 - histocompatibility 2, class ii antigen a, beta 1  H2-Aa - histocompatibility 2, class ii antigen a, alpha  Fmnl2 - formin-like 2  Thy1 - thymus cell antigen 1, theta  Aldoc - aldolase c, fructose-bisphosphate  H2-K1 - histocompatibility 2, k1, k region  Actr3b - arp3 actin-related protein 3b  Inhba - inhibin beta-a  Acvr1 - activin a receptor, type 1  Mfge8 - milk fat globule-egf factor 8 protein |
| GO:0045145 | single-stranded DNA 5'-3' exodeoxyribonuclease activity | 6.38E-4 | 1.12E-1 | 48.04 (17680,4,184,2) | Pld3 - phospholipase d family, member 3  Pld4 - phospholipase d family, member 4 |
| GO:0005124 | scavenger receptor binding | 6.38E-4 | 1.08E-1 | 48.04 (17680,4,184,2) | Fpr1 - formyl peptide receptor 1  Fpr2 - formyl peptide receptor 2 |

Differentially expressed RNA was ranked according to the p-values of differential expression and degree of enrichment compared with the total number of expressed genes analysed (17680 GO terms). The GOrilla database updated on Mar 6, 2021 was used.

**'P-value'** is the enrichment p-value computed according to the mHG or HG model. This p-value is not corrected for multiple testing of 4580 GO terms.

**'FDR q-value'** is the correction of the above p-value for multiple testing using the Benjamini and Hochberg (1995) method. Namely, for the ith term (ranked according to p-value) the FDR q-value is (p-value * number of GO terms) / i.

**Enrichment (N, B, n, b)** is defined as follows:

N - is the total number of genes

B - is the total number of genes associated with a specific GO term

n - is the number of genes in the top of the user's input list or in the target set when appropriate b - is the number of genes in the intersection

Enrichment = (b/n) / (B/N)

**Genes:** For each GO term you can see the list of associated genes that appear in the optimal top of the list. Each gene name is specified by gene symbol followed by a short description of the gene
