# Supplementary material for: Proteomic and transcriptomic characterisation of FIA10, a novel murine leukemic cell line that metastasizes into the brain
Source: PLoS One. 2024 Jan 12;19(1):e0295641. doi: 10.1371/journal.pone.0295641 (PMC10786371; doi:10.1371/journal.pone.0295641)
Supplement: S8 Table — (DOCX) [file pone.0295641.s013.docx]

**Gene Ontology: Cellular component FIA10 vs FIA18 RNA upregulated**

| **GO term** | **Description** | **P-value** | **FDR q-value** | **Enrichment (N, B, n, b)** | **Genes** |
| --- | --- | --- | --- | --- | --- |
| GO:0042611 | MHC protein complex | 9.94E-14 | 1.94E-10 | 45.51 (17680,19,184,9) | Cd74 - cd74 antigen (invariant polypeptide of major histocompatibility complex, class ii antigen-associated)  H2-Oa - histocompatibility 2, o region alpha locus  H2-D1 - histocompatibility 2, d region locus 1  H2-Ab1 - histocompatibility 2, class ii antigen a, beta 1  H2-Aa - histocompatibility 2, class ii antigen a, alpha  H2-M3 - histocompatibility 2, m region locus 3  H2-DMa - histocompatibility 2, class ii, locus dma  H2-K1 - histocompatibility 2, k1, k region  H2-Eb1 - histocompatibility 2, class ii antigen e beta |
| GO:0009897 | external side of plasma membrane | 1.43E-12 | 1.4E-9 | 6.08 (17680,379,184,24) | Itgax - integrin alpha x  Ccr5 - chemokine (c-c motif) receptor 5  Cd200r3 - cd200 receptor 3  Lgals3 - lectin, galactose binding, soluble 3  H2-D1 - histocompatibility 2, d region locus 1  Il7r - interleukin 7 receptor  H2-Ab1 - histocompatibility 2, class ii antigen a, beta 1  H2-Aa - histocompatibility 2, class ii antigen a, alpha  Icam1 - intercellular adhesion molecule 1  Ctsb - cathepsin b  Tnfrsf9 - tumor necrosis factor receptor superfamily, member 9  Cd274 - cd274 antigen  Thy1 - thymus cell antigen 1, theta  H2-K1 - histocompatibility 2, k1, k region  Il1rl1 - interleukin 1 receptor-like 1  Cd74 - cd74 antigen (invariant polypeptide of major histocompatibility complex, class ii antigen-associated)  Cd9 - cd9 antigen  Cd34 - cd34 antigen  Csf2ra - colony stimulating factor 2 receptor, alpha, low-affinity (granulocyte-macrophage)  Alcam - activated leukocyte cell adhesion molecule  H2-M3 - histocompatibility 2, m region locus 3  Ccrl2 - chemokine (c-c motif) receptor-like 2  Il3ra - interleukin 3 receptor, alpha chain  Mfge8 - milk fat globule-egf factor 8 protein |
| GO:0044421 | extracellular region part | 1.65E-12 | 1.07E-9 | 2.89 (17680,1696,184,51) | Trf - transferrin  Cd74 - cd74 antigen (invariant polypeptide of major histocompatibility complex, class ii antigen-associated)  Cd9 - cd9 antigen  Mmp2 - matrix metallopeptidase 2  Mmp13 - matrix metallopeptidase 13  Mmp12 - matrix metallopeptidase 12  H2-M3 - histocompatibility 2, m region locus 3  Saa3 - serum amyloid a 3  Lgals3bp - lectin, galactoside-binding, soluble, 3 binding protein  Ear2 - eosinophil-associated, ribonuclease a family, member 2  Ccl2 - chemokine (c-c motif) ligand 2  Ccl17 - chemokine (c-c motif) ligand 17  Cp - ceruloplasmin  Tnfrsf9 - tumor necrosis factor receptor superfamily, member 9  Fabp5 - fatty acid binding protein 5, epidermal  Glb1 - galactosidase, beta 1  Mmp8 - matrix metallopeptidase 8  Cd34 - cd34 antigen  Nenf - neuron derived neurotrophic factor  Col22a1 - collagen, type xxii, alpha 1  Ctsb - cathepsin b  Icam1 - intercellular adhesion molecule 1  Mcpt8 - mast cell protease 8  Ctso - cathepsin o  Cst7 - cystatin f (leukocystatin)  Cd274 - cd274 antigen  Fgl2 - fibrinogen-like protein 2  Il1rl1 - interleukin 1 receptor-like 1  Cxcl2 - chemokine (c-x-c motif) ligand 2  Csf2 - colony stimulating factor 2 (granulocyte-macrophage)  Fam20c -family with sequence similarity 20, member c  Ccl3 - chemokine (c-c motif) ligand 3  Ccl4 - chemokine (c-c motif) ligand 4  Csf1 - colony stimulating factor 1 (macrophage)  Ccl5 - chemokine (c-c motif) ligand 5  Ctss - cathepsin s  Procr - protein c receptor, endothelial  Igfbp7 - insulin-like growth factor binding protein 7  H2-D1 - histocompatibility 2, d region locus 1  Lgals3 - lectin, galactose binding, soluble 3  Epx - eosinophil peroxidase  Wfdc17 - wap four-disulfide core domain 17  Atrnl1 - attractin like 1  H2-K1 - histocompatibility 2, k1, k region  Tgm2 - transglutaminase 2, c polypeptide  Inhba - inhibin beta-a  Il1rn - interleukin 1 receptor antagonist  Serpinb2 - serine (or cysteine) peptidase inhibitor, clade b, member 2  Runx1 - runt related transcription factor 1  Mfge8 - milk fat globule-egf factor 8 protein  Prg2 - proteoglycan 2, bone marrow |
| GO:0005615 | extracellular space | 6.52E-12 | 3.19E-9 | 3.12 (17680,1353,184,44) | Mcpt8 - mast cell protease 8  Ctsb - cathepsin b  Trf - transferrin  Icam1 - intercellular adhesion molecule 1  Ctso - cathepsin o  Cst7 - cystatin f (leukocystatin)  Fgl2 - fibrinogen-like protein 2  Il1rl1 - interleukin 1 receptor-like 1  Cd74 - cd74 antigen (invariant polypeptide of major histocompatibility complex, class ii antigen-associated)  Cxcl2 - chemokine (c-x-c motif) ligand 2  Mmp2 - matrix metallopeptidase 2  Csf2 - colony stimulating factor 2 (granulocyte-macrophage)  Mmp13 - matrix metallopeptidase 13  Fam20c - family with sequence similarity 20, member c  Mmp12 - matrix metallopeptidase 12  Ccl3 - chemokine (c-c motif) ligand 3  H2-M3 - histocompatibility 2, m region locus 3  Ccl4 - chemokine (c-c motif) ligand 4  Saa3 - serum amyloid a 3  Lgals3bp - lectin, galactoside-binding, soluble, 3 binding protein  Csf1 - colony stimulating factor 1 (macrophage)  Ccl5 - chemokine (c-c motif) ligand 5  Ctss - cathepsin s  Procr - protein c receptor, endothelial  Ear2 - eosinophil-associated, ribonuclease a family, member 2  Ccl2 - chemokine (c-c motif) ligand 2  Igfbp7 - insulin-like growth factor binding protein 7  Lgals3 - lectin, galactose binding, soluble 3  H2-D1 - histocompatibility 2, d region locus 1  Ccl17 - chemokine (c-c motif) ligand 17  Cp - ceruloplasmin  Tnfrsf9 - tumor necrosis factor receptor superfamily, member 9  Epx - eosinophil peroxidase  Fabp5 - fatty acid binding protein 5, epidermal  Wfdc17 - wap four-disulfide core domain 17  Glb1 - galactosidase, beta 1  Mmp8 - matrix metallopeptidase 8  H2-K1 - histocompatibility 2, k1, k region  Inhba - inhibin beta-a  Il1rn - interleukin 1 receptor antagonist  Serpinb2 - serine (or cysteine) peptidase inhibitor, clade b, member 2  Nenf - neuron derived neurotrophic factor  Col22a1 - collagen, type xxii, alpha 1  Mfge8 - milk fat globule-egf factor 8 protein |
| GO:0098552 | side of membrane | 8.82E-12 | 3.45E-9 | 5.33 (17680,451,184,25) | Icam1 - intercellular adhesion molecule 1  Ctsb - cathepsin b  Gm2a - gm2 ganglioside activator protein  Cd274 - cd274 antigen  Il1rl1 - interleukin 1 receptor-like 1  Cd74 - cd74 antigen (invariant polypeptide of major histocompatibility complex, class ii antigen-associated)  Cd9 - cd9 antigen  Csf2ra - colony stimulating factor 2 receptor, alpha, low-affinity (granulocyte-macrophage)  Alcam - activated leukocyte cell adhesion molecule  H2-M3 - histocompatibility 2, m region locus 3  Itgax - integrin alpha x  Lgals3 - lectin, galactose binding, soluble 3  H2-D1 - histocompatibility 2, d region locus 1  Cd200r3 - cd200 receptor 3  Ccr5 - chemokine (c-c motif) receptor 5  Il7r - interleukin 7 receptor  H2-Ab1 - histocompatibility 2, class ii antigen a, beta 1  H2-Aa - histocompatibility 2, class ii antigen a, alpha  Tnfrsf9 - tumor necrosis factor receptor superfamily, member 9  Thy1 - thymus cell antigen 1, theta  H2-K1 - histocompatibility 2, k1, k region  Cd34 - cd34 antigen  Ccrl2 - chemokine (c-c motif) receptor-like 2  Il3ra - interleukin 3 receptor, alpha chain  Mfge8 - milk fat globule-egf factor 8 protein |
| GO:0042613 | MHC class II protein complex | 9.59E-11 | 3.12E-8 | 64.06 (17680,9,184,6) | Cd74 - cd74 antigen (invariant polypeptide of major histocompatibility complex, class ii antigen-associated)  H2-Oa - histocompatibility 2, o region alpha locus  H2-Ab1 - histocompatibility 2, class ii antigen a, beta 1  H2-Aa - histocompatibility 2, class ii antigen a, alpha  H2-DMa - histocompatibility 2, class ii, locus dma  H2-Eb1 - histocompatibility 2, class ii antigen e beta |
| GO:0044459 | plasma membrane part | 5.3E-8 | 1.48E-5 | 2.11 (17680,2413,184,53) | Trf - transferrin  Slc22a3 - solute carrier family 22 (organic cation transporter), member 3  Prkcb - protein kinase c, beta  Cd74 - cd74 antigen (invariant polypeptide of major histocompatibility complex, class ii antigen-associated)  Cd9 - cd9 antigen  H2-Oa - histocompatibility 2, o region alpha locus  Alcam - activated leukocyte cell adhesion molecule  Dab2 - disabled 2, mitogen-responsive phosphoprotein  H2-M3 - histocompatibility 2, m region locus 3  Emb - embigin  P2rx4 - purinergic receptor p2x, ligand-gated ion channel 4  H2-DMa - histocompatibility 2, class ii, locus dma  Gpnmb - glycoprotein (transmembrane) nmb  Itgax - integrin alpha x  Pde4b - phosphodiesterase 4b, camp specific  Cp - ceruloplasmin  Alox15 - arachidonate 15-lipoxygenase  Tnfrsf9 - tumor necrosis factor receptor superfamily, member 9  Cd34 - cd34 antigen  Plxnc1 - plexin c1  Acvr1 - activin a receptor, type 1  Ccrl2 - chemokine (c-c motif) receptor-like 2  Cyp4f18 - cytochrome p450, family 4, subfamily f, polypeptide 18  Ctsb - cathepsin b  Icam1 - intercellular adhesion molecule 1  Myo7a - myosin viia  Slc6a12 - solute carrier family 6 (neurotransmitter transporter, betaine/gaba), member 12  Gm2a - gm2 ganglioside activator protein  Trem2 - triggering receptor expressed on myeloid cells 2  Fgr - gardner-rasheed feline sarcoma viral (fgr) oncogene homolog  Cd274 - cd274 antigen  Ppap2c - phosphatidic acid phosphatase type 2c  Emr4 - egf-like module containing, mucin-like, hormone receptor-like sequence 4  Il1rl1 - interleukin 1 receptor-like 1  Hck - hemopoietic cell kinase  Csf1r - colony stimulating factor 1 receptor  Csf2ra - colony stimulating factor 2 receptor, alpha, low-affinity (granulocyte-macrophage)  Cd200r3 - cd200 receptor 3  Ccr5 - chemokine (c-c motif) receptor 5  H2-D1 - histocompatibility 2, d region locus 1  Lgals3 - lectin, galactose binding, soluble 3  Il7r - interleukin 7 receptor  Ms4a4b - membrane-spanning 4-domains, subfamily a, member 4b  H2-Ab1 - histocompatibility 2, class ii antigen a, beta 1  H2-Aa - histocompatibility 2, class ii antigen a, alpha  Sirpa - signal-regulatory protein alpha  Thy1 - thymus cell antigen 1, theta  Atp6v0d2 - atpase, h+ transporting, lysosomal v0 subunit d2  H2-K1 - histocompatibility 2, k1, k region  H2-Eb1 - histocompatibility 2, class ii antigen e beta  Tgm2 - transglutaminase 2, c polypeptide  Il3ra - interleukin 3 receptor, alpha chain  Mfge8 - milk fat globule-egf factor 8 protein |
| GO:0000323 | lytic vacuole | 1.32E-7 | 3.23E-5 | 4.23 (17680,432,184,19) | H2-Aa - histocompatibility 2, class ii antigen a, alpha  Ctsb - cathepsin b  Ctso - cathepsin o  Gm2a - gm2 ganglioside activator protein  Glb1 - galactosidase, beta 1  Siglec5 - sialic acid binding ig-like lectin 5  Cst7 - cystatin f (leukocystatin)  Slc11a1 - solute carrier family 11 (proton-coupled divalent metal ion transporters), member 1  Hck - hemopoietic cell kinase  Ifi30 - interferon gamma inducible protein 30  Cd74 - cd74 antigen (invariant polypeptide of major histocompatibility complex, class ii antigen-associated)  Slc15a3 - solute carrier family 15, member 3  Cd34 - cd34 antigen  Mmp13 - matrix metallopeptidase 13  Pld3 - phospholipase d family, member 3  H2-DMa - histocompatibility 2, class ii, locus dma  Tnfaip3 - tumor necrosis factor, alpha-induced protein 3  Slc2a6 - solute carrier family 2 (facilitated glucose transporter), member 6  Ctss - cathepsin s |
| GO:0005764 | lysosome | 1.32E-7 | 2.87E-5 | 4.23 (17680,432,184,19) | H2-Aa - histocompatibility 2, class ii antigen a, alpha  Ctsb - cathepsin b  Ctso - cathepsin o  Gm2a - gm2 ganglioside activator protein  Glb1 - galactosidase, beta 1  Siglec5 - sialic acid binding ig-like lectin 5  Cst7 - cystatin f (leukocystatin)  Slc11a1 - solute carrier family 11 (proton-coupled divalent metal ion transporters), member 1  Hck - hemopoietic cell kinase  Ifi30 - interferon gamma inducible protein 30  Cd74 - cd74 antigen (invariant polypeptide of major histocompatibility complex, class ii antigen-associated)  Slc15a3 - solute carrier family 15, member 3  Cd34 - cd34 antigen  Mmp13 - matrix metallopeptidase 13  Pld3 - phospholipase d family, member 3  H2-DMa - histocompatibility 2, class ii, locus dma  Tnfaip3 - tumor necrosis factor, alpha-induced protein 3  Slc2a6 - solute carrier family 2 (facilitated glucose transporter), member 6  Ctss - cathepsin s |
| GO:0009986 | cell surface | 6.93E-7 | 1.35E-4 | 3.36 (17680,630,184,22) | Itgax - integrin alpha x  Ccr5 - chemokine (c-c motif) receptor 5  H2-D1 - histocompatibility 2, d region locus 1  Lgals3 - lectin, galactose binding, soluble 3  Mrc1 - mannose receptor, c type 1  H2-Ab1 - histocompatibility 2, class ii antigen a, beta 1  Ctsb - cathepsin b  Sirpa - signal-regulatory protein alpha  Icam1 - intercellular adhesion molecule 1  Trf - transferrin  Cd274 - cd274 antigen  Thy1 - thymus cell antigen 1, theta  Emr4 - egf-like module containing, mucin-like, hormone receptor-like sequence 4  Il1rl1 - interleukin 1 receptor-like 1  H2-K1 - histocompatibility 2, k1, k region  Cd74 - cd74 antigen (invariant polypeptide of major histocompatibility complex, class ii antigen-associated)  Csf1r - colony stimulating factor 1 receptor  Cd34 - cd34 antigen  Cd9 - cd9 antigen  Clec7a - c-type lectin domain family 7, member a  H2-DMa - histocompatibility 2, class ii, locus dma  Ctss - cathepsin s |
| GO:0005773 | vacuole | 7.98E-7 | 1.42E-4 | 3.76 (17680,486,184,19) | H2-Aa - histocompatibility 2, class ii antigen a, alpha  Ctsb - cathepsin b  Ctso - cathepsin o  Gm2a - gm2 ganglioside activator protein  Glb1 - galactosidase, beta 1  Siglec5 - sialic acid binding ig-like lectin 5  Cst7 - cystatin f (leukocystatin)  Slc11a1 - solute carrier family 11 (proton-coupled divalent metal ion transporters), member 1  Hck - hemopoietic cell kinase  Ifi30 - interferon gamma inducible protein 30  Cd74 - cd74 antigen (invariant polypeptide of major histocompatibility complex, class ii antigen-associated)  Slc15a3 - solute carrier family 15, member 3  Cd34 - cd34 antigen  Mmp13 - matrix metallopeptidase 13  Pld3 - phospholipase d family, member 3  H2-DMa - histocompatibility 2, class ii, locus dma  Tnfaip3 - tumor necrosis factor, alpha-induced protein 3  Slc2a6 - solute carrier family 2 (facilitated glucose transporter), member 6  Ctss - cathepsin s |
| GO:0005576 | extracellular region | 2.02E-6 | 3.29E-4 | 2.29 (17680,1511,184,36) | Mcpt8 - mast cell protease 8  Trf - transferrin  Ctsb - cathepsin b  Trem2 - triggering receptor expressed on myeloid cells 2  Cst7 - cystatin f (leukocystatin)  Fgl2 - fibrinogen-like protein 2  Il1rl1 - interleukin 1 receptor-like 1  Ifi30 - interferon gamma inducible protein 30  Cxcl2 - chemokine (c-x-c motif) ligand 2  Mmp2 - matrix metallopeptidase 2  Cd9 - cd9 antigen  Csf2 - colony stimulating factor 2 (granulocyte-macrophage)  Mmp13 - matrix metallopeptidase 13  Fam20c - family with sequence similarity 20, member c  Pga5 - pepsinogen 5, group i  Mmp12 - matrix metallopeptidase 12  Ccl3 - chemokine (c-c motif) ligand 3  Ccl4 - chemokine (c-c motif) ligand 4  Saa3 - serum amyloid a 3  Lgals3bp - lectin, galactoside-binding, soluble, 3 binding protein  Ccl5 - chemokine (c-c motif) ligand 5  Csf1 - colony stimulating factor 1 (macrophage)  Ctss - cathepsin s  Ccl2 - chemokine (c-c motif) ligand 2  Igfbp7 - insulin-like growth factor binding protein 7  Lgals3 - lectin, galactose binding, soluble 3  Cp - ceruloplasmin  Casp1 - caspase 1  Fabp5 - fatty acid binding protein 5, epidermal  Mmp8 - matrix metallopeptidase 8  Cd34 - cd34 antigen  Inhba - inhibin beta-a  Il1rn - interleukin 1 receptor antagonist  Serpinb2 - serine (or cysteine) peptidase inhibitor, clade b, member 2  Nenf - neuron derived neurotrophic factor  Mfge8 - milk fat globule-egf factor 8 protein |
| GO:0044425 | membrane part | 4.81E-6 | 7.22E-4 | 1.51 (17680,5475,184,86) | Mgll - monoglyceride lipase  Dsc2 - desmocollin 2  Trf - transferrin  Slc22a3 - solute carrier family 22 (organic cation transporter), member 3  Fpr1 - formyl peptide receptor 1  BC023829 - cdna sequence bc023829  Fpr2 - formyl peptide receptor 2  Prkcb - protein kinase c, beta  Cd74 - cd74 antigen (invariant polypeptide of major histocompatibility complex, class ii antigen-associated)  Cd9 - cd9 antigen  H2-Oa - histocompatibility 2, o region alpha locus  Socs3 - suppressor of cytokine signaling 3  Snx10 - sorting nexin 10  Alcam - activated leukocyte cell adhesion molecule  Dab2 - disabled 2, mitogen-responsive phosphoprotein  H2-M3 - histocompatibility 2, m region locus 3  Emb - embigin  P2rx4 - purinergic receptor p2x, ligand-gated ion channel 4  H2-DMa - histocompatibility 2, class ii, locus dma  Gpnmb - glycoprotein (transmembrane) nmb  Slc2a6 - solute carrier family 2 (facilitated glucose transporter), member 6  Neto2 - neuropilin (nrp) and tolloid (tll)-like 2  Itgax - integrin alpha x  Pde4b - phosphodiesterase 4b, camp specific  Cp - ceruloplasmin  Tnfrsf1b - tumor necrosis factor receptor superfamily, member 1b  Clec4n - c-type lectin domain family 4, member n  Alox15 - arachidonate 15-lipoxygenase  Tnfrsf9 - tumor necrosis factor receptor superfamily, member 9  Ms4a6d - membrane-spanning 4-domains, subfamily a, member 6d  Sestd1 - sec14 and spectrin domains 1  Slc11a1 - solute carrier family 11 (proton-coupled divalent metal ion transporters), member 1  Cd34 - cd34 antigen  Plxnc1 - plexin c1  P2ry14 - purinergic receptor p2y, g-protein coupled, 14  Acvr1 - activin a receptor, type 1  Ccrl2 - chemokine (c-c motif) receptor-like 2  Stxbp6 - syntaxin binding protein 6 (amisyn)  Pld4 - phospholipase d family, member 4  Pilra - paired immunoglobin-like type 2 receptor alpha  Cyp4f18 - cytochrome p450, family 4, subfamily f, polypeptide 18  Rnf128 - ring finger protein 128  Chst11 - carbohydrate sulfotransferase 11  Mrc1 - mannose receptor, c type 1  AF251705 - cdna sequence af251705  Ctsb - cathepsin b  Icam1 - intercellular adhesion molecule 1  Acsl4 - acyl-coa synthetase long-chain family member 4  Myo7a - myosin viia  Siglec5 - sialic acid binding ig-like lectin 5  Slc6a12 - solute carrier family 6 (neurotransmitter transporter, betaine/gaba), member 12  Gm2a - gm2 ganglioside activator protein  Trem2 - triggering receptor expressed on myeloid cells 2  Cd274 - cd274 antigen  Fgr - gardner-rasheed feline sarcoma viral (fgr) oncogene homolog  Ppap2c - phosphatidic acid phosphatase type 2c  Emr4 - egf-like module containing, mucin-like, hormone receptor-like sequence 4  Il1rl1 - interleukin 1 receptor-like 1  Hck - hemopoietic cell kinase  Slc15a3 - solute carrier family 15, member 3  Csf1r - colony stimulating factor 1 receptor  Csf2ra - colony stimulating factor 2 receptor, alpha, low-affinity (granulocyte-macrophage)  Degs1 - degenerative spermatocyte homolog 1 (drosophila)  Ms4a6c - membrane-spanning 4-domains, subfamily a, member 6c  Csf1 - colony stimulating factor 1 (macrophage)  Procr - protein c receptor, endothelial  Cd200r3 - cd200 receptor 3  Ccr5 - chemokine (c-c motif) receptor 5  H2-D1 - histocompatibility 2, d region locus 1  Lgals3 - lectin, galactose binding, soluble 3  Il7r - interleukin 7 receptor  Ms4a4b - membrane-spanning 4-domains, subfamily a, member 4b  H2-Ab1 - histocompatibility 2, class ii antigen a, beta 1  H2-Aa - histocompatibility 2, class ii antigen a, alpha  Sirpa - signal-regulatory protein alpha  Atrnl1 - attractin like 1  Thy1 - thymus cell antigen 1, theta  Atp6v0d2 - atpase, h+ transporting, lysosomal v0 subunit d2  H2-K1 - histocompatibility 2, k1, k region  H2-Eb1 - histocompatibility 2, class ii antigen e beta  Tgm2 - transglutaminase 2, c polypeptide  Slc35d1 - solute carrier family 35 (udp-glucuronic acid/udp-n-acetylgalactosamine dual transporter), member d1  Il3ra - interleukin 3 receptor, alpha chain  Pld3 - phospholipase d family, member 3  Lpcat2 - lysophosphatidylcholine acyltransferase 2  Mfge8 - milk fat globule-egf factor 8 protein |
| GO:0031012 | extracellular matrix | 4.47E-5 | 6.24E-3 | 3.20 (17680,481,184,16) | Igfbp7 - insulin-like growth factor binding protein 7  Lgals3 - lectin, galactose binding, soluble 3  Ctsb - cathepsin b Trf - transferrin  Atrnl1 - attractin like 1  Fgl2 - fibrinogen-like protein 2  Il1rl1 - interleukin 1 receptor-like 1  Mmp8 - matrix metallopeptidase 8  Tgm2 - transglutaminase 2, c polypeptide  Mmp2 - matrix metallopeptidase 2  Mmp13 - matrix metallopeptidase 13  Mmp12 - matrix metallopeptidase 12  Runx1 - runt related transcription factor 1  Col22a1 - collagen, type xxii, alpha 1  Prg2 - proteoglycan 2, bone marrow  Mfge8 - milk fat globule-egf factor 8 protein |
| GO:0005886 | plasma membrane | 6.47E-5 | 8.43E-3 | 1.57 (17680,3852,184,63) | Dsc2 - desmocollin 2  Zmat3 - zinc finger matrin type 3  Trf - transferrin  Mtmr9 - myotubularin related protein 9  Slc22a3 - solute carrier family 22 (organic cation transporter), member 3  Fpr1 - formyl peptide receptor 1  Akap11 - a kinase (prka) anchor protein 11  Fpr2 - formyl peptide receptor 2  Cd74 - cd74 antigen (invariant polypeptide of major histocompatibility complex, class ii antigen-associated)  Prkcb - protein kinase c, beta  Cd9 - cd9 antigen  Mmp2 - matrix metallopeptidase 2  Alcam - activated leukocyte cell adhesion molecule  Dab2 - disabled 2, mitogen-responsive phosphoprotein  H2-M3 - histocompatibility 2, m region locus 3  Emb - embigin  P2rx4 - purinergic receptor p2x, ligand-gated ion channel 4  Gpnmb - glycoprotein (transmembrane) nmb  Neto2 - neuropilin (nrp) and tolloid (tll)-like 2  Cp - ceruloplasmin  Clec4n - c-type lectin domain family 4, member n  Alox15 - arachidonate 15-lipoxygenase  Slc11a1 - solute carrier family 11 (proton-coupled divalent metal ion transporters), member 1  Mpp1 - membrane protein, palmitoylated  Cd34 - cd34 antigen  Mcts1 - malignant t cell amplified sequence 1  P2ry14 - purinergic receptor p2y, g-protein coupled, 14  Acvr1 - activin a receptor, type 1  Ccrl2 - chemokine (c-c motif) receptor-like 2  Stxbp6 - syntaxin binding protein 6 (amisyn)  Parvb - parvin, beta  Lpxn - leupaxin  Mrc1 - mannose receptor, c type 1  AF251705 - cdna sequence af251705  Ctsb - cathepsin b  Icam1 - intercellular adhesion molecule 1  Acsl4 - acyl-coa synthetase long-chain family member 4  Rnf157 - ring finger protein 157  Siglec5 - sialic acid binding ig-like lectin 5  Trem2 - triggering receptor expressed on myeloid cells 2  Fgr - gardner-rasheed feline sarcoma viral (fgr) oncogene homolog  Cd274 - cd274 antigen  Ppap2c - phosphatidic acid phosphatase type 2c  Emr4 - egf-like module containing, mucin-like, hormone receptor-like sequence 4  Il1rl1 - interleukin 1 receptor-like 1  Hck - hemopoietic cell kinase  Csf1r - colony stimulating factor 1 receptor  Csf2ra - colony stimulating factor 2 receptor, alpha, low-affinity (granulocyte-macrophage)  Csf1 - colony stimulating factor 1 (macrophage)  Ccr5 - chemokine (c-c motif) receptor 5  H2-D1 - histocompatibility 2, d region locus 1  Il7r - interleukin 7 receptor  H2-Ab1 - histocompatibility 2, class ii antigen a, beta 1  H2-Aa - histocompatibility 2, class ii antigen a, alpha  Casp1 - caspase 1  Sirpa - signal-regulatory protein alpha  Cpne7 - copine vii  Atrnl1 - attractin like 1  Thy1 - thymus cell antigen 1, theta  H2-K1 - histocompatibility 2, k1, k region  Tgm2 - transglutaminase 2, c polypeptide  Il3ra - interleukin 3 receptor, alpha chain  Lpcat2 - lysophosphatidylcholine acyltransferase 2 |
| GO:0098797 | plasma membrane protein complex | 1.04E-4 | 1.27E-2 | 2.97 (17680,517,184,16) | Itgax - integrin alpha x  Pde4b - phosphodiesterase 4b, camp specific  H2-D1 - histocompatibility 2, d region locus 1  H2-Ab1 - histocompatibility 2, class ii antigen a, beta 1  H2-Aa - histocompatibility 2, class ii antigen a, alpha  Trf - transferrin  Atp6v0d2 - atpase, h+ transporting, lysosomal v0 subunit d2  H2-K1 - histocompatibility 2, k1, k region  H2-Eb1 - histocompatibility 2, class ii antigen e beta  Cd74 - cd74 antigen (invariant polypeptide of major histocompatibility complex, class ii antigen-associated)  H2-Oa - histocompatibility 2, o region alpha locus  Acvr1 - activin a receptor, type 1  Alcam - activated leukocyte cell adhesion molecule  Dab2 - disabled 2, mitogen-responsive phosphoprotein  H2-M3 - histocompatibility 2, m region locus 3  H2-DMa - histocompatibility 2, class ii, locus dma |
| GO:1990682 | CSF1-CSF1R complex | 1.08E-4 | 1.24E-2 | 96.09 (17680,2,184,2) | Csf1r - colony stimulating factor 1 receptor  Csf1 - colony stimulating factor 1 (macrophage) |
| GO:0031224 | intrinsic component of membrane | 1.36E-4 | 1.48E-2 | 1.50 (17680,4282,184,67) | Dsc2 - desmocollin 2  Slc22a3 - solute carrier family 22 (organic cation transporter), member 3  Fpr1 - formyl peptide receptor 1  BC023829 - cdna sequence bc023829  Fpr2 - formyl peptide receptor 2  Cd74 - cd74 antigen (invariant polypeptide of major histocompatibility complex, class ii antigen-associated)  Cd9 - cd9 antigen  Alcam - activated leukocyte cell adhesion molecule  Emb - embigin  H2-DMa - histocompatibility 2, class ii, locus dma  P2rx4 - purinergic receptor p2x, ligand-gated ion channel 4  Gpnmb - glycoprotein (transmembrane) nmb  Slc2a6 - solute carrier family 2 (facilitated glucose transporter), member 6  Neto2 - neuropilin (nrp) and tolloid (tll)-like 2  Itgax - integrin alpha x  Cp - ceruloplasmin  Tnfrsf1b - tumor necrosis factor receptor superfamily, member 1b  Clec4n - c-type lectin domain family 4, member n  Tnfrsf9 - tumor necrosis factor receptor superfamily, member 9  Ms4a6d - membrane-spanning 4-domains, subfamily a, member 6d  Slc11a1 - solute carrier family 11 (proton-coupled divalent metal ion transporters), member 1  Cd34 - cd34 antigen  Plxnc1 - plexin c1  P2ry14 - purinergic receptor p2y, g-protein coupled, 14  Acvr1 - activin a receptor, type 1  Ccrl2 - chemokine (c-c motif) receptor-like 2  Stxbp6 - syntaxin binding protein 6 (amisyn)  Pld4 - phospholipase d family, member 4  Pilra - paired immunoglobin-like type 2 receptor alpha  Cyp4f18 - cytochrome p450, family 4, subfamily f, polypeptide 18  Chst11 - carbohydrate sulfotransferase 11  Mrc1 - mannose receptor, c type 1  Rnf128 - ring finger protein 128  AF251705 - cdna sequence af251705  Icam1 - intercellular adhesion molecule 1  Acsl4 - acyl-coa synthetase long-chain family member 4  Slc6a12 - solute carrier family 6 (neurotransmitter transporter, betaine/gaba), member 12  Siglec5 - sialic acid binding ig-like lectin 5  Trem2 - triggering receptor expressed on myeloid cells 2  Cd274 - cd274 antigen  Ppap2c - phosphatidic acid phosphatase type 2c  Emr4 - egf-like module containing, mucin-like, hormone receptor-like sequence 4  Il1rl1 - interleukin 1 receptor-like 1  Slc15a3 - solute carrier family 15, member 3  Csf1r - colony stimulating factor 1 receptor  Csf2ra - colony stimulating factor 2 receptor, alpha, low-affinity (granulocyte-macrophage)  Ms4a6c - membrane-spanning 4-domains, subfamily a, member 6c  Degs1 - degenerative spermatocyte homolog 1 (drosophila)  Csf1 - colony stimulating factor 1 (macrophage)  Procr - protein c receptor, endothelial  Cd200r3 - cd200 receptor 3  Ccr5 - chemokine (c-c motif) receptor 5  H2-D1 - histocompatibility 2, d region locus 1  Il7r - interleukin 7 receptor  Ms4a4b - membrane-spanning 4-domains, subfamily a, member 4b  H2-Ab1 - histocompatibility 2, class ii antigen a, beta 1  H2-Aa - histocompatibility 2, class ii antigen a, alpha  Sirpa - signal-regulatory protein alpha  Atrnl1 - attractin like 1  Thy1 - thymus cell antigen 1, theta  H2-K1 - histocompatibility 2, k1, k region  H2-Eb1 - histocompatibility 2, class ii antigen e beta  Tgm2 - transglutaminase 2, c polypeptide  Slc35d1 - solute carrier family 35 (udp-glucuronic acid/udp-n-acetylgalactosamine dual transporter), member d1  Il3ra - interleukin 3 receptor, alpha chain  Pld3 - phospholipase d family, member 3  Lpcat2 - lysophosphatidylcholine acyltransferase 2 |
| GO:0016020 | membrane | 2.13E-4 | 2.19E-2 | 1.33 (17680,7105,184,98) | Dsc2 - desmocollin 2  Slc22a3 - solute carrier family 22 (organic cation transporter), member 3  BC023829 - cdna sequence bc023829  Akap11 - a kinase (prka) anchor protein 11  Prkcb - protein kinase c, beta  Snx10 - sorting nexin 10  Dab2 - disabled 2, mitogen-responsive phosphoprotein  H2-M3 - histocompatibility 2, m region locus 3  Gch1 - gtp cyclohydrolase 1  H2-DMa - histocompatibility 2, class ii, locus dma  Slc2a6 - solute carrier family 2 (facilitated glucose transporter), member 6  Neto2 - neuropilin (nrp) and tolloid (tll)-like 2  Pde4b - phosphodiesterase 4b, camp specific  Slc11a1 - solute carrier family 11 (proton-coupled divalent metal ion transporters), member 1  Plxnc1 - plexin c1  Mcts1 - malignant t cell amplified sequence 1  Acvr1 - activin a receptor, type 1  Parvb - parvin, beta  Lpxn - leupaxin  Pilra - paired immunoglobin-like type 2 receptor alpha  Cyp4f18 - cytochrome p450, family 4, subfamily f, polypeptide 18  Mrc1 - mannose receptor, c type 1  Ikbke - inhibitor of kappab kinase epsilon  Icam1 - intercellular adhesion molecule 1  Ctsb - cathepsin b  Slc6a12 - solute carrier family 6 (neurotransmitter transporter, betaine/gaba), member 12  Trem2 - triggering receptor expressed on myeloid cells 2  Cd274 - cd274 antigen  Il1rl1 - interleukin 1 receptor-like 1  Emr4 - egf-like module containing, mucin-like, hormone receptor-like sequence 4  Hck - hemopoietic cell kinase  Slc15a3 - solute carrier family 15, member 3  Ctss - cathepsin s  Ccr5 - chemokine (c-c motif) receptor 5  Casp1 - caspase 1  Sirpa - signal-regulatory protein alpha  Ifi27 - interferon, alpha-inducible protein 27  Pld3 - phospholipase d family, member 3  Lpcat2 - lysophosphatidylcholine acyltransferase 2  Mgll - monoglyceride lipase  Zmat3 - zinc finger matrin type 3  Trf - transferrin  Mtmr9 - myotubularin related protein 9  Fpr1 - formyl peptide receptor 1  Fpr2 - formyl peptide receptor 2  Cd74 - cd74 antigen (invariant polypeptide of major histocompatibility complex, class ii antigen-associated)  Mmp2 - matrix metallopeptidase 2  Cd9 - cd9 antigen  Alcam - activated leukocyte cell adhesion molecule  Emb - embigin  P2rx4 - purinergic receptor p2x, ligand-gated ion channel 4  Lgals3bp - lectin, galactoside-binding, soluble, 3 binding protein  Gpnmb - glycoprotein (transmembrane) nmb  Itgax - integrin alpha x  Cp - ceruloplasmin  Tnfrsf1b - tumor necrosis factor receptor superfamily, member 1b  Clec4n - c-type lectin domain family 4, member n  Alox15 - arachidonate 15-lipoxygenase  Tnfrsf9 - tumor necrosis factor receptor superfamily, member 9  Ms4a6d - membrane-spanning 4-domains, subfamily a, member 6d  Mpp1 - membrane protein, palmitoylated  Cd34 - cd34 antigen  P2ry14 - purinergic receptor p2y, g-protein coupled, 14  Ccrl2 - chemokine (c-c motif) receptor-like 2  Stxbp6 - syntaxin binding protein 6 (amisyn)  Nenf - neuron derived neurotrophic factor  Pld4 - phospholipase d family, member 4  Chst11 - carbohydrate sulfotransferase 11  Rnf128 - ring finger protein 128  AF251705 - cdna sequence af251705  Acsl4 - acyl-coa synthetase long-chain family member 4  Rnf157 - ring finger protein 157  Myo7a - myosin viia  Siglec5 - sialic acid binding ig-like lectin 5  Fgr - gardner-rasheed feline sarcoma viral (fgr) oncogene homolog  Ppap2c - phosphatidic acid phosphatase type 2c  Csf1r - colony stimulating factor 1 receptor  Csf2ra - colony stimulating factor 2 receptor, alpha, low-affinity (granulocyte-macrophage)  Degs1 - degenerative spermatocyte homolog 1 (drosophila)  Ms4a6c - membrane-spanning 4-domains, subfamily a, member 6c  Csf1 - colony stimulating factor 1 (macrophage)  Procr - protein c receptor, endothelial  Lgals3 - lectin, galactose binding, soluble 3  H2-D1 - histocompatibility 2, d region locus 1  Cd200r3 - cd200 receptor 3  Il7r - interleukin 7 receptor  H2-Ab1 - histocompatibility 2, class ii antigen a, beta 1  H2-Aa - histocompatibility 2, class ii antigen a, alpha  Cpne7 - copine vii  Atrnl1 - attractin like 1  Thy1 - thymus cell antigen 1, theta  Atp6v0d2 - atpase, h+ transporting, lysosomal v0 subunit d2  H2-K1 - histocompatibility 2, k1, k region  H2-Eb1 - histocompatibility 2, class ii antigen e beta  Tgm2 - transglutaminase 2, c polypeptide  Slc35d1 - solute carrier family 35 (udp-glucuronic acid/udp-n-acetylgalactosamine dual transporter), member d1  Il3ra - interleukin 3 receptor, alpha chain  Mfge8 - milk fat globule-egf factor 8 protein |
| GO:0016021 | integral component of membrane | 2.51E-4 | 2.45E-2 | 1.49 (17680,4114,184,64) | Dsc2 - desmocollin 2  Slc22a3 - solute carrier family 22 (organic cation transporter), member 3  Fpr1 - formyl peptide receptor 1  BC023829 - cdna sequence bc023829  Fpr2 - formyl peptide receptor 2  Cd74 - cd74 antigen (invariant polypeptide of major histocompatibility complex, class ii antigen-associated)  Cd9 - cd9 antigen  Alcam - activated leukocyte cell adhesion molecule  Emb - embigin  H2-DMa - histocompatibility 2, class ii, locus dma  P2rx4 - purinergic receptor p2x, ligand-gated ion channel 4  Gpnmb - glycoprotein (transmembrane) nmb  Slc2a6 - solute carrier family 2 (facilitated glucose transporter), member 6  Neto2 - neuropilin (nrp) and tolloid (tll)-like 2  Itgax - integrin alpha x  Tnfrsf1b - tumor necrosis factor receptor superfamily, member 1b  Clec4n - c-type lectin domain family 4, member n  Tnfrsf9 - tumor necrosis factor receptor superfamily, member 9  Ms4a6d - membrane-spanning 4-domains, subfamily a, member 6d  Slc11a1 - solute carrier family 11 (proton-coupled divalent metal ion transporters), member 1  Cd34 - cd34 antigen  Plxnc1 - plexin c1  P2ry14 - purinergic receptor p2y, g-protein coupled, 14  Acvr1 - activin a receptor, type 1  Ccrl2 - chemokine (c-c motif) receptor-like 2  Stxbp6 - syntaxin binding protein 6 (amisyn)  Pld4 - phospholipase d family, member 4  Pilra - paired immunoglobin-like type 2 receptor alpha  Cyp4f18 - cytochrome p450, family 4, subfamily f, polypeptide 18  Chst11 - carbohydrate sulfotransferase 11  Mrc1 - mannose receptor, c type 1  Rnf128 - ring finger protein 128  AF251705 - cdna sequence af251705  Icam1 - intercellular adhesion molecule 1  Acsl4 - acyl-coa synthetase long-chain family member 4  Slc6a12 - solute carrier family 6 (neurotransmitter transporter, betaine/gaba), member 12  Siglec5 - sialic acid binding ig-like lectin 5  Trem2 - triggering receptor expressed on myeloid cells 2  Cd274 - cd274 antigen  Ppap2c - phosphatidic acid phosphatase type 2c  Emr4 - egf-like module containing, mucin-like, hormone receptor-like sequence 4  Il1rl1 - interleukin 1 receptor-like 1  Slc15a3 - solute carrier family 15, member 3  Csf1r - colony stimulating factor 1 receptor  Csf2ra - colony stimulating factor 2 receptor, alpha, low-affinity (granulocyte-macrophage)  Ms4a6c - membrane-spanning 4-domains, subfamily a, member 6c  Degs1 - degenerative spermatocyte homolog 1 (drosophila)  Csf1 - colony stimulating factor 1 (macrophage)  Procr - protein c receptor, endothelial  Cd200r3 - cd200 receptor 3  Ccr5 - chemokine (c-c motif) receptor 5  H2-D1 - histocompatibility 2, d region locus 1  Il7r - interleukin 7 receptor  Ms4a4b - membrane-spanning 4-domains, subfamily a, member 4b  H2-Ab1 - histocompatibility 2, class ii antigen a, beta 1  H2-Aa - histocompatibility 2, class ii antigen a, alpha  Sirpa - signal-regulatory protein alpha  Atrnl1 - attractin like 1  H2-K1 - histocompatibility 2, k1, k region  H2-Eb1 - histocompatibility 2, class ii antigen e beta  Slc35d1 - solute carrier family 35 (udp-glucuronic acid/udp-n-acetylgalactosamine dual transporter), member d1  Il3ra - interleukin 3 receptor, alpha chain  Pld3 - phospholipase d family, member 3  Lpcat2 - lysophosphatidylcholine acyltransferase 2 |
| GO:0098852 | lytic vacuole membrane | 3.87E-4 | 3.6E-2 | 5.25 (17680,128,184,7) | Slc15a3 - solute carrier family 15, member 3  Myo7a - myosin viia  Pld3 - phospholipase d family, member 3  H2-DMa - histocompatibility 2, class ii, locus dma  Atp6v0d2 - atpase, h+ transporting, lysosomal v0 subunit d2  Slc2a6 - solute carrier family 2 (facilitated glucose transporter), member 6  H2-Eb1 - histocompatibility 2, class ii antigen e beta |
| GO:0005765 | lysosomal membrane | 3.87E-4 | 3.43E-2 | 5.25 (17680,128,184,7) | Slc15a3 - solute carrier family 15, member 3  Myo7a - myosin viia  Pld3 - phospholipase d family, member 3  H2-DMa - histocompatibility 2, class ii, locus dma  Atp6v0d2 - atpase, h+ transporting, lysosomal v0 subunit d2  Slc2a6 - solute carrier family 2 (facilitated glucose transporter), member 6  H2-Eb1 - histocompatibility 2, class ii antigen e beta |
| GO:0005770 | Late endosome | 4.29E-4 | 3.65E-2 | 4.50 (17680,171,184,8) | Cd74 - cd74 antigen (invariant polypeptide of major histocompatibility complex, class ii antigen-associated)  Rnf128 - ring finger protein 128  H2-Ab1 - histocompatibility 2, class ii antigen a, beta 1  Trf - transferrin  Cst7 - cystatin f (leukocystatin)  Slc11a1 - solute carrier family 11 (proton-coupled divalent metal ion transporters), member 1  H2-DMa - histocompatibility 2, class ii, locus dma  Ctss - cathepsin s |
| GO:0005768 | Endosome | 4.72E-4 | 3.84E-2 | 2.29 (17680,838,184,20) | Ccr5 - chemokine (c-c motif) receptor 5  Mrc1 - mannose receptor, c type 1  Rnf128 - ring finger protein 128  H2-Ab1 - histocompatibility 2, class ii antigen a, beta 1  Trf - transferrin  Rnf157 - ring finger protein 157  Cd274 - cd274 antigen  Cst7 - cystatin f (leukocystatin)  Slc11a1 - solute carrier family 11 (proton-coupled divalent metal ion transporters), member 1  Atp6v0d2 - atpase, h+ transporting, lysosomal v0 subunit d2  Ppap2c - phosphatidic acid phosphatase type 2c  Slc15a3 - solute carrier family 15, member 3  Cd74 - cd74 antigen (invariant polypeptide of major histocompatibility complex, class ii antigen-associated)  Snx10 - sorting nexin 10  H2-M3 - histocompatibility 2, m region locus 3  Pld3 - phospholipase d family, member 3  H2-DMa - histocompatibility 2, class ii, locus dma  Pld4 - phospholipase d family, member 4  Gpnmb - glycoprotein (transmembrane) nmb  Ctss - cathepsin s |
| GO:0005771 | Multivesicular body | 8.51E-4 | 6.65E-2 | 9.37 (17680,41,184,4) | Cd74 - cd74 antigen (invariant polypeptide of major histocompatibility complex, class ii antigen-associated)  H2-Ab1 - histocompatibility 2, class ii antigen a, beta 1  Cst7 - cystatin f (leukocystatin)  H2-DMa - histocompatibility 2, class ii, locus dma |

Differentially expressed RNA was ranked according to the p-values of differential expression and degree of enrichment compared with the total number of expressed genes analysed (17680 GO terms). The GOrilla database updated on Mar 6, 2021 was used.

**'P-value'** is the enrichment p-value computed according to the mHG or HG model. This p-value is not corrected for multiple testing of 1953 GO terms.

**'FDR q-value'** is the correction of the above p-value for multiple testing using the Benjamini and Hochberg (1995) method. Namely, for the ith term (ranked according to p-value) the FDR q-value is (p-value * number of GO terms) / i.

**Enrichment (N, B, n, b)** is defined as follows:

N - is the total number of genes

B - is the total number of genes associated with a specific GO term

n - is the number of genes in the top of the user's input list or in the target set when appropriate b - is the number of genes in the intersection

Enrichment = (b/n) / (B/N)

**Genes:** For each GO term you can see the list of associated genes that appear in the optimal top of the list. Each gene name is specified by gene symbol followed by a short description of the gene
