# Supplementary material for: Proteomic and transcriptomic characterisation of FIA10, a novel murine leukemic cell line that metastasizes into the brain
Source: PLoS One. 2024 Jan 12;19(1):e0295641. doi: 10.1371/journal.pone.0295641 (PMC10786371; doi:10.1371/journal.pone.0295641)
Supplement: S9 Table — (DOCX) [file pone.0295641.s014.docx]

**Gene Ontology: Biological process FIA10 vs FIA18 Protein upregulated**

| **GO term** | **Description** | **P-value** | **FDR q-value** | **Enrichment (N, B, n, b)** | **Genes** |
| --- | --- | --- | --- | --- | --- |
| GO:0048002 | antigen processing and presentation of peptide antigen | 2.76E-11 | 3.26E-7 | 12.88 (6008,29,193,12) | Cd74 - cd74 antigen (invariant polypeptide of major histocompatibility complex, class ii antigen-associated) H2-D1 - histocompatibility 2, d region locus 1 H2-Ab1 - histocompatibility 2, class ii antigen a, beta 1 H2-Aa - histocompatibility 2, class ii antigen a, alpha H2-L - histocompatibility 2, d region locus l B2m - beta-2 microglobulin H2-DMa - histocompatibility 2, class ii, locus dma H2-K1 - histocompatibility 2, k1, k region H2-DMb2 - histocompatibility 2, class ii, locus mb2 H2-Eb1 - histocompatibility 2, class ii antigen e beta H2-DMb1 - histocompatibility 2, class ii, locus mb1 Ctss - cathepsin s |
| GO:0019882 | antigen processing and presentation | 1.44E-10 | 8.47E-7 | 9.08 (6008,48,193,14) | H2-D1 - histocompatibility 2, d region locus 1 H2-Ab1 - histocompatibility 2, class ii antigen a, beta 1 H2-Aa - histocompatibility 2, class ii antigen a, alpha H2-L - histocompatibility 2, d region locus l B2m - beta-2 microglobulin H2-K1 - histocompatibility 2, k1, k region H2-Eb1 - histocompatibility 2, class ii antigen e beta Cd74 - cd74 antigen (invariant polypeptide of major histocompatibility complex, class ii antigen-associated) Psap - prosaposin H2-DMa - histocompatibility 2, class ii, locus dma Psmb9 - proteasome (prosome, macropain) subunit, beta type 9 (large multifunctional peptidase 2) H2-DMb2 - histocompatibility 2, class ii, locus mb2 H2-DMb1 - histocompatibility 2, class ii, locus mb1 Ctss - cathepsin s |
| GO:0002478 | antigen processing and presentation of exogenous peptide antigen | 7.38E-10 | 2.9E-6 | 13.53 (6008,23,193,10) | Cd74 - cd74 antigen (invariant polypeptide of major histocompatibility complex, class ii antigen-associated) H2-Ab1 - histocompatibility 2, class ii antigen a, beta 1 H2-Aa - histocompatibility 2, class ii antigen a, alpha B2m - beta-2 microglobulin H2-DMa - histocompatibility 2, class ii, locus dma H2-DMb2 - histocompatibility 2, class ii, locus mb2 H2-K1 - histocompatibility 2, k1, k region Ctss - cathepsin s H2-DMb1 - histocompatibility 2, class ii, locus mb1 H2-Eb1 - histocompatibility 2, class ii antigen e beta |
| GO:0002504 | antigen processing and presentation of peptide or polysaccharide antigen via MHC class II | 2.5E-9 | 7.36E-6 | 17.79 (6008,14,193,8) | Cd74 - cd74 antigen (invariant polypeptide of major histocompatibility complex, class ii antigen-associated) H2-Ab1 - histocompatibility 2, class ii antigen a, beta 1 H2-Aa - histocompatibility 2, class ii antigen a, alpha H2-DMa - histocompatibility 2, class ii, locus dma H2-DMb2 - histocompatibility 2, class ii, locus mb2 H2-DMb1 - histocompatibility 2, class ii, locus mb1 Ctss - cathepsin s H2-Eb1 - histocompatibility 2, class ii antigen e beta |
| GO:0002495 | antigen processing and presentation of peptide antigen via MHC class II | 2.5E-9 | 5.89E-6 | 17.79 (6008,14,193,8) | Cd74 - cd74 antigen (invariant polypeptide of major histocompatibility complex, class ii antigen-associated) H2-Ab1 - histocompatibility 2, class ii antigen a, beta 1 H2-Aa - histocompatibility 2, class ii antigen a, alpha H2-DMa - histocompatibility 2, class ii, locus dma H2-DMb2 - histocompatibility 2, class ii, locus mb2 Ctss - cathepsin s H2-DMb1 - histocompatibility 2, class ii, locus mb1 H2-Eb1 - histocompatibility 2, class ii antigen e beta |
| GO:0019886 | antigen processing and presentation of exogenous peptide antigen via MHC class II | 2.5E-9 | 4.91E-6 | 17.79 (6008,14,193,8) | Cd74 - cd74 antigen (invariant polypeptide of major histocompatibility complex, class ii antigen-associated) H2-Ab1 - histocompatibility 2, class ii antigen a, beta 1 H2-Aa - histocompatibility 2, class ii antigen a, alpha H2-DMa - histocompatibility 2, class ii, locus dma H2-DMb2 - histocompatibility 2, class ii, locus mb2 H2-DMb1 - histocompatibility 2, class ii, locus mb1 Ctss - cathepsin s H2-Eb1 - histocompatibility 2, class ii antigen e beta |
| GO:0019884 | antigen processing and presentation of exogenous antigen | 3.15E-9 | 5.3E-6 | 11.97 (6008,26,193,10) | Cd74 - cd74 antigen (invariant polypeptide of major histocompatibility complex, class ii antigen-associated) H2-Ab1 - histocompatibility 2, class ii antigen a, beta 1 H2-Aa - histocompatibility 2, class ii antigen a, alpha B2m - beta-2 microglobulin H2-DMa - histocompatibility 2, class ii, locus dma H2-K1 - histocompatibility 2, k1, k region H2-DMb2 - histocompatibility 2, class ii, locus mb2 H2-Eb1 - histocompatibility 2, class ii antigen e beta Ctss - cathepsin s H2-DMb1 - histocompatibility 2, class ii, locus mb1 |
| GO:0006955 | immune response | 7.39E-9 | 1.09E-5 | 3.32 (6008,272,193,29) | Fgr - gardner-rasheed feline sarcoma viral (fgr) oncogene homolog Ddx58 - dead (asp-glu-ala-asp) box polypeptide 58 Cd74 - cd74 antigen (invariant polypeptide of major histocompatibility complex, class ii antigen-associated) Prkcb - protein kinase c, beta Ltf - lactotransferrin Ndufs4 - nadh dehydrogenase (ubiquinone) fe-s protein 4 Cd68 - cd68 antigen H2-DMa - histocompatibility 2, class ii, locus dma Dlg1 - discs, large homolog 1 (drosophila) Ctss - cathepsin s H2-DMb1 - histocompatibility 2, class ii, locus mb1 Ear2 - eosinophil-associated, ribonuclease a family, member 2 Lgals3 - lectin, galactose binding, soluble 3 H2-D1 - histocompatibility 2, d region locus 1 Pycard - pyd and card domain containing H2-Ab1 - histocompatibility 2, class ii antigen a, beta 1 H2-Aa - histocompatibility 2, class ii antigen a, alpha H2-L - histocompatibility 2, d region locus l B2m - beta-2 microglobulin H2-K1 - histocompatibility 2, k1, k region H2-Eb1 - histocompatibility 2, class ii antigen e beta Parp9 - poly (adp-ribose) polymerase family, member 9 Notch2 - notch 2 Il1rn - interleukin 1 receptor antagonist Slc15a4 - solute carrier family 15, member 4 Ear10 - eosinophil-associated, ribonuclease a family, member 10 Pld4 - phospholipase d family, member 4 Ccr1 - chemokine (c-c motif) receptor 1 Lcn2 - lipocalin 2 |
| GO:0002376 | immune system process | 3E-8 | 3.92E-5 | 2.37 (6008,579,193,44) | Nfatc2 - nuclear factor of activated t cells, cytoplasmic, calcineurin dependent 2 Nfatc1 - nuclear factor of activated t cells, cytoplasmic, calcineurin dependent 1 Retnlg - resistin like gamma Card11 - caspase recruitment domain family, member 11 Ddx58 - dead (asp-glu-ala-asp) box polypeptide 58 Mnda - myeloid cell nuclear differentiation antigen Fgr - gardner-rasheed feline sarcoma viral (fgr) oncogene homolog Cd74 - cd74 antigen (invariant polypeptide of major histocompatibility complex, class ii antigen-associated) Prkcb - protein kinase c, beta Ltf - lactotransferrin Ndufs4 - nadh dehydrogenase (ubiquinone) fe-s protein 4 Cd68 - cd68 antigen H2-DMa - histocompatibility 2, class ii, locus dma Dlg1 - discs, large homolog 1 (drosophila) H2-DMb2 - histocompatibility 2, class ii, locus mb2 Ctss - cathepsin s H2-DMb1 - histocompatibility 2, class ii, locus mb1 Ear2 - eosinophil-associated, ribonuclease a family, member 2 H2-D1 - histocompatibility 2, d region locus 1 Lgals3 - lectin, galactose binding, soluble 3 Pycard - pyd and card domain containing H2-Ab1 - histocompatibility 2, class ii antigen a, beta 1 C5ar1 - complement component 5a receptor 1 H2-Aa - histocompatibility 2, class ii antigen a, alpha Ercc1 - excision repair cross-complementing rodent repair deficiency, complementation group 1 Inppl1 - inositol polyphosphate phosphatase-like 1 H2-L - histocompatibility 2, d region locus l B2m - beta-2 microglobulin H2-K1 - histocompatibility 2, k1, k region H2-Eb1 - histocompatibility 2, class ii antigen e beta Ifi205 - interferon activated gene 205 Psap - prosaposin Notch2 - notch 2 Parp9 - poly (adp-ribose) polymerase family, member 9 Il1rn - interleukin 1 receptor antagonist Slc15a4 - solute carrier family 15, member 4 Mndal - myeloid nuclear differentiation antigen like Ear10 - eosinophil-associated, ribonuclease a family, member 10 Pld4 - phospholipase d family, member 4 Stat2 - signal transducer and activator of transcription 2 Ccr1 - chemokine (c-c motif) receptor 1 Psmb9 - proteasome (prosome, macropain) subunit, beta type 9 (large multifunctional peptidase 2) Lcn2 - lipocalin 2 Psmb10 - proteasome (prosome, macropain) subunit, beta type 10 |
| GO:0006342 | chromatin silencing | 3.49E-6 | 4.11E-3 | 7.00 (6008,40,193,9) | Hist1h2af - histone cluster 1, h2af Hist1h2ab - histone cluster 1, h2ab Hist2h2ac - histone cluster 2, h2ac H2afj - h2a histone family, member j Hist3h2a - histone cluster 3, h2a Hist1h2aa - histone cluster 1, h2aa Hist1h2ad - histone cluster 1, h2ad Hist1h2ak - histone cluster 1, h2ak Hist1h2ah - histone cluster 1, h2ah |
| GO:0050778 | positive regulation of immune response | 1.18E-5 | 1.26E-2 | 2.85 (6008,229,193,21) | Nfatc2 - nuclear factor of activated t cells, cytoplasmic, calcineurin dependent 2 H2-D1 - histocompatibility 2, d region locus 1 Pycard - pyd and card domain containing H2-Ab1 - histocompatibility 2, class ii antigen a, beta 1 C5ar1 - complement component 5a receptor 1 Card11 - caspase recruitment domain family, member 11 B2m - beta-2 microglobulin H2-L - histocompatibility 2, d region locus l Mnda - myeloid cell nuclear differentiation antigen Ddx58 - dead (asp-glu-ala-asp) box polypeptide 58 Fgr - gardner-rasheed feline sarcoma viral (fgr) oncogene homolog H2-K1 - histocompatibility 2, k1, k region Prkcb - protein kinase c, beta Cd74 - cd74 antigen (invariant polypeptide of major histocompatibility complex, class ii antigen-associated) Ifi205 - interferon activated gene 205 Parp9 - poly (adp-ribose) polymerase family, member 9 Wdfy1 - wd repeat and fyve domain containing 1 Slc15a4 - solute carrier family 15, member 4 Mndal - myeloid nuclear differentiation antigen like Ltf - lactotransferrin H2-DMa - histocompatibility 2, class ii, locus dma |
| GO:0045814 | negative regulation of gene expression, epigenetic | 1.43E-5 | 1.4E-2 | 5.96 (6008,47,193,9) | Hist1h2af - histone cluster 1, h2af Hist1h2ab - histone cluster 1, h2ab Hist2h2ac - histone cluster 2, h2ac H2afj - h2a histone family, member j Hist3h2a - histone cluster 3, h2a Hist1h2aa - histone cluster 1, h2aa Hist1h2ad - histone cluster 1, h2ad Hist1h2ak - histone cluster 1, h2ak Hist1h2ah - histone cluster 1, h2ah |
| GO:0006952 | defense response | 1.79E-5 | 1.62E-2 | 2.39 (6008,352,193,27) | Fgr - gardner-rasheed feline sarcoma viral (fgr) oncogene homolog Ddx58 - dead (asp-glu-ala-asp) box polypeptide 58 Cd74 - cd74 antigen (invariant polypeptide of major histocompatibility complex, class ii antigen-associated) Ltf - lactotransferrin Cers6 - ceramide synthase 6 Cd68 - cd68 antigen Ndufs4 - nadh dehydrogenase (ubiquinone) fe-s protein 4 Ear2 - eosinophil-associated, ribonuclease a family, member 2 Lgals3 - lectin, galactose binding, soluble 3 Ngp - neutrophilic granule protein Pycard - pyd and card domain containing Lsp1 - lymphocyte specific 1 C5ar1 - complement component 5a receptor 1 H2-L - histocompatibility 2, d region locus l B2m - beta-2 microglobulin H2-K1 - histocompatibility 2, k1, k region Parp9 - poly (adp-ribose) polymerase family, member 9 Notch2 - notch 2 Il1rn - interleukin 1 receptor antagonist Itih4 - inter alpha-trypsin inhibitor, heavy chain 4 Slc15a4 - solute carrier family 15, member 4 Chi3l3 - chitinase 3-like 3 Ear10 - eosinophil-associated, ribonuclease a family, member 10 Pld4 - phospholipase d family, member 4 Stat2 - signal transducer and activator of transcription 2 Lcn2 - lipocalin 2 Ccr1 - chemokine (c-c motif) receptor 1 |
| GO:0009617 | response to bacterium | 2.99E-5 | 2.51E-2 | 3.82 (6008,106,193,13) | Pycard - pyd and card domain containing C5ar1 - complement component 5a receptor 1 B2m - beta-2 microglobulin Mnda - myeloid cell nuclear differentiation antigen Fgr - gardner-rasheed feline sarcoma viral (fgr) oncogene homolog H2-K1 - histocompatibility 2, k1, k region Ifi205 - interferon activated gene 205 Notch2 - notch 2 Pygl - liver glycogen phosphorylase Mndal - myeloid nuclear differentiation antigen like Ltf - lactotransferrin Psmb9 - proteasome (prosome, macropain) subunit, beta type 9 (large multifunctional peptidase 2) Lcn2 - lipocalin 2 |
| GO:0002684 | positive regulation of immune system process | 3.27E-5 | 2.57E-2 | 2.31 (6008,364,193,27) | Nfatc2 - nuclear factor of activated t cells, cytoplasmic, calcineurin dependent 2 Card11 - caspase recruitment domain family, member 11 Fgr - gardner-rasheed feline sarcoma viral (fgr) oncogene homolog Ddx58 - dead (asp-glu-ala-asp) box polypeptide 58 Mnda - myeloid cell nuclear differentiation antigen Cd74 - cd74 antigen (invariant polypeptide of major histocompatibility complex, class ii antigen-associated) Prkcb - protein kinase c, beta Ltf - lactotransferrin H2-DMa - histocompatibility 2, class ii, locus dma H2-D1 - histocompatibility 2, d region locus 1 Lgals3 - lectin, galactose binding, soluble 3 Pycard - pyd and card domain containing H2-Ab1 - histocompatibility 2, class ii antigen a, beta 1 C5ar1 - complement component 5a receptor 1 H2-Aa - histocompatibility 2, class ii antigen a, alpha H2-L - histocompatibility 2, d region locus l B2m - beta-2 microglobulin H2-K1 - histocompatibility 2, k1, k region Mmp8 - matrix metallopeptidase 8 Ifi205 - interferon activated gene 205 Parp9 - poly (adp-ribose) polymerase family, member 9 Notch2 - notch 2 Wdfy1 - wd repeat and fyve domain containing 1 Slc15a4 - solute carrier family 15, member 4 Mndal - myeloid nuclear differentiation antigen like Zmiz1 - zinc finger, miz-type containing 1 Ccr1 - chemokine (c-c motif) receptor 1 |
| GO:0002577 | regulation of antigen processing and presentation | 6.53E-5 | 4.81E-2 | 15.56 (6008,8,193,4) | Cd74 - cd74 antigen (invariant polypeptide of major histocompatibility complex, class ii antigen-associated) Pycard - pyd and card domain containing H2-Ab1 - histocompatibility 2, class ii antigen a, beta 1 Cd68 - cd68 antigen |
| GO:0002428 | antigen processing and presentation of peptide antigen via MHC class Ib | 6.53E-5 | 4.52E-2 | 15.56 (6008,8,193,4) | H2-D1 - histocompatibility 2, d region locus 1 H2-L - histocompatibility 2, d region locus l B2m - beta-2 microglobulin H2-K1 - histocompatibility 2, k1, k region |
| GO:0050776 | regulation of immune response | 6.58E-5 | 4.3E-2 | 2.31 (6008,337,193,25) | Nfatc2 - nuclear factor of activated t cells, cytoplasmic, calcineurin dependent 2 Card11 - caspase recruitment domain family, member 11 Fgr - gardner-rasheed feline sarcoma viral (fgr) oncogene homolog Ddx58 - dead (asp-glu-ala-asp) box polypeptide 58 Mnda - myeloid cell nuclear differentiation antigen Cd74 - cd74 antigen (invariant polypeptide of major histocompatibility complex, class ii antigen-associated) Prkcb - protein kinase c, beta Ptprs - protein tyrosine phosphatase, receptor type, s Ltf - lactotransferrin H2-DMa - histocompatibility 2, class ii, locus dma H2-D1 - histocompatibility 2, d region locus 1 Lgals3 - lectin, galactose binding, soluble 3 Pycard - pyd and card domain containing H2-Ab1 - histocompatibility 2, class ii antigen a, beta 1 C5ar1 - complement component 5a receptor 1 H2-L - histocompatibility 2, d region locus l B2m - beta-2 microglobulin H2-K1 - histocompatibility 2, k1, k region Ifi205 - interferon activated gene 205 Parp9 - poly (adp-ribose) polymerase family, member 9 Wdfy1 - wd repeat and fyve domain containing 1 Slc15a4 - solute carrier family 15, member 4 Mndal - myeloid nuclear differentiation antigen like Angpt1 - angiopoietin 1 Ccr1 - chemokine (c-c motif) receptor 1 |
| GO:0051239 | regulation of multicellular organismal process | 6.78E-5 | 4.2E-2 | 1.65 (6008,1018,193,54) | Nfatc2 - nuclear factor of activated t cells, cytoplasmic, calcineurin dependent 2 Aatk - apoptosis-associated tyrosine kinase Nfatc1 - nuclear factor of activated t cells, cytoplasmic, calcineurin dependent 1 Ddx58 - dead (asp-glu-ala-asp) box polypeptide 58 Mnda - myeloid cell nuclear differentiation antigen Decr1 - 2,4-dienoyl coa reductase 1, mitochondrial Cd74 - cd74 antigen (invariant polypeptide of major histocompatibility complex, class ii antigen-associated) Prkcb - protein kinase c, beta Jup - junction plakoglobin Alb - albumin Sgpp1 - sphingosine-1-phosphate phosphatase 1 H2-DMa - histocompatibility 2, class ii, locus dma Pycard - pyd and card domain containing Nedd4l - neural precursor cell expressed, developmentally down-regulated gene 4-like C5ar1 - complement component 5a receptor 1 Fabp5 - fatty acid binding protein 5, epidermal Mmp8 - matrix metallopeptidase 8 Gng5 - guanine nucleotide binding protein (g protein), gamma 5 Oma1 - oma1 homolog, zinc metallopeptidase (s. cerevisiae) Ifi205 - interferon activated gene 205 Notch2 - notch 2 Ptpn23 - protein tyrosine phosphatase, non-receptor type 23 Pld4 - phospholipase d family, member 4 Sh3glb1 - sh3-domain grb2-like b1 (endophilin) Anxa6 - annexin a6 Card11 - caspase recruitment domain family, member 11 Syne1 - spectrin repeat containing, nuclear envelope 1 Fgr - gardner-rasheed feline sarcoma viral (fgr) oncogene homolog Dsp - desmoplakin Ehmt1 - euchromatic histone methyltransferase 1 Ptprs - protein tyrosine phosphatase, receptor type, s Tmem106b - transmembrane protein 106b Ltf - lactotransferrin Dlg1 - discs, large homolog 1 (drosophila) Ctss - cathepsin s Hip1r - huntingtin interacting protein 1 related H2-D1 - histocompatibility 2, d region locus 1 Lgals3 - lectin, galactose binding, soluble 3 Ngp - neutrophilic granule protein Ndrg2 - n-myc downstream regulated gene 2 H2-Aa - histocompatibility 2, class ii antigen a, alpha Inppl1 - inositol polyphosphate phosphatase-like 1 B2m - beta-2 microglobulin H2-K1 - histocompatibility 2, k1, k region Ap2a2 - adaptor-related protein complex 2, alpha 2 subunit Il1r2 - interleukin 1 receptor, type ii Il1rn - interleukin 1 receptor antagonist Slc15a4 - solute carrier family 15, member 4 Setx - senataxin Mndal - myeloid nuclear differentiation antigen like Angpt1 - angiopoietin 1 Zmiz1 - zinc finger, miz-type containing 1 Ccr1 - chemokine (c-c motif) receptor 1 Lcn2 - lipocalin 2 |
| GO:0002579 | positive regulation of antigen processing and presentation | 1.28E-4 | 7.51E-2 | 23.35 (6008,4,193,3) | Cd74 - cd74 antigen (invariant polypeptide of major histocompatibility complex, class ii antigen-associated) Pycard - pyd and card domain containing H2-Ab1 - histocompatibility 2, class ii antigen a, beta 1 |
| GO:0002475 | antigen processing and presentation via MHC class Ib | 1.86E-4 | 1.04E-1 | 12.45 (6008,10,193,4) | H2-D1 - histocompatibility 2, d region locus 1 B2m - beta-2 microglobulin H2-L - histocompatibility 2, d region locus l H2-K1 - histocompatibility 2, k1, k region |
| GO:0002821 | positive regulation of adaptive immune response | 1.91E-4 | 1.02E-1 | 4.88 (6008,51,193,8) | Cd74 - cd74 antigen (invariant polypeptide of major histocompatibility complex, class ii antigen-associated) H2-D1 - histocompatibility 2, d region locus 1 Pycard - pyd and card domain containing H2-Ab1 - histocompatibility 2, class ii antigen a, beta 1 B2m - beta-2 microglobulin H2-L - histocompatibility 2, d region locus l H2-DMa - histocompatibility 2, class ii, locus dma H2-K1 - histocompatibility 2, k1, k region |
| GO:0002682 | regulation of immune system process | 2.54E-4 | 1.3E-1 | 1.88 (6008,547,193,33) | Nfatc2 - nuclear factor of activated t cells, cytoplasmic, calcineurin dependent 2 Card11 - caspase recruitment domain family, member 11 Ddx58 - dead (asp-glu-ala-asp) box polypeptide 58 Mnda - myeloid cell nuclear differentiation antigen Fgr - gardner-rasheed feline sarcoma viral (fgr) oncogene homolog Cd74 - cd74 antigen (invariant polypeptide of major histocompatibility complex, class ii antigen-associated) Prkcb - protein kinase c, beta Ptprs - protein tyrosine phosphatase, receptor type, s Ltf - lactotransferrin Cd68 - cd68 antigen H2-DMa - histocompatibility 2, class ii, locus dma Dlg1 - discs, large homolog 1 (drosophila) Lgals3 - lectin, galactose binding, soluble 3 H2-D1 - histocompatibility 2, d region locus 1 Pycard - pyd and card domain containing H2-Ab1 - histocompatibility 2, class ii antigen a, beta 1 H2-Aa - histocompatibility 2, class ii antigen a, alpha C5ar1 - complement component 5a receptor 1 H2-L - histocompatibility 2, d region locus l B2m - beta-2 microglobulin H2-K1 - histocompatibility 2, k1, k region Mmp8 - matrix metallopeptidase 8 Ap2a2 - adaptor-related protein complex 2, alpha 2 subunit Ifi205 - interferon activated gene 205 Mpp1 - membrane protein, palmitoylated Parp9 - poly (adp-ribose) polymerase family, member 9 Notch2 - notch 2 Wdfy1 - wd repeat and fyve domain containing 1 Slc15a4 - solute carrier family 15, member 4 Mndal - myeloid nuclear differentiation antigen like Angpt1 - angiopoietin 1 Zmiz1 - zinc finger, miz-type containing 1 Ccr1 - chemokine (c-c motif) receptor 1 |
| GO:0002227 | innate immune response in mucosa | 3.11E-4 | 1.53E-1 | 18.68 (6008,5,193,3) | Ear2 - eosinophil-associated, ribonuclease a family, member 2 Ltf - lactotransferrin Ear10 - eosinophil-associated, ribonuclease a family, member 10 |
| GO:0001778 | plasma membrane repair | 3.11E-4 | 1.47E-1 | 18.68 (6008,5,193,3) | Myof - myoferlin Anxa6 - annexin a6 Myh10 - myosin, heavy polypeptide 10, non-muscle |
| GO:0098911 | regulation of ventricular cardiac muscle cell action potential | 3.11E-4 | 1.41E-1 | 18.68 (6008,5,193,3) | Jup - junction plakoglobin Dsp - desmoplakin Dlg1 - discs, large homolog 1 (drosophila) |
| GO:0002483 | antigen processing and presentation of endogenous peptide antigen | 4.17E-4 | 1.82E-1 | 10.38 (6008,12,193,4) | H2-D1 - histocompatibility 2, d region locus 1 H2-L - histocompatibility 2, d region locus l B2m - beta-2 microglobulin H2-K1 - histocompatibility 2, k1, k region |
| GO:0019885 | antigen processing and presentation of endogenous peptide antigen via MHC class I | 4.17E-4 | 1.75E-1 | 10.38 (6008,12,193,4) | H2-D1 - histocompatibility 2, d region locus 1 H2-L - histocompatibility 2, d region locus l B2m - beta-2 microglobulin H2-K1 - histocompatibility 2, k1, k region |
| GO:0006826 | iron ion transport | 5.88E-4 | 2.39E-1 | 9.58 (6008,13,193,4) | Cp - ceruloplasmin Ltf - lactotransferrin B2m - beta-2 microglobulin Fth1 - ferritin heavy chain 1 |
| GO:0019883 | antigen processing and presentation of endogenous antigen | 5.88E-4 | 2.31E-1 | 9.58 (6008,13,193,4) | H2-D1 - histocompatibility 2, d region locus 1 H2-L - histocompatibility 2, d region locus l B2m - beta-2 microglobulin H2-K1 - histocompatibility 2, k1, k region |
| GO:0002486 | antigen processing and presentation of endogenous peptide antigen via MHC class I via ER pathway, TAP-independent | 6.08E-4 | 2.31E-1 | 15.56 (6008,6,193,3) | H2-D1 - histocompatibility 2, d region locus 1 H2-L - histocompatibility 2, d region locus l H2-K1 - histocompatibility 2, k1, k region |
| GO:0031349 | positive regulation of defense response | 6.77E-4 | 2.49E-1 | 2.81 (6008,144,193,13) | Pycard - pyd and card domain containing Mnda - myeloid cell nuclear differentiation antigen Ddx58 - dead (asp-glu-ala-asp) box polypeptide 58 Mmp8 - matrix metallopeptidase 8 Cd74 - cd74 antigen (invariant polypeptide of major histocompatibility complex, class ii antigen-associated) Ifi205 - interferon activated gene 205 Parp9 - poly (adp-ribose) polymerase family, member 9 Tgm2 - transglutaminase 2, c polypeptide Wdfy1 - wd repeat and fyve domain containing 1 Slc15a4 - solute carrier family 15, member 4 Mndal - myeloid nuclear differentiation antigen like Ltf - lactotransferrin Ctss - cathepsin s |
| GO:0051707 | response to other organism | 6.85E-4 | 2.44E-1 | 2.39 (6008,221,193,17) | Lgals3 - lectin, galactose binding, soluble 3 Pycard - pyd and card domain containing C5ar1 - complement component 5a receptor 1 B2m - beta-2 microglobulin Fgr - gardner-rasheed feline sarcoma viral (fgr) oncogene homolog Mnda - myeloid cell nuclear differentiation antigen Ddx58 - dead (asp-glu-ala-asp) box polypeptide 58 H2-K1 - histocompatibility 2, k1, k region Ifi205 - interferon activated gene 205 Parp9 - poly (adp-ribose) polymerase family, member 9 Notch2 - notch 2 Pygl - liver glycogen phosphorylase Mndal - myeloid nuclear differentiation antigen like Ltf - lactotransferrin Stat2 - signal transducer and activator of transcription 2 Psmb9 - proteasome (prosome, macropain) subunit, beta type 9 (large multifunctional peptidase 2) Lcn2 - lipocalin 2 |
| GO:0006959 | humoral immune response | 8.39E-4 | 2.9E-1 | 6.49 (6008,24,193,5) | Notch2 - notch 2 Lgals3 - lectin, galactose binding, soluble 3 H2-Ab1 - histocompatibility 2, class ii antigen a, beta 1 Ltf - lactotransferrin B2m - beta-2 microglobulin |
| GO:0002819 | regulation of adaptive immune response | 8.9E-4 | 2.99E-1 | 3.55 (6008,79,193,9) | Cd74 - cd74 antigen (invariant polypeptide of major histocompatibility complex, class ii antigen-associated) H2-D1 - histocompatibility 2, d region locus 1 Pycard - pyd and card domain containing H2-Ab1 - histocompatibility 2, class ii antigen a, beta 1 Slc15a4 - solute carrier family 15, member 4 B2m - beta-2 microglobulin H2-L - histocompatibility 2, d region locus l H2-DMa - histocompatibility 2, class ii, locus dma H2-K1 - histocompatibility 2, k1, k region |

Differentially expressed protein was ranked according to the p-values of differential expression and degree of enrichment compared with the total number of expressed genes analysed (6008 GO terms). The GOrilla database updated on Mar 6, 2021 was used.

**'P-value'** is the enrichment p-value computed according to the mHG or HG model. This p-value is not corrected for multiple testing of 11773 GO terms.

**'FDR q-value'** is the correction of the above p-value for multiple testing using the Benjamini and Hochberg (1995) method.

Namely, for the ith term (ranked according to p-value) the FDR q-value is (p-value * number of GO terms) / i.

**Enrichment (N, B, n, b)** is defined as follows:

N - is the total number of genes

B - is the total number of genes associated with a specific GO term

n - is the number of genes in the top of the user's input list or in the target set when appropriate b - is the number of genes in the intersection

Enrichment = (b/n) / (B/N)

**Genes:** For each GO term you can see the list of associated genes that appear in the optimal top of the list. Each gene name is specified by gene symbol followed by a short description of the gene.
