# Supplementary material for: Proteomic and transcriptomic characterisation of FIA10, a novel murine leukemic cell line that metastasizes into the brain
Source: PLoS One. 2024 Jan 12;19(1):e0295641. doi: 10.1371/journal.pone.0295641 (PMC10786371; doi:10.1371/journal.pone.0295641)
Supplement: S11 Table — (DOCX) [file pone.0295641.s016.docx]

**Gene Ontology: Cellular component FIA10 vs FIA18 Protein upregulated**

| **GO term** | **Description** | **P-value** | **FDR q-value** | **Enrichment (N, B, n, b)** | **Genes** |
| --- | --- | --- | --- | --- | --- |
| GO:0042611 | MHC protein complex | 9.49E-15 | 1.57E-11 | 24.46 (6008,14,193,11) | Cd74 - cd74 antigen (invariant polypeptide of major histocompatibility complex, class ii antigen-associated) H2-D1 - histocompatibility 2, d region locus 1 H2-Ab1 - histocompatibility 2, class ii antigen a, beta 1 H2-Aa - histocompatibility 2, class ii antigen a, alpha B2m - beta-2 microglobulin H2-L - histocompatibility 2, d region locus l H2-DMa - histocompatibility 2, class ii, locus dma H2-DMb2 - histocompatibility 2, class ii, locus mb2 H2-K1 - histocompatibility 2, k1, k region H2-DMb1 - histocompatibility 2, class ii, locus mb1 H2-Eb1 - histocompatibility 2, class ii antigen e beta |
| GO:0042613 | MHC class II protein complex | 3.17E-11 | 2.63E-8 | 31.13 (6008,7,193,7) | Cd74 - cd74 antigen (invariant polypeptide of major histocompatibility complex, class ii antigen-associated) H2-Ab1 - histocompatibility 2, class ii antigen a, beta 1 H2-Aa - histocompatibility 2, class ii antigen a, alpha H2-DMa - histocompatibility 2, class ii, locus dma H2-DMb2 - histocompatibility 2, class ii, locus mb2 H2-Eb1 - histocompatibility 2, class ii antigen e beta H2-DMb1 - histocompatibility 2, class ii, locus mb1 |
| GO:0005576 | extracellular region | 2.1E-10 | 1.16E-7 | 3.98 (6008,219,193,28) | Retnlg - resistin like gamma St3gal2 - st3 beta-galactoside alpha-2,3-sialyltransferase 2 Pon2 - paraoxonase 2 Epdr1 - ependymin related protein 1 (zebrafish) Alb - albumin Ltf - lactotransferrin Retnla - resistin like alpha Fth1 - ferritin heavy chain 1 Ctss - cathepsin s Lgals3 - lectin, galactose binding, soluble 3 Ngp - neutrophilic granule protein Pycard - pyd and card domain containing Apob - apolipoprotein b Cp - ceruloplasmin Fabp5 - fatty acid binding protein 5, epidermal Asah1 - n-acylsphingosine amidohydrolase 1 B2m - beta-2 microglobulin Mmp8 - matrix metallopeptidase 8 Il1r2 - interleukin 1 receptor, type ii Psap - prosaposin Notch2 - notch 2 Itih4 - inter alpha-trypsin inhibitor, heavy chain 4 Il1rn - interleukin 1 receptor antagonist Itih1 - inter-alpha trypsin inhibitor, heavy chain 1 Scpep1 - serine carboxypeptidase 1 Chi3l3 - chitinase 3-like 3 Angpt1 - angiopoietin 1 Lcn2 - lipocalin 2 |
| GO:0044421 | extracellular region part | 6.44E-8 | 2.67E-5 | 2.81 (6008,354,193,32) | Anxa6 - annexin a6 Retnlg - resistin like gamma Serpinb10 - serine (or cysteine) peptidase inhibitor, clade b (ovalbumin), member 10 Cd74 - cd74 antigen (invariant polypeptide of major histocompatibility complex, class ii antigen-associated) Alb - albumin Ltf - lactotransferrin Dlg1 - discs, large homolog 1 (drosophila) Retnla - resistin like alpha Ctss - cathepsin s Ear2 - eosinophil-associated, ribonuclease a family, member 2 H2-D1 - histocompatibility 2, d region locus 1 Lgals3 - lectin, galactose binding, soluble 3 Ngp - neutrophilic granule protein Apob - apolipoprotein b Cp - ceruloplasmin S100a11 - s100 calcium binding protein a11 (calgizzarin) Fabp5 - fatty acid binding protein 5, epidermal Asah1 - n-acylsphingosine amidohydrolase 1 H2-L - histocompatibility 2, d region locus l B2m - beta-2 microglobulin Mmp8 - matrix metallopeptidase 8 H2-K1 - histocompatibility 2, k1, k region Psap - prosaposin Notch2 - notch 2 Tgm2 - transglutaminase 2, c polypeptide Itih4 - inter alpha-trypsin inhibitor, heavy chain 4 Il1rn - interleukin 1 receptor antagonist Itih1 - inter-alpha trypsin inhibitor, heavy chain 1 Setx - senataxin Ear10 - eosinophil-associated, ribonuclease a family, member 10 Angpt1 - angiopoietin 1 Lcn2 - lipocalin 2 |
| GO:00005615 | extracellular space | 1.42E-7 | 4.7E-5 | 3.24 (6008,240,193,25) | Retnlg - resistin like gamma Serpinb10 - serine (or cysteine) peptidase inhibitor, clade b (ovalbumin), member 10 Cd74 - cd74 antigen (invariant polypeptide of major histocompatibility complex, class ii antigen-associated) Alb - albumin Ltf - lactotransferrin Retnla - resistin like alpha Ctss - cathepsin s Ear2 - eosinophil-associated, ribonuclease a family, member 2 Lgals3 - lectin, galactose binding, soluble 3 H2-D1 - histocompatibility 2, d region locus 1 Ngp - neutrophilic granule protein Apob - apolipoprotein b Cp - ceruloplasmin S100a11 - s100 calcium binding protein a11 (calgizzarin) Fabp5 - fatty acid binding protein 5, epidermal Asah1 - n-acylsphingosine amidohydrolase 1 H2-L - histocompatibility 2, d region locus l B2m - beta-2 microglobulin H2-K1 - histocompatibility 2, k1, k region Mmp8 - matrix metallopeptidase 8 Psap - prosaposin Il1rn - interleukin 1 receptor antagonist Ear10 - eosinophil-associated, ribonuclease a family, member 10 Angpt1 - angiopoietin 1 Lcn2 - lipocalin 2 |
| GO:0098797 | plasma membrane protein complex | 2.95E-7 | 8.14E-5 | 4.09 (6008,137,193,18) | H2-D1 - histocompatibility 2, d region locus 1 H2-Ab1 - histocompatibility 2, class ii antigen a, beta 1 Lin7c - lin-7 homolog c (c. elegans) H2-Aa - histocompatibility 2, class ii antigen a, alpha Stxbp5 - syntaxin binding protein 5 (tomosyn) H2-L - histocompatibility 2, d region locus l B2m - beta-2 microglobulin Atp6v0d2 - atpase, h+ transporting, lysosomal v0 subunit d2 H2-K1 - histocompatibility 2, k1, k region H2-Eb1 - histocompatibility 2, class ii antigen e beta Ap2a2 - adaptor-related protein complex 2, alpha 2 subunit Gng5 - guanine nucleotide binding protein (g protein), gamma 5 Cd74 - cd74 antigen (invariant polypeptide of major histocompatibility complex, class ii antigen-associated) Jup - junction plakoglobin H2-DMa - histocompatibility 2, class ii, locus dma Dlg1 - discs, large homolog 1 (drosophila) H2-DMb2 - histocompatibility 2, class ii, locus mb2 H2-DMb1 - histocompatibility 2, class ii, locus mb1 |
| GO:0044437 | vacuolar part | 6.95E-7 | 1.64E-4 | 4.58 (6008,102,193,15) | Slc29a3 - solute carrier family 29 (nucleoside transporters), member 3 Anxa6 - annexin a6 Atp6v0d2 - atpase, h+ transporting, lysosomal v0 subunit d2 H2-Eb1 - histocompatibility 2, class ii antigen e beta Tmem106b - transmembrane protein 106b Slc15a4 - solute carrier family 15, member 4 Loh12cr1 - loss of heterozygosity, 12, chromosomal region 1 homolog (human) Tecpr1 - tectonin beta-propeller repeat containing 1 Atp6v0c - atpase, h+ transporting, lysosomal v0 subunit c H2-DMa - histocompatibility 2, class ii, locus dma Cd68 - cd68 antigen H2-DMb2 - histocompatibility 2, class ii, locus mb2 H2-DMb1 - histocompatibility 2, class ii, locus mb1 Vps39 - vacuolar protein sorting 39 (yeast) Sh3glb1 - sh3-domain grb2-like b1 (endophilin) |
| GO:0044459 | plasma membrane part | 1.18E-6 | 2.44E-4 | 2.19 (6008,569,193,40) | Anxa6 - annexin a6 Lin7c - lin-7 homolog c (c. elegans) Stxbp5 - syntaxin binding protein 5 (tomosyn) Syne1 - spectrin repeat containing, nuclear envelope 1 Card11 - caspase recruitment domain family, member 11 Gm2a - gm2 ganglioside activator protein Ddx58 - dead (asp-glu-ala-asp) box polypeptide 58 Fgr - gardner-rasheed feline sarcoma viral (fgr) oncogene homolog Myh10 - myosin, heavy polypeptide 10, non-muscle Prkcb - protein kinase c, beta Cd74 - cd74 antigen (invariant polypeptide of major histocompatibility complex, class ii antigen-associated) Jup - junction plakoglobin Dsp - desmoplakin Ptprs - protein tyrosine phosphatase, receptor type, s Emb - embigin H2-DMa - histocompatibility 2, class ii, locus dma Dlg1 - discs, large homolog 1 (drosophila) H2-DMb2 - histocompatibility 2, class ii, locus mb2 C2cd5 - c2 calcium-dependent domain containing 5 H2-DMb1 - histocompatibility 2, class ii, locus mb1 Hip1r - huntingtin interacting protein 1 related Lgals3 - lectin, galactose binding, soluble 3 H2-D1 - histocompatibility 2, d region locus 1 Cp - ceruloplasmin H2-Ab1 - histocompatibility 2, class ii antigen a, beta 1 H2-Aa - histocompatibility 2, class ii antigen a, alpha C5ar1 - complement component 5a receptor 1 H2-L - histocompatibility 2, d region locus l B2m - beta-2 microglobulin Pkn2 - protein kinase n2 Atp6v0d2 - atpase, h+ transporting, lysosomal v0 subunit d2 H2-K1 - histocompatibility 2, k1, k region Gng5 - guanine nucleotide binding protein (g protein), gamma 5 Ap2a2 - adaptor-related protein complex 2, alpha 2 subunit H2-Eb1 - histocompatibility 2, class ii antigen e beta Tgm2 - transglutaminase 2, c polypeptide Myof - myoferlin Notch2 - notch 2 Prkar2a - protein kinase, camp dependent regulatory, type ii alpha Ccr1 - chemokine (c-c motif) receptor 1 |
| GO:0098852 | lytic vacuole membrane | 1.52E-6 | 2.79E-4 | 5.41 (6008,69,193,12) | Slc29a3 - solute carrier family 29 (nucleoside transporters), member 3 Anxa6 - annexin a6 Tmem106b - transmembrane protein 106b Slc15a4 - solute carrier family 15, member 4 Tecpr1 - tectonin beta-propeller repeat containing 1 Cd68 - cd68 antigen H2-DMa - histocompatibility 2, class ii, locus dma Atp6v0d2 - atpase, h+ transporting, lysosomal v0 subunit d2 H2-DMb2 - histocompatibility 2, class ii, locus mb2 H2-Eb1 - histocompatibility 2, class ii antigen e beta H2-DMb1 - histocompatibility 2, class ii, locus mb1 Vps39 - vacuolar protein sorting 39 (yeast) |
| GO:0005765 | lysosomal membrane | 1.52E-6 | 2.51E-4 | 5.41 (6008,69,193,12) | Slc29a3 - solute carrier family 29 (nucleoside transporters), member 3 Anxa6 - annexin a6 Tmem106b - transmembrane protein 106b Slc15a4 - solute carrier family 15, member 4 Tecpr1 - tectonin beta-propeller repeat containing 1 Cd68 - cd68 antigen H2-DMa - histocompatibility 2, class ii, locus dma Atp6v0d2 - atpase, h+ transporting, lysosomal v0 subunit d2 H2-DMb2 - histocompatibility 2, class ii, locus mb2 H2-Eb1 - histocompatibility 2, class ii antigen e beta Vps39 - vacuolar protein sorting 39 (yeast) H2-DMb1 - histocompatibility 2, class ii, locus mb1 |
| GO:0005774 | vacuolar membrane | 2.87E-6 | 4.32E-4 | 4.71 (6008,86,193,13) | Slc29a3 - solute carrier family 29 (nucleoside transporters), member 3 Anxa6 - annexin a6 Atp6v0d2 - atpase, h+ transporting, lysosomal v0 subunit d2 H2-Eb1 - histocompatibility 2, class ii antigen e beta Tmem106b - transmembrane protein 106b Slc15a4 - solute carrier family 15, member 4 Tecpr1 - tectonin beta-propeller repeat containing 1 Cd68 - cd68 antigen H2-DMa - histocompatibility 2, class ii, locus dma H2-DMb2 - histocompatibility 2, class ii, locus mb2 H2-DMb1 - histocompatibility 2, class ii, locus mb1 Vps39 - vacuolar protein sorting 39 (yeast) Sh3glb1 - sh3-domain grb2-like b1 (endophilin) |
| GO:0000323 | lytic vacuole | 4.36E-6 | 6.01E-4 | 3.04 (6008,215,193,21) | Slc29a3 - solute carrier family 29 (nucleoside transporters), member 3 H2-Aa - histocompatibility 2, class ii antigen a, alpha Pon2 - paraoxonase 2 Gm2a - gm2 ganglioside activator protein Asah1 - n-acylsphingosine amidohydrolase 1 Epdr1 - ependymin related protein 1 (zebrafish) Psap - prosaposin Cd74 - cd74 antigen (invariant polypeptide of major histocompatibility complex, class ii antigen-associated) Sgsh - n-sulfoglucosamine sulfohydrolase (sulfamidase) Tmem106b - transmembrane protein 106b Slc15a4 - solute carrier family 15, member 4 Loh12cr1 - loss of heterozygosity, 12, chromosomal region 1 homolog (human) Atp6v0c - atpase, h+ transporting, lysosomal v0 subunit c Tecpr1 - tectonin beta-propeller repeat containing 1 H2-DMa - histocompatibility 2, class ii, locus dma Cd68 - cd68 antigen H2-DMb2 - histocompatibility 2, class ii, locus mb2 H2-DMb1 - histocompatibility 2, class ii, locus mb1 Vps39 - vacuolar protein sorting 39 (yeast) Ctss - cathepsin s Fth1 - ferritin heavy chain 1 |
| GO:0005764 | lysosome | 4.36E-6 | 5.55E-4 | 3.04 (6008,215,193,21) | [Slc29a3 - solute carrier family 29 (nucleoside transporters), member 3 H2-Aa - histocompatibility 2, class ii antigen a, alpha Pon2 - paraoxonase 2 Gm2a - gm2 ganglioside activator protein Asah1 - n-acylsphingosine amidohydrolase 1 Epdr1 - ependymin related protein 1 (zebrafish) Psap - prosaposin Cd74 - cd74 antigen (invariant polypeptide of major histocompatibility complex, class ii antigen-associated) Sgsh - n-sulfoglucosamine sulfohydrolase (sulfamidase) Tmem106b - transmembrane protein 106b Slc15a4 - solute carrier family 15, member 4 Loh12cr1 - loss of heterozygosity, 12, chromosomal region 1 homolog (human) Atp6v0c - atpase, h+ transporting, lysosomal v0 subunit c Tecpr1 - tectonin beta-propeller repeat containing 1 H2-DMa - histocompatibility 2, class ii, locus dma Cd68 - cd68 antigen H2-DMb2 - histocompatibility 2, class ii, locus mb2 Vps39 - vacuolar protein sorting 39 (yeast) H2-DMb1 - histocompatibility 2, class ii, locus mb1 Ctss - cathepsin s Fth1 - ferritin heavy chain 1 |
| GO:0044433 | cytoplasmic vesicle part | 1.4E-5 | 1.65E-3 | 2.59 (6008,288,193,24) | H2-D1 - histocompatibility 2, d region locus 1 Anxa6 - annexin a6 Stxbp5 - syntaxin binding protein 5 (tomosyn) B2m - beta-2 microglobulin H2-L - histocompatibility 2, d region locus l H2-K1 - histocompatibility 2, k1, k region H2-Eb1 - histocompatibility 2, class ii antigen e beta Ap2a2 - adaptor-related protein complex 2, alpha 2 subunit Ptprs - protein tyrosine phosphatase, receptor type, s Aftph - aftiphilin Slc15a4 - solute carrier family 15, member 4 Loh12cr1 - loss of heterozygosity, 12, chromosomal region 1 homolog (human) Itpr2 - inositol 1,4,5-triphosphate receptor 2 Dlg1 - discs, large homolog 1 (drosophila) Stard3nl - stard3 n-terminal like H2-DMa - histocompatibility 2, class ii, locus dma Cd68 - cd68 antigen Sec24c - sec24 related gene family, member c (s. cerevisiae) H2-DMb2 - histocompatibility 2, class ii, locus mb2 Vps39 - vacuolar protein sorting 39 (yeast) H2-DMb1 - histocompatibility 2, class ii, locus mb1 Ctss - cathepsin s C2cd5 - c2 calcium-dependent domain containing 5 Fth1 - ferritin heavy chain 1 |
| GO:0042612 | MHC class I protein complex | 1.47E-5 | 1.62E-3 | 20.75 (6008,6,193,4) | H2-D1 - histocompatibility 2, d region locus 1 B2m - beta-2 microglobulin H2-L - histocompatibility 2, d region locus l H2-K1 - histocompatibility 2, k1, k region |
| GO:0031902 | late endosome membrane | 1.7E-5 | 1.76E-3 | 6.73 (6008,37,193,8) | Anxa6 - annexin a6 Cd68 - cd68 antigen H2-DMa - histocompatibility 2, class ii, locus dma Stard3nl - stard3 n-terminal like H2-DMb2 - histocompatibility 2, class ii, locus mb2 H2-Eb1 - histocompatibility 2, class ii antigen e beta H2-DMb1 - histocompatibility 2, class ii, locus mb1 Vps39 - vacuolar protein sorting 39 (yeast) |
| GO:0005773 | vacuole | 3.29E-5 | 3.2E-3 | 2.67 (6008,245,193,21) | Slc29a3 - solute carrier family 29 (nucleoside transporters), member 3 H2-Aa - histocompatibility 2, class ii antigen a, alpha Pon2 - paraoxonase 2 Gm2a - gm2 ganglioside activator protein Asah1 - n-acylsphingosine amidohydrolase 1 Epdr1 - ependymin related protein 1 (zebrafish) Psap - prosaposin Cd74 - cd74 antigen (invariant polypeptide of major histocompatibility complex, class ii antigen-associated) Sgsh - n-sulfoglucosamine sulfohydrolase (sulfamidase) Tmem106b - transmembrane protein 106b Slc15a4 - solute carrier family 15, member 4 Loh12cr1 - loss of heterozygosity, 12, chromosomal region 1 homolog (human) Atp6v0c - atpase, h+ transporting, lysosomal v0 subunit c Tecpr1 - tectonin beta-propeller repeat containing 1 H2-DMa - histocompatibility 2, class ii, locus dma Cd68 - cd68 antigen H2-DMb2 - histocompatibility 2, class ii, locus mb2 Vps39 - vacuolar protein sorting 39 (yeast) H2-DMb1 - histocompatibility 2, class ii, locus mb1 Ctss - cathepsin s Fth1 - ferritin heavy chain 1 |
| GO:0098552 | side of membrane | 4.95E-5 | 4.55E-3 | 3.43 (6008,127,193,14) | Lgals3 - lectin, galactose binding, soluble 3 H2-D1 - histocompatibility 2, d region locus 1 H2-Ab1 - histocompatibility 2, class ii antigen a, beta 1 H2-Aa - histocompatibility 2, class ii antigen a, alpha Gm2a - gm2 ganglioside activator protein B2m - beta-2 microglobulin H2-L - histocompatibility 2, d region locus l H2-K1 - histocompatibility 2, k1, k region Myh10 - myosin, heavy polypeptide 10, non-muscle Cd74 - cd74 antigen (invariant polypeptide of major histocompatibility complex, class ii antigen-associated) Jup - junction plakoglobin Loh12cr1 - loss of heterozygosity, 12, chromosomal region 1 homolog (human) Dlg1 - discs, large homolog 1 (drosophila) Ccr1 - chemokine (c-c motif) receptor 1 |
| GO:0000786 | nucleosome | 8.44E-5 | 7.35E-3 | 7.78 (6008,24,193,6) | Hist1h2af - histone cluster 1, h2af H2afj - h2a histone family, member j Hist2h2ac - histone cluster 2, h2ac Hist3h2a - histone cluster 3, h2a Hist1h2ak - histone cluster 1, h2ak Hist1h2ah - histone cluster 1, h2ah |
| GO:0031012 | extracellular matrix | 1.15E-4 | 9.55E-3 | 3.85 (6008,89,193,11) | Tgm2 - transglutaminase 2, c polypeptide Lgals3 - lectin, galactose binding, soluble 3 Notch2 - notch 2 Anxa6 - annexin a6 Itih4 - inter alpha-trypsin inhibitor, heavy chain 4 Itih1 - inter-alpha trypsin inhibitor, heavy chain 1 Alb - albumin S100a11 - s100 calcium binding protein a11 (calgizzarin) Dlg1 - discs, large homolog 1 (drosophila) Angpt1 - angiopoietin 1 Mmp8 - matrix metallopeptidase 8 |
| GO:0005886 | plasma membrane | 1.57E-4 | 1.24E-2 | 1.57 (6008,1128,193,57) | Mtmr9 - myotubularin related protein 9 Stxbp5 - syntaxin binding protein 5 (tomosyn) Pon2 - paraoxonase 2 Magt1 - magnesium transporter 1 Ddx58 - dead (asp-glu-ala-asp) box polypeptide 58 Fam129b - family with sequence similarity 129, member b Cd74 - cd74 antigen (invariant polypeptide of major histocompatibility complex, class ii antigen-associated) Prkcb - protein kinase c, beta Jup - junction plakoglobin Sgpp1 - sphingosine-1-phosphate phosphatase 1 Loh12cr1 - loss of heterozygosity, 12, chromosomal region 1 homolog (human) Emb - embigin Cd68 - cd68 antigen C2cd5 - c2 calcium-dependent domain containing 5 Cp - ceruloplasmin Nedd4l - neural precursor cell expressed, developmentally down-regulated gene 4-like Lsp1 - lymphocyte specific 1 C5ar1 - complement component 5a receptor 1 Fmnl1 - formin-like 1 Gng5 - guanine nucleotide binding protein (g protein), gamma 5 Mpp1 - membrane protein, palmitoylated Notch2 - notch 2 Itih4 - inter alpha-trypsin inhibitor, heavy chain 4 Mcts1 - malignant t cell amplified sequence 1 Itpr2 - inositol 1,4,5-triphosphate receptor 2 Stxbp6 - syntaxin binding protein 6 (amisyn) Stat2 - signal transducer and activator of transcription 2 Slc29a3 - solute carrier family 29 (nucleoside transporters), member 3 Rhbdf2 - rhomboid 5 homolog 2 (drosophila) Anxa6 - annexin a6 Lin7c - lin-7 homolog c (c. elegans) Card11 - caspase recruitment domain family, member 11 Fgr - gardner-rasheed feline sarcoma viral (fgr) oncogene homolog Tmed1 - transmembrane emp24 domain containing 1 Dsp - desmoplakin Ptprs - protein tyrosine phosphatase, receptor type, s Dcxr - dicarbonyl l-xylulose reductase Ltf - lactotransferrin Prrc2a - proline-rich coiled-coil 2a Dlg1 - discs, large homolog 1 (drosophila) Hip1r - huntingtin interacting protein 1 related Atp11b - atpase, class vi, type 11b H2-D1 - histocompatibility 2, d region locus 1 H2-Ab1 - histocompatibility 2, class ii antigen a, beta 1 H2-Aa - histocompatibility 2, class ii antigen a, alpha Inppl1 - inositol polyphosphate phosphatase-like 1 B2m - beta-2 microglobulin Pkn2 - protein kinase n2 Lpcat1 - lysophosphatidylcholine acyltransferase 1 H2-K1 - histocompatibility 2, k1, k region Ap2a2 - adaptor-related protein complex 2, alpha 2 subunit Il1r2 - interleukin 1 receptor, type ii Tgm2 - transglutaminase 2, c polypeptide Myof - myoferlin Prkar2a - protein kinase, camp dependent regulatory, type ii alpha Angpt1 - angiopoietin 1 Ccr1 - chemokine (c-c motif) receptor 1 |
| GO:0044815 | DNA packaging complex | 4.58E-4 | 3.45E-2 | 5.84 (6008,32,193,6) | Hist1h2af - histone cluster 1, h2af Hist2h2ac - histone cluster 2, h2ac H2afj - h2a histone family, member j Hist3h2a - histone cluster 3, h2a Hist1h2ak - histone cluster 1, h2ak Hist1h2ah - histone cluster 1, h2ah |
| GO:0062023 | collagen-containing extracellular matrix | 8.9E-4 | 6.4E-2 | 3.55 (6008,79,193,9) | Tgm2 - transglutaminase 2, c polypeptide Lgals3 - lectin, galactose binding, soluble 3 Anxa6 - annexin a6 Itih4 - inter alpha-trypsin inhibitor, heavy chain 4 Itih1 - inter-alpha trypsin inhibitor, heavy chain 1 Alb - albumin S100a11 - s100 calcium binding protein a11 (calgizzarin) Dlg1 - discs, large homolog 1 (drosophila) Angpt1 - angiopoietin 1 |

Differentially expressed RNA was ranked according to the p-values of differential expression and degree of enrichment compared with the total number of expressed genes analysed (6008 GO terms). The GOrilla database updated on Mar 6, 2021 was used.

**'P-value'** is the enrichment p-value computed according to the mHG or HG model. This p-value is not corrected for multiple testing of 1655 GO terms.

**'FDR q-value'** is the correction of the above p-value for multiple testing using the Benjamini and Hochberg (1995) method.

Namely, for the ith term (ranked according to p-value) the FDR q-value is (p-value * number of GO terms) / i.

**Enrichment (N, B, n, b)** is defined as follows:

N - is the total number of genes

B - is the total number of genes associated with a specific GO term

n - is the number of genes in the top of the user's input list or in the target set when appropriate b - is the number of genes in the intersection

Enrichment = (b/n) / (B/N)

**Genes:** For each GO term you can see the list of associated genes that appear in the optimal top of the list. Each gene name is specified by gene symbol followed by a short description of the gene.
