# Supplementary material for: Proteomic and transcriptomic characterisation of FIA10, a novel murine leukemic cell line that metastasizes into the brain
Source: PLoS One. 2024 Jan 12;19(1):e0295641. doi: 10.1371/journal.pone.0295641 (PMC10786371; doi:10.1371/journal.pone.0295641)
Supplement: S12 Table — (DOCX) [file pone.0295641.s017.docx]

**Gene Ontology: Biological Process FIA10 vs FIA18 RNA downregulated**

| **GO term** | **Description** | [**P-value**](http://cbl-gorilla.cs.technion.ac.il/GOrilla/oq8hw4z5/GOResultsPROCESS.html#p_value_info) | [**FDR q-value**](http://cbl-gorilla.cs.technion.ac.il/GOrilla/oq8hw4z5/GOResultsPROCESS.html#fdr_info) | [**Enrichment (N, B, n, b)**](http://cbl-gorilla.cs.technion.ac.il/GOrilla/oq8hw4z5/GOResultsPROCESS.html#enrich_info) | **Genes** |
| --- | --- | --- | --- | --- | --- |
| [GO:0006954](http://www.godatabase.org/cgi-bin/amigo/go.cgi?query=GO:0006954&view=details) | inflammatory response | 5.84E-6 | 8.96E-2 | 4.53 (15727,389,116,13) | Dab2ip - disabled 2 interacting protein Nupr1 - nuclear protein transcription regulator 1 Ptgs1 - prostaglandin-endoperoxide synthase 1 Lbp - lipopolysaccharide binding protein Itgb2l - integrin beta 2-like Elane - elastase, neutrophil expressed Il1rap - interleukin 1 receptor accessory protein Stab1 - stabilin 1 Fcgr1 - fc receptor, igg, high affinity i Thbs1 - thrombospondin 1 Axl - axl receptor tyrosine kinase Pf4 - platelet factor 4 Ppbp - pro-platelet basic protein |
| [GO:0006952](http://www.godatabase.org/cgi-bin/amigo/go.cgi?query=GO:0006952&view=details) | defense response | 2.46E-5 | 1.89E-1 | 2.80 (15727,967,116,20) | Dab2ip - disabled 2 interacting protein Fcnb - ficolin b Nupr1 - nuclear protein transcription regulator 1 Sla - src-like adaptor Ptgs1 - prostaglandin-endoperoxide synthase 1 Lbp - lipopolysaccharide binding protein Cd177 - cd177 antigen Itgb2l - integrin beta 2-like Elane - elastase, neutrophil expressed Il1rap - interleukin 1 receptor accessory protein Stab1 - stabilin 1 Fcgr1 - fc receptor, igg, high affinity i Ighm - immunoglobulin heavy constant mu Ear11 - eosinophil-associated, ribonuclease a family, member 11 Thbs1 - thrombospondin 1 Axl - axl receptor tyrosine kinase Ltf - lactotransferrin Ifitm6 - interferon induced transmembrane protein 6 Pf4 - platelet factor 4 Ppbp - pro-platelet basic protein |
| [GO:0006910](http://www.godatabase.org/cgi-bin/amigo/go.cgi?query=GO:0006910&view=details) | phagocytosis, recognition | 2.66E-5 | 1.36E-1 | 22.60 (15727,24,116,4) | Fcnb - ficolin b Fcgr1 - fc receptor, igg, high affinity i Ighm - immunoglobulin heavy constant mu Scarb1 - scavenger receptor class b, member 1 |
| [GO:0015920](http://www.godatabase.org/cgi-bin/amigo/go.cgi?query=GO:0015920&view=details) | lipopolysaccharide transport | 5.39E-5 | 2.07E-1 | 135.58 (15727,2,116,2) | Lbp - lipopolysaccharide binding protein Scarb1 - scavenger receptor class b, member 1 |
| [GO:0010896](http://www.godatabase.org/cgi-bin/amigo/go.cgi?query=GO:0010896&view=details) | regulation of triglyceride catabolic process | 1.67E-4 | 5.12E-1 | 27.12 (15727,15,116,3) | Apoc2 - apolipoprotein c-ii Abhd5 - abhydrolase domain containing 5 Sorl1 - sortilin-related receptor,  ldlr class a repeats-containing |
| [GO:1903725](http://www.godatabase.org/cgi-bin/amigo/go.cgi?query=GO:1903725&view=details) | regulation of phospholipid metabolic process | 2.02E-4 | 5.16E-1 | 9.29 (15727,73,116,5) | Dab2ip - disabled 2 interacting protein Apoc2 - apolipoprotein c-ii Cd81 - cd81 antigen Capn2 - calpain 2 Scarb1 - scavenger receptor class b, member 1 |
| [GO:0045087](http://www.godatabase.org/cgi-bin/amigo/go.cgi?query=GO:0045087&view=details) | innate immune response | 2.8E-4 | 6.15E-1 | 3.54 (15727,421,116,11) | Dab2ip - disabled 2 interacting protein Fcnb - ficolin b Il1rap - interleukin 1 receptor accessory protein Sla - src-like adaptor Fcgr1 - fc receptor, igg, high affinity i Ighm - immunoglobulin heavy constant mu Lbp - lipopolysaccharide binding protein Ear11 - eosinophil-associated, ribonuclease a family, member 11 Axl - axl receptor tyrosine kinase Ltf - lactotransferrin Cd177 - cd177 antigen |
| [GO:0000720](http://www.godatabase.org/cgi-bin/amigo/go.cgi?query=GO:0000720&view=details) | pyrimidine dimer repair by nucleotide-excision repair | 3.21E-4 | 6.15E-1 | 67.79 (15727,4,116,2) | Hmgn1 - high mobility group nucleosomal binding domain 1 Xpc - xeroderma pigmentosum, complementation group c |
| [GO:2000563](http://www.godatabase.org/cgi-bin/amigo/go.cgi?query=GO:2000563&view=details) | positive regulation of CD4-positive, alpha-beta T cell proliferation | 3.21E-4 | 5.47E-1 | 67.79 (15727,4,116,2) | Cd81 - cd81 antigen Ceacam1 - carcinoembryonic antigen-related cell adhesion molecule 1 |
| [GO:0034143](http://www.godatabase.org/cgi-bin/amigo/go.cgi?query=GO:0034143&view=details) | regulation of toll-like receptor 4 signaling pathway | 3.48E-4 | 5.34E-1 | 21.41 (15727,19,116,3) | Dab2ip - disabled 2 interacting protein Lbp - lipopolysaccharide binding protein Ltf - lactotransferrin |
| [GO:0002684](http://www.godatabase.org/cgi-bin/amigo/go.cgi?query=GO:0002684&view=details) | positive regulation of immune system process | 3.74E-4 | 5.23E-1 | 2.62 (15727,828,116,16) | Fcnb - ficolin b Lbp - lipopolysaccharide binding protein Cd177 - cd177 antigen Elane - elastase, neutrophil expressed Mmp9 - matrix metallopeptidase 9 Fcgr1 - fc receptor, igg, high affinity i Cd81 - cd81 antigen Ighm - immunoglobulin heavy constant mu Thbs1 - thrombospondin 1 Axl - axl receptor tyrosine kinase Ltf - lactotransferrin Ceacam1 - carcinoembryonic antigen-related cell adhesion molecule 1 Tox - thymocyte selection-associated high mobility group box F7 - coagulation factor vii Kcnn4 - potassium intermediate/small conductance calcium-activated channel, subfamily n, member 4 Pf4 - platelet factor 4 |
| [GO:0051704](http://www.godatabase.org/cgi-bin/amigo/go.cgi?query=GO:0051704&view=details) | multi-organism process | 4.6E-4 | 5.89E-1 | 2.48 (15727,930,116,17) | Slfn4 - schlafen 4 Lbp - lipopolysaccharide binding protein Hba-a1 - hemoglobin alpha, adult chain 1 Elane - elastase, neutrophil expressed Mmp9 - matrix metallopeptidase 9 Pla2g16 - phospholipase a2, group xvi Stab1 - stabilin 1 Fcgr1 - fc receptor, igg, high affinity i Etf1 - eukaryotic translation termination factor 1 Ighm - immunoglobulin heavy constant mu Axl - axl receptor tyrosine kinase Ltf - lactotransferrin Ifitm6 - interferon induced transmembrane protein 6 Scarb1 - scavenger receptor class b, member 1 Ceacam1 - carcinoembryonic antigen-related cell adhesion molecule 1 Pf4 - platelet factor 4 Ppbp - pro-platelet basic protein |
| [GO:0090207](http://www.godatabase.org/cgi-bin/amigo/go.cgi?query=GO:0090207&view=details) | regulation of triglyceride metabolic process | 4.61E-4 | 5.44E-1 | 11.07 (15727,49,116,4) | Apoc2 - apolipoprotein c-ii Sorl1 - sortilin-related receptor,  ldlr class a repeats-containing Abhd5 - abhydrolase domain containing 5 Scarb1 - scavenger receptor class b, member 1 |
| [GO:0051817](http://www.godatabase.org/cgi-bin/amigo/go.cgi?query=GO:0051817&view=details) | modification of morphology or physiology of other organism involved in symbiotic interaction | 4.82E-4 | 5.28E-1 | 7.70 (15727,88,116,5) | Ltf - lactotransferrin Ceacam1 - carcinoembryonic antigen-related cell adhesion molecule 1 Elane - elastase, neutrophil expressed Pf4 - platelet factor 4 Mmp9 - matrix metallopeptidase 9 |
| [GO:0043654](http://www.godatabase.org/cgi-bin/amigo/go.cgi?query=GO:0043654&view=details) | recognition of apoptotic cell | 5.32E-4 | 5.44E-1 | 54.23 (15727,5,116,2) | Fcnb - ficolin b Scarb1 - scavenger receptor class b, member 1 |
| [GO:0060696](http://www.godatabase.org/cgi-bin/amigo/go.cgi?query=GO:0060696&view=details) | regulation of phospholipid catabolic process | 5.32E-4 | 5.1E-1 | 54.23 (15727,5,116,2) | Apoc2 - apolipoprotein c-ii Scarb1 - scavenger receptor class b, member 1 |
| [GO:0002523](http://www.godatabase.org/cgi-bin/amigo/go.cgi?query=GO:0002523&view=details) | leukocyte migration involved in inflammatory response | 5.44E-4 | 4.91E-1 | 18.49 (15727,22,116,3) | Lbp - lipopolysaccharide binding protein Elane - elastase, neutrophil expressed Ppbp - pro-platelet basic protein |
| [GO:0043207](http://www.godatabase.org/cgi-bin/amigo/go.cgi?query=GO:0043207&view=details) | response to external biotic stimulus | 6.16E-4 | 5.25E-1 | 2.60 (15727,781,116,15) | Dab2ip - disabled 2 interacting protein Slfn4 - schlafen 4 Lbp - lipopolysaccharide binding protein Hba-a1 - hemoglobin alpha, adult chain 1 Elane - elastase, neutrophil expressed Pla2g16 - phospholipase a2, group xvi Stab1 - stabilin 1 Fcgr1 - fc receptor, igg, high affinity i Ighm - immunoglobulin heavy constant mu Axl - axl receptor tyrosine kinase Ltf - lactotransferrin Ifitm6 - interferon induced transmembrane protein 6 Scarb1 - scavenger receptor class b, member 1 Pf4 - platelet factor 4 Ppbp - pro-platelet basic protein |
| [GO:0070861](http://www.godatabase.org/cgi-bin/amigo/go.cgi?query=GO:0070861&view=details) | regulation of protein exit from endoplasmic reticulum | 6.22E-4 | 5.02E-1 | 17.68 (15727,23,116,3) | Cd81 - cd81 antigen Svip - small vcp/p97-interacting protein Sorl1 - sortilin-related receptor, ldlr class a repeats-containing |
| [GO:1903900](http://www.godatabase.org/cgi-bin/amigo/go.cgi?query=GO:1903900&view=details) | regulation of viral life cycle | 6.9E-4 | 5.29E-1 | 5.65 (15727,144,116,6) | Fcnb - ficolin b Tarbp2 - tar (hiv) rna binding protein 2 Axl - axl receptor tyrosine kinase Ltf - lactotransferrin Ifitm6 - interferon induced transmembrane protein 6 Ceacam1 - carcinoembryonic antigen-related cell adhesion molecule 1 |
| [GO:0006955](http://www.godatabase.org/cgi-bin/amigo/go.cgi?query=GO:0006955&view=details) | immune response | 7.11E-4 | 5.2E-1 | 2.57 (15727,792,116,15) | Dab2ip - disabled 2 interacting protein Fcnb - ficolin b Sla - src-like adaptor Lbp - lipopolysaccharide binding protein Cd177 - cd177 antigen Elane - elastase, neutrophil expressed Il1rap - interleukin 1 receptor accessory protein Fcgr1 - fc receptor, igg, high affinity i Cd81 - cd81 antigen Ighm - immunoglobulin heavy constant mu Ear11 - eosinophil-associated, ribonuclease a family, member 11 Axl - axl receptor tyrosine kinase Ltf - lactotransferrin Pf4 - platelet factor 4 Ppbp - pro-platelet basic protein |
| [GO:0045833](http://www.godatabase.org/cgi-bin/amigo/go.cgi?query=GO:0045833&view=details) | negative regulation of lipid metabolic process | 7.52E-4 | 5.25E-1 | 6.99 (15727,97,116,5) | Dab2ip - disabled 2 interacting protein Apoc2 - apolipoprotein c-ii Sorl1 - sortilin-related receptor, ldlr class a repeats-containing Ceacam1 - carcinoembryonic antigen-related cell adhesion molecule 1 Trib3 - tribbles homolog 3 (drosophila) |
| [GO:0002682](http://www.godatabase.org/cgi-bin/amigo/go.cgi?query=GO:0002682&view=details) | regulation of immune system process | 7.89E-4 | 5.27E-1 | 2.17 (15727,1252,116,20) | Dab2ip - disabled 2 interacting protein Fcnb - ficolin b Hist1h4i - histone cluster 1, h4i Lbp - lipopolysaccharide binding protein Mtus1 - mitochondrial tumor suppressor 1 Cd177 - cd177 antigen Elane - elastase, neutrophil expressed Mmp9 - matrix metallopeptidase 9 Fcgr1 - fc receptor, igg, high affinity i Tarbp2 - tar (hiv) rna binding protein 2 Cd81 - cd81 antigen Ighm - immunoglobulin heavy constant mu Thbs1 - thrombospondin 1 Axl - axl receptor tyrosine kinase Ltf - lactotransferrin Ceacam1 - carcinoembryonic antigen-related cell adhesion molecule 1 Tox - thymocyte selection-associated high mobility group box F7 - coagulation factor vii Kcnn4 - potassium intermediate/small conductance calcium-activated channel, subfamily n, member 4 Pf4 - platelet factor 4 |
| [GO:0034384](http://www.godatabase.org/cgi-bin/amigo/go.cgi?query=GO:0034384&view=details) | high-density lipoprotein particle clearance | 7.94E-4 | 5.08E-1 | 45.19 (15727,6,116,2) | Apoc2 - apolipoprotein c-ii Scarb1 - scavenger receptor class b, member 1 |
| [GO:0002455](http://www.godatabase.org/cgi-bin/amigo/go.cgi?query=GO:0002455&view=details) | humoral immune response mediated by circulating immunoglobulin | 7.94E-4 | 4.87E-1 | 45.19 (15727,6,116,2) | Cd81 - cd81 antigen Ighm - immunoglobulin heavy constant mu |
| [GO:0086073](http://www.godatabase.org/cgi-bin/amigo/go.cgi?query=GO:0086073&view=details) | bundle of His cell-Purkinje myocyte adhesion involved in cell communication | 7.94E-4 | 4.69E-1 | 45.19 (15727,6,116,2) | Dsp - desmoplakin Pkp2 - plakophilin 2 |
| [GO:0016064](http://www.godatabase.org/cgi-bin/amigo/go.cgi?query=GO:0016064&view=details) | immunoglobulin mediated immune response | 7.99E-4 | 4.54E-1 | 16.27 (15727,25,116,3) | Fcgr1 - fc receptor, igg, high affinity i Ighm - immunoglobulin heavy constant mu Cd81 - cd81 antigen |
| [GO:0030334](http://www.godatabase.org/cgi-bin/amigo/go.cgi?query=GO:0030334&view=details) | regulation of cell migration | 8.29E-4 | 4.55E-1 | 2.43 (15727,891,116,16) | Dab2ip - disabled 2 interacting protein Glipr2 - gli pathogenesis-related 2 Lbp - lipopolysaccharide binding protein Mtus1 - mitochondrial tumor suppressor 1 Sorl1 - sortilin-related receptor, ldlr class a repeats-containing Elane - elastase, neutrophil expressed Phlda2 - pleckstrin homology-like domain, family a, member 2 Mmp9 - matrix metallopeptidase 9 Arid2 - at rich interactive domain 2 (arid, rfx-like) Serpinf1 - serine (or cysteine) peptidase inhibitor, clade f, member 1 Cd81 - cd81 antigen Pkp2 - plakophilin 2 Thbs1 - thrombospondin 1 Diap1 - diaphanous homolog 1 (drosophila) Ceacam1 - carcinoembryonic antigen-related cell adhesion molecule 1 F7 - coagulation factor vii |
| [GO:0002685](http://www.godatabase.org/cgi-bin/amigo/go.cgi?query=GO:0002685&view=details) | regulation of leukocyte migration | 8.35E-4 | 4.42E-1 | 4.61 (15727,206,116,7) | Lbp - lipopolysaccharide binding protein Cd81 - cd81 antigen Mtus1 - mitochondrial tumor suppressor 1 Thbs1 - thrombospondin 1 F7 - coagulation factor vii Elane - elastase, neutrophil expressed Mmp9 - matrix metallopeptidase 9 |
| [GO:0009607](http://www.godatabase.org/cgi-bin/amigo/go.cgi?query=GO:0009607&view=details) | response to biotic stimulus | 8.4E-4 | 4.3E-1 | 2.53 (15727,805,116,15) | Dab2ip - disabled 2 interacting protein Slfn4 - schlafen 4 Lbp - lipopolysaccharide binding protein Hba-a1 - hemoglobin alpha, adult chain 1 Elane - elastase, neutrophil expressed Pla2g16 - phospholipase a2, group xvi Stab1 - stabilin 1 Fcgr1 - fc receptor, igg, high affinity i Ighm - immunoglobulin heavy constant mu Axl - axl receptor tyrosine kinase Ltf - lactotransferrin Ifitm6 - interferon induced transmembrane protein 6 Scarb1 - scavenger receptor class b, member 1 Pf4 - platelet factor 4 Ppbp - pro-platelet basic protein |
| [GO:0098901](http://www.godatabase.org/cgi-bin/amigo/go.cgi?query=GO:0098901&view=details) | regulation of cardiac muscle cell action potential | 8.98E-4 | 4.45E-1 | 15.64 (15727,26,116,3) | Dsp - desmoplakin Rangrf - ran guanine nucleotide release factor Pkp2 - plakophilin 2 |
| [GO:0002526](http://www.godatabase.org/cgi-bin/amigo/go.cgi?query=GO:0002526&view=details) | acute inflammatory response | 9.36E-4 | 4.49E-1 | 9.19 (15727,59,116,4) | Nupr1 - nuclear protein transcription regulator 1 Fcgr1 - fc receptor, igg, high affinity i Lbp - lipopolysaccharide binding protein Elane - elastase, neutrophil expressed |

Differentially expressed RNA was ranked according to the p-values of differential expression and degree of enrichment compared with the total number of expressed genes analysed (15727 GO terms). The GOrilla database updated on Mar 6, 2021 was used.

**'P-value'** is the enrichment p-value computed according to the mHG or HG model. This p-value is not corrected for multiple testing of 15353 GO terms.

**'FDR q-value'** is the correction of the above p-value for multiple testing using the Benjamini and Hochberg (1995) method.

Namely, for the ith term (ranked according to p-value) the FDR q-value is (p-value * number of GO terms) / i.

**Enrichment (N, B, n, b)** is defined as follows:

N - is the total number of genes

B - is the total number of genes associated with a specific GO term

n - is the number of genes in the top of the user's input list or in the target set when appropriate b - is the number of genes in the intersection

Enrichment = (b/n) / (B/N)

**Genes:** For each GO term you can see the list of associated genes that appear in the optimal top of the list. Each gene name is specified by gene symbol followed by a short description of the gene.
