# Supplementary material for: Proteomic and transcriptomic characterisation of FIA10, a novel murine leukemic cell line that metastasizes into the brain
Source: PLoS One. 2024 Jan 12;19(1):e0295641. doi: 10.1371/journal.pone.0295641 (PMC10786371; doi:10.1371/journal.pone.0295641)
Supplement: S13 Table — (DOCX) [file pone.0295641.s018.docx]

**Gene Ontology: Molecular function FIA10 vs FIA18 RNA downregulated**

| **GO term** | **Description** | **P-value** | **FDR q-value** | **Enrichment (N, B, n, b)** | **Genes** |
| --- | --- | --- | --- | --- | --- |

| GO:0030169 | low-density lipoprotein particle binding | 7.94E-6 | 3.61E-2 | 30.13 (15727,18,116,4) | Stab1 - stabilin 1  Sorl1 - sortilin- related receptor, ldlr class a repeats-containing  Thbs1 - thrombospondin 1  Scarb1 - scavenger receptor class b, member 1 |
| --- | --- | --- | --- | --- | --- |
| GO:0071813 | lipoprotein particle binding | 5.79E-5 | 1.31E-1 | 18.70 (15727,29,116,4) | Stab1 - stabilin 1  Sorl1 - sortilin- related receptor, ldlr class a repeats-containing  Thbs1 - thrombospondin 1  Scarb1 - scavenger receptor class b, member 1 |
| GO:0071814 | protein-lipid complex binding | 5.79E-5 | 8.76E-2 | 18.70 (15727,29,116,4) | Stab1 - stabilin 1  Sorl1 - sortilin- related receptor, ldlr class a repeats-containing  Thbs1 - thrombospondin 1  Scarb1 - scavenger receptor class b, member 1 |
| GO:0044877 | protein-containing complex binding | 1.39E-4 | 1.26E-1 | 2.28 (15727,1366,116,23) | Dab2ip - disabled 2 interacting protein  Hmgn1 - high mobility group nucleosomal binding domain 1 Sorl1 - sortilin- related receptor, ldlr class a repeats-containing  Wiz - widely- interspaced zinc finger motifs  Tgfbi - transforming growth factor, beta induced  Xpc - xeroderma pigmentosum, complementation group c  Hba-a1 - hemoglobin alpha, adult chain 1  Cd177 - cd177 antigen  Itgb2l - integrin beta 2-like  Pcolce - procollagen c- endopeptidase enhancer protein  Tspan8 - tetraspanin 8  Mmp9 – matrix metallopeptidase 9  Stab1 - stabilin 1  Fcgr1 - fc receptor, igg, high affinity i  Mtm1 - x-linked myotubular myopathy gene 1  Pkp2 - plakophilin 2  Capn2 - calpain 2  Cd81 - cd81 antigen  Rbm3 - rna binding motif protein 3  Thbs1 -thrombospondin 1  Nebl - nebulette  Scarb1 - scavenger receptor class b, member 1  Coro2a - coronin, actin bindingprotein 2a |
| GO:0005178 | integrin binding | 4.01E-4 | 3.04E-1 | 6.26 (15727,130,116,6) | Cd81 - cd81 antigen  Thbs1 -thrombospondin 1  Tgfbi - transforming growth factor, beta induced  Cd177 - cd177antigen  Itgb2l - integrin beta 2-like  Tspan8 - tetraspanin 8 |

|  |  |  |  |  |  |
| --- | --- | --- | --- | --- | --- |
| GO:0050839 | cell adhesion molecule binding | 7.61E-5 | 8.64E-2 | 5.04 (15727,242,116,9) | Dsp – desmoplakin  Cd81 - cd81 antigen  Pkp2 - plakophilin 2  Thbs1 - thrombospondin 1  Tgfbi - transforming growth factor, beta induced  Ndrg1 - n-myc downstream regulated gene 1  Cd177 - cd177 antigen  Itgb2l - integrin beta 2-like  Tspan8 - tetraspanin 8 |
| GO:0001530 | lipopolysaccharide binding | 8.98E-4 | 5.83E-1 | 15.64 (15727,26,116,3) | Lbp - lipopolysaccharide binding protein  Ltf - lactotransferrin  Scarb1 - scavenger receptor class b, member 1 |

Differentially expressed RNA was ranked according to the p-values of differential expression and degree of enrichment compared with the total number of expressed genes analysed (15727 GO terms). The GOrilla database updated on Mar 6, 2021 was used.

**'P-value'** is the enrichment p-value computed according to the mHG or HG model. This p-value is not corrected for multiple testing of 4542 GO terms.

**'FDR q-value'** is the correction of the above p-value for multiple testing using the Benjamini and Hochberg (1995) method. Namely, for the ith term (ranked according to p-value) the FDR q-value is (p-value * number of GO terms) / i.

**Enrichment (N, B, n, b)** is defined as follows:

N - is the total number of genes

B - is the total number of genes associated with a specific GO term

n - is the number of genes in the top of the user's input list or in the target set when appropriate b - is the number of genes in the intersection

Enrichment = (b/n) / (B/N)

**Genes:** For each GO term you can see the list of associated genes that appear in the optimal top of the list. Each gene name is specified by gene symbol followed by a short description of the gene
