# Supplementary material for: Proteomic and transcriptomic characterisation of FIA10, a novel murine leukemic cell line that metastasizes into the brain
Source: PLoS One. 2024 Jan 12;19(1):e0295641. doi: 10.1371/journal.pone.0295641 (PMC10786371; doi:10.1371/journal.pone.0295641)
Supplement: S14 Table — (DOCX) [file pone.0295641.s019.docx]

**Gene Ontology: Cellular component FIA10 vs FIA18 RNA downregulated**

| **GO term** | **Description** | **P-value** | **FDR q-value** | **Enrichment (N, B, n, b)** | **Genes** |
| --- | --- | --- | --- | --- | --- |
| GO:0044421 | extracellular region part | 3.24E-4 | 6.28E-1 | 2.07 (15727,1641,116,25) | Lbp - lipopolysaccharide binding protein  Tgfbi - transforming growth factor, beta induced  Elane - elastase, neutrophil expressed  Pcolce - procollagen c-endopeptidase enhancer protein  Serpinf1 - serine (or cysteine) peptidase inhibitor, clade f, member 1  Cd81 - cd81 antigen  Axl - axl receptor tyrosine kinase  Ltf - lactotransferrin  Ceacam1 - carcinoembryonic antigen-related cell adhesion molecule 1  Olfml2b - olfactomedin-like 2b  Glipr2 - gli pathogenesis-related 2  Fcnb - ficolin b  Nupr1 - nuclear protein transcription regulator 1  C1qtnf6 - c1q and tumor necrosis factor related protein 6  Sorl1 - sortilin-related receptor, ldlr class a repeats-containing  Hba-a1 - hemoglobin alpha, adult chain 1  Mmp9 - matrix metallopeptidase 9  Scnn1a - sodium channel, nonvoltage-gated 1 alpha  Apoc2 - apolipoprotein c-ii  Ighm - immunoglobulin heavy constant mu  Thbs1 - thrombospondin 1  Ear11 - eosinophil-associated, ribonuclease a family, member 11  F7 - coagulation factor vii  Pf4 - platelet factor 4  Ppbp - pro-platelet basic protein |
| GO:0005615 | extracellular space | 5.37E-4 | 5.2E-1 | 2.18 (15727,1308,116,21) | Glipr2 - gli pathogenesis-related 2  Fcnb - ficolin b  C1qtnf6 - c1q and tumor necrosis factor related protein 6  Lbp - lipopolysaccharide binding protein  Sorl1 - sortilin-related receptor, ldlr class a repeats-containing  Tgfbi - transforming growth factor, beta induced  Hba-a1 - hemoglobin alpha, adult chain 1  Pcolce - procollagen c-endopeptidase enhancer protein  Elane - elastase, neutrophil expressed  Mmp9 - matrix metallopeptidase 9  Apoc2 - apolipoprotein c-ii  Serpinf1 - serine (or cysteine) peptidase inhibitor, clade f, member 1  Ighm - immunoglobulin heavy constant mu  Ear11 - eosinophil-associated, ribonuclease a family, member 11  Thbs1 - thrombospondin 1  Axl - axl receptor tyrosine kinase  Ltf - lactotransferrin  Ceacam1 - carcinoembryonic antigen-related cell adhesion molecule 1  F7 - coagulation factor vii  Pf4 - platelet factor 4  Ppbp - pro-platelet basic protein |
| GO:0009986 | cell surface | 6.89E-4 | 4.46E-1 | 2.82 (15727,624,116,13) | Lbp - lipopolysaccharide binding protein  Sorl1 - sortilin-related receptor, ldlr class a repeats-containing  Itgb2l - integrin beta 2-like  Elane - elastase, neutrophil expressed  Tspan8 - tetraspanin 8  Ighm - immunoglobulin heavy constant mu  Thbs1 - thrombospondin 1  Axl - axl receptor tyrosine kinase  Ltf - lactotransferrin  Ckap4 - cytoskeleton-associated protein 4  Scarb1 - scavenger receptor class b, member 1  Ceacam1 - carcinoembryonic antigen-related cell adhesion molecule 1  Bace1 - beta-site app cleaving enzyme 1 |

Differentially expressed RNA was ranked according to the p-values of differential expression and degree of enrichment compared with the total number of expressed genes analysed (15727 GO terms). The GOrilla database updated on Mar 6, 2021 was used.

**'P-value'** is the enrichment p-value computed according to the mHG or HG model. This p-value is not corrected for multiple testing of 1940 GO terms.

**'FDR q-value'** is the correction of the above p-value for multiple testing using the Benjamini and Hochberg (1995) method.

Namely, for the ith term (ranked according to p-value) the FDR q-value is (p-value * number of GO terms) / i.

**Enrichment (N, B, n, b)** is defined as follows:

N - is the total number of genes

B - is the total number of genes associated with a specific GO term

n - is the number of genes in the top of the user's input list or in the target set when appropriate b - is the number of genes in the intersection

Enrichment = (b/n) / (B/N)

**Genes:** For each GO term you can see the list of associated genes that appear in the optimal top of the list. Each gene name is specified by gene symbol followed by a short description of the gene.
