# Supplementary material for: Proteomic and transcriptomic characterisation of FIA10, a novel murine leukemic cell line that metastasizes into the brain
Source: PLoS One. 2024 Jan 12;19(1):e0295641. doi: 10.1371/journal.pone.0295641 (PMC10786371; doi:10.1371/journal.pone.0295641)
Supplement: S17 Table — (DOCX) [file pone.0295641.s022.docx]

Gene Ontology: Cellular component FIA10 vs FIA18 Protein downregulated

| **GO term** | **Description** | **P-value** | **FDR q-value** | **Enrichment (N, B, n, b)** | **Genes** |
| --- | --- | --- | --- | --- | --- |
| GO:0044421 | extracellular region part | 9.17E-5 | 1.53E-1 | 2.31 (6384,379,175,24) | Cpa3 - carboxypeptidase a3, mast cell Fcnb - ficolin b  Mcpt8 - mast cell protease 8 Itga2b - integrin alpha 2b  Epx - eosinophil peroxidase  Pycrl - pyrroline-5-carboxylate reductase-like Sra1 - steroid receptor rna activator 1  Ahnak - ahnak nucleoprotein (desmoyokin) Vtn - vitronectin  Elane - elastase, neutrophil expressed  S100a6 - s100 calcium binding protein a6 (calcyclin)  Prtn3 - proteinase 3  Gstm2 - glutathione s-transferase, mu 2 Il1f9 - interleukin 1 family, member 9 Nucb2 - nucleobindin 2  Car2 - carbonic anhydrase 2  Gpt2 - glutamic pyruvate transaminase (alanine aminotransferase) 2  F13a1 - coagulation factor xiii, a1 subunit Cd151 - cd151 antigen |

Differentially expressed RNA was ranked according to the p-values of differential expression and degree of enrichment compared with the total number of expressed genes analysed (6003 GO terms). The GOrilla database updated on Mar 6, 2021 was used.

**'P-value'** is the enrichment p-value computed according to the mHG or HG model. This p-value is not corrected for multiple testing of 1940 GO terms.

**'FDR q-value'** is the correction of the above p-value for multiple testing using the Benjamini and Hochberg (1995) method. Namely, for the ith term (ranked according to p-value) the FDR q-value is (p-value * number of GO terms) / i.

**Enrichment (N, B, n, b)** is defined as follows:

N - is the total number of genes

B - is the total number of genes associated with a specific GO term

n - is the number of genes in the top of the user's input list or in the target set when appropriate b - is the number of genes in the intersection Enrichment = (b/n) / (B/N)

**Genes:** For each GO term you can see the list of associated genes that appear in the optimal top of the list. Each gene name is specified by gene symbol followed by a short description of the gene.
