# Supplementary material for: Proteomic and transcriptomic characterisation of FIA10, a novel murine leukemic cell line that metastasizes into the brain
Source: PLoS One. 2024 Jan 12;19(1):e0295641. doi: 10.1371/journal.pone.0295641 (PMC10786371; doi:10.1371/journal.pone.0295641)
Supplement: S2 Fig — Differentially expressed RNAs were ranked according to their p-values of differential expression and degree of enrichment compared with the total number of expressed genes analysed. (PDF) [file pone.0295641.s002.pdf]

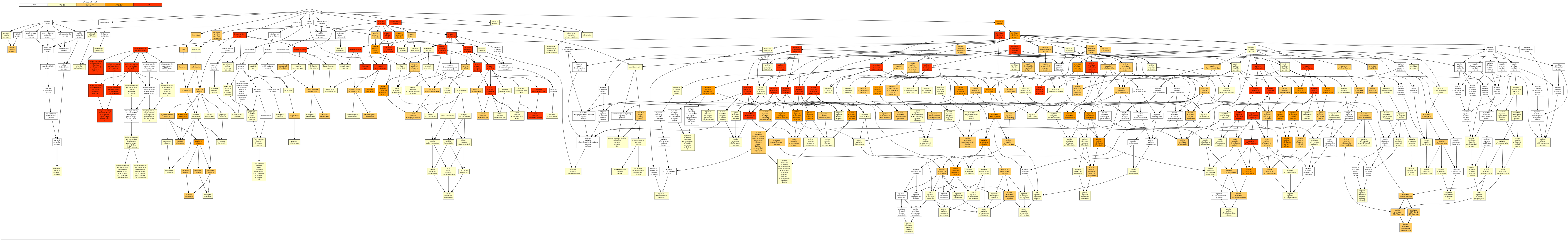
P-value color scale
> 10^{-3}
10^{-3} to 10^{-5}
10^{-5} to 10^{-7}
10^{-7} to 10^{-9}
< 10^{-9}
