# Supplementary material for: Proteomic and transcriptomic characterisation of FIA10, a novel murine leukemic cell line that metastasizes into the brain
Source: PLoS One. 2024 Jan 12;19(1):e0295641. doi: 10.1371/journal.pone.0295641 (PMC10786371; doi:10.1371/journal.pone.0295641)

FIA10 RNA down

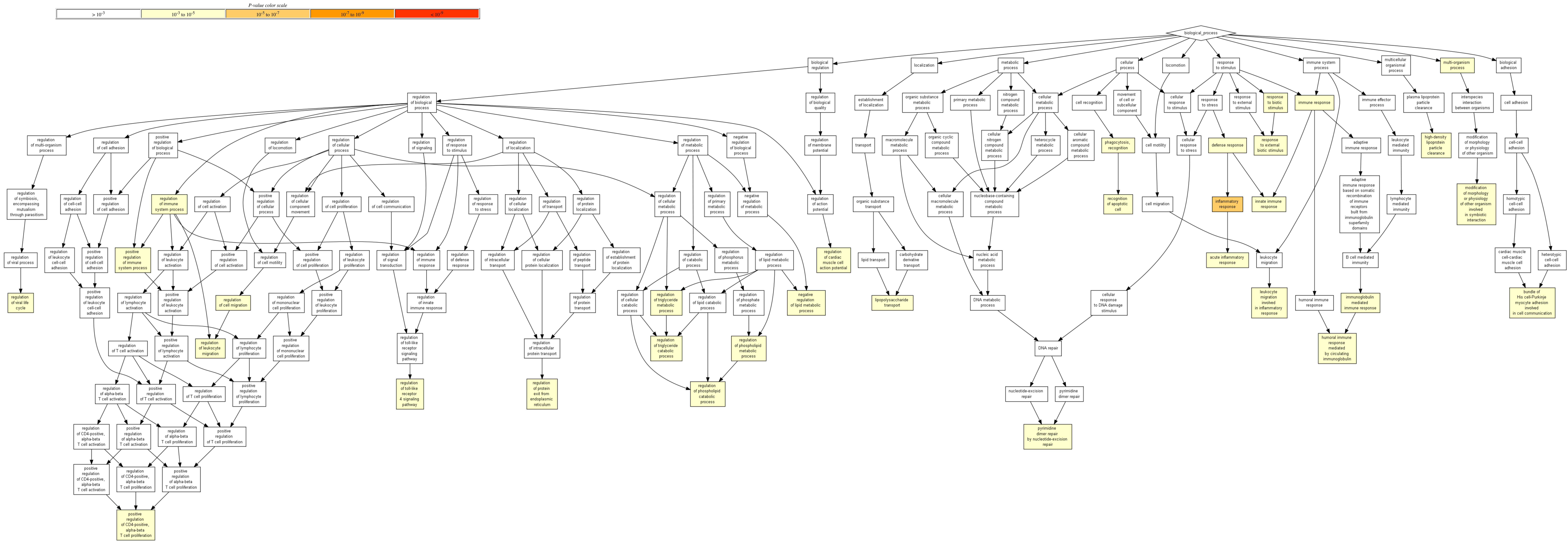

Supplement: S4 Fig — Differentially expressed RNAs were ranked according to their p-values of differential expression and degree of enrichment compared with the total number of expressed genes analysed. (PDF) [file pone.0295641.s004.pdf]
